# Adjuvant Everolimus in Non–Clear Cell Renal Cell Carcinoma: A Secondary Analysis of a Randomized Clinical Trial

**DOI:** 10.1001/jamanetworkopen.2024.25288

**Published:** 2024-08-06

**Authors:** Shuchi Gulati, Catherine Tangen, Christopher W. Ryan, Ulka N. Vaishampayan, Brian M. Shuch, Pedro C. Barata, Deepak K. Pruthi, Cristiane D. Bergerot, Abhishek Tripathi, Seth P. Lerner, Ian M. Thompson, Primo N. Lara, Sumanta K. Pal

**Affiliations:** 1 University of California Davis Comprehensive Cancer Center, Sacramento; 2 SWOG Statistical Center, Seattle, Washington; 3 Oregon Health & Science University, Knight Cancer Institute, Portland; 4 Rogel Cancer Center, University of Michigan, Ann Arbor; 5 University of California, Los Angeles; 6 University Hospitals Seidman Cancer Center, Cleveland, Ohio; 7 University of Texas Health San Antonio, San Antonio; 8 Oncoclinicas, Brasilia, Brazil; 9 City of Hope Comprehensive Cancer Center, Duarte, California; 10 Baylor College of Medicine, Houston, Texas; 11 CHRISTUS Medical Center Hospital, San Antonio, Texas

## Abstract

**Importance:**

Clinical trial data on adjuvant therapy in patients with non–clear cell renal cell carcinoma (RCC) are scant.

**Objective:**

To evaluate the effect of adjuvant everolimus after nephrectomy on recurrence-free survival (RFS) and overall survival (OS) in patients with localized papillary and chromophobe RCC.

**Design, Setting, and Participants:**

This prespecified subgroup analysis of a phase 3 randomized clinical trial, EVEREST, included patients enrolled between April 1, 2011, and September 15, 2016. Eligible patients had fully resected RCC at intermediate-high risk (pT1 grade 3-4, N0 to pT3a grade 1-2, N0) or very-high risk (pT3a grade 3-4 to pT4 any grade or N+) for recurrence who had received radical or partial nephrectomy. Final analyses was completed in March 2022.

**Intervention:**

The intervention group received 54 weeks of everolimus (10 mg orally daily); the control group received a matching placebo.

**Main Outcomes and Measures:**

The main outcomes were RFS, OS, and rates of adverse events. For testing the hazard ratio (HR) for treatment effect, a Cox regression model was used for both OS and RFS.

**Results:**

Of 1545 adult patients with treatment-naive, nonmetastatic, fully resected RCC in EVEREST, 109 had papillary RCC (median [range] age, 60 [19-81] years; 82 [75%] male; 50 patients [46%] with very high–risk disease) and 99 had chromophobe RCC (median [range] age 51 [18-71] years; 53 [54%] male; 34 patients [34%] with very high–risk disease). Among 57 patients with papillary RCC in the intervention group, 26 (46%) completed 54 weeks of treatment, and among 53 patients with chromophobe RCC in the intervention group, 26 (49%) completed 54 weeks of treatment. With a median (IQR) follow-up of 76 (61-96) months, adjuvant everolimus did not improve RFS compared with placebo in either papillary RCC (5-year RFS: 62% vs 70%; HR, 1.19; 95% CI, 0.61-2.33; *P* = .61) or chromophobe RCC (5-year RFS: 79% vs 77%; HR, 0.89; 95% CI, 0.37-2.13; *P* = .79). In the combined non–clear RCC cohort, grade 3 or higher adverse events occurred in 48% of patients who received everolimus and 9% of patients who received placebo.

**Conclusions and Relevance:**

In this clinical trial assessing the use of adjuvant everolimus, postoperative everolimus did not show evidence of improved RFS among patients with papillary or chromophobe RCC, and results from the study do not support adjuvant everolimus for this cohort. However, since the lower bounds of the 95% CIs were 0.61 and 0.89, respectively, potential treatment benefit in these subgroups cannot be ruled out.

**Trial Registration:**

ClinicalTrials.gov Identifier: NCT01120249

## Key Points

**Question:**

Does adjuvant everolimus improve recurrence-free and overall survival in patients with localized non–clear cell renal cell carcinoma (RCC)?

**Findings:**

This secondary analysis of a randomized clinical trial subgroup included 109 patients with papillary RCC and 99 patients with chromophobe RCC. In both groups, patients treated with adjuvant everolimus did not experience an improvement in recurrence-free survival compared with patients treated with placebo.

**Meaning:**

In this randomized clinical trial subset analysis, everolimus was not found to significantly improve either outcome when used in the adjuvant setting.

## Teaser

This secondary analysis of a randomized clinical trial assessed the effect of adjuvant everolimus after nephrectomy on recurrence-free and overall survival in patients with localized papillary or chromophobe renal cell carcinoma.

## Introduction

Non–clear cell renal cell carcinoma (RCC) accounts for approximately 25% of RCC and includes biologically and clinically distinct tumors, including papillary RCC, chromophobe RCC, microphthalmia transcription factor family translocation RCC, renal medullary carcinoma, fumarate hydratase-deficient RCC, collecting duct carcinoma, and unclassified RCC.^1^ While current practice guidelines recommend consideration of adjuvant pembrolizumab or sunitinib for the treatment of patients with clear-cell RCC after nephrectomy, no such recommendations exist for patients with non–clear cell RCC.^2^ This stems primarily from a lack of representation of these patients in clinical trials.^3,4,5,6^ The phase 3 randomized clinical trial (RCT), EVEREST, assessed the mammalian target of rapamycin (mTOR) pathway inhibitor, everolimus, in the adjuvant setting.^7^ In this study, we present a subgroup analysis from this RCT, in which our objective was to identify benefit (if any) from this drug in the papillary and chromophobe RCC subgroups.

## Methods

This RCT was a prespecified secondary analysis of the EVEREST RCT. The trial protocol was approved by all participating institutions’ institutional review boards and the National Cancer Institute (NCI) central institutional review board, and all participants provided written informed consent. The trial protocol and statistical analysis plan are provided in Supplement 1. This study is reported following the Consolidated Standards of Reporting Trials (CONSORT) reporting guideline.

### Trial Design and Participants

EVEREST was a phase 3, double-blind, placebo-controlled RCT.^7^ Between April 1, 2011, and September 15, 2016, a total of 1545 patients were enrolled across 398 sites in the US. Eligible patients included those with clear-cell and non–clear cell histologies (excluding those with collecting duct or medullary carcinomas). We conducted a subgroup analyses among 109 patients with papillary RCC and 99 patients with chromophobe RCC (eFigure in Supplement 2).

Eligible patients had to have a full surgical resection with negative margins (radical or partial nephrectomy) within 84 days prior to randomization and not in receipt of any other systemic therapy. Additionally, patients were required to either be categorized as intermediate-high risk for recurrence (tumor stage 1B with grade 3 or 4, tumor stage 2 any grade, or tumor stage 3A with grade 1 or 2 and no nodal metastases) or very-high risk for recurrence (tumor stage 3A with grade 3 or 4; tumor stage 3B, 3C, or 4 with any grade; or nodal metastases with any tumor stage or grade), as adapted from prior staging systems and clinical trials.^8,9^ Additional eligibility criteria are provided in the trial protocol (Supplement 1).

### Intervention

Eligible patients were randomized 1:1 to receive either everolimus or placebo using a dynamic balancing algorithm with stratification based on risk group (intermediate high vs very high), histology (clear cell vs non–clear cell RCC), and Zubrod performance status (scored as 0 vs 1). Randomization procedures were conducted by the SWOG Statistics and Data Management Centre, and patients were randomly assigned on study registration through the NCI OPEN web-based application. Participants, investigators, and those assessing outcomes were masked to group assignment. The study was double-blinded, with everolimus being administered (10 mg orally once daily) to the intervention group and matched placebo to the other group. Treatment was continued for 54 weeks or until disease recurrence, unacceptable adverse events, delay of more than 28 days, or patient refusal. Dose interruptions and reductions were allowed for toxic effects (trial protocol in Supplement 1).

### Outcomes

To monitor for recurrence, patients underwent clinical evaluation (history and physical examinations), radiologic assessment (scans of the chest, abdomen, and pelvis) every 18 weeks in the first year, every 6 months in the next 2 years, and then annually thereafter until cancer recurrence, death, or a maximum of 10 years after randomization. Information on self-reported race and ethnicity was collected as part of the demographic data of the study participants. Race and ethnicity were classified as Asian, Black, White, Native American, Pacific Islander, multirace, and unknown. The primary end point was recurrence-free survival (RFS), defined as the time from randomization to first documented recurrence (local or distant) or death due to any cause, whichever event occurred first. Postrecurrence, the patients’ treatment was unblinded to identify the next best treatments. Secondary end points included overall survival (OS). Survival was defined from the date of randomization to death from any cause. Data on adverse events for safety assessment were assessed using the Common Terminology Criteria for Adverse Events (CTCAE) Version 4.0.^10^

### Statistical Analysis

Statistical analysis for the primary analysis has been published elsewhere.^7^ Subgroup analyses based on the 3 stratification factors, risk group (intermediate-high vs very high risk), histology (clear cell vs non–clear cell), and performance status (0 vs 1), were prespecified. The final analysis was performed in March 2022. For testing the hazard ratio (HR) for treatment effect, a Cox regression model was used for both OS and RFS. Due to the small sample sizes for the 2 subgroups, no additional covariates were included in the model. For RFS, recurrence or death was the event of interest. Censoring for both end points was the last contact date. To evaluate whether the treatment effect differed for clear cell vs papillary or chromophobe, all patients were included in the Cox model, and an indicator for histology (clear cell vs non–clear cell) and an interaction term for treatment with histology were placed in the model and evaluated with a residual χ^2^ test. Kaplan-Meier curves were used to estimate OS and RFS distributions by treatment group. *P* values are reported as 2-sided, and significance was set at P ≤ .044. Analyses were conducted using SAS software version 9.4 (SAS Institute).

## Results

From April 1, 2011, to September 15, 2016, a total of 1545 patients were randomized, of whom 109 patients had papillary RCC (57 received everolimus and 52 received placebo; median [range] age, 60 [19-81] years; 82 [75%] male; 50 patients [46%] with very high–risk disease) and 99 patients had chromophobe RCC (53 received everolimus and 46 placebo; median [range] age 51 [18-71] years; 53 [54%] male; 34 patients [34%] with very high–risk disease). Patient characteristics, as shown in Table 1, were comparable between the groups, and representation was similar across the 3 histologic groups. The papillary RCC group included 1 Asian patient (1%), 14 Black patients (13%), 91 White patients (83%), and 3 patients with unknown race or ethnicity (3%). The chromophobe RCC group included 2 Asian patients (2%), 7 Black patients (7%), 1 Pacific Islander patient (1%), and 89 White patients (90%). Most patients had a Zubrod performance status of 0 (86 patients [79%] with papillary RCC and 84 patients [85%] with chromophobe RCC). Details about the entire cohort have been published previously.^7^ Of 755 patients assigned to the everolimus group, 355 (47%) did not start treatment or stopped earlier than the designated 54 weeks due to adverse events, refusal, or other reasons. In the placebo group of 744 eligible patients, 275 patients (37%) did not complete treatment (eFigure in Supplement 2). Additionally, most patients had undergone a radical nephrectomy as opposed to a partial nephrectomy in both subgroups (84 patients [77%] with papillary RCC and 87 patients [88%] with chromophobe RCC).

**Table 1.** Patient Characteristics

Characteristic	Patients, No. (%)		
	Clear cell (n = 1248)	Papillary (n = 109)	Chromophobe (n = 99)
Randomization group, No.			
Everolimus	626	57	53
Placebo	622	52	46
Age, median (range), y	59 (19-90)	60 (19-81)	51 (18-71)
Sex			
Male	883 (71)	82 (75)	53 (54)
Female	365 (29)	27 (25)	46 (46)
Zubrod performance status			
0	991 (79)	86 (79)	84 (85)
1	257 (21)	23 (21)	15 (15)
Race and ethnicity			
Asian	27 (2)	1 (1)	2 (2)
Black	37 (3)	14 (13)	7 (7)
Native American	11 (1))	0 (0))	0 (0))
Pacific Islander	0 (0)	0 (0)	1 (1)
White	1141 (91)	91 (83)	89 (90)
Multirace	4 (<1)	0 (0)	0 (0)
Unknown	28 (2)	3 (3)	0 (0)
Risk group			
Very high	699 (56)	50 (46)	34 (34)
Intermediate high	549 (44)	59 (54)	65 (66)
Nephrectomy			
Radical	1142 (92)	84 (77)	87 (88)
Partial	106 (8)	25 (23)	12 (12)
Pathologic LN status			
pN0	380 (30)	17 (16)	28 (28)
pNR (>but fully resected)	70 (6)	23 (21)	9 (9)
PNx (clinically N0)	798 (64)	69 (63)	62 (63)

After a median (IQR) follow-up of 76 (61-96) months, RFS was not found to be improved with everolimus compared with placebo in papillary RCC (5-year RFS: 62% vs 70%; HR, 1.19; 95% CI, 0.61-2.33, *P* = .61) or in chromophobe RCC (5-year RFS: 79% vs 77%; HR, 0.89; 95% CI, 0.37-2.13; *P* = .79) (Figure, A). At the time of analysis, 290 of 1499 total patients (19%) had died, including 26 patients (24%) in the papillary subgroup and 14 patients (14%) in the chromophobe subgroup. Median OS was not reached (papillary RCC HR for death, 1.47; 95% CI, 0.67-3.24; *P* = .34; chromophobe RCC HR for death, 0.93; 95% CI, 0.33-2.65; *P* = .89) (Figure, B). The estimated survival rate at 5 years was 76% in the everolimus group vs 82% in the placebo group in the papillary RCC subgroup and 89% in both groups of the chromophobe RCC subgroup.

**Figure.**
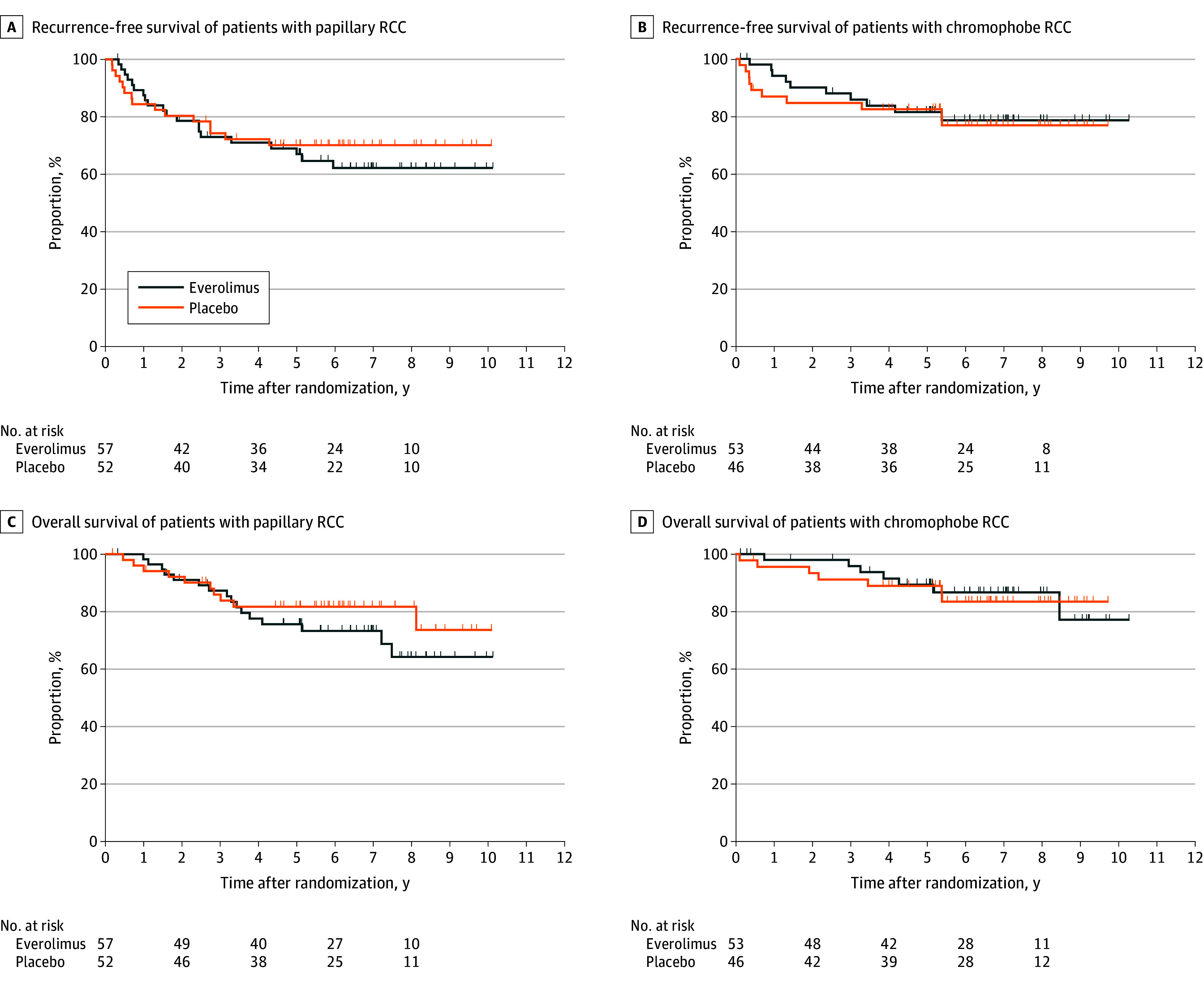
Kaplan-Meier Curves for Recurrence-Free Survival and Overall Survival in Patients with Papillary and Chromophobe Renal Cell Carcinoma (RCC)

Adverse events were assessed in the combined papillary and chromophobe cohort. Adverse events of any grade attributable to treatment occurred in 97of 101 patients (96%) treated with everolimus and in 66 of 81 patients (81%) treated with placebo. In the combined non–clear RCC cohort, grade 3 or higher adverse events occurred in 48% of patients who received everolimus and 9% of patients who received placebo. The most common grade 3 and above adverse events in the everolimus group were mucositis (14 patients [13%]), hypertriglyceridemia (15 patients [14%]), fatigue (6 patients [6%]), and hyperglycemia (5 patients [5%]) (Table 2). No treatment-related deaths were reported. Of eligible patients who were randomized to everolimus, 31 of 57 patients (54%) in the papillary RCC subgroup and 27 of 53 patients (51%) in the chromophobe RCC group discontinued treatment before the full 54 weeks due to adverse events, refusal, recurrence, or other unrelated reasons, compared with 15 of 52 patients (29%) with papillary RCC and 12 of 46 patients with chromophobe RCC (26%) in the placebo group (eTable 1 and eTable 2 in Supplement 2).

**Table 2.** AEs in the Combined Papillary (n = 108) and Chromophobe (n = 97) Safety Cohort^a^

Treatment-related AE	Participants by AE grade, No. (%)					
	Everolimus group (n = 110)			Placebo group (n = 95)		
	1-2	3	4	1-2	3	4
Anemia	41 (41)	1 (1)	0	7 (7)	0	0
Gastrointestinal disorders						
Abdominal pain	10 (10)	1 (1)	0	2 (2)	0	0
Mucositis oral	61 (56)	14 (13)	0	18 (19)	0	0
General disorders and administration site conditions						
Fatigue	50 (45)	6 (6)	0	33 (35)	1 (1)	0
Irritability	1 (1)	0	0	0	1 (1)	0
Infections and infestations						
Infections or infestations, other	0	0	0	0	1 (1)	0
Skin infection	4 (4)	1 (1)	0	0	0	0
Investigations						
ALT elevated	19 (17)	2 (2)	0	3 (3)	0	0
AST elevated	22 (20)	1 (1)	0	3 (1)	1 (1)	0
Cholesterol high	54 (49)	1 (1)	0	10 (11)	1 (1)	0
Metabolism and nutrition disorders						
Hyperglycemia	29 (26)	5 (5)	0	18 (19)	0	1 (1)
Hyperkalemia	2 (2)	1 (1)	0	1 (1)	1 (1)	0
Hypertriglyceridemia	42 (38)	10 (9)	5 (5)	24 (25)	0	0
Hypophosphatemia	0	2 (2)	0	0	1 (1)	0
Back pain	4 (4)	1 (1)	0	2 (2)	0	0
Kidney and urinary disorders						
Proteinuria	1 (1)	1 (1)	0	0	0	0
Kidney and urinary disorders, others	1 (1)	1 (1)	0	0	0	0
Respiratory, thoracic, and mediastinal disorders						
Hypoxia	0	1 (1)	0	0	0	0
Pneumonitis	9 (8)	3 (3)	0	0	0	0
Skin and subcutaneous tissue disorders						
Rash (acneiform)	38 (35)	2 (2)	0	7 (7)	0	0
Rash (maculopapular)	29 (26)	1 (1)	0	9 (10)	0	0
Skin and subcutaneous tissue disorders, others	10 (9)	0	0	4 (4)	1 (1)	0
Hypertension	18 (16)	6 (6)	0	11 (12)	4 (4)	0
Maximum grade: all hematologic AEs	61 (55)	1 (1)	0	16 (17)	0	0
Maximum grade: all nonhematologic AEs	56 (51)	44 (40)	5 (5)	64 (65)	10 (11)	1 (1)
Maximum grade: any AEs	49 (49)	43 (43)	5 (5)	63 (66)	11 (12)	1 (1)

Abbreviations: AE, adverse event; ALT, alanine transaminase; AST, Aspartate transaminase.

^a^ AEs with at least 1 grade 3 event reported. Attributions include possible, probable, and definite.

## Discussion

In this secondary analysis of an RCT, we report one of the largest non–clear cell RCC cohorts treated with an mTOR inhibitor, everolimus, after partial or radical nephrectomy. The role of everolimus is of particular interest in patients with chromophobe RCC. Mutations in *mTOR*, *NRAS*, *TSC1*, or *TSC2* have been reported in approximately 23% of patients with chromophobe RCC,^10^ suggesting possible therapeutic benefit from an mTOR inhibitor in this subset. While most prior studies in metastatic RCC did not include patients with non–clear cell RCC, there are some that have included these cohorts and have shown a benefit with either single-agent everolimus or when combined with other agents.^11,12,13,14^ In a subgroup of 16 patients with chromophobe RCC included in the phase 3 ASPEN study, there was no statistically significant benefit from everolimus (median PFS: 11.4 vs 5.5 months; HR. 0.7; 95% CI, 0.3-1.7).^16^ In another phase 2 single-group study that included 9 patients with chromophobe RCC, a disease control rate of 78% was reported with a combination of everolimus and lenvatinib.^13^ Similarly, everolimus has been shown to be clinically efficacious in patients with papillary RCC as well, based on results from a front-line phase 2 RCT,^15^ thus warranting assessment of benefit in the adjuvant setting as well. Although the administration of adjuvant everolimus in this study did not improve RFS in either papillary RCC or chromophobe RCC subgroups, the lower bound of the 95% CI was 0.61 for papillary RCC, and 0.89 for chromophobe RCC, so a potential treatment benefit in these subgroups cannot be ruled out.

Although the analysis was underpowered to detect a significant difference and was not designed as the primary analysis, our study benefits from a relatively large sample size for non–clear cell histologies. While the use of adjuvant therapies has received US Food and Drug Administration approval for patients with clear-cell RCC, the non–clear cell cohorts remain an area of unmet need.

### Limitations

This study has limitations. The subgroup analyses were not powered to test a difference, and there is a concern for false positives with multiple testing. The lack of a central pathology review to confirm non–clear cell histologies is also a shortcoming for this study. Nevertheless, EVEREST enrolled one of the largest cohorts of patients with papillary or chromophobe RCC in the adjuvant setting. Future biomarker analysis should enrich these data and help us elucidate the differences between these cohorts in greater depth.

## Conclusions

This secondary analysis of the EVEREST RCT found that patients with papillary or chromophobe RCC did not benefit from treatment with everolimus in the adjuvant setting. Our study highlights an area of unmet need in the kidney cancer field. It thus serves to provide a foundational background for future RCTs to address specific subgroups of RCC for risk mitigation strategies in the adjuvant setting.

## Notes

Supplement 1.
Trial Protocol and Statistical Analysis Plan

Supplement 2. **eTable 1.** Reasons to Go Off-Study in Each Cohort Analyzed **eFigure.** Patient Enrollment Flowchart **eTable 2.** Duration of Treatment in Each Cohort for Those Who Stopped Treatment Early

Supplement 3.
Data Sharing Statement
