# Supplementary material for: Adjuvant Everolimus in Non–Clear Cell Renal Cell Carcinoma: A Secondary Analysis of a Randomized Clinical Trial
Source: JAMA Netw Open. 2024 Aug 6;7(8):e2425288. doi: 10.1001/jamanetworkopen.2024.25288 (PMC11304111; doi:10.1001/jamanetworkopen.2024.25288)
Supplement: Supplement 1. — Trial Protocol and Statistical Analysis Plan [file jamanetwopen-e2425288-s001.pdf]

SWOG CANCER RESEARCH NETWORK

Version Date: December 11, 2020

TO: ALL NATIONAL CLINICAL TRIALS NETWORK (NCTN) MEMBERS

FROM: Veronica Garcia, M.S., Protocol Coordinator II (E-mail: vgarcia@swog.org)

RE: **S0931**, "EVEREST: EVErolimus for Renal Cancer Ensuing Surgical Therapy, A Phase III Study." Study Chairs: Drs. C.W. Ryan, E.I. Heath, P.N. Lara, G.S. Palapattu, and P.C. Mack.

REVISION #10

Study Chair: Christopher W. Ryan, M.D.
Phone number: 503/494-8487
E-mail: ryanc@ohsu.edu

IRB Review Requirements
(√) Expedited review

REVISION #10

1. The version date of the protocol and consent form were updated. No other changes were made to the consent form.

2. Section 11.2: The statistical plan was updated by the addition of the section titled “December 2020 Revision”. The Timing of Scheduled Analyses table was updated.

This memorandum serves to notify the NCI and the SWOG Statistics and Data Management Center.

cc: PROTOCOL & INFORMATION OFFICE

SWOG CANCER RESEARCH NETWORK

November 1, 2019

TO: ALL NATIONAL CLINICAL TRIALS NETWORK (NCTN) MEMBERS

FROM: Veronica Garcia, M.S., Protocol Coordinator II (E-mail: vgarcia@swog.org)

RE: **S0931**, "EVEREST: EVErolimus for Renal Cancer Ensuing Surgical Therapy, A Phase III Study." Study Chairs: Drs. C.W. Ryan, E.I. Heath, P.N. Lara, G.S. Palapattu, and P.C. Mack.

MEMORANDUM

Study Chair: Christopher W. Ryan, M.D.
Phone number: 503/494-8487
E-mail: ryanc@ohsu.edu

IRB Review Requirements
(√) No review required

MEMORANDUM

The purpose of this memorandum is to notify sites of the following changes to the **S0931** Master Forms Set:

Specific Follow Up Form (#36343)

- A note was added to the "Disease Follow-Up Status" section indicating when disease should be assessed.

This memorandum serves to notify the NCI and the SWOG Statistics and Data Management Center.

cc: PROTOCOL & INFORMATION OFFICE

October 15, 2019

TO: ALL NATIONAL CLINICAL TRIALS NETWORK (NCTN) MEMBERS

FROM: SWOG Operations Office

RE: IND Safety Reports for Everolimus

MEMORANDUM

IRB Review Requirements
(√) Expedited review allowed

MEMORANDUM

T The following new safety report(s) have been posted regarding adverse events that occurred in association with the drug everolimus. Please access the safety report(s) via the SWOG or CTSU website. On the SWOG website, view the study's abstract page or the safety report link (https://www.swog.org/member-resources/safety-reports). On the CTSU website, view the drug safety notifications on the study's abstract page (www.ctsu.org).

The safety report(s) pertain to the following study(ies):	Report(s):
**S0931** Genitourinary<br>**S1207** Breast<br>**S1222** Breast	Aug. 19, 2019 Mfr Rpt #PHHO2019BE006135

Protocol amendments are not necessary at this time, but your consent form may be revised to include the information provided in the report(s) if it is deemed necessary by your institution. Please append this notice and the report(s) to your copy of the protocol and forward copies to your Institutional Review Board (IRB) as required by your local policies and procedures. Should any further information regarding these adverse events be made available, it will be forwarded to you.

This memorandum serves to notify the NCI and the SWOG Statistics and Data Management Center.

cc: PROTOCOL & INFORMATION OFFICE

SWOG
Leading cancer research. Together.

GROUP CHAIR'S OFFICE
Charles D. Blanke, MD
CHAIR
3181 SW Sam Jackson Pk Rd
MC: L586
Portland, OR 97239
503-494-5586
503-346-8038 FAX

OPERATIONS OFFICE
4201 Medical Dr
Suite 250
San Antonio, TX 78229
210-614-8808
210-614-0006 FAX

STATISTICAL CENTER
1730 Minor Ave
Suite 1900
Seattle, WA 98101
206-652-2267
206-342-1616 FAX

1100 Fairview Ave North
M3-C102
PO Box 19024
Seattle, WA 98109
206-667-4623
206-667-4408 FAX

swog.org

January 15, 2018

TO: ALL NATIONAL CLINICAL TRIALS NETWORK (NCTN) MEMBERS

FROM: SWOG Operations Office

RE: IND Safety Reports for Everolimus

MEMORANDUM

IRB Review Requirements
(√) Expedited review allowed

MEMORANDUM

The following new safety reports have been posted regarding adverse events that occurred in association with the drug everolimus. Please access this safety report via the safety report link in the member section of the SWOG website (https://swog.org/member-resources/safety-reports).

These safety reports pertain to the following studies:	Reports:
**S0931** Genitourinary<br>**S1207** Breast<br>**S1222** Breast	Dec. 08, 2017 AE-2542577

Protocol amendments are not necessary at this time, but your consent form may be revised to include the information provided in this report if it is deemed necessary by your institution. Please append this notice and this report to your copy of the protocol and forward copies to your Institutional Review Board (IRB) as required by your local policies and procedures. Should any further information regarding these adverse events be made available, it will be forwarded to you.

This memorandum serves to notify the NCI and the SWOG Statistical Center.

cc: PROTOCOL & INFORMATION OFFICE

SWOG Authorized Document

SWOG
Leading cancer research. **Together.**

GROUP CHAIR'S OFFICE

Charles D. Blanke, MD
CHAIR

3181 SW Sam Jackson Pk Rd
MC: L586
Portland, OR 97239

503-494-5586
503-346-8038 FAX

OPERATIONS OFFICE

4201 Medical Dr
Suite 250
San Antonio, TX 78229

210-614-8808
210-614-0006 FAX

STATISTICAL CENTER

1730 Minor Ave
Suite 1900
Seattle, WA 98101

206-652-2267
206-34[illegible] FAX

1100 Fair[illegible]w [illegible]ve[illegible]th
M3-C102
PO Box 19024
Seattle, WA 98109

206-667-4623
206-667-4408 FAX

swog.org

CLOSED EFFECTIVE 09/15/2016

Distribution Date: November 1, 2017
E-mail Date: October 17, 2017
Version Date: October 3, 2017

TO: ALL NATIONAL CLINICAL TRIALS NETWORK (NCTN) MEMBERS – U.S. INSTITUTIONS ONLY

FROM: Nicki Trevino, Protocol Coordinator (E-mail: ntrevino@swog.org)

RE: **S0931**, "EVEREST: EVErolimus for Renal Cancer Ensuing Surgical Therapy, A Phase III Study. Study Chairs: Drs. C.W. Ryan, E.I. Heath, P.N. Lara, G.S. Palapattu, and P.C. Mack.

REVISION #9

Study Chair: Christopher W. Ryan, M.D.
Phone number: 503/494-8487
E-mail: ryanc@ohsu.edu

IRB Review Requirements
(√) Expedited review allowed

Protocol changes
(√) Informed Consent changes
(√) Patient notification required: See below
(√) Other: Updated everolimus risk information

Sites using the CIRB as their IRB of record: The protocol and/or informed consent form changes have been approved by the CIRB and must be activated within 30 days of the CIRB posting of this notice. If local approval is not granted within 30 days, accrual must be suspended until approval is obtained.

Sites not using the NCI CIRB: Per CTMB Guidelines, the protocol updates and/or informed consent changes must be approved by local IRBs within 90 days of distribution of this notice. The changes in this revision are effective upon approval by the local IRB; however, any changes to eligibility become effective 6 weeks after distribution of this notice. If local approval is not granted within 6 weeks, accrual must be suspended until approval is obtained.

REVISION #9

The above-referenced study has been updated as follows:

This revision has been prepared in response to the Request for Rapid Amendment (RRA) for Everolimus (NSC-733504) (IND-115643) from Dr. Austin Doyle (doylela@mail.nih.gov), Dr. James Zwiebel (zwiebelj@ctep.nci.nih.gov), and Dr. Meg

SWOG Authorized Document

Mooney (mooneym@ctep.nci.nih.gov) on September 19, 2017. The associated action letter is attached.

1. The Version Dates of the protocol and Model Consent Form have been updated.

2. Table of Contents: The page numbers have been updated.

3. Section 3.1c: the CAEPR for Everolimus, Version 2.3, June 30, 2016 has been replaced with Version 2.4, July 10, 2017. The section has been updated as follows:

   - Increase in Risk Attribution:
     - Changed to Rare But Serious from Also Reported on Everolimus Trials But With Insufficient Evidence for Attribution: Skin and subcutaneous tissue disorders - Other (angioedema)
   - Provided Further Clarification:
     - The order of the footnotes has changed.

Model Consent Form Changes

Patients currently receiving everolimus **must** be informed of the bolded changes below. The manner by which this notification takes place is at the discretion of the local institution. At a minimum, the patient must be notified by the next visit and this notification must be documented in the patient chart. A Consent Addendum outlining the Model Consent Form changes is enclosed for sites that choose to utilize it for patient notification; however, use of the Consent Addendum is not required. Patients need not be informed of the non-bolded changes unless required by the local IRB.

1. The following changes have been made to the risk tables for Everolimus in the Model Consent Form:

   - Increase in Risk Attribution:
     - Changed to Rare from Also Reported on Everolimus Trials But With Insufficient Evidence for Attribution (i.e., added to the Risk Profile): **Allergic reaction which may cause rash, low blood pressure, wheezing, shortness of breath, swelling of the face or throat**.

   - Provided Further Clarification:
     - Swelling of the lungs which may cause shortness of breath (under Occasional) is now reported as Damage to the lungs which may cause shortness of breath (under Occasional).
     - Sores in mouth which may cause difficulty swallowing (under Common) is now reported as Sores in the mouth which may cause difficulty swallowing (under Common).

cc: PROTOCOL & INFORMATION OFFICE
Meredith Lavin - Novartis
Jason Koury, Pharm.D. - VACSP
Sharon Jenkins – VACSP

CLOSED EFFECTIVE 09/15/2016

SWOG Authorized Document

Informed Consent Addendum Model for S0931

Study Title for Study Participants: Everolimus in Treating Patients With Kidney Cancer Who Have Undergone Surgery

Official Study Title for Internet Search on

http://www.ClinicalTrials.gov: ***S0931*** *EVEREST: EVErolimus for Renal Cancer Ensuing Surgical Therapy, a Phase III Study*

*NOTES FOR LOCAL INSTITUTION INFORMED CONSENT AUTHORS:

The purpose of a consent addendum is to:

- Inform patients of any significant new findings developed during the course of participation that may have a bearing on their willingness to continue in the study.
- Inform patients of specific changes (rather than having them sign a modified consent). The addendum will facilitate discussion since the changes/new findings are the focus of the document.

This consent addendum has been prepared for patients currently registered to S0931 and/or receiving the study drug everolimus or matching placebo.

The following information should be read as an addition to the original Consent form that you read and signed at the beginning of the study. Unless specifically stated otherwise in the following paragraphs, all information contained in that original Consent Form is still true and remains in effect. Your participation continues to be voluntary. You may refuse to participate, or may withdraw your consent to participate at any time, and for any reason, without jeopardizing your future care at this institution or your relationship with your study doctor.

New or additional information

The way that risk information is explained has recently changed. Please note that while the way the information is explained has changed, the risks related to everolimus/placebo have not changed, except as specifically noted below. The following changes have been made to the risk tables for everolimus/placebo:

SWOG Authorized Document

The following item has been added to the "Rare, and Serious" side effect table for everolimus/placebo:

- **Allergic reaction which may cause rash, low blood pressure, wheezing, shortness of breath, swelling of the face or throat.**

In the Common, Some may be Serious section:

- **"Sores in mouth which may cause difficulty swallowing" is now reported as, "Sores in the mouth which may cause difficulty swallowing."**

In the Occasional, Some may be Serious section:

- **"Swelling of the lungs which may cause shortness of breath" is now reported as, "Damage to the lungs which may cause shortness of breath".**
- **The bullet for "nausea, vomiting" was removed, as these risks are already listed in the bullet point, "Constipation, nausea, vomiting."**

The tables provided below are the most current risk tables for everolimus. These tables show the most common and the most serious side effects that researchers know about related to everolimus/placebo. There might be other side effects that researchers do not yet know about. If important new side effects are found, the study doctor will discuss these with you.

COMMON, SOME MAY BE SERIOUS<br>In 100 people receiving Everolimus/Placebo, more than 20 and up to 100 may have:
• Anemia which may require blood transfusion<br>• Diarrhea<br>• Sores in the mouth which may cause difficulty swallowing<br>• Tiredness<br>• Rash

SWOG Authorized Document

OCCASIONAL, SOME MAY BE SERIOUS
In 100 people receiving everolimus, from 4 to 20 may have:

- Pain
- Constipation, nausea, vomiting
- Swelling of arms, legs
- Fever
- Infection, especially when white blood cell count is low
- Bruising, bleeding
- Weight loss, loss of appetite
- Changes in taste
- Headache
- Cough, shortness of breath
- Nose bleed
- Damage to the lungs which may cause shortness of breath
- Dry skin
- Itching

RARE, AND SERIOUS
In 100 people receiving everolimus, 3 or fewer may have:

- Non-healing surgical site
- Kidney damage which may require dialysis
- Allergic reaction which may cause rash, low blood pressure, wheezing, shortness of breath, swelling of the face or throat

My Signature Agreeing to Take Part in the Study

I have read this consent form or had it read to me. I have discussed it with the study doctor and my questions have been answered. I will be given a signed copy of this form. I agree to take part in the main study *and any additional studies where I circled 'yes'.*

Participant's signature____

Date of signature________________________________

SWOG Authorized Document

DEPARTMENT OF HEALTH & HUMAN SERVICES

Public Health Service

National Institutes of Health
National Cancer Institute
Bethesda, Maryland 20892

Action Letter

DATE: **October 10, 2017**

FROM: L. Austin Doyle, MD, Medical Officer, IDB, CTEP, DCTD, NCI
James Zwiebel, MD, Branch Chief, IDB, CTEP, DCTD, NCI
Meg Mooney, MD, Branch Chief, CIB, CTEP, DCTD, NCI

SUBJECT: **CONFIDENTIAL COMMUNICATION** – Action Letter for Everolimus (RAD-001, NSC 733504)

TO: Investigators for CTEP-supported Studies Involving Everolimus (RAD-001, NSC 733504)

The purpose of this Action Letter is to alert patients and investigators in studies conducted by the Cancer Therapy Evaluation Program (CTEP), National Cancer Institute (NCI), of new and/or modified risk information associated with everolimus, and to request all trials with everolimus be amended to reflect these changes. You are receiving this letter because you are conducting a CTEP-sponsored trial that includes everolimus. See the accompanying list of CTEP trials with everolimus.

In response to the new/modified risk information CTEP is requiring that all trials with everolimus be amended to reflect this new information. Detailed description of the new/modified risk(s) as well as detailed instructions regarding amendment requirements are described in this Action Letter. **Amendments are due to the Protocol and Information Office (PIO) at PIO@CTEP.NCI.NIH.GOV by 5 PM ET on October 24, 2017,** or as required based on protocol status (see the *General Actions Required Based on Protocol Status* section). The cover letter for the submitted amendment should state that it is being submitted in response to an Action Letter from Dr. L. Austin Doyle (doylela@mail.nih.gov). Failure to respond in a timely fashion may result in suspension of the Principal Investigator or permanent study closure.

After review of all the available data, CTEP believes that the new and/or modified risk information does **NOT** significantly alter the risk-benefit profile for patients in the study since everolimus is already known to cause serious adverse events and this new risk information does not change the overall weight given to risks versus benefits for patients in the study. CTEP considers all the proposed protocol and informed consent changes for studies affected by this Action Letter to be minor. Therefore, under the provisions of Department of Health and Human Services regulations for the protection of human subjects at 45 CFR 46.110, a protocol amendment that includes this new information can undergo expedited review if the Institutional Review Board (IRB) Chair (or other experienced IRB member designated to conduct expedited review by the Chair) concurs that the changes are minor. Additional information from the Office of Human Research Protections (OHRP) regarding this process is available at: http://www.hhs.gov/ohrp/policy/Correspondence/nci200870929.html.

The following section, *Specific Instruction*, includes background information on the risk(s), any risk mitigation strategies, and amendment requirements. The revised Comprehensive Adverse Events and Potential Risks (CAEPR) list (Attachment 1) and Informed Consent Document (ICD) risk information (Attachment 2) are also attached. Action Letter general instructions as well as instructions regarding amendment preparation (if a CTEP-approved amendment does not already accompany this Action Letter) are included in Attachment 3. **You MUST follow the instructions outlined in Attachment 3.**

Action Letter

SPECIFIC INSTRUCTION

Background

As part of Good Clinical Practice, CTEP reviews each CAEPR list on an annual basis. The review includes literature search, CTEP-AERS submission review, and comparison to the latest agent Investigator's Brochure. After review of all the available data, CTEP has identified new and/or modified risk information associated with everolimus.

Detailed Description of Required Protocol Changes for the Amendment/ Sample Change Memo Template

1) New Protocol Amendment/Version Date Included on the Title/Cover Page per Operations Office Policy:

   Protocol Cover Page: Page Number(s): ______
   Version Date: __________

2) Revision of the Protocol CAEPR:

   Protocol Section(s) for Insertion of Revised CAEPR (Version 2.4, July 10, 2017): ___
   Page Number(s): ____

   - Increase in Risk Attribution:
     - Changed to Rare But Serious from Also Reported on Everolimus Trials But With Insufficient Evidence for Attribution: Skin and subcutaneous tissue disorders - Other (angioedema)

   - Provided Further Clarification:
     - The order of the footnotes has changed.

   PLEASE NOTE: The specific detailed changes listed here compare the new revised CAEPR Version 2.4, and associated risk information for the ICD, to the most recent CAEPR Version 2.3. If your trial contains an older CAEPR version (i.e., does **NOT** currently contain CAEPR Version 2.3), you **MUST** include a description of any additional changes resulting from migration from the older CAEPR version.

3) Revision of the ICD as Specified Below:

   The terminology for CTEP's suggested lay terms may change periodically. The condensed risk profile represents CAEPR risks in lay terms in a "patient-friendly" condensed form. It is provided as a guide and its use is completely optional; however, all risks listed in the current CAEPR must be reflected in the ICD. Expanding or condensing similar terms is acceptable. If you have chosen to reformat and/or reword the condensed risk profile in any way, please state, "The condensed risk profile has been modified" in the cover memo.

   - Increase in Risk Attribution:
     - Changed to Rare from Also Reported on Everolimus Trials But With Insufficient Evidence for Attribution (i.e., added to the Risk Profile): Allergic reaction which may cause rash, low blood pressure, wheezing, shortness of breath, swelling of the face or throat

   - Provided Further Clarification:

- Swelling of the lungs which may cause shortness of breath (under Occasional) is now reported as Damage to the lungs which may cause shortness of breath (under Occasional).
- Sores in mouth which may cause difficulty swallowing (under Common) is now reported as Sores in the mouth which may cause difficulty swallowing (under Common).

PLEASE NOTE: The potential risks listed in the CAEPR whose relationship to everolimus is still undetermined are not required by CTEP to be described in the ICD; however, they may be communicated to patients according to local IRB requirements.

Action Letter

Attachment 1: Revised Everolimus (RAD-001) CAEPR – Version 2.4, July 10, 2017

Comprehensive Adverse Events and Potential Risks list (CAEPR) for Everolimus (RAD-001, NSC 733504)

The Comprehensive Adverse Events and Potential Risks list (CAEPR) provides a single list of reported and/or potential adverse events (AE) associated with an agent using a uniform presentation of events by body system. In addition to the comprehensive list, a subset, the Specific Protocol Exceptions to Expedited Reporting (SPEER), appears in a separate column and is identified with bold and italicized text. This subset of AEs (SPEER) is a list of events that are protocol specific exceptions to expedited reporting to NCI (except as noted below). Refer to the 'CTEP, NCI Guidelines: Adverse Event Reporting Requirements' http://ctep.cancer.gov/protocolDevelopment/electronic_applications/docs/aeguidelines.pdf for further clarification. *Frequency is provided based on 3033 patients.* Below is the CAEPR for Everolimus (RAD-001).

> **NOTE**: Report AEs on the SPEER **ONLY IF** they exceed the grade noted in parentheses next to the AE in the SPEER. If this CAEPR is part of a combination protocol using multiple investigational agents and has an AE listed on different SPEERs, use the lower of the grades to determine if expedited reporting is required.

Version 2.4, July 10, 2017[1]

Adverse Events with Possible Relationship to Everolimus (RAD-001) (CTCAE 4.0 Term) [n= 3033]			Specific Protocol Exceptions to Expedited Reporting (SPEER)
**Likely (>20%)**	**Less Likely (<=20%)**	**Rare but Serious (<3%)**	
BLOOD AND LYMPHATIC SYSTEM DISORDERS			
Anemia			***Anemia (Gr 2)***
GASTROINTESTINAL DISORDERS			
	Abdominal pain		
	Constipation		
Diarrhea[2]			***Diarrhea[2] (Gr 2)***
Mucositis oral[3]			***Mucositis oral[3] (Gr 2)***
	Nausea		***Nausea (Gr 2)***
	Vomiting		***Vomiting (Gr 2)***
GENERAL DISORDERS AND ADMINISTRATION SITE CONDITIONS			
	Edema limbs		***Edema limbs (Gr 2)***
Fatigue			***Fatigue (Gr 2)***
	Fever		***Fever (Gr 2)***
INFECTIONS AND INFESTATIONS			
	Infection[4]		***Infection[4] (Gr 2)***
INJURY, POISONING AND PROCEDURAL COMPLICATIONS			
		Wound complication[5]	
INVESTIGATIONS			
	Alanine aminotransferase increased		***Alanine aminotransferase increased (Gr 2)***
	Alkaline phosphatase increased		***Alkaline phosphatase increased (Gr 2)***
	Aspartate aminotransferase increased		***Aspartate aminotransferase increased (Gr 2)***
	Cholesterol high		***Cholesterol high (Gr 2)***
	Creatinine increased		***Creatinine increased (Gr 2)***
	Lymphocyte count decreased		***Lymphocyte count decreased (Gr 2)***
	Neutrophil count decreased		***Neutrophil count decreased (Gr 2)***
	Platelet count decreased		***Platelet count decreased (Gr 2)***

Adverse Events with Possible Relationship to Everolimus (RAD-001) (CTCAE 4.0 Term) [n= 3033]			Specific Protocol Exceptions to Expedited Reporting (SPEER)
**Likely (>20%)**	**Less Likely (<=20%)**	**Rare but Serious (<3%)**	
	Weight loss		
	White blood cell decreased		***White blood cell decreased (Gr 2)***
METABOLISM AND NUTRITION DISORDERS			
	Anorexia		***Anorexia (Gr 2)***
	Hyperglycemia[6]		***Hyperglycemia[6] (Gr 2)***
	Hypertriglyceridemia		***Hypertriglyceridemia (Gr 2)***
	Hypophosphatemia		***Hypophosphatemia (Gr 2)***
MUSCULOSKELETAL AND CONNECTIVE TISSUE DISORDERS			
	Arthralgia		
	Back pain		
	Pain in extremity		
NERVOUS SYSTEM DISORDERS			
	Dysgeusia		
	Headache		***Headache (Gr 2)***
RENAL AND URINARY DISORDERS			
		Acute kidney injury	
RESPIRATORY, THORACIC AND MEDIASTINAL DISORDERS			
	Cough		***Cough (Gr 2)***
	Dyspnea		***Dyspnea (Gr 2)***
	Epistaxis		***Epistaxis (Gr 2)***
	Pneumonitis[7]		
SKIN AND SUBCUTANEOUS TISSUE DISORDERS			
	Dry skin		
	Pruritus		
Rash maculo-papular			***Rash maculo-papular (Gr 2)***
		Skin and subcutaneous tissue disorders - Other (angioedema)[8]	

[1]This table will be updated as the toxicity profile of the agent is revised. Updates will be distributed to all Principal Investigators at the time of revision. The current version can be obtained by contacting PIO@CTEP.NCI.NIH.GOV. Your name, the name of the investigator, the protocol and the agent should be included in the e-mail.

[2]Includes diarrhea, enteritis, enterocolitis, colitis, defecation urgency, and steatorrhea.

[3]Includes stomatitis, aphthous stomatitis, gingival pain/swelling/ulceration, glossitis, glossodynia, lip ulceration, mouth ulceration, tongue ulceration, and mucosal inflammation.

[4]Infection includes all 75 infection sites under the INFECTIONS AND INFESTATIONS SOC.

[5]Everolimus delays wound healing and increases the occurrence of wound-related complications like wound dehiscence, wound infection, incisional hernia, lymphocele, and seroma.

[6]Hyperglycemia may result in either exacerbation of or development of new onset diabetes mellitus.

[7]Includes pneumonitis, interstitial lung disease, lung infiltration, pulmonary alveolar hemorrhage, pulmonary toxicity, alveolitis, pulmonary fibrosis, and restrictive pulmonary disease.

[8]Patients taking concomitant ACE inhibitor therapy may be at increased risk for angioedema.

[9]Includes agitation, anxiety, panic attack, aggression, abnormal behavior, and obsessive compulsive disorder.

Adverse events reported on Everolimus (RAD-001) trials, but for which there is insufficient evidence to suggest that there was a reasonable possibility that Everolimus (RAD-001) caused the adverse event:

BLOOD AND LYMPHATIC SYSTEM DISORDERS - Blood and lymphatic system disorders - Other (thrombotic microangiopathy)
CARDIAC DISORDERS - Atrial fibrillation; Cardiac disorders - Other (myocardial abnormality); Chest pain - cardiac; Heart failure; Left ventricular systolic dysfunction; Myocardial infarction; Sinus bradycardia; Sinus tachycardia; Supraventricular tachycardia
ENDOCRINE DISORDERS - Endocrine disorders - Other (increased blood follicle stimulating hormone [FSH] levels); Endocrine disorders - Other (increased blood luteinizing hormone [LH] levels); Endocrine disorders - Other (low testosterone); Hypothyroidism
EYE DISORDERS - Blurred vision; Conjunctivitis; Keratitis
GASTROINTESTINAL DISORDERS - Ascites; Colitis; Dry mouth; Dyspepsia; Dysphagia; Flatulence; Gastroesophageal reflux disease; Gastrointestinal disorders - Other (Dieulafoy's lesion); Hemorrhoids; Intra-abdominal hemorrhage; Oral pain; Pancreatitis; Periodontal disease; Toothache
GENERAL DISORDERS AND ADMINISTRATION SITE CONDITIONS - Chills; Edema face; Edema trunk; Flu like symptoms; Irritability; Non-cardiac chest pain; Pain
HEPATOBILIARY DISORDERS - Hepatic failure; Hepatobiliary disorders - Other (hepatomegaly)
IMMUNE SYSTEM DISORDERS - Allergic reaction
INVESTIGATIONS - Activated partial thromboplastin time prolonged; Blood bilirubin increased; CPK increased; GGT increased; INR increased; Investigations - Other (bicarbonate decreased); Investigations - Other (increased lactate dehydrogenase); Investigations - Other (low density lipoprotein raised); Investigations - Other (thrombocythemia)
METABOLISM AND NUTRITION DISORDERS - Dehydration; Glucose intolerance; Hypercalcemia; Hyperkalemia; Hypoalbuminemia; Hypocalcemia; Hypokalemia; Hypomagnesemia; Hyponatremia; Metabolism and nutrition disorders - Other (high ammonia); Metabolism and nutrition disorders - Other (hyperlipidemia)
MUSCULOSKELETAL AND CONNECTIVE TISSUE DISORDERS - Bone pain; Chest wall pain; Generalized muscle weakness; Muscle weakness lower limb; Musculoskeletal and connective tissue disorder - Other (muscle spasms); Myalgia
NEOPLASMS BENIGN, MALIGNANT AND UNSPECIFIED (INCL CYSTS AND POLYPS) - Neoplasms benign, malignant and unspecified (incl cysts and polyps) - Other (ovarian cysts)
NERVOUS SYSTEM DISORDERS - Dizziness; Encephalopathy; Hydrocephalus; Lethargy; Paresthesia
PSYCHIATRIC DISORDERS - Agitation; Anxiety[9]; Delirium; Depression; Insomnia; Mania
RENAL AND URINARY DISORDERS - Hematuria; Proteinuria; Urinary frequency
REPRODUCTIVE SYSTEM AND BREAST DISORDERS - Dysmenorrhea; Genital edema; Irregular menstruation; Menorrhagia; Vaginal hemorrhage
RESPIRATORY, THORACIC AND MEDIASTINAL DISORDERS - Bronchopulmonary hemorrhage; Pharyngolaryngeal pain; Pleural effusion; Respiratory failure; Respiratory, thoracic and mediastinal disorders - Other (rales); Respiratory, thoracic and mediastinal disorders - Other (rhinorrhea); Sore throat; Voice alteration
SKIN AND SUBCUTANEOUS TISSUE DISORDERS - Nail loss; Palmar-plantar erythrodysesthesia syndrome; Rash acneiform; Skin and subcutaneous tissue disorders - Other (nail disorder); Skin and subcutaneous tissue disorders - Other (skin lesion); Skin ulceration
VASCULAR DISORDERS - Flushing; Hypertension; Lymphedema; Phlebitis; Thromboembolic event; Vascular disorders - Other (acute bowel ischemia); Vascular disorders - Other (hemorrhage)

Note: Everolimus (RAD-001) in combination with other agents could cause an exacerbation of any adverse event currently known to be caused by the other agent, or the combination may result in events never previously associated with either agent.

Action Letter

Attachment 2: Revised ICD Section(s) for Everolimus (RAD-001)

Please note that the terminology for CTEP's suggested lay terms may change periodically. The condensed risk profile represents CAEPR risks in lay terms in a "patient-friendly" condensed format. It is provided as a guide and its use is completely optional; however, all risks listed in the current CAEPR must be reflected in the ICD. Expanding or condensing similar terms is acceptable. If you have chosen to reformat and/or reword the condensed risk profile in any way, please state, "The condensed risk profile has been modified" in the cover memo. Please insert this condensed risk profile as the Table of Possible Side Effects for Everolimus in your ICD.

Risk Profile for Everolimus (RAD-001) (CAEPR Version 2.4, July 10, 2017)

NOTE: The risk list section in the new NCI Consent Form Template (latest version: May 2013) will include the wording below:

"If you choose to take part in this study, there is a risk that:

- You may lose time at work or home and spend more time in the hospital or doctor's office than usual
- You may be asked sensitive or private questions which you normally do not discuss

The everolimus used in this study may affect how different parts of your body work such as your liver, kidneys, heart, and blood. The study doctor will be testing your blood and will let you know if changes occur that may affect your health.

There is also a risk that you could have side effects from the study drug(s)/study approach.

Here are important points about side effects:

- The study doctors do not know who will or will not have side effects.
- Some side effects may go away soon, some may last a long time, or some may never go away.
- Some side effects may interfere with your ability to have children.
- Some side effects may be serious and may even result in death.

Here are important points about how you and the study doctor can make side effects less of a problem:

- Tell the study doctor if you notice or feel anything different so they can see if you are having a side effect.
- The study doctor may be able to treat some side effects.
- The study doctor may adjust the study drugs to try to reduce side effects.

The tables below show the most common and the most serious side effects that researchers know about. There might be other side effects that researchers do not yet know about. If important new side effects are found, the study doctor will discuss these with you."

Please insert this condensed risk profile as the Table of Possible Side Effects for Everolimus (RAD-001) in your ICD.

**COMMON, SOME MAY BE SERIOUS**<br>In 100 people receiving everolimus (RAD-001), more than 20 and up to 100 may have:

• Anemia which may require blood transfusion<br>• Diarrhea<br>• Sores in the mouth which may cause difficulty swallowing<br>• Tiredness<br>• Rash

**OCCASIONAL, SOME MAY BE SERIOUS**<br>In 100 people receiving everolimus (RAD-001), from 4 to 20 may have:
• Pain<br>• Constipation, nausea, vomiting<br>• Swelling of arms, legs<br>• Fever<br>• Infection, especially when white blood cell count is low<br>• Bruising, bleeding<br>• Weight loss, loss of appetite<br>• Changes in taste<br>• Headache<br>• Cough, shortness of breath<br>• Nose bleed<br>• Damage to the lungs which may cause shortness of breath<br>• Dry skin<br>• Itching

**RARE, AND SERIOUS**<br>In 100 people receiving everolimus (RAD-001), 3 or fewer may have:
• Non-healing surgical site<br>• Kidney damage which may require dialysis<br>• Allergic reaction which may cause rash, low blood pressure, wheezing, shortness of breath, swelling of the face or throat

Action Letter

Attachment 3: Action Letter GENERAL INSTRUCTIONS

1. **Distribute this Action Letter (and any accompanying CTEP-approved amendment) to all participating investigators and IRBs within 2 working days.** For National Clinical Trials Network (NCTN) studies, please follow instructions from your Operations office. For sites participating in the NCI Central-IRB (CIRB) Initiative, CTEP has provided a copy of this Action Letter to the CIRB if the Action Letter affects any studies under CIRB review.

2. Accrual of new patients must be suspended until the IRB of record has reviewed and approved a CTEP-approved amendment created in response to this Action Letter. This amendment can undergo expedited approval at the discretion of the IRB Chair/designee as explained on the first page of the Action Letter.

3. **Patients currently on study may continue on study provided they are informed of the new and/or modified risk information.** This information should be communicated to patients already enrolled on study without waiting for IRB review/approval since this information represents a significant new finding(s) that developed during the course of the research that may relate to a patient's willingness to continue participation and per the Office for Human Research Protections, the regulations do not require IRB review and approval of statements describing such significant new findings before they are provided to already enrolled patients. Documentation of their informed consent should be carried out according to local IRB requirements.

4. **Save a copy of the Action Letter (and any CTEP approval letter for an accompanying amendment) for your records.**

INSTRUCTIONS FOR PREPARATION OF AN AMENDMENT IN RESPONSE TO THIS Action Letter (if a CTEP-Approved amendment for your trial does not already accompany the Action Letter)

General Instructions on Amendment Preparation:

1. Instructions regarding the due date for an amendment and where to send it are included on the first page of this Action Letter. The Clinical Trials Operations Offices may use the *Detailed Description of Required Protocol Changes* section in this Action Letter as the template for their Change Memo; however, it is not required if an Operations Office prefers to use its own Change Memo.
2. If any of the NCI-required changes are not applicable for your trial (i.e., already appear in your protocol), note this in your Change Memo.
3. **The ICD changes include CTEP's suggested lay terms for each adverse event identified in the CAEPR. You may substitute different lay terms for each concept if appropriate for your patient population. If you have chosen to reformat and/or reword the risk profile in any way, please state, "The risk profile has been modified" in the cover memo.**

Specific Instructions on Amendment Preparation Based on Protocol Status:

A. Trials with a current CTEP status of **"Active"**
   - Review and follow **ALL** the instructions outlined in this Action Letter.
   - The information provided in this memo outlines the **ONLY** changes that can be made in response to the Action Letter, except for changes that are consistent with CTEP's editorial and administrative update policy.
   - Suspend accrual of new patients until the IRB of record has reviewed and approved a CTEP-approved amendment created in response to this Action Letter.

<u>Action Letter</u>

B. <u>Trials with a current status of **“Approved”, “Temporarily Closed to Accrual and Treatment”, or “Temporarily Closed to Accrual**”</u>
- The protocol and ICD must be revised as per the instructions outlined in the Action Letter in the next revised protocol draft submitted to CTEP. The protocol amendment must be submitted and approved by CTEP <u>before</u> the trial can be activated or re-opened.
- You may include additional non-Action Letter related changes (any type) in your amendment response.

C. <u>Trials with a current CTEP status of **“In Review”**</u>
- The protocol and ICD must be revised as per the instructions outlined in the Action Letter. These changes must be included in the next revised protocol draft submitted to and approved by CTEP <u>before</u> the trial can be activated.
- You may include additional non-Action Letter related changes (any type) in your revision response. Note the inclusion of non-Action Letter related changes may delay approval of your trial.

D. <u>Trials with a current CTEP status of **“Closed to Accrual”**</u>

If your trial is under a CTEP-held IND:
- Review and follow **<u>ALL</u>** the instructions outlined in this Action Letter.
- The information provided in this memo outlines the **<u>ONLY</u>** changes that can be made in response to the Action Letter, except for changes that are consistent with CTEP’s amendment request submission policy.

If your trial is NOT under a CTEP-held IND:
- If Action Letter INCLUDES information that impacts patient care (e.g., new/adjusted dose modifications or special monitoring for patient population at risk) - An amendment is required. Review and follow **<u>ALL</u>** the instructions outlined in this Action Letter. The information provided in this memo outlines the **<u>ONLY</u>** changes that can be made in response to the Action Letter, except for changes that are consistent with CTEP’s amendment request submission policy.
  - **If Action Letter does NOT INCLUDE information that impacts patient care - Amendment is typically NOT required.**

E. <u>Trials with a current CTEP status of **“Closed to Accrual and Treatment”** or **“Complete”**</u>
- Amendment is not required. This information is being sent for informational purposes only. You may follow local IRB or Operations Office procedures and requirements.

SWOG
Leading cancer research. Together.

Distribution Date: December 1, 2016
E-mailed Date: November 22, 2016
CTEP Submission Date: November 11, 2016

TO: ALL NATIONAL CLINICAL TRIALS NETWORK (NCTN) MEMBERS – U.S. INSTITUTIONS ONLY

FROM: Nicki Trevino, Protocol Coordinator (E-mail: ntrevino@swog.org)

RE: **S0931**, "EVEREST: EVErolimus for Renal Cancer Ensuing Surgical Therapy, A Phase III Study. Study Chairs: Drs. C.W. Ryan, E.I. Heath, P.N. Lara, G.S. Palapattu, and P.C. Mack.

REVISION #8

Study Chair: Christopher W. Ryan, M.D.
Phone number: 503/494-8487
E-mail: ryanc@ohsu.edu

IRB Review Requirements
( ) Full board review required
(√) Expedited review allowed
( ) No review required

Status Change
( ) IRB Review only
( ) Activation
( ) Closure
( ) Reactivation

Protocol changes
( ) Eligibility changes
( ) Treatment / Dose Modification / Study Calendar changes
(√) Informed Consent changes
 ( ) Patient notification not required
 (√) Patient notification required: see below for details.
( ) Scientific / Statistical Consideration changes
( ) Specimen Submission changes
( ) Data Submission / Forms changes
( ) Editorial / Administrative changes
( ) Other:

REVISION #8

The above-referenced study has been updated as follows:

This revision has been prepared in response to the Request for Rapid Amendment (RRA) for Everolimus (NSC- 733504) (IND-115643) from Dr. Austin Doyle (doylela@mail.nih.gov), Dr. James Zwiebel (zwiebelj@ctep.nci.nih.gov), and Dr. Meg Mooney (mooneym@ctep.nci.nih.gov) on October 12, 2016. The associated action letter is attached.

1. The Version Dates of the protocol and Model Consent Form have been updated.

GROUP CHAIR'S OFFICE
Charles D. Blanke, MD
CHAIR
3181 SW Sam Jackson Pk Rd
MC: L586
Portland, OR 97239
503-494-5586
503-346-8038 FAX

OPERATIONS OFFICE
4201 Medical Dr
Suite 250
San Antonio, TX 78229
210-614-8808
210-614-0006 FAX

STATISTICAL CENTER
1730 Minor Ave
Suite 1900
Seattle, WA 98101
206-652-2267
206-34[illegible] FAX

1100 Fair[illegible]w Ave[illegible]th
M3-C102
PO Box 19024
Seattle, WA 98109
206-667-4623
206-667-4408 FAX

swog.org

CLOSED EFFECTIVE 09/15/2016

SWOG Authorized Document

2. Table of Contents: The page numbers have been updated.

3. Section 3.1c, the CAEPR for Everolimus, Version 2.2, March 21, 2016 has been replaced with Version 2.3, June 30, 2016. The section has been updated as follows:

- Added New Risk:
  - Reported but with Insufficient Evidence for Attribution: Ascites; Blood and lymphatic system disorders - Other (thrombotic microangiopathy); Bronchopulmonary hemorrhage; Cardiac disorders - Other (myocardial abnormality); Chest wall pain; Colitis; Delirium; Edema trunk; Encephalopathy; Endocrine disorders - Other (low testosterone); Flu like symptoms; Gastroesophageal reflux disease; Gastrointestinal disorders - Other (Dieulafoy's lesion); Generalized muscle weakness; Genital edema; Glucose intolerance; Hematuria; Hepatobiliary disorders - Other (hepatomegaly); Hydrocephalus; Hypomagnesemia; Hypothyroidism; INR increased; Intra-abdominal hemorrhage; Investigations - Other (low density lipoprotein raised); Investigations - Other (thrombocythemia); Keratitis; Left ventricular systolic dysfunction; Lethargy; Mania; Metabolism and nutrition disorders - Other (high ammonia); Muscle weakness lower limb; Myalgia; Pancreatitis; Respiratory, thoracic and mediastinal disorders - Other (rales); Sinus bradycardia; Supraventricular tachycardia; Toothache; Vascular disorders - Other (acute bowel ischemia); Voice alteration.
- Increase in Risk Attribution:
  - Changed to Less Likely from Reported with Insufficient Evidence for Attribution: Constipation
- Decrease in Risk Attribution:
  - Changed to Less Likely from Likely: Aspartate aminotransferase increased; Cholesterol high; Hyperglycemia; Hypertriglyceridemia; lymphocyte count decreased; Platelet count decreased
  - Changed to Reported with Insufficient Evidence for Attribution from Less Likely: Activated partial thromboplastin time prolonged; Dizziness; Dry mouth; Hypoalbuminemia; Hypocalcemia; Hypokalemia; Insomnia; Investigations - Other (bicarbonate decreased); Rash acneiform; Skin and subcutaneous tissue disorders - Other (nail disorder)
- Deleted Risk:
  - Reported But With Insufficient Evidence for Attribution: Hypernatremia
- Provided Further Clarification:
  - Nasal congestion (under Also Reported But With Insufficient Evidence for Attribution) is now reported as Respiratory, thoracic and mediastinal disorders - Other (rhinorrhea).

CLOSED EFFECTIVE 09/15/2016

Model Consent Form Changes

Patients currently receiving everolimus **must** be informed of the bolded changes below. The manner by which this notification takes place is at the discretion of the local institution. At a minimum, the patient must be notified by the next visit and this notification must be documented in the patient chart. Patients need not be informed of the non-bolded changes unless required by the local IRB.

1. The following changes have been made to the risk tables for Everolimus in the Model Consent Form:

- Increase in Risk Attribution:
  - Added to Occasional: **Constipation**
- Decrease In Risk Attribution:
  - Changed to Occasional from Common: Bruising, Bleeding

SWOG Authorized Document

- Removed from risk tables: Acne; Change in or loss of some or all of the finger or toenails; Difficulty sleeping; Dizziness; Dry mouth

This memorandum serves to notify the NCI and the SWOG Statistical Center.

cc: PROTOCOL & INFORMATION OFFICE
Meredith Lavin - Novartis
Jason Koury, Pharm.D. - VACSP
Sharon Jenkins - VACSP

CLOSED EFFECTIVE 09/15/2016

SWOG Authorized Document

DEPARTMENT OF HEALTH & HUMAN SERVICES
Public Health Service

National Institutes of Health
National Cancer Institute
Bethesda, Maryland 20892

Action Letter

DATE: **November 21, 2016**

FROM: L. Austin Doyle, MD, Medical Officer, IDB, CTEP, DCTD, NCI
James Zwiebel, MD, Branch Chief, IDB, CTEP, DCTD, NCI
Meg Mooney, MD, Branch Chief, CIB, CTEP, DCTD, NCI

SUBJECT: **CONFIDENTIAL COMMUNICATION** – Action Letter for Everolimus (RAD-001, NSC 733504)

TO: Investigators for CTEP-supported Studies Involving Everolimus (RAD-001, NSC 733504)

The purpose of this Action Letter is to alert patients and investigators in studies conducted by the Cancer Therapy Evaluation Program (CTEP), National Cancer Institute (NCI), of new and/or modified risk information associated with everolimus, and to request all trials with everolimus be amended to reflect these changes. You are receiving this letter because you are conducting a CTEP-sponsored trial that includes everolimus. See the accompanying list of CTEP trials with everolimus.

Although there is modified risk information for everolimus, the added risks are very similar to risks that were already included in the previous version of the CAEPR and would have been communicated to patients in the informed consent document (ICD). In this case, (1) constipation is associated with previously identified risks, abdominal pain and vomiting; and (2) a decrease in the frequency of aspartate aminotransferase increased, cholesterol high, lymphocyte count decreased, platelet count decreased, hyperglycemia and hypertriglyceridemia resulted in the risks being moved from likely to less likely in the CAEPR.

When changes such as these are made to the ICD (i.e., changes as to how risk information is presented and/or additional clarifying information), it is not necessary to suspend enrollment of new subjects until a revised ICD is reviewed and approved by the IRB. For this requested amendment, patient enrollment may continue before the IRB reviews and approves such changes to the ICD; however, changes to the ICDs cannot be implemented until they are approved by the IRB. An amendment that includes the new version of the CAEPR for everolimus and this additional clarification of risks in the ICD must be included in a protocol amendment as outlined in this Action Letter and per the time-lines specified.

In response to the new/modified risk information CTEP is requiring that all trials with everolimus be amended to reflect this new information. Detailed description of the new/modified risk(s) as well as detailed instructions regarding amendment requirements are described in this Action Letter. **Amendments are due to the Protocol and Information Office (PIO) at PIO@CTEP.NCI.NIH.GOV by 5 PM ET on December 2, 2016** or as required based on protocol status (see the *General Actions Required Based on Protocol Status* section). The cover letter for the submitted amendment should state that it is being submitted in response to an Action Letter from Dr. L. Austin Doyle (doylela@mail.nih.gov). Failure to respond in a timely fashion may result in suspension of the Principal Investigator or permanent study closure.

Action Letter

After review of all the available data, CTEP believes that the new and/or modified risk information does **NOT** significantly alter the risk-benefit profile for patients in the study since everolimus is already known to cause serious adverse events and this new risk information does not change the overall weight given to risks versus benefits for patients in the study. CTEP considers all the proposed protocol and informed consent changes for studies affected by this Action Letter to be minor. Therefore, under the provisions of Department of Health and Human Services regulations for the protection of human subjects at 45 CFR 46.110, a protocol amendment that includes this new information can undergo expedited review if the Institutional Review Board (IRB) Chair (or other experienced IRB member designated to conduct expedited review by the Chair) concurs that the changes are minor. Additional information from the Office of Human Research Protections (OHRP) regarding this process is available at: http://www.hhs.gov/ohrp/policy/Correspondence/nci200870929.html.

The following section, *Specific Instruction*, includes background information on the risk(s), any risk mitigation strategies, and amendment requirements. The revised Comprehensive Adverse Events and Potential Risks (CAEPR) list (Attachment 1) and Informed Consent Document (ICD) risk information (Attachment 2) are also attached. Action Letter general instructions as well as instructions regarding amendment preparation (if a CTEP-approved amendment does not already accompany this Action Letter) are included in Attachment 3. **You MUST follow the instructions outlined in Attachment 3.**

Action Letter

SPECIFIC INSTRUCTION

Background

As part of Good Clinical Practice, CTEP reviews each CAEPR list on an annual basis. The review includes literature search, CTEP-AERS submission review, and comparison to the latest agent Investigator's Brochure. After review of all the available data, CTEP has identified new and/or modified risk information associated with everolimus.

Detailed Description of Required Protocol Changes for the Amendment/ Sample Change Memo Template

1) New Protocol Amendment/Version Date Included on the Title/Cover Page per Operations Office Policy:

   Protocol Cover Page: Page Number(s): ______
   Version Date: __________

2) Revision of the Protocol CAEPR:

   Protocol Section(s) for Insertion of Revised CAEPR (Version 2.3, June 30, 2016): ___
   Page Number(s): ____

- Added New Risk:
  - Also Reported on Everolimus Trials But With Insufficient Evidence for Attribution: Ascites; Blood and lymphatic system disorders - Other (thrombotic microangiopathy); Bronchopulmonary hemorrhage; Cardiac disorders - Other (myocardial abnormality); Chest wall pain; Colitis; Delirium; Edema trunk; Encephalopathy; Endocrine disorders - Other (low testosterone); Flu like symptoms; Gastroesophageal reflux disease; Gastrointestinal disorders - Other (Dieulafoy's lesion); Generalized muscle weakness; Genital edema; Glucose intolerance; Hematuria; Hepatobiliary disorders - Other (hepatomegaly); Hydrocephalus; Hypomagnesemia; Hypothyroidism; INR increased; Intra-abdominal hemorrhage; Investigations - Other (low density lipoprotein raised); Investigations - Other (thrombocythemia); Keratitis; Left ventricular systolic dysfunction; Lethargy; Mania; Metabolism and nutrition disorders - Other (high ammonia); Muscle weakness lower limb; Myalgia; Pancreatitis; Respiratory, thoracic and mediastinal disorders - Other (rales); Sinus bradycardia; Supraventricular tachycardia; Toothache; Vascular disorders - Other (acute bowel ischemia); Voice alteration

- Increase in Risk Attribution:
  - Changed to Less Likely from Also Reported on Everolimus Trials But With Insufficient Evidence for Attribution: Constipation

- Decrease in Risk Attribution:
  - Changed to Less Likely from Likely: Aspartate aminotransferase increased; Cholesterol high; Hyperglycemia; Hypertriglyceridemia; Lymphocyte count decreased; Platelet count decreased
  - Changed to Also Reported on Everolimus Trials But With Insufficient Evidence for Attribution from Less Likely: Activated partial thromboplastin time prolonged; Dizziness; Dry mouth; Hypoalbuminemia; Hypocalcemia; Hypokalemia; Insomnia; Investigations - Other (bicarbonate decreased); Rash acneiform; Skin and subcutaneous tissue disorders - Other (nail disorder)

- Deleted Risk:
  - Also Reported on Everolimus Trials But With Insufficient Evidence for Attribution: Hypernatremia

- Provided Further Clarification:
  - Nasal congestion (under Also Reported But With Insufficient Evidence for Attribution) is now reported as Respiratory, thoracic and mediastinal disorders - Other (rhinorrhea).

PLEASE NOTE: The specific detailed changes listed here compare the new revised CAEPR Version 2.3, and associated risk information for the ICD, to the most recent CAEPR Version 2.2. If your trial contains an older CAEPR version (i.e., does **NOT** currently contain CAEPR Version 2.2), you **MUST** include a description of any additional changes resulting from migration from the older CAEPR version.

3) Revision of the ICD as Specified Below:

The terminology for CTEP's suggested lay terms may change periodically. The condensed risk profile represents CAEPR risks in lay terms in a "patient-friendly" condensed form. It is provided as a guide and its use is completely optional; however, all risks listed in the current CAEPR must be reflected in the ICD. Expanding or condensing similar terms is acceptable. If you have chosen to reformat and/or reword the condensed risk profile in any way, please state, "The condensed risk profile has been modified" in the cover memo.

- Increase in Risk Attribution:
  - Changed to Occasional from Also Reported on Everolimus Trials But With Insufficient Evidence for Attribution (i.e., added to the Risk Profile): Constipation

- Decrease in Risk Attribution:
  - Changed to Occasional from Common: Bruising, bleeding
  - Changed to Also Reported on Everolimus Trials But With Insufficient Evidence for Attribution from Occasional (i.e., deleted from the Risk Profile): Acne; Change in or loss of some or all of the finger or toenails; Difficulty sleeping; Dizziness; Dry mouth

PLEASE NOTE: The potential risks listed in the CAEPR whose relationship to everolimus is still undetermined are not required by CTEP to be described in the ICD; however, they may be communicated to patients according to local IRB requirements.

Action Letter

Attachment 1: Revised Everolimus CAEPR – Version 2.3, June 30, 2016

Comprehensive Adverse Events and Potential Risks list (CAEPR) for Everolimus (RAD-001, NSC 733504)

The Comprehensive Adverse Events and Potential Risks list (CAEPR) provides a single list of reported and/or potential adverse events (AE) associated with an agent using a uniform presentation of events by body system. In addition to the comprehensive list, a subset, the Specific Protocol Exceptions to Expedited Reporting (SPEER), appears in a separate column and is identified with bold and italicized text. This subset of AEs (SPEER) is a list of events that are protocol specific exceptions to expedited reporting to NCI (except as noted below). Refer to the 'CTEP, NCI Guidelines: Adverse Event Reporting Requirements' http://ctep.cancer.gov/protocolDevelopment/electronic_applications/docs/aeguidelines.pdf for further clarification. *Frequency is provided based on 3033 patients.* Below is the CAEPR for Everolimus (RAD-001).

NOTE: Report AEs on the SPEER **ONLY IF** they exceed the grade noted in parentheses next to the AE in the SPEER. If this CAEPR is part of a combination protocol using multiple investigational agents and has an AE listed on different SPEERs, use the lower of the grades to determine if expedited reporting is required.

Version 2.3, June 30, 2016[1]

**Adverse Events with Possible Relationship to Everolimus (RAD-001) (CTCAE 4.0 Term) [n= 3033]**			**Specific Protocol Exceptions to Expedited Reporting (SPEER)**
**Likely (>20%)**	**Less Likely (<=20%)**	**Rare but Serious (<3%)**	
BLOOD AND LYMPHATIC SYSTEM DISORDERS			
Anemia			***Anemia (Gr 2)***
GASTROINTESTINAL DISORDERS			
	Abdominal pain		
	Constipation		
Diarrhea[2]			***Diarrhea[2] (Gr 2)***
Mucositis oral[3]			***Mucositis oral[3] (Gr 2)***
	Nausea		***Nausea (Gr 2)***
	Vomiting		***Vomiting (Gr 2)***
GENERAL DISORDERS AND ADMINISTRATION SITE CONDITIONS			
	Edema limbs		***Edema limbs (Gr 2)***
Fatigue			***Fatigue (Gr 2)***
	Fever		***Fever (Gr 2)***
INFECTIONS AND INFESTATIONS			
	Infection[4]		***Infection[4] (Gr 2)***
INJURY, POISONING AND PROCEDURAL COMPLICATIONS			
		Wound complication[5]	
INVESTIGATIONS			
	Alanine aminotransferase increased		***Alanine aminotransferase increased (Gr 2)***
	Alkaline phosphatase increased		***Alkaline phosphatase increased (Gr 2)***
	Aspartate aminotransferase increased		***Aspartate aminotransferase increased (Gr 2)***
	Cholesterol high		***Cholesterol high (Gr 2)***
	Creatinine increased		***Creatinine increased (Gr 2)***
	Lymphocyte count decreased		***Lymphocyte count decreased (Gr 2)***
	Neutrophil count decreased		***Neutrophil count decreased (Gr 2)***
	Platelet count decreased		***Platelet count decreased (Gr 2)***
	Weight loss		

Action Letter

Adverse Events with Possible Relationship to Everolimus (RAD-001) (CTCAE 4.0 Term) [n= 3033]			Specific Protocol Exceptions to Expedited Reporting (SPEER)
**Likely (>20%)**	**Less Likely (<=20%)**	**Rare but Serious (<3%)**	
	White blood cell decreased		*White blood cell decreased (Gr 2)*
METABOLISM AND NUTRITION DISORDERS			
	Anorexia		*Anorexia (Gr 2)*
	Hyperglycemia[6]		*Hyperglycemia[6] (Gr 2)*
	Hypertriglyceridemia		*Hypertriglyceridemia (Gr 2)*
	Hypophosphatemia		*Hypophosphatemia (Gr 2)*
MUSCULOSKELETAL AND CONNECTIVE TISSUE DISORDERS			
	Arthralgia		
	Back pain		
	Pain in extremity		
NERVOUS SYSTEM DISORDERS			
	Dysgeusia		
	Headache		*Headache (Gr 2)*
RENAL AND URINARY DISORDERS			
		Acute kidney injury	
RESPIRATORY, THORACIC AND MEDIASTINAL DISORDERS			
	Cough		*Cough (Gr 2)*
	Dyspnea		*Dyspnea (Gr 2)*
	Epistaxis		*Epistaxis (Gr 2)*
	Pneumonitis[7]		
SKIN AND SUBCUTANEOUS TISSUE DISORDERS			
	Dry skin		
	Pruritus		
Rash maculo-papular			*Rash maculo-papular (Gr 2)*

[1]This table will be updated as the toxicity profile of the agent is revised. Updates will be distributed to all Principal Investigators at the time of revision. The current version can be obtained by contacting PIO@CTEP.NCI.NIH.GOV. Your name, the name of the investigator, the protocol and the agent should be included in the e-mail.

[2]Includes diarrhea, enteritis, enterocolitis, colitis, defecation urgency, and steatorrhea.

[3]Includes stomatitis, aphthous stomatitis, gingival pain/swelling/ulceration, glossitis, glossodynia, lip ulceration, mouth ulceration, tongue ulceration, and mucosal inflammation.

[4]Infection includes all 75 infection sites under the INFECTIONS AND INFESTATIONS SOC.

[5]Everolimus delays wound healing and increases the occurrence of wound-related complications like wound dehiscence, wound infection, incisional hernia, lymphocele, and seroma.

[6]Hyperglycemia may result in either exacerbation of or development new onset diabetes mellitus.

[7]Includes pneumonitis, interstitial lung disease, lung infiltration, pulmonary alveolar hemorrhage, pulmonary toxicity, alveolitis, pulmonary fibrosis, and restrictive pulmonary disease.

[8]Includes agitation, anxiety, panic attack, aggression, abnormal behavior, and obsessive compulsive disorder.

[9]Patients taking concomitant ACE inhibitor therapy may be at increased risk for angioedema.

Adverse events reported on Everolimus (RAD-001) trials, but for which there is insufficient evidence to suggest that there was a reasonable possibility that Everolimus (RAD-001) caused the adverse event:

BLOOD AND LYMPHATIC SYSTEM DISORDERS - Blood and lymphatic system disorders - Other (thrombotic microangiopathy)
CARDIAC DISORDERS - Atrial fibrillation; Cardiac disorders - Other (myocardial abnormality); Chest pain - cardiac; Heart failure; Left ventricular systolic dysfunction; Myocardial infarction; Sinus bradycardia; Sinus tachycardia; Supraventricular tachycardia
ENDOCRINE DISORDERS - Endocrine disorders - Other (increased blood follicle stimulating hormone [FSH] levels); Endocrine disorders - Other (increased blood luteinizing hormone [LH] levels); Endocrine disorders - Other (low testosterone); Hypothyroidism
EYE DISORDERS - Blurred vision; Conjunctivitis; Keratitis
GASTROINTESTINAL DISORDERS - Ascites; Colitis; Dry mouth; Dyspepsia; Dysphagia; Flatulence; Gastroesophageal reflux disease; Gastrointestinal disorders - Other (Dieulafoy's lesion); Hemorrhoids; Intra-abdominal hemorrhage; Oral pain; Pancreatitis; Periodontal disease; Toothache
GENERAL DISORDERS AND ADMINISTRATION SITE CONDITIONS - Chills; Edema face; Edema trunk; Flu like symptoms; Irritability; Non-cardiac chest pain; Pain
HEPATOBILIARY DISORDERS - Hepatic failure; Hepatobiliary disorders - Other (hepatomegaly)
IMMUNE SYSTEM DISORDERS - Allergic reaction
INVESTIGATIONS - Activated partial thromboplastin time prolonged; Blood bilirubin increased; CPK increased; GGT increased; INR increased; Investigations - Other (bicarbonate decreased); Investigations - Other (increased lactate dehydrogenase); Investigations - Other (low density lipoprotein raised); Investigations - Other (thrombocythemia)
METABOLISM AND NUTRITION DISORDERS - Dehydration; Glucose intolerance; Hypercalcemia; Hyperkalemia; Hypoalbuminemia; Hypocalcemia; Hypokalemia; Hypomagnesemia; Hyponatremia; Metabolism and nutrition disorders - Other (high ammonia); Metabolism and nutrition disorders - Other (hyperlipidemia)
MUSCULOSKELETAL AND CONNECTIVE TISSUE DISORDERS - Bone pain; Chest wall pain; Generalized muscle weakness; Muscle weakness lower limb; Musculoskeletal and connective tissue disorder - Other (muscle spasms); Myalgia
NEOPLASMS BENIGN, MALIGNANT AND UNSPECIFIED (INCL CYSTS AND POLYPS) - Neoplasms benign, malignant and unspecified (incl cysts and polyps) - Other (ovarian cysts)
NERVOUS SYSTEM DISORDERS - Dizziness; Encephalopathy; Hydrocephalus; Lethargy; Paresthesia
PSYCHIATRIC DISORDERS - Agitation; Anxiety[8]; Delirium; Depression; Insomnia; Mania
RENAL AND URINARY DISORDERS - Hematuria; Proteinuria; Urinary frequency
REPRODUCTIVE SYSTEM AND BREAST DISORDERS - Dysmenorrhea; Genital edema; Irregular menstruation; Menorrhagia; Vaginal hemorrhage
RESPIRATORY, THORACIC AND MEDIASTINAL DISORDERS - Bronchopulmonary hemorrhage; Pharyngolaryngeal pain; Pleural effusion; Respiratory failure; Respiratory, thoracic and mediastinal disorders - Other (rales); Respiratory, thoracic and mediastinal disorders - Other (rhinorrhea); Sore throat; Voice alteration
SKIN AND SUBCUTANEOUS TISSUE DISORDERS - Nail loss; Palmar-plantar erythrodysesthesia syndrome; Rash acneiform; Skin and subcutaneous tissue disorders - Other (angioedema)[9]; Skin and subcutaneous tissue disorders - Other (nail disorder); Skin and subcutaneous tissue disorders - Other (skin lesion); Skin ulceration
VASCULAR DISORDERS - Flushing; Hypertension; Lymphedema; Phlebitis; Thromboembolic event; Vascular disorders - Other (acute bowel ischemia); Vascular disorders - Other (hemorrhage)

Note: Everolimus (RAD-001) in combination with other agents could cause an exacerbation of any adverse event currently known to be caused by the other agent, or the combination may result in events never previously associated with either agent.

Action Letter

Attachment 2: Revised ICD Section(s) for Everolimus

Please note that the terminology for CTEP's suggested lay terms may change periodically. The condensed risk profile represents CAEPR risks in lay terms in a "patient-friendly" condensed format. It is provided as a guide and its use is completely optional; however, all risks listed in the current CAEPR must be reflected in the ICD. Expanding or condensing similar terms is acceptable. If you have chosen to reformat and/or reword the condensed risk profile in any way, please state, "The condensed risk profile has been modified" in the cover memo. Please insert this condensed risk profile as the Table of Possible Side Effects for everolimus in your ICD.

Risk Profile for Everolimus (CAEPR Version 2.3, June 30, 2016)

NOTE: The risk list section in the new NCI Consent Form Template (latest version: May 2013) will include the wording below:

"If you choose to take part in this study, there is a risk that:

- You may lose time at work or home and spend more time in the hospital or doctor's office than usual
- You may be asked sensitive or private questions which you normally do not discuss

The everolimus used in this study may affect how different parts of your body work such as your liver, kidneys, heart, and blood. The study doctor will be testing your blood and will let you know if changes occur that may affect your health.

There is also a risk that you could have side effects from the study drug(s)/study approach.

Here are important points about side effects:

- The study doctors do not know who will or will not have side effects.
- Some side effects may go away soon, some may last a long time, or some may never go away.
- Some side effects may interfere with your ability to have children.
- Some side effects may be serious and may even result in death.

Here are important points about how you and the study doctor can make side effects less of a problem:

- Tell the study doctor if you notice or feel anything different so they can see if you are having a side effect.
- The study doctor may be able to treat some side effects.
- The study doctor may adjust the study drugs to try to reduce side effects.

The tables below show the most common and the most serious side effects that researchers know about. There might be other side effects that researchers do not yet know about. If important new side effects are found, the study doctor will discuss these with you."

Please insert this condensed risk profile as the Table of Possible Side Effects for Everolimus in your ICD.

Action Letter

COMMON, SOME MAY BE SERIOUS<br>In 100 people receiving everolimus, more than 20 and up to 100 may have:
• Anemia which may require blood transfusion<br>• Diarrhea<br>• Sores in the mouth which may cause difficulty swallowing<br>• Tiredness<br>• Rash

OCCASIONAL, SOME MAY BE SERIOUS<br>In 100 people receiving everolimus, from 4 to 20 may have:
• Pain<br>• Constipation, nausea, vomiting<br>• Swelling of the arms, legs<br>• Fever<br>• Infection, especially when white blood cell count is low<br>• Bruising, bleeding<br>• Weight loss, loss of appetite<br>• Changes in taste<br>• Headache<br>• Cough, shortness of breath<br>• Nose bleed<br>• Swelling of the lungs which may cause shortness of breath<br>• Dry skin<br>• Itching

RARE, AND SERIOUS<br>In 100 people receiving everolimus, 3 or fewer may have:
• Non-healing surgical site<br>• Kidney damage which may require dialysis

Action Letter

Attachment 3: Action Letter GENERAL INSTRUCTIONS

1. **Distribute this Action Letter (and any accompanying CTEP-approved amendment) to all participating investigators and IRBs within 2 working days.** For National Clinical Trials Network (NCTN) studies, please follow instructions from your Operations office. For sites participating in the NCI Central-IRB (CIRB) Initiative, CTEP has provided a copy of this Action Letter to the CIRB if the Action Letter affects any studies under CIRB review.

2. **Patients currently on study may continue on study provided they are informed of the new and/or modified risk information.** This information should be communicated to patients already enrolled on study without waiting for IRB review/approval since this information represents a significant new finding(s) that developed during the course of the research that may relate to a patient's willingness to continue participation and per the Office for Human Research Protections, the regulations do not require IRB review and approval of statements describing such significant new findings before they are provided to already enrolled patients. Documentation of their informed consent should be carried out according to local IRB requirements.

3. **Save a copy of the Action Letter (and any CTEP approval letter for an accompanying amendment) for your records.**

INSTRUCTIONS FOR PREPARATION OF AN AMENDMENT IN RESPONSE TO THIS Action Letter (if a CTEP-Approved amendment for your trial does not already accompany the Action Letter)

General Instructions on Amendment Preparation:

1. Instructions regarding the due date for an amendment and where to send it are included on the first page of this Action Letter. The Clinical Trials Operations Offices may use the *Detailed Description of Required Protocol Changes* section in this Action Letter as the template for their Change Memo; however, it is not required if an Operations Office prefers to use its own Change Memo.
2. If any of the NCI-required changes are not applicable for your trial (i.e., already appear in your protocol), note this in your Change Memo.
3. **The ICD changes include CTEP's suggested lay terms for each adverse event identified in the CAEPR. You may substitute different lay terms for each concept if appropriate for your patient population. If you have chosen to reformat and/or reword the risk profile in any way, please state, "The risk profile has been modified" in the cover memo.**

Specific Instructions on Amendment Preparation Based on Protocol Status:

A. Trials with a current CTEP status of **"Active"**
   - Review and follow **ALL** the instructions outlined in this Action Letter.
   - The information provided in this memo outlines the **ONLY** changes that can be made in response to the Action Letter, except for changes that are consistent with CTEP's editorial and administrative update policy.

B. Trials with a current status of **"Approved", "Temporarily Closed to Accrual and Treatment", or "Temporarily Closed to Accrual"**
   - The protocol and ICD must be revised as per the instructions outlined in the Action Letter in the next revised protocol draft submitted to CTEP. The protocol amendment must be submitted and approved by CTEP before the trial can be activated or re-opened.
   - You may include additional non-Action Letter related changes (any type) in your amendment response.

Action Letter

C. Trials with a current CTEP status of **"In Review"**

- The protocol and ICD must be revised as per the instructions outlined in the Action Letter. These changes must be included in the next revised protocol draft submitted to and approved by CTEP before the trial can be activated.
- You may include additional non-Action Letter related changes (any type) in your revision response. Note the inclusion of non-Action Letter related changes may delay approval of your trial.

D. Trials with a current CTEP status of **"Closed to Accrual"**

If your trial is under a CTEP-held IND:

- Review and follow **ALL** the instructions outlined in this Action Letter.
- The information provided in this memo outlines the **ONLY** changes that can be made in response to the Action Letter, except for changes that are consistent with CTEP's amendment request submission policy.

If your trial is NOT under a CTEP-held IND:

- If Action Letter INCLUDES information that impacts patient care (e.g., new/adjusted dose modifications or special monitoring for patient population at risk) - An amendment is required. Review and follow **ALL** the instructions outlined in this Action Letter. The information provided in this memo outlines the **ONLY** changes that can be made in response to the Action Letter, except for changes that are consistent with CTEP's amendment request submission policy.
  - **If Action Letter does NOT INCLUDE information that impacts patient care - Amendment is typically NOT required.**

E. Trials with a current CTEP status of **"Closed to Accrual and Treatment"** or **"Complete"**

- Amendment is not required. This information is being sent for informational purposes only. You may follow local IRB or Operations Office procedures and requirements.

SWOG
Leading cancer research. Together.

GROUP CHAIR'S OFFICE
Charles D. Blanke, MD
CHAIR
3181 SW Sam Jackson Pk Rd
MC: L586
Portland, OR 97239
503-494-5586
503-346-8038 FAX

OPERATIONS OFFICE
4201 Medical Dr
Suite 250
San Antonio, TX 78229
210-614-8808
210-614-0006 FAX

STATISTICAL CENTER
1730 Minor Ave
Suite 1900
Seattle, WA 98101
206-652-2267
206-34[illegible] FAX

1100 Fair[illegible]ve[illegible]th
M3-C102
PO Box 19024
Seattle, WA 98109
206-667-4623
206-667-4408 FAX

swog.org

CLOSED EFFECTIVE 09/15/2016

November 1, 2016

TO: ALL NATIONAL CLINICAL TRIALS NETWORK (NCTN) MEMBERS

FROM: SWOG Operations Office

RE: IND Safety Reports for Everolimus

MEMORANDUM

IRB Review Requirements
( ) Full board review required
(√) Expedited review allowed
( ) No review required

Status Change
( ) IRB Review only
( ) Activation
( ) Closure
( ) Reactivation

Protocol changes
( ) Eligibility changes
( ) Treatment / Dose Modification / Study Calendar changes
( ) Informed Consent changes
  ( ) Patient notification not required
  ( ) Patient notification required
( ) Scientific / Statistical Consideration changes
( ) Specimen Submission changes
( ) Data Submission / Forms changes
( ) Editorial / Administrative changes
( ) Other:

MEMORANDUM

The following new safety report has been posted regarding an adverse event that occurred in association with the drug everolimus. Please access this safety report via the study's abstract page or the safety report link on the SWOG website (https://swog.org/safetyreports/safetyreports.asp).

This safety report pertains to the following studies:	Reports:
**S0528** Early Therapeutics	Sep. 21, 2016 Mfr Rpt #PHHO2016US012541
**S0931** Genitourinary	
**S1207** Breast	
**S1222** Breast	

Protocol amendments are not necessary at this time, but your consent form may be revised to include the information provided in this report if it is deemed necessary by your institution. Please append this notice and this report to your copy of the protocol and forward copies to your Institutional Review Board (IRB) as required by your local policies and procedures. Should any further information regarding these adverse events be made available, it will be forwarded to you.

SWOG Authorized Document

This memorandum serves to notify the NCI and the SWOG Statistical Center.

cc: PROTOCOL & INFORMATION OFFICE
Carol French – AstraZeneca
John Parnell – AstraZeneca
Jean Sowa – AstraZeneca
Meredith Lavin – Novartis
Kristin White – Novartis
Rebecca DeLos Rios - Novartis
Heather Campbell, PharmD – VACSP/PCC
Anne Davis – VACSP/PCC
Sharon Jenkins – VACSP/PCC
James Rasp – VACSP/PCC
Emily Tabinski – VACSP/PCC
Jason Koury – VACSP/PCC
Jeffrey Huminik – VACSP/PCC

CLOSED EFFECTIVE 09/15/2016

SWOG Authorized Document

SWOG
Leading cancer research. Together.

GROUP CHAIR'S OFFICE
Charles D. Blanke, MD
CHAIR
3181 SW Sam Jackson Pk Rd
MC: L586
Portland, OR 97239
503-494-5586
503-346-8038 FAX

OPERATIONS OFFICE
4201 Medical Dr
Suite 250
San Antonio, TX 78229
210-614-8808
210-614-0006 FAX

STATISTICAL CENTER
1730 Minor Ave
Suite 1900
Seattle, WA 98101
206-652-2267
206-342-1616 FAX

1100 Fairview Ave North
M3-C102
PO Box 19024
Seattle, WA 98109
206-667-4623
206-667-4408 FAX

swog.org

September 1, 2016

TO: ALL NATIONAL CLINICAL TRIALS NETWORK (NCTN) MEMBERS – U.S. INSTITUTIONS ONLY

FROM: Nicki Trevino, Protocol Coordinator (E-mail: ntrevino@swog.org)

RE: **S0931**, "EVEREST: EVErolimus for Renal Cancer Ensuing Surgical Therapy, A Phase III Study. Study Chairs: Drs. C.W. Ryan, E.I. Heath, P.N. Lara, G.S. Palapattu, and P.C. Mack.

MEMORANDUM

Study Chair: Christopher W. Ryan, M.D.
Phone number: 503/494-8487
E-mail: ryanc@ohsu.edu

IRB Review Requirements
( ) Full board review required
( ) Expedited review allowed
(√) No review required

Status Change
( ) IRB Review only
( ) Activation
( ) Closure
( ) Reactivation

Protocol changes
( ) Eligibility changes
( ) Treatment / Dose Modification / Study Calendar changes
( ) Informed Consent changes
  ( ) Patient notification not required
  ( ) Patient notification required
( ) Scientific / Statistical Consideration changes
( ) Specimen Submission changes
( ) Data Submission / Forms changes
( ) Editorial / Administrative changes
( ) Other:

MEMORANDUM

The purpose of this memorandum is to correct an error in the Revision #7 cover memo that was distributed on July 1st. That memo indicated that patients need not be notified of the changes. It has come to our attention that, despite the CIRB's assessment that patients need not be notified of the changes in this revision, CTEP's Action Letter **does** require patient notification.

Therefore, patients currently receiving everolimus/plecebo and patients who sign a consent form prior to the revised consent being implemented must be informed of the new and modified risk information outlined in Revision #7.

SWOG Authorized Document

The manner by which the notification takes place is at the discretion of the local institution. At a minimum, the patient must be notified by the next visit and this notification process must be documented in the patient chart.

This memorandum serves to notify the NCI and the SWOG Statistical Center.

cc: PROTOCOL & INFORMATION OFFICE
Meredith Lavin - Novartis
Jason Koury, Pharm.D. - VACSP
Sharon Jenkins - VACSP

SWOG Authorized Document

SWOG
Leading cancer research. Together.

GROUP CHAIR'S OFFICE
Charles D. Blanke, MD
CHAIR
3181 SW Sam Jackson Pk Rd
MC: L586
Portland, OR 97239
503-494-5586
503-346-8038 FAX

OPERATIONS OFFICE
4201 Medical Dr
Suite 250
San Antonio, TX 78229
210-614-8808
210-614-0006 FAX

STATISTICAL CENTER
1730 Minor Ave
Suite 1900
Seattle, WA 98101
206-652-2267
206-342-1616 FAX

1100 Fairview Ave North
M3-C102
PO Box 19024
Seattle, WA 98109
206-667-4623
206-667-4408 FAX

swog.org

August 1, 2016

TO: ALL NATIONAL CLINICAL TRIALS NETWORK (NCTN) MEMBERS – U.S. INSTITUTIONS ONLY

FROM: SWOG Operations Office (E-mail: protocols@swog.org)

RE: S0931, "EVEREST: EVErolimus for Renal Cancer Ensuing Surgical Therapy, A Phase III Study. Study Chairs: Drs. C.W. Ryan, E.I. Heath, P.N. Lara, G.S. Palapattu, and P.C. Mack.

STATUS NOTICE

Study Chair: Christopher W. Ryan, M.D.
Phone number: 503/494-8487
E-mail: ryanc@ohsu.edu

IRB Review Requirements
( ) Full board review required
(√) Expedited review allowed
( ) No review required

Status Change
( ) IRB Review only
( ) Activation
(√) Closure - Permanent
( ) Reactivation

Protocol changes
( ) Eligibility changes
( ) Treatment / Dose Modification / Study Calendar changes
( ) Informed Consent changes
    ( ) Patient notification not required
    ( ) Patient notification required
( ) Scientific / Statistical Consideration changes
( ) Specimen Submission changes
( ) Data Submission / Forms changes
( ) Editorial / Administrative changes
( ) Other:

PERMANENT CLOSURE

The above-referenced study has met its accrual goal and will permanently close to accrual **effective September 15, 2016 at 11:59 p.m. Pacific**

This memorandum serves to notify the NCI and the SWOG Statistical Center.

cc: PROTOCOL & INFORMATION OFFICE
Meredith Lavin - Novartis
Jason Koury, Pharm.D. - VACSP
Sharon Jenkins - VACSP

SWOG Authorized Document

SWOG
Leading cancer research. Together.

GROUP CHAIR'S OFFICE
Charles D. Blanke, MD
CHAIR
3181 SW Sam Jackson Pk Rd
MC: L586
Portland, OR 97239
503-494-5586
503-346-8038 FAX

OPERATIONS OFFICE
4201 Medical Dr
Suite 250
San Antonio, TX 78229
210-614-8808
210-614-0006 FAX

STATISTICAL CENTER
1730 Minor Ave
Suite 1900
Seattle, WA 98101
206-652-2267
206-342-1616 FAX

1100 Fairview Ave North
M3-C102
PO Box 19024
Seattle, WA 98109
206-667-4623
206-667-4408 FAX

swog.org

Distribution Date: July 1, 2016
CTEP Submission Date: June 6, 2016

TO: ALL NATIONAL CLINICAL TRIALS NETWORK (NCTN) MEMBERS – U.S. INSTITUTIONS ONLY

FROM: SWOG Operations Office (E-mail: protocols@swog.org)

RE: **S0931**, "EVEREST: EVErolimus for Renal Cancer Ensuing Surgical Therapy, A Phase III Study. Study Chairs: Drs. C.W. Ryan, E.I. Heath, P.N. Lara, G.S. Palapattu, and P.C. Mack.

REVISION #7

Study Chair: Christopher W. Ryan, M.D.
Phone number: 503/494-8487
E-mail: ryanc@ohsu.edu

IRB Review Requirements
( ) Full board review required
(√) Expedited review allowed
( ) No review required

Status Change
( ) IRB Review only
( ) Activation
( ) Closure
( ) Reactivation

Protocol changes
( ) Eligibility changes
( ) Treatment / Dose Modification / Study Calendar changes
(√) Informed Consent changes
  (√) Patient notification not required
  ( ) Patient notification required
( ) Scientific / Statistical Consideration changes
( ) Specimen Submission changes
( ) Data Submission / Forms changes
( ) Editorial / Administrative changes
( ) Other:

REVISION #7

The above noted protocol document has been revised as follows:

The changes in Section 3.1c and the Model Consent Form noted below are in response to a Request for Rapid Amendment (RRA) from Dr. L. Austin Doyle (doylela@mail.nih.gov) received on May 6, 2016. The associated Action Letter is attached.

SWOG Authorized Document

1. Title Page, Page 1: The Version Date of the protocol and model consent form has been updated.

2. Section 3.1c, Pages 12-15: The everolimus adverse event (AE) table has been replaced with a CAEPR Version 2.2 (March 21, 2016). The section has been updated as follows:

    - Increase in Risk Attribution:
        - Changed to Likely from Less Likely: Aspartate aminotransferase increased
        - Changed to Less Likely from Reported but With Insufficient Evidence for Attribution: Hypoalbuminemia

    - Decrease in Risk Attribution:
        - Changed to Reported but With Insufficient Evidence for Attribution from Less Likely: Constipation; Hypertension; Pharyngolaryngeal pain

    - Provided Further Clarification:
        - Respiratory, thoracic and mediastinal disorders - Other (pulmonary toxicity) (under Less Likely) is now reported as Pneumonitis (under Less Likely), and footnote #7 now states "Includes pneumonitis, interstitial lung disease, lung infiltration, pulmonary alveolar hemorrhage, pulmonary toxicity, alveolitis, pulmonary fibrosis, and restrictive pulmonary disease."
        - The following footnote #9, "Patients taking concomitant ACE inhibitor therapy may be at increased risk for angioedema," was added.

    - Modified Specific Protocol Exceptions to Expedited Reporting (SPEER) reporting requirements:
        - Added: Infection

3. Model Consent Form, Page 6: The following changes have been made to the risk tables for everolimus/placebo:

    - Decrease in Risk Attribution:
        - Changed to Reported but With Insufficient Evidence for Attribution from Occasional (i.e., removed from the Risk Profile): Constipation; High blood pressure which may cause blurred vision

    - Provided Further Clarification:
        - Damage to the lungs which may cause difficulty breathing (under Occasional) is now reported as Swelling of the lungs which may cause shortness of breath (under Occasional).

This memorandum serves to notify the NCI and the SWOG Statistical Center.

cc: PROTOCOL & INFORMATION OFFICE
Meredith Lavin - Novartis
Jason Koury, Pharm.D. - VACSP
Sharon Jenkins - VACSP

SWOG Authorized Document

DEPARTMENT OF HEALTH & HUMAN SERVICES Public Health Service

National Institutes of Health
National Cancer Institute
Bethesda, Maryland 20892

Action Letter

DATE: **June 30, 2016**

FROM: L. Austin Doyle, MD, Medical Officer, IDB, CTEP, DCTD, NCI
James Zwiebel, MD, Branch Chief, IDB, CTEP, DCTD, NCI
Meg Mooney, MD, Branch Chief, CIB, CTEP, DCTD, NCI

SUBJECT: **CONFIDENTIAL COMMUNICATION** – Action Letter for Everolimus (RAD-001, NSC 733504)

TO: Investigators for CTEP-supported Studies Involving Everolimus (RAD-001, NSC 733504)

The purpose of this Action Letter is to alert patients and investigators in studies conducted by the Cancer Therapy Evaluation Program (CTEP), National Cancer Institute (NCI), of new and/or modified risk information associated with everolimus, and to request all trials with everolimus be amended to reflect these changes. You are receiving this letter because you are conducting a CTEP-sponsored trial that includes everolimus. See the accompanying list of CTEP trials with everolimus.

Although there is modified risk information for everolimus, the added risks are very similar to risks that were already included in the previous version of the CAEPR and would have been communicated to patients in the informed consent document (ICD). In this case, (1) an increase in the frequency of aspartate aminotransferase increased resulted in the risk being moved from less likely to likely in the CAEPR, and (2) hypoalbuminemia is an abnormal laboratory value that is not included in the ICD, as it is felt that patients would not perceive that they are experiencing this adverse event; also, the consent states that the study intervention may affect how different parts of the body work and that the study doctor will be testing blood and will let patients know if changes occur that may affect their health.

When changes such as these are made to the ICD (i.e., changes as to how risk information is presented and/or additional clarifying information), it is not necessary to suspend enrollment of new subjects until a revised ICD is reviewed and approved by the IRB. For this requested amendment, patient enrollment may continue before the IRB reviews and approves such changes to the ICD; however, changes to the ICDs cannot be implemented until they are approved by the IRB. An amendment that includes the new version of the CAEPR for everolimus and this additional clarification of risks in the ICD must be included in a protocol amendment as outlined in this RRA and per the time-lines specified.

In response to the new/modified risk information CTEP is requiring that all trials with everolimus be amended to reflect this new information. Detailed description of the new/modified risk(s) as well as detailed instructions regarding amendment requirements are described in this Action Letter. **Amendments are due to the Protocol and Information Office (PIO) at PIO@CTEP.NCI.NIH.GOV by 5 PM ET on July 14, 2016** or as required based on protocol status (see the *General Actions Required Based on Protocol Status* section). The cover letter for the submitted amendment should state that it is being submitted in response to an Action Letter from Dr. L. Austin Doyle (doylela@mail.nih.gov). Failure to respond in a timely fashion may result in suspension of the Principal Investigator or permanent study closure.

Action Letter

After review of all the available data, CTEP believes that the new and/or modified risk information does **NOT** significantly alter the risk-benefit profile for patients in the study since everolimus is already known to cause serious adverse events and this new risk information does not change the overall weight given to risks versus benefits for patients in the study. CTEP considers all the proposed protocol and informed consent changes for studies affected by this Action Letter to be minor. Therefore, under the provisions of Department of Health and Human Services regulations for the protection of human subjects at 45 CFR 46.110, a protocol amendment that includes this new information can undergo expedited review if the Institutional Review Board (IRB) Chair (or other experienced IRB member designated to conduct expedited review by the Chair) concurs that the changes are minor. Additional information from the Office of Human Research Protections (OHRP) regarding this process is available at: http://www.hhs.gov/ohrp/policy/Correspondence/nci200870929.html.

The following section, *Specific Instruction*, includes background information on the risk(s), any risk mitigation strategies, and amendment requirements. The revised Comprehensive Adverse Events and Potential Risks (CAEPR) list (Attachment 1) and Informed Consent Document (ICD) risk information (Attachment 2) are also attached. Action Letter general instructions as well as instructions regarding amendment preparation (if a CTEP-approved amendment does not already accompany this Action Letter) are included in Attachment 3. **You MUST follow the instructions outlined in Attachment 3.**

Action Letter

SPECIFIC INSTRUCTION

Background

As part of Good Clinical Practice, CTEP reviews each CAEPR list on an annual basis. The review includes literature search, CTEP-AERS submission review, and comparison to the latest agent Investigator's Brochure. After review of all the available data, CTEP has identified new and/or modified risk information associated with everolimus.

Detailed Description of Required Protocol Changes for the Amendment/ Sample Change Memo Template

1) New Protocol Amendment/Version Date Included on the Title/Cover Page per Operations Office Policy:

    Protocol Cover Page: Page Number(s): ______
    Version Date: __________

2) Revision of the Protocol CAEPR:

    Protocol Section(s) for Insertion of Revised CAEPR (Version 2.2, March 21, 2016): ___
    Page Number(s): ____

    - Increase in Risk Attribution:
        - Changed to Likely from Less Likely: Aspartate aminotransferase increased
        - Changed to Less Likely from Reported but With Insufficient Evidence for Attribution: Hypoalbuminemia
    - Decrease in Risk Attribution:
        - Changed to Reported but With Insufficient Evidence for Attribution from Less Likely: Constipation; Hypertension; Pharyngolaryngeal pain
    - Provided Further Clarification:
        - Respiratory, thoracic and mediastinal disorders - Other (pulmonary toxicity) (under Less Likely) is now reported as Pneumonitis (under Less Likely), and footnote #7 now states "Includes pneumonitis, interstitial lung disease, lung infiltration, pulmonary alveolar hemorrhage, pulmonary toxicity, alveolitis, pulmonary fibrosis, and restrictive pulmonary disease."
        - The following footnote #9, "Patients taking concomitant ACE inhibitor therapy may be at increased risk for angioedema," was added.
    - Modified Specific Protocol Exceptions to Expedited Reporting (SPEER) reporting requirements:
        - Added: Infection

    PLEASE NOTE: The specific detailed changes listed here compare the new revised CAEPR Version 2.2, and associated risk information for the Informed Consent Document (ICD), to the most recent CAEPR Version 2.1. If your trial contains an older CAEPR version (i.e., does **NOT** currently contain CAEPR Version 2.1), you **MUST** include a description of any additional changes resulting from migration from the older CAEPR version.

3) Revision of the ICD as Specified Below:

Action Letter

The terminology for CTEP's suggested lay terms may change periodically. The condensed risk profile represents CAEPR risks in lay terms in a "patient-friendly" condensed form. It is provided as a guide and its use is completely optional; however, all risks listed in the current CAEPR must be reflected in the ICD. Expanding or condensing similar terms is acceptable. If you have chosen to reformat and/or reword the condensed risk profile in any way, please state, "The condensed risk profile has been modified" in the cover memo.

- Decrease in Risk Attribution:
  - Changed to Reported but With Insufficient Evidence for Attribution from Occasional (i.e., removed from the Risk Profile): Constipation; High blood pressure which may cause blurred vision

- Provided Further Clarification:
  - Damage to the lungs which may cause difficulty breathing (under Occasional) is now reported as Swelling of the lungs which may cause shortness of breath (under Occasional).

PLEASE NOTE: The potential risks listed in the CAEPR whose relationship to everolimus is still undetermined are not required by CTEP to be described in the ICD; however, they may be communicated to patients according to local IRB requirements.

Attachment 1: **Revised Everolimus CAEPR – Version 2.2, March 21, 2016**

Comprehensive Adverse Events and Potential Risks list (CAEPR) for Everolimus (RAD-001, NSC 733504)

The Comprehensive Adverse Events and Potential Risks list (CAEPR) provides a single list of reported and/or potential adverse events (AE) associated with an agent using a uniform presentation of events by body system. In addition to the comprehensive list, a subset, the Specific Protocol Exceptions to Expedited Reporting (SPEER), appears in a separate column and is identified with bold and italicized text. This subset of AEs (SPEER) is a list of events that are protocol specific exceptions to expedited reporting to NCI (except as noted below). Refer to the 'CTEP, NCI Guidelines: Adverse Event Reporting Requirements' http://ctep.cancer.gov/protocolDevelopment/electronic_applications/docs/aeguidelines.pdf for further clarification. *Frequency is provided based on 1423 patients.* Below is the CAEPR for Everolimus (RAD-001).

NOTE: Report AEs on the SPEER **ONLY IF** they exceed the grade noted in parentheses next to the AE in the SPEER. If this CAEPR is part of a combination protocol using multiple investigational agents and has an AE listed on different SPEERs, use the lower of the grades to determine if expedited reporting is required.

Version 2.2, March 21, 2016[1]

Adverse Events with Possible Relationship to Everolimus (RAD-001) (CTCAE 4.0 Term) [n= 1423]			Specific Protocol Exceptions to Expedited Reporting (SPEER)
**Likely (>20%)**	**Less Likely (<=20%)**	**Rare but Serious (<3%)**	
BLOOD AND LYMPHATIC SYSTEM DISORDERS			
Anemia			***Anemia (Gr 2)***
GASTROINTESTINAL DISORDERS			
	Abdominal pain		
Diarrhea[2]			***Diarrhea[2] (Gr 2)***
	Dry mouth		
Mucositis oral[3]			***Mucositis oral[3] (Gr 2)***
	Nausea		***Nausea (Gr 2)***
	Vomiting		***Vomiting (Gr 2)***
GENERAL DISORDERS AND ADMINISTRATION SITE CONDITIONS			
	Edema limbs		***Edema limbs (Gr 2)***
Fatigue			***Fatigue (Gr 2)***
	Fever		***Fever (Gr 2)***
INFECTIONS AND INFESTATIONS			
	Infection[4]		***Infection[4] (Gr 2)***
INJURY, POISONING AND PROCEDURAL COMPLICATIONS			
		Wound complication[5]	
INVESTIGATIONS			
	Activated partial thromboplastin time prolonged		
	Alanine aminotransferase increased		***Alanine aminotransferase increased (Gr 2)***
	Alkaline phosphatase increased		***Alkaline phosphatase increased (Gr 2)***
Aspartate aminotransferase increased			***Aspartate aminotransferase increased (Gr 2)***
Cholesterol high			***Cholesterol high (Gr 2)***
	Creatinine increased		***Creatinine increased (Gr 2)***
	Investigations - Other (bicarbonate decreased)		

Action Letter

Adverse Events with Possible Relationship to Everolimus (RAD-001) (CTCAE 4.0 Term) [n= 1423]			Specific Protocol Exceptions to Expedited Reporting (SPEER)
**Likely (>20%)**	**Less Likely (<=20%)**	**Rare but Serious (<3%)**	
Lymphocyte count decreased			***Lymphocyte count decreased (Gr 2)***
	Neutrophil count decreased		***Neutrophil count decreased (Gr 2)***
Platelet count decreased			***Platelet count decreased (Gr 2)***
	Weight loss		
	White blood cell decreased		***White blood cell decreased (Gr 2)***
METABOLISM AND NUTRITION DISORDERS			
	Anorexia		***Anorexia (Gr 2)***
Hyperglycemia[6]			***Hyperglycemia[6] (Gr 2)***
Hypertriglyceridemia			***Hypertriglyceridemia (Gr 2)***
	Hypoalbuminemia		
	Hypocalcemia		
	Hypokalemia		
	Hypophosphatemia		***Hypophosphatemia (Gr 2)***
MUSCULOSKELETAL AND CONNECTIVE TISSUE DISORDERS			
	Arthralgia		
	Back pain		
	Pain in extremity		
NERVOUS SYSTEM DISORDERS			
	Dizziness		
	Dysgeusia		
	Headache		***Headache (Gr 2)***
PSYCHIATRIC DISORDERS			
	Insomnia		
RENAL AND URINARY DISORDERS			
		Acute kidney injury	
RESPIRATORY, THORACIC AND MEDIASTINAL DISORDERS			
	Cough		***Cough (Gr 2)***
	Dyspnea		***Dyspnea (Gr 2)***
	Epistaxis		***Epistaxis (Gr 2)***
	Pneumonitis[7]		
SKIN AND SUBCUTANEOUS TISSUE DISORDERS			
	Dry skin		
	Pruritus		
	Rash acneiform		
Rash maculo-papular			***Rash maculo-papular (Gr 2)***
	Skin and subcutaneous tissue disorders - Other (nail disorder)		

[1]This table will be updated as the toxicity profile of the agent is revised. Updates will be distributed to all Principal Investigators at the time of revision. The current version can be obtained by contacting PIO@CTEP.NCI.NIH.GOV. Your name, the name of the investigator, the protocol and the agent should be included in the e-mail.

[2]Includes diarrhea, enteritis, enterocolitis, colitis, defecation urgency, and steatorrhea.

[3]Includes stomatitis, aphthous stomatitis, gingival pain/swelling/ulceration, glossitis, glossodynia, lip ulceration, mouth ulceration, tongue ulceration, and mucosal inflammation.

[4]Infection includes all 75 infection sites under the INFECTIONS AND INFESTATIONS SOC.

[5]Everolimus delays wound healing and increases the occurrence of wound-related complications like wound dehiscence, wound infection, incisional hernia, lymphocele, and seroma.

[6]Hyperglycemia may result in either exacerbation of or development new onset diabetes mellitus.

[7]Includes pneumonitis, interstitial lung disease, lung infiltration, pulmonary alveolar hemorrhage, pulmonary toxicity, alveolitis, pulmonary fibrosis, and restrictive pulmonary disease.

[8]Includes agitation, anxiety, panic attack, aggression, abnormal behavior, and obsessive compulsive disorder.

[9]Patients taking concomitant ACE inhibitor therapy may be at increased risk for angioedema.

Adverse events reported on Everolimus (RAD-001) trials, but for which there is insufficient evidence to suggest that there was a reasonable possibility that Everolimus (RAD-001) caused the adverse event:

CARDIAC DISORDERS - Atrial fibrillation; Chest pain - cardiac; Heart failure; Myocardial infarction; Sinus tachycardia
ENDOCRINE DISORDERS - Endocrine disorders - Other (increased blood follicle stimulating hormone [FSH] levels); Endocrine disorders - Other (increased blood luteinizing hormone [LH] levels)
EYE DISORDERS - Blurred vision; Conjunctivitis
GASTROINTESTINAL DISORDERS - Constipation; Dyspepsia; Dysphagia; Flatulence; Hemorrhoids; Oral pain; Periodontal disease
GENERAL DISORDERS AND ADMINISTRATION SITE CONDITIONS - Chills; Edema face; Irritability; Non-cardiac chest pain; Pain
HEPATOBILIARY DISORDERS - Hepatic failure
IMMUNE SYSTEM DISORDERS - Allergic reaction
INVESTIGATIONS - Blood bilirubin increased; CPK increased; GGT increased; Investigations - Other (increased lactate dehydrogenase)
METABOLISM AND NUTRITION DISORDERS - Dehydration; Hypercalcemia; Hyperkalemia; Hypernatremia; Hyponatremia; Metabolism and nutrition disorders - Other (hyperlipidemia)
MUSCULOSKELETAL AND CONNECTIVE TISSUE DISORDERS - Bone pain; Musculoskeletal and connective tissue disorder - Other (muscle spasms)
NEOPLASMS BENIGN, MALIGNANT AND UNSPECIFIED (INCL CYSTS AND POLYPS) - Neoplasms benign, malignant and unspecified (incl cysts and polyps) - Other (ovarian cysts)
NERVOUS SYSTEM DISORDERS - Paresthesia
PSYCHIATRIC DISORDERS - Agitation; Anxiety[8]; Depression
RENAL AND URINARY DISORDERS - Proteinuria; Urinary frequency
REPRODUCTIVE SYSTEM AND BREAST DISORDERS - Dysmenorrhea; Irregular menstruation; Menorrhagia; Vaginal hemorrhage
RESPIRATORY, THORACIC AND MEDIASTINAL DISORDERS - Nasal congestion; Pharyngolaryngeal pain; Pleural effusion; Respiratory failure; Sore throat
SKIN AND SUBCUTANEOUS TISSUE DISORDERS - Nail loss; Palmar-plantar erythrodysesthesia syndrome; Skin and subcutaneous tissue disorders - Other (angioedema)[9]; Skin and subcutaneous tissue disorders - Other (skin lesion); Skin ulceration
VASCULAR DISORDERS - Flushing; Hypertension; Lymphedema; Phlebitis; Thromboembolic event; Vascular disorders - Other (hemorrhage)

Note: Everolimus (RAD-001) in combination with other agents could cause an exacerbation of any adverse event currently known to be caused by the other agent, or the combination may result in events never previously associated with either agent.

Attachment 2: **Revised ICD Section(s) for Everolimus**

Please note that the terminology for CTEP's suggested lay terms may change periodically. The condensed risk profile represents CAEPR risks in a "patient-friendly" condensed form. It is provided as a guide and its use is completely optional; however, all risks listed in the current CAEPR must be reflected in the ICD. Expanding or condensing similar terms is acceptable. If you have chosen to reformat and/or reword the condensed risk profile in any way, please state, "The condensed risk profile has been modified" in the cover memo. Please insert this condensed risk profile as the Table of Possible Side Effects for everolimus in your ICD.

Risk Profile for Everolimus (CAEPR Version 2.2, March 21, 2016)

NOTE: The risk list section in the new NCI Consent Form Template (latest version: May 2013) will include the wording below:

"If you choose to take part in this study, there is a risk that:
- You may lose time at work or home and spend more time in the hospital or doctor's office than usual
- You may be asked sensitive or private questions which you normally do not discuss

The everolimus used in this study may affect how different parts of your body work such as your liver, kidneys, heart, and blood. The study doctor will be testing your blood and will let you know if changes occur that may affect your health.

There is also a risk that you could have side effects from the study drug(s)/study approach.

Here are important points about side effects:
- The study doctors do not know who will or will not have side effects.
- Some side effects may go away soon, some may last a long time, or some may never go away.
- Some side effects may interfere with your ability to have children.
- Some side effects may be serious and may even result in death.

Here are important points about how you and the study doctor can make side effects less of a problem:
- Tell the study doctor if you notice or feel anything different so they can see if you are having a side effect.
- The study doctor may be able to treat some side effects.
- The study doctor may adjust the study drugs to try to reduce side effects.

The tables below show the most common and the most serious side effects that researchers know about. There might be other side effects that researchers do not yet know about. If important new side effects are found, the study doctor will discuss these with you."

Please insert this condensed risk profile as the Table of Possible Side Effects for Everolimus in your ICD.

**COMMON, SOME MAY BE SERIOUS**<br>In 100 people receiving everolimus, more than 20 and up to 100 may have:

Action Letter

- Anemia which may require blood transfusion
- Diarrhea
- Sores in the mouth which may cause difficulty swallowing
- Tiredness
- Bruising, bleeding
- Rash

OCCASIONAL, SOME MAY BE SERIOUS<br>In 100 people receiving everolimus, from 4 to 20 may have:
• Pain<br>• Dry mouth, skin<br>• Nausea, vomiting<br>• Swelling of the arms, legs<br>• Fever<br>• Infection, especially when white blood cell count is low<br>• Weight loss, loss of appetite<br>• Dizziness, headache<br>• Changes in taste<br>• Difficulty sleeping<br>• Cough, shortness of breath<br>• Nose bleed<br>• Swelling of the lungs which may cause shortness of breath<br>• Itching, acne<br>• Change in or loss of some or all of the finger or toenails

RARE, AND SERIOUS<br>In 100 people receiving everolimus, 3 or fewer may have:
• Non-healing surgical site<br>• Kidney damage which may require dialysis

Action Letter

Attachment 3: Action Letter GENERAL INSTRUCTIONS

1. **Distribute this Action Letter (and any accompanying CTEP-approved amendment) to all participating investigators and IRBs within 2 working days.** For National Clinical Trials Network (NCTN) studies, please follow instructions from your Operations office. For sites participating in the NCI Central-IRB (CIRB) Initiative, CTEP has provided a copy of this Action Letter to the CIRB if the Action Letter affects any studies under CIRB review.

2. **Patients currently on study may continue on study provided they are informed of the new and/or modified risk information.** This information should be communicated to patients already enrolled on study without waiting for IRB review/approval since this information represents a significant new finding(s) that developed during the course of the research that may relate to a patient's willingness to continue participation and per the Office for Human Research Protections, the regulations do not require IRB review and approval of statements describing such significant new findings before they are provided to already enrolled patients. Documentation of their informed consent should be carried out according to local IRB requirements.

3. **Save a copy of the Action Letter (and any CTEP approval letter for an accompanying amendment) for your records.**

INSTRUCTIONS FOR PREPARATION OF AN AMENDMENT IN RESPONSE TO THIS Action Letter (if a CTEP-Approved amendment for your trial does not already accompany the Action Letter)

General Instructions on Amendment Preparation:

1. Instructions regarding the due date for an amendment and where to send it are included on the first page of this Action Letter. The Clinical Trials Operations Offices may use the *Detailed Description of Required Protocol Changes* section in this Action Letter as the template for their Change Memo; however, it is not required if an Operations Office prefers to use its own Change Memo.
2. If any of the NCI-required changes are not applicable for your trial (i.e., already appear in your protocol), note this in your Change Memo.
3. **The ICD changes include CTEP's suggested lay terms for each adverse event identified in the CAEPR. You may substitute different lay terms for each concept if appropriate for your patient population. If you have chosen to reformat and/or reword the risk profile in any way, please state, "The risk profile has been modified" in the cover memo.**

Specific Instructions on Amendment Preparation Based on Protocol Status:

A. Trials with a current CTEP status of **"Active"**
- Review and follow **ALL** the instructions outlined in this Action Letter.
- The information provided in this memo outlines the **ONLY** changes that can be made in response to the Action Letter, except for changes that are consistent with CTEP's editorial and administrative update policy (http://ctep.cancer.gov/protocolDevelopment/docs/requestsubmissionpolicyfinal.pdf).

B. Trials with a current status of **"Approved", "Temporarily Closed to Accrual and Treatment", or "Temporarily Closed to Accrual"**
- The protocol and ICD must be revised as per the instructions outlined in the Action Letter in the next revised protocol draft submitted to CTEP. The protocol amendment must be submitted and approved by CTEP before the trial can be activated or re-opened.
- You may include additional non-Action Letter related changes (any type) in your amendment response.

Action Letter

C. Trials with a current CTEP status of **"In Review"**

- The protocol and ICD must be revised as per the instructions outlined in the Action Letter. These changes must be included in the next revised protocol draft submitted to and approved by CTEP before the trial can be activated.
- You may include additional non-Action Letter related changes (any type) in your revision response. Note the inclusion of non-Action Letter related changes may delay approval of your trial.

D. Trials with a current CTEP status of **"Closed to Accrual"**

If your trial is under a CTEP-held IND:

- Review and follow **ALL** the instructions outlined in this Action Letter.
- The information provided in this memo outlines the **ONLY** changes that can be made in response to the Action Letter, except for changes that are consistent with CTEP's amendment request submission policy (http://ctep.cancer.gov/protocolDevelopment/amendments.htm).

If your trial is NOT under a CTEP-held IND:

- If Action Letter INCLUDES information that impacts patient care (e.g., new/adjusted dose modifications or special monitoring for patient population at risk) - An amendment is required. Review and follow **ALL** the instructions outlined in this Action Letter. The information provided in this memo outlines the **ONLY** changes that can be made in response to the Action Letter, except for changes that are consistent with CTEP's amendment request submission policy (http://ctep.cancer.gov/protocolDevelopment/amendments.htm).
    - **If Action Letter does NOT INCLUDE information that impacts patient care - Amendment is typically NOT required.**

E. Trials with a current CTEP status of **"Closed to Accrual and Treatment"** or **"Complete"**

- Amendment is not required. This information is being sent for informational purposes only. You may follow local IRB or Operations Office procedures and requirements.

SWOG
Leading cancer research. Together.

GROUP CHAIR'S OFFICE
Charles D. Blanke, MD
CHAIR
3181 SW Sam Jackson Pk Rd
MC: L586
Portland, OR 97239
503-494-5586
503-346-8038 FAX

OPERATIONS OFFICE
4201 Medical Dr
Suite 250
San Antonio, TX 78229
210-614-8808
210-614-0006 FAX

STATISTICAL CENTER
1730 Minor Ave
Suite 1900
Seattle, WA 98101
206-652-2267
206-342-1616 FAX

1100 Fairview Ave North
M3-C102
PO Box 19024
Seattle, WA 98109
206-667-4623
206-667-4408 FAX

swog.org

Distribution Date: May 15, 2016
Submission Date: February 11, 2016

TO: ALL NATIONAL CLINICAL TRIALS NETWORK (NCTN) MEMBERS – U.S. INSTITUTIONS ONLY

FROM: SWOG Operations Office (E-mail: protocols@swog.org)

RE: **S0931**, "EVEREST: EVErolimus for Renal Cancer Ensuing Surgical Therapy, A Phase III Study. Study Chairs: Drs. C.W. Ryan, E.I. Heath, P.N. Lara, G.S. Palapattu, and P.C. Mack.

REVISION #6

Study Chair: Christopher W. Ryan, M.D.
Phone number: 503/494-8487
E-mail: ryanc@ohsu.edu

IRB Review Requirements

( ) Full board review required. Reason:
- ( ) Initial activation (should your institution choose to participate)
- ( ) Increased risk to patient
- ( ) Complete study redesign
- ( ) Addition of tissue banking requirements
- ( ) Study closure due to new risk information

(√) Expedited review allowed

( ) No review required

REVISION #6

The above noted protocol document has been revised as follows:

The changes in Section 3.1c and the risk section of the Model Consent Form noted below are in response to a Request for Amendment (RA) from Dr. L. Austin Doyle (doylela@mail.nih.gov) received on December 16, 2015. There is not an Action Letter associated with this RA.

1. Title Page, Page 1: The Version Date of the protocol and model consent form has been updated. The NCT number has been revised from NCT #01120145 to NCT #01120249. Dr. Primo Lara and Dr. Mark Stein's contact information has been updated.

2. Section 3.1c, Pages 12-15: CTEP now requires all protocols involving everolimus to include a Comprehensive Adverse Events and Potential Risks (CAEPR) list. Therefore, the adverse event (AE) table has been replaced with a CAEPR Version 2.1 (July 29, 2015). Information has been displaced through Page 15 and subsequent sections/pages have been renumbered.

SWOG Authorized Document

3. Section 5.0, Page 20: "guquestion@crab.org" has been added to this section as an alternate contact method to the Data Operations Center for eligibility issues.

4. Section 7.4, Page 26: This section has been revised to provide clarification to sites who use the CIRB (vs. sites who do not) that they must utilize the Intake Calendar.

5. Section 15.2e.1, Page 56: The phone number for the Data Operations Center has been revised.

Institutions should update their local consent forms to include the changes to the Model Consent Form. SWOG considers that the Model Consent Form changes **do not** represent an alteration in risk/benefit ratio. Therefore, local accrual does **not** need to be suspended pending implementation of these changes. Patients currently on treatment and patients who sign a consent form prior to local implementation of the consent form changes **must** be informed of the following changes in bold. The manner by which this notification takes place is at the discretion of the local institution. At a minimum, the patient must be notified by the next visit and this notification process must be documented in the patient chart.

6. Model Consent Form, Page 4, What will happen if I take part in this research study?: Week "20" has been revised to "19" in the 2nd, 3rd and 4th bullet points of this section as a cycle is 6 weeks in length.

7. Model Consent Form, Page 6: The following changes have been made to the risk tables for everolimus/placebo:

<u>Added New Risk:</u>
- **<u>Common:</u> bruising**
- **<u>Occasional:</u> constipation; dizziness; shortness of breath**

<u>Increase in Risk Attribution:</u>
- **<u>Changed to Common from Occasional:</u> bleeding**

<u>Decrease in Risk Attribution:</u>
- <u>Changed to Occasional from Common</u>: Nausea; itching; headache; nosebleed; weight loss; loss of appetite; itching; swelling of the hands and/or feet (now referred to as swelling of arms and legs); altered taste (now referred to as changes in taste)

<u>Provided Further Clarification:</u>
- <u>Common:</u> "Mouth or lip sores" has been changed to "Sores in the mouth which may cause difficulty swallowing"; "Lack or loss of strength, fatigue" is now referred to as "Tiredness"
- <u>Occasional:</u> "Difficult or labored breathing" is now referred to as "Damage to the lungs which may cause difficulty breathing"; "dry skin" and "dry mouth" are now in the same section; "-chest, oral, joint, abdominal" has been removed from the pain risk; "which may cause blurred vision" has been added to the high blood pressure risk; "inability to sleep" is now referred to as "difficulty sleeping"; "Nail disorder" is now referred to as "change in or loss of some or all of the fingernails or toenails"; "decrease in white blood cells, which can increase your risk of infection" is now referred to as "Infection, especially when white blood cell count is low."
- <u>Rare:</u> Lengthy wound healing" is now referred to as "non-healing surgical site"; "kidney damage which may cause swelling, may require dialysis" is now referred to as "kidney damage which may require dialysis"

<u>Deleted Risk:</u>
- <u>Common:</u> inflammation of the lungs

SWOG Authorized Document

- Occasional: indigestion or upset stomach; dehydration; flushing, reddening of skin; infection of the lungs (pneumonia); kidney failure; frequent urination; abnormal or loss of menstrual period; diabetes; Hand-foot syndrome: reddening, swelling, numbness, and peeling on palms of the hands and soles of the feet; decrease in blood platelets which can increase your risk of bleeding or bruising
- Rare: Blood clot which may cause swelling, pain, shortness of breath; heart failure which may cause shortness of breath, swelling of ankles and tiredness; coughing or spitting up blood from the lungs or respiratory tract; allergic reaction, which can cause swelling of the face or throat; severe reduction in blood cells, which can cause weakness, bruising or make infections more likely

This memorandum serves to notify the NCI and the SWOG Statistical Center.

cc: PROTOCOL & INFORMATION OFFICE
Cathy M. Tangen, Dr.P.H.
Melissa Plets, M.S.
Jean Barce
Amy Johnson
Diana Heaney
Meredith Lavin - Novartis
Jason Koury, Pharm.D. - VACSP
Sharon Jenkins - VACSP

SWOG Authorized Document

SWOG
Leading cancer research. Together.

GROUP CHAIR'S OFFICE
Charles D. Blanke, MD
CHAIR
3181 SW Sam Jackson Pk Rd
MC: L586
Portland, OR 97239
503-494-5586
503-346-8038 FAX

OPERATIONS OFFICE
4201 Medical Dr
Suite 250
San Antonio, TX 78229
210-614-8808
210-614-0006 FAX

STATISTICAL CENTER
1730 Minor Ave
Suite 1900
Seattle, WA 98101
206-652-2267
206-342-1616 FAX

1100 Fairview Ave North
M3-C102
PO Box 19024
Seattle, WA 98109
206-667-4623
206-667-4408 FAX

swog.org

February 15, 2016

TO: ALL NATIONAL CLINICAL TRIALS NETWORK (NCTN) MEMBERS

FROM: SWOG Operations Office

RE: IND Safety Reports for Everolimus

MEMORANDUM

IRB Review Requirements

( ) Full board review required. Reason:
- ( ) Initial activation (should your institution choose to participate)
- ( ) Increased risk to patient
- ( ) Complete study redesign
- ( ) Addition of tissue banking requirements
- ( ) Study closure due to new risk information

(√) Expedited review allowed

( ) No review required

MEMORANDUM

The following new safety reports have been posted regarding adverse events that occurred in association with the drug everolimus. Please access these safety reports via the safety report link in the member section of the SWOG website (https://swog.org/SafetyReports/SafetyReports.asp).

These safety reports pertain to the following studies:	Reports:
**S0931** Genitourinary	Nov. 12, 2015 Mfr Rpt #PHHO2015AR018401 FU
**S1207** Breast	Nov. 23, 2015 Mfr Rpt #PHHO2015DE014592
**S1222** Breast	Nov. 26, 2015 Mfr Rpt #PHHO2014US015000
	Dec. 9, 2015 Mfr Rpt #PHH02014FR016631 FU
	Dec. 21, 2015 Mfr Rpt #PHHO2015ES020063

Protocol amendments are not necessary at this time, but your consent form may be revised to include the information provided in these reports if it is deemed necessary by your institution. Please append this notice and these reports to your copy of the protocol and forward copies to your Institutional Review Board (IRB) as required by your local policies and procedures. Should any further information regarding these adverse events be made available, it will be forwarded to you.

This memorandum serves to notify the NCI and the SWOG Statistical Center.

cc: PROTOCOL & INFORMATION OFFICE
Cathy M. Tangen, Dr.P.H.
William Barlow, Ph.D.
Danika Lew, M.S.
Melissa Plets, M.S.
Jean Barce
Jennie Barrett
Austin Hamm
Diana Heaney
Amy Johnson
Larry Kaye
Iris Syquia

SWOG Authorized Document

SWOG
Leading cancer research. Together.

GROUP CHAIR'S OFFICE
Charles D. Blanke, MD
CHAIR
3181 SW Sam Jackson Pk Rd
MC: L586
Portland, OR 97239
503-494-5586
503-346-8038 FAX

OPERATIONS OFFICE
4201 Medical Dr
Suite 250
San Antonio, TX 78229
210-614-8808
210-614-0006 FAX

STATISTICAL CENTER
1730 Minor Ave
Suite 1900
Seattle, WA 98101
206-652-2267
206-342-1616 FAX

1100 Fairview Ave North
M3-C102
PO Box 19024
Seattle, WA 98109
206-667-4623
206-667-4408 FAX

swog.org

November 15, 2015

TO: ALL NATIONAL CLINICAL TRIALS NETWORK (NCTN) MEMBERS

FROM: SWOG Operations Office

RE: IND Safety Reports for Everolimus

MEMORANDUM

IRB Review Requirements

( ) Full board review required. Reason:
- ( ) Initial activation (should your institution choose to participate)
- ( ) Increased risk to patient
- ( ) Complete study redesign
- ( ) Addition of tissue banking requirements
- ( ) Study closure due to new risk information

(√) Expedited review allowed

( ) No review required

MEMORANDUM

The following new safety reports have been posted regarding adverse events that occurred in association with the drug everolimus. Please access these safety reports via the safety report link in the member section of the SWOG website (https://swog.org/SafetyReports/SafetyReports.asp).

These safety reports pertain to the following studies:	Reports:
**S0931** Genitourinary	Aug. 5, 2015 Mfr Rpt # PHHO2015DE011077
**S1207** Breast	Aug. 14, 2015 Mfr Rpt # PHHO2015DE12647
**S1222** Breast	Aug. 28, 2015 Mfr Rpt # PHJP2015JP013606
	Oct. 9, 2015 Mfr Rpt # PHHO2015US014557
	Nov. 2, 2015 Mfr Rpt # PHHO2015AR018401

Protocol amendments are not necessary at this time, but your consent form may be revised to include the information provided in these reports if it is deemed necessary by your institution. Please append this notice and these reports to your copy of the protocol and forward copies to your Institutional Review Board (IRB) as required by your local policies and procedures. Should any further information regarding these adverse events be made available, it will be forwarded to you.

This memorandum serves to notify the NCI and the SWOG Statistical Center.

cc: PROTOCOL & INFORMATION OFFICE
Cathy M. Tangen, Dr.P.H.
William Barlow, Ph.D.
Danika Lew, M.S.
Melissa Plets, M.S.
Jean Barce
Jennie Barrett
Austin Hamm
Diana Heaney
Amy Johnson
Larry Kaye
Iris Syquia

SWOG Authorized Document

SWOG
Leading cancer research. Together.

GROUP CHAIR'S OFFICE
Charles D. Blanke, MD
CHAIR
3181 SW Sam Jackson Pk Rd
MC: L586
Portland, OR 97239
503-494-5586
503-346-8038 FAX

OPERATIONS OFFICE
4201 Medical Dr
Suite 250
San Antonio, TX 78229
210-614-8808
210-614-0006 FAX

STATISTICAL CENTER
1730 Minor Ave
Suite 1900
Seattle, WA 98101
206-652-2267
206-342-1616 FAX

1100 Fairview Ave North
M3-C102
PO Box 19024
Seattle, WA 98109
206-667-4623
206-667-4408 FAX

swog.org

September 15, 2015

TO: ALL NATIONAL CLINICAL TRIALS NETWORK (NCTN) MEMBERS – U.S. INSTITUTIONS ONLY

FROM: Jennifer I. Scott, Protocol Coordinator (E-mail: jscott@swog.org)

RE: **S0931**, "EVEREST: EVErolimus for Renal Cancer Ensuing Surgical Therapy, A Phase III Study. Study Chairs: Drs. C.W. Ryan, E.I. Heath, P.N. Lara, G.S. Palapattu, and P.C. Mack.

STATUS NOTICE

Study Chair: Christopher W. Ryan, M.D.
Phone number: 503/494-8487
E-mail: ryanc@ohsu.edu

IRB Review Requirements

( ) Full board review required. Reason:
- ( ) Initial activation (should your institution choose to participate)
- ( ) Increased risk to patient
- ( ) Complete study redesign
- ( ) Addition of tissue banking requirements
- ( ) Study closure due to new risk information

(√) Expedited review allowed

( ) No review required

RE-ACTIVATION

The purpose of this memorandum is to inform sites that this study has been re-opened to accrual **effective immediately**.

This memorandum serves to notify the NCI and the SWOG Statistical Center.

cc: PROTOCOL & INFORMATION OFFICE
Cathy M. Tangen, Dr.P.H.
Melissa Plets, M.S.
Jean Barce
Diana Heaney
Amy Johnson
Meredith Lavin – Novartis
Jason Koury, Pharm.D. – VACSP
Sharon Jenkins - VACSP

SWOG Authorized Document

SWOG
Leading cancer research. Together.

GROUP CHAIR'S OFFICE
Charles D. Blanke, MD
CHAIR
3181 SW Sam Jackson Pk Rd
MC: L586
Portland, OR 97239
503-494-5586
503-346-8038 FAX

OPERATIONS OFFICE
4201 Medical Dr
Suite 250
San Antonio, TX 78229
210-614-8808
210-614-0006 FAX

STATISTICAL CENTER
1730 Minor Ave
Suite 1900
Seattle, WA 98101
206-652-2267
206-342-1616 FAX

1100 Fairview Ave North
M3-C102
PO Box 19024
Seattle, WA 98109
206-667-4623
206-667-4408 FAX

swog.org

Distribution Date: September 15, 2015
CTEP Submission Date: July 24, 2015

TO: ALL NATIONAL CLINICAL TRIALS NETWORK (NCTN) MEMBERS – U.S. INSTITUTIONS ONLY

FROM: Jennifer I. Scott, Protocol Coordinator (E-mail: jscott@swog.org)

RE: **S0931**, "EVEREST: EVErolimus for Renal Cancer Ensuing Surgical Therapy, A Phase III Study. Study Chairs: Drs. C.W. Ryan, E.I. Heath, P.N. Lara, G.S. Palapattu, and P.C. Mack.

REVISION #5

Study Chair: Christopher W. Ryan, M.D.
Phone number: 503/494-8487
E-mail: ryanc@ohsu.edu

IRB Review Requirements

( ) Full board review required. Reason:
- ( ) Initial activation (should your institution choose to participate)
- ( ) Increased risk to patient
- ( ) Complete study redesign
- ( ) Addition of tissue banking requirements
- ( ) Study closure due to new risk information

(√) Expedited review allowed

( ) No review required

REVISION #5

The above noted protocol document has been revised as follows:

The drop-out rate on **S0931** has been greater than what was originally anticipated when the study was designed. While a 15% non-compliance rate was anticipated, the dropout rate is closer to 28%. The higher-than-anticipated drop-out rate has raised concern that the potential effect of everolimus on recurrence-free survival could be diminished due to suboptimal drug exposure. Thus in order to compensate for early dropouts, the study has been revised is to increase the sample size from 1218 to 1537 patients. This is the primary reason for this revision and changes related to the increased sample size as well as additional protocol and consent modifications are detailed below.

We estimate that this additional accrual will add one year to the study duration. The assumptions and parameters of the design change were specified by study leadership who had not seen any outcome data. The Data and Safety Monitoring Committee (DSMC) has seen the proposed design change with no further comment.

The above-noted protocol has been revised as follows:

1. **Face Page, page 1:** The version date of the protocol has been updated. The email addresses for Dr. Lara and the biostatisticians have been revised.

SWOG Authorized Document

2. **Table of Contents, page 4:** The Table of Contents has been revised to reflect changes made in the protocol.

3. **Section 2.0, page 11:** Based on the increased sample size, the table in Section 2.0 has been revised to estimate accrual in the ethnicity/race and sex categories.

4. **Section 3.1c.3, page 14:** The reference to Appendix 18.4 has been revised to reference Appendix 18.3.

5. **Section 3.1f.3, page 17:** Section a (the drug accountability section) has been revised to remove reference to the **S0931** Drug Accountability Record Form and include reference to the NCI Oral Drug Accountability Record Form (NCI Oral DARF). The **S0931** Drug Accountability Record Form has been removed from the SWOG protocol abstract page, but as now noted in this section, the NCI Oral DARF is available at http://ctep.cancer.gov. Section b has been revised to specify "Oral" in the text "current NCI DARF."

6. **Sections 7.1 and 7.3, pages 24-25:** The references to Appendix items have been updated in these sections.

7. **Section 9.0, page 39:** The "¶" footnote has been revised to be consistent with the changes made to the " Ж" footnote in Revision #4.

8. **Section 11.1, pages 41-42:** The accrual goal for this study has been revised and three paragraphs were added to this section to provide background rationale and explanation regarding the design change.

9. **Section 11.2, pages 42-43:** This section has been revised to indicate that the total projected accrual is 1537 patients rather than the previously planned 1218. The second paragraph on page 42 as well as the table have been revised to adjust the timing of the analyses.

10. **Section 11.3, page 43:** This section has been updated to revise the number of patients required and the confidence interval for analysis of the secondary endpoints.

11. **Section 11.4, pages 43-44:** This section has been revised to replace the text "X" with "by". The percentages have been deleted from the table heading. In the last paragraph, "80% of" has been replaced with "936."

12. **Section 11.5, pages 44-45:** This section has been added to provide plans for pharmacokinetic analysis of blood samples and the remainder of Section 11 has been renumbered accordingly.

13. **Section 16.1a, page 56:** The last sentence of this section has been deleted as Appendix 18.3 has been removed from the protocol.

14. **Section 18.3, page 68:** Appendix 18.3 has been deleted from the protocol as it was intended to provide guidance for both the legacy and new reporting tables and is no longer necessary. The remainder of Section 18.0 has been renumbered accordingly.

SWOG considers that the Model Consent Form changes **do not** represent an alteration in risk/benefit ratio. Therefore, local accrual does **not** need to be suspended pending implementation of these changes. As of 90 days from distribution of this revision, patients must be consented under the 7/24/15 version of the consent form. Patients currently receiving everolimus and patients who sign a consent form prior to local implementation of the consent

form changes **must** be informed of the following changes. The manner by which this notification takes place is at the discretion of the local institution. At a minimum, the patient must be notified by the next visit and this notification process must be documented in the patient chart.

The model consent form has been revised as follows:

1. **Model Informed Consent, page 1:** The version date of the model informed consent has been updated. The first paragraph under "Notes for Local Institution Informed Consent Authors" has been revised to add the word "additions" to the fifth sentence.

2. **Page 3:** The total accrual has been revised in the "How many people will take part in the study" section of the model informed consent.

3. **Page 7:** The text "Inflammation" has been changed to "Infection" in the third to last bullet point under "Occasional, Some May Be Serious" section as this was noted in error in Revision #4.

4. **Page 8:** The text "Patients taking angiotensin converting enzyme (ACE) inhibitor therapy may be at an increased risk for swelling of the airways or tongue, with or without damage to the lungs" has been added to the second paragraph of this page to be consistent with text added to the protocol in Revision #4.

5. **Page 10:** The url for the NCI's web page for more information on clinical trials and insurance coverage has been corrected.

6. **Page 14:** The TTY phone number has been deleted as it is no longer correct.

This memorandum serves to notify the NCI and the SWOG Statistical Center.

cc: PROTOCOL & INFORMATION OFFICE
Cathy M. Tangen, Dr.P.H.
Melissa Plets, M.S.
Jean Barce
Amy Johnson
Diana Heaney
Meredith Lavin - Novartis
Jason Koury, Pharm.D. - VACSP
Sharon Jenkins - VACSP

SWOG Authorized Document

SWOG
Leading cancer research. Together.

GROUP CHAIR'S OFFICE
Charles D. Blanke, MD
CHAIR
3181 SW Sam Jackson Pk Rd
MC: L586
Portland, OR 97239
503-494-5586
503-346-8038 FAX

OPERATIONS OFFICE
4201 Medical Dr
Suite 250
San Antonio, TX 78229
210-614-8808
210-614-0006 FAX

STATISTICAL CENTER
1730 Minor Ave
Suite 1900
Seattle, WA 98101
206-652-2267
206-342-1616 FAX

1100 Fairview Ave North
M3-C102
PO Box 19024
Seattle, WA 98109
206-667-4623
206-667-4408 FAX

swog.org

August 1, 2015

TO: ALL NATIONAL CLINICAL TRIALS NETWORK (NCTN) MEMBERS – U.S. INSTITUTIONS ONLY

FROM: Jennifer I. Scott, Protocol Coordinator (E-mail: jscott@swog.org)

RE: **S0931**, "EVEREST: EVErolimus for Renal Cancer Ensuing Surgical Therapy, A Phase III Study. Study Chairs: Drs. C.W. Ryan, E.I. Heath, P.N. Lara, G.S. Palapattu, and P.C. Mack.

STATUS NOTICE

Study Chair: Christopher W. Ryan, M.D.
Phone number: 503/494-8487
E-mail: ryanc@ohsu.edu

IRB Review Requirements

( ) Full board review required. Reason:
- ( ) Initial activation (should your institution choose to participate)
- ( ) Increased risk to patient
- ( ) Complete study redesign
- ( ) Addition of tissue banking requirements
- ( ) Study closure due to new risk information

(√) Expedited review allowed

( ) No review required

TEMPORARY CLOSURE

The above-referenced study is temporarily closed **effective August 15, 2015 at 11:00 p.m. Eastern Time** as it has met its current accrual goal. The study is currently being revised to increase the sample size and the trial will be reopened once the design change has been approved.

This memorandum serves to notify the NCI and the SWOG Statistical Center.

cc: PROTOCOL & INFORMATION OFFICE
Cathy M. Tangen, Dr.P.H.
Melissa Plets, M.S.
Jean Barce
Amy Johnson
Meredith Lavin – Novartis
Jason Koury, Pharm.D. – VACSP
Sharon Jenkins - VACSP

SWOG Authorized Document

SWOG
Leading cancer research. Together.

GROUP CHAIR'S OFFICE
Charles D. Blanke, MD
CHAIR
3181 SW Sam Jackson Pk Rd
MC: L586
Portland, OR 97239
503-494-5586
503-346-8038 FAX

OPERATIONS OFFICE
4201 Medical Dr
Suite 250
San Antonio, TX 78229
210-614-8808
210-614-0006 FAX

STATISTICAL CENTER
1730 Minor Ave
Suite 1900
Seattle, WA 98101
206-652-2267
206-342-1616 FAX

1100 Fairview Ave North
M3-C102
PO Box 19024
Seattle, WA 98109
206-667-4623
206-667-4408 FAX

swog.org

August 1, 2015

TO: ALL NATIONAL CLINICAL TRIALS NETWORK (NCTN) MEMBERS

FROM: SWOG Operations Office

RE: IND Safety Reports for Everolimus

MEMORANDUM

IRB Review Requirements

( ) Full board review required. Reason:
- ( ) Initial activation (should your institution choose to participate)
- ( ) Increased risk to patient
- ( ) Complete study redesign
- ( ) Addition of tissue banking requirements
- ( ) Study closure due to new risk information

(√) Expedited review allowed

( ) No review required

MEMORANDUM

The following new safety reports have been posted regarding adverse events that occurred in association with the drug everolimus. Please access these safety reports via the safety report link in the member section of the SWOG website (https://swog.org/SafetyReports/SafetyReports.asp).

These safety reports pertain to the following studies:	Reports:
**S0931** Genitourinary	May 13, 2015 Mfr Rpt #PHHO2015IT008256
**S1207** Breast	May 13, 2015 Mfr Rpt #PHHO2015IT008257
**S1222** Breast	May 13, 2015 Mfr Rpt #PHHO2015IT008272
	May 26, 2015 Mfr Rpt #PHHO2015US009217
	May 27, 2015 Mfr Rpt #PHHO2015US009213
	June 16, 2015 Mfr Rpt #PHHO2014US008515
	June 16, 2015 Mfr Rpt #PHHO2013US012889
	June 19, 2015 Mfr Rpt #PHHO2015US006784 FU
	July 2, 2015 Mfr Rpt #PHHO2015FR010015
	July 6, 2015 Mfr Rpt #PHHO2015FR010935
	July 8, 2015 Mfr Rpt #PHHO2013BE001444
	July 8, 2015 Mfr Rpt #PHHO2015SK002702
	July 13, 2015 Mfr Rpt #PHHO2015DE009757

Protocol amendments are not necessary at this time, but your consent form may be revised to include the information provided in these reports if it is deemed necessary by your institution. Please append this notice and these reports to your copy of the protocol and forward copies to your Institutional Review Board (IRB) as required by your local policies and procedures. Should any further information regarding these adverse events be made available, it will be forwarded to you.

SWOG Authorized Document

This memorandum serves to notify the NCI and the SWOG Statistical Center.

cc: PROTOCOL & INFORMATION OFFICE	Jennie Barrett
Cathy M. Tangen, Dr.P.H.	Austin Hamm
William Barlow, Ph.D.	Diana Heaney
Danika Lew, M.S.	Amy Johnson
Melissa Plets, M.S.	Larry Kaye
Jean Barce	Iris Syquia

SWOG
Leading cancer research. Together.

GROUP CHAIR'S OFFICE
Charles D. Blanke, MD
CHAIR
3181 SW Sam Jackson Pk Rd
MC: L586
Portland, OR 97239
503-494-5586
503-346-8038 FAX

OPERATIONS OFFICE
4201 Medical Dr
Suite 250
San Antonio, TX 78229
210-614-8808
210-614-0006 FAX

STATISTICAL CENTER
1730 Minor Ave
Suite 1900
Seattle, WA 98101
206-652-2267
206-342-1616 FAX

1100 Fairview Ave North
M3-C102
PO Box 19024
Seattle, WA 98109
206-667-4623
206-667-4408 FAX

swog.org

July 1, 2015

TO: ALL NATIONAL CLINICAL TRIALS NETWORK (NCTN) MEMBERS

FROM: SWOG Operations Office

RE: IND Safety Report for Everolimus

MEMORANDUM

IRB Review Requirements

( ) Full board review required. Reason:
- ( ) Initial activation (should your institution choose to participate)
- ( ) Increased risk to patient
- ( ) Complete study redesign
- ( ) Addition of tissue banking requirements
- ( ) Study closure due to new risk information

(√) Expedited review allowed

( ) No review required

MEMORANDUM

The following safety report was inadvertently posted for the drug everolimus. In actuality, the event that occurred was in association with the drug LEE011 and should be disregarded. The safety report has been removed from the SWOG website (https://swog.org/safetyreports/safetyreports.asp).

This safety report pertains to the following studies:	Report:
**S0528** Early Therapeutics<br>**S0931** Genitourinary<br>**S1207** Breast<br>**S1222** Breast	Apr. 29, 2015 Mfr Rpt #PHHO2015US006628

Please append this notice and this report to your copy of the protocol and forward a copy to your Institutional Review Board (IRB) as required by your local policies and procedures.

This memorandum serves to notify the NCI and the SWOG Statistical Center.

cc: PROTOCOL & INFORMATION OFFICE
Megan Othus, Ph.D.
Cathy M. Tangen, Dr.P.H.
William Barlow, Ph.D.
Danika Lew, M.S.
Melissa Plets, M.S.
Jean Barce
Jennie Barrett
Austin Hamm
Louise Highleyman
Amy Johnson
Larry Kaye
Laura Kingsbury, M.R.T.
Christine McLeod
Iris Syquia

SWOG Authorized Document

SWOG
Leading cancer research. Together.

GROUP CHAIR'S OFFICE
Charles D. Blanke, MD
CHAIR
3181 SW Sam Jackson Pk Rd
MC: L586
Portland, OR 97239
503-494-5586
503-346-8038 FAX

OPERATIONS OFFICE
4201 Medical Dr
Suite 250
San Antonio, TX 78229
210-614-8808
210-614-0006 FAX

STATISTICAL CENTER
1730 Minor Ave
Suite 1900
Seattle, WA 98101
206-652-2267
206-342-1616 FAX

1100 Fairview Ave North
M3-C102
PO Box 19024
Seattle, WA 98109
206-667-4623
206-667-4408 FAX

swog.org

May 15, 2015

TO: ALL NATIONAL CLINICAL TRIALS NETWORK (NCTN) MEMBERS

FROM: SWOG Operations Office

RE: IND Safety Reports for Everolimus

MEMORANDUM

IRB Review Requirements

( ) Full board review required. Reason:
- ( ) Initial activation (should your institution choose to participate)
- ( ) Increased risk to patient
- ( ) Complete study redesign
- ( ) Addition of tissue banking requirements
- ( ) Study closure due to new risk information

(√) Expedited review allowed

( ) No review required

MEMORANDUM

The following new safety reports have been posted regarding adverse events that occurred in association with the drug everolimus. Please access these safety reports via the study's abstract page or the safety report link on the SWOG website (https://swog.org/safetyreports/safetyreports.asp).

These safety reports pertain to the following studies:

S0528 Early Therapeutics
S0931 Genitourinary
S1207 Breast
S1222 Breast

Reports:

Mar. 5, 2015	Mfr Rpt #PHHO2015FR003167
Mar. 5, 2015	Mfr Rpt #PHHO2010FR14815
Apr. 8, 2015	Mfr Rpt #PHHO2015DE003225
Apr. 16, 2015	Mfr Rpt #PHHY2013BR124787
Apr. 20, 2015	Mfr Rpt #PHHO2015BR006547
Apr. 21, 2015	Mfr Rpt #PHHO2015BR006547 FU
Apr. 21, 2015	Mfr Rpt #PHHO2013BR017080
Apr. 21, 2015	Mfr Rpt #PHHO2012BR009857
Apr. 24, 2015	Mfr Rpt #PHHO2013BR017080 FU
Apr. 29, 2015	Mfr Rpt #PHHO2015US006628
Apr. 29, 2015	Mfr Rpt #PHHO2015US007003
Apr. 30, 2015	Mfr Rpt #PHHO2015US006784
Apr. 30, 2015	Mfr Rpt #PHHO2015US007456
Apr. 30, 2015	Mfr Rpt #PHHO2015US007463
May 5, 2015	Mfr Rpt #PHHY2015MX052874
May 6, 2015	Mfr Rpt #PHHO2015DE003225 FU
May 6, 2015	Mfr Rpt #PHHO2015FR007310

Protocol amendments are not necessary at this time, but your consent form may be revised to include the information provided in these reports if it is deemed necessary by your institution. Please append this notice and these reports to your copy of the protocol and forward copies to your Institutional Review Board (IRB) as required by your local

SWOG Authorized Document

policies and procedures. Should any further information regarding these adverse events be made available, it will be forwarded to you.

This memorandum serves to notify the NCI and the SWOG Statistical Center.

cc: PROTOCOL & INFORMATION OFFICE
Megan Othus, Ph.D.
Cathy M. Tangen, Dr.P.H.
William Barlow, Ph.D.
Danika Lew, M.S.
Melissa Plets, M.S.
Jean Barce
Jennie Barrett
Austin Hamm
Louise Highleyman
Amy Johnson
Larry Kaye
Laura Kingsbury, M.R.T.
Christine McLeod
Iris Syquia
Carol French – AstraZeneca
William Turnbull – AstraZeneca
Jean Sowa – AstraZeneca
Kathleen DeRose – GSK
Royce Ann Adkins – Path-tec
Meredith Lavin – Novartis
Kristin White – Novartis
Heather Campbell, PharmD – VACSP/PCC
Anne Davis – VACSP/PCC
Michael Moody – VACSP/PCC
Sharon Jenkins – VACSP/PCC
James Rasp – VACSP/PCC
Emily Tabinski – VACSP/PCC
Katie VonDerau – WPC

SWOG Authorized Document

SWOG
Leading cancer research. Together.

GROUP CHAIR'S OFFICE
Charles D. Blanke, MD
CHAIR
3181 SW Sam Jackson Pk Rd
MC: L586
Portland, OR 97239
503-494-5586
503-346-8038 FAX

OPERATIONS OFFICE
4201 Medical Dr
Suite 250
San Antonio, TX 78229
210-614-8808
210-614-0006 FAX

STATISTICAL CENTER
1730 Minor Ave
Suite 1900
Seattle, WA 98101
206-652-2267
206-342-1616 FAX

1100 Fairview Ave North
M3-C102
PO Box 19024
Seattle, WA 98109
206-667-4623
206-667-4408 FAX

swog.org

Distribution Date: April 15, 2015
CTEP Submission Date: December 11, 2014

TO: ALL NATIONAL CLINICAL TRIALS NETWORK (NCTN) MEMBERS – U.S. INSTITUTIONS ONLY

FROM: Jennifer I. Scott, Protocol Coordinator (E-mail: jscott@swog.org)

RE: **S0931**, "EVEREST: EVErolimus for Renal Cancer Ensuing Surgical Therapy, A Phase III Study. Study Chairs: Drs. C.W. Ryan, E.I. Heath, P.N. Lara, G.S. Palapattu, and P.C. Mack.

REVISION #4

Study Chair: Christopher W. Ryan, M.D.
Phone number: 503/494-8487
E-mail: ryanc@ohsu.edu

IRB Review Requirements

( ) Full board review required. Reason:
- ( ) Initial activation (should your institution choose to participate)
- ( ) Increased risk to patient
- ( ) Complete study redesign
- ( ) Addition of tissue banking requirements
- ( ) Study closure due to new risk information

(√) Expedited review allowed

( ) No review required

REVISION #4

The above noted protocol document has been revised as follows:

1. **Page 1 (Title page)**: The following changes have been made to this page:
   - The version date has been updated.
   - Biostatisticians: The contact information for Melissa Plets, M.S. has replaced Hongli Li, M.S.
   - Formatting changes to the Alliance and ECOG-ACRIN Study Chair information has been made, but otherwise the content remains unchanged.

2. **Pages 3-4, Table of Contents**: The pages numbers in the table of contents have been updated.

3. **Pages 12-14, Section 3.1c.1, Everolimus Drug Information**: Due to recent updates with the everolimus investigator brochure (edition 13), the following changes have been made to the adverse effects table:

SWOG Authorized Document

Adverse Events with Possible Relationship to Everolimus		
**Likely (>20%)**	**Less Likely (≤20%)**	**Rare but Serious (<3%)**
BLOOD AND LYMPHATIC SYSTEM DISORDERS		
Anemia	Leukopenia	Pancytopenia
~~Thrombocytopenia~~	Lymphopenia	Pure red cell aplasia
	Neutropenia	
	Thrombocytopenia	
CARDIAC DISORDERS		
		Congestive cardiac failure
GASTROINTESTINAL DISORDERS		
Stomatitis	Abdominal pain	
Diarrhea	Dry mouth	
Nausea	Dyspepsia	
~~Vomiting~~	Dysphagia	
	Oral pain	
	Vomiting	
GENERAL DISORDERS AND ADMINISTRATION SITE CONDITIONS		
Asthenia	Mucosal inflammation	Impaired wound healing
Fatigue	Pyrexia	
Peripheral edema	Non-cardiac chest pain	
IMMUNE SYSTEM DISORDERS		
		Hypersensitivity
INFECTIONS AND INFESTATIONS		
Infections	***Pneumonia***	
INVESTIGATIONS		
Weight decreased	ALT increased	
	AST increased	
	Creatinine increased	
METABOLISM AND NUTRITION DISORDERS		
Decreased appetite	Dehydration	
Hypercholesterolemia	Diabetes mellitus	
Hyperglycemia	Hypokalemia	
	Hyperlipidemia	
	Hypophosphatemia	
	Hypertriglyceridemia	
MUSCULOSKELETAL AND CONNECTIVE TISSURE DISORDERS		
	Arthralgia	
NERVOUS SYSTEM DISORDERS		
Dysgeusia	Ageusia	
Headache		
PSYCHIATRIC DISORDERS		
	Insomnia	
RENAL AND URINARY DISORDERS		
	Renal failure	Acute renal failure
	Increased daytime urination	~~Proteinuria~~
	Proteinuria	
REPRODUCTIVE SYSTEM AND BREAST DISORDERS		
	Menstruation irregular	
	Amenorrhea	
RESPIRATORY, THORACIC AND MEDIASTINAL DISORDERS		
~~Cough~~	***Cough***	Acute respiratory distress syndrome
~~Dyspnea~~	***Dyspnea***	Hemoptysis
Epistaxis		Pulmonary embolism
Pneumonitis		

SWOG Authorized Document

SKIN AND SUBCUTANEOUS TISSUE DISORDERS		
Rash	Acne	***Angioedema***
Pruritus	Dry skin	
	Erythema	
	Hand-foot syndrome	
	Nail disorder	
VASCULAR DISORDERS		
	Hypertension	Deep vein thrombosis
	Hemorrhage	

4. **Page 14, Section 3.1c.1, Everolimus Drug Information**: The following additional information has been added to the everolimus adverse effects section regarding cases of pneumocystis jirovecii pneumonia (PJP):

   "*Cases of pneumocystis jirovecii pneumonia (PJP), some with a fatal outcome, have been reported in patients who received everolimus. PJP may be associated with concomitant use of corticosteroids or other immunosuppressive agents. Prophylaxis for PJP should be considered when concomitant use of corticosteroids or other immunosuppressive agents are required."*

5. **Page 14, Section 3.1c.3, Everolimus Drug Information**: The following additional information has been added to the everolimus drug interactions section regarding concomitant ACE inhibitor therapy:

   "*Patients taking concomitant angiotensin-converting enzyme (ACE) inhibitor therapy may be at increased risk for angioedema (e.g. swelling of the airways or tongue, with or without respiratory impairment).*"

6. **Page 14, Section 3.1c.4, Everolimus Drug Information**: The second sentence regarding hepatic impairment instructions has been updated as follows:

   Original: "Refer to the Section 8.0 Dosage Modification."

   New: "*Contact the Study Chair for guidance*."

   (Due to the changes noted in items #3-5, information has been displaced to page 15 and protocol repagination was required.)

7. **Page 26, Section 7.5a, Criteria for Planned Unblinding**: In the first paragraph, third sentence, the contact information for the GU Data Coordinator has been updated as follows:

   <u>Original</u>
   "If any questions arise with regard to recurrence for a patient, please contact the GU Data Coordinator in Seattle by telephone at 206/652-2267, 6:30 a.m. to 4:00 p.m. Pacific Time, Monday through Friday, excluding holidays."

   <u>New</u>
   "If any questions arise with regard to recurrence for a patient, please contact the GU Data Coordinator in Seattle *by email at guquestion@crab.org or* by telephone at 206/652-2267, 6:30 a.m. to 4:00 p.m. Pacific Time, Monday through Friday, excluding holidays."

SWOG Authorized Document

8. **Page 27, Section 7.6b&c, Criteria for Removal from Protocol Treatment**: Instructions regarding criteria for removal from protocol treatment have been updated as follows:

Section 7.6b
Original: "Unacceptable toxicity defined as any toxicity requiring discontinuation of study drug per Section 8.0."

New: "Unacceptable toxicity defined as any toxicity requiring discontinuation of study drug per Section 8.0. *If a patient removed from protocol treatment due to toxicity, they must continue to be followed for recurrence according to the same schedule as detailed in sections 7.2 and 9.0 (Every 4 months [18 weeks] during Year 1 (regardless whether patient is still on active protocol treatment), then every six months for Years 2 and 3, and then annually thereafter until recurrence, death, or 10 years after registration, whichever comes first. Recurrence assessments consist of physical exams and CT scans).*"

Section 7.6c
Original: "Delay in protocol treatment > 21 days due to pneumonitis or > 28 days due to any other reason. If patient removed from protocol treatment due to toxicity, they must continue to be followed for recurrence."

New: "Delay in protocol treatment > 21 days due to pneumonitis or > 28 days due to any other reason. If patient removed from protocol treatment due to toxicity, they must continue to be followed for recurrence *according to the same schedule as detailed in Sections 7.2 and 9.0 (Every 4 months [18 weeks] during Year 1 (regardless whether patient is still on active protocol treatment), then every six months for two years, and then annually thereafter until recurrence, death, or 10 years after registration, whichever comes first. Recurrence assessments consist of physical exams and CT scans)."*

9. **Page 33, Section 8.5, Infection Precaution**: Updated instructions regarding infection precautions have been updated as follows:

Original:
"Everolimus has immunosuppressive properties and may predispose patients to infections, especially those with opportunistic pathogens. Localized and systemic infections, including pneumonia, other bacterial infections and invasive fungal infections, such as aspergillosis or candidiasis, have been described in patients taking everolimus. Some of these infections have been severe (e.g. leading to respiratory failure) and occasionally have had a fatal outcome. Physicians and patients should be aware of the increased risk of infection with everolimus, be vigilant for symptoms and signs of infection, and institute appropriate treatment promptly."

New
"Everolimus has immunosuppressive properties and may predispose patients to infections, especially those with opportunistic pathogens. Localized and systemic infections, including pneumonia, other bacterial infections and invasive fungal infections, such as aspergillosis, candidiasis, *or pneumocystis jirovecii pneumonia (PJP) and viral infections including reactivation of hepatitis B virus*, have been described in patients taking everolimus. Some of these infections have been severe (e.g. *leading to sepsis;* respiratory *or hepatic failure*) and occasionally have had a fatal outcome. Physicians and patients should be aware of the increased risk of infection with everolimus, be vigilant for symptoms and signs of infection, and institute appropriate treatment promptly."

SWOG Authorized Document

10. **Page 37 & 39, Section 9.0, Study Calendar**: The following changes have been made to these pages:

- Calendar: Toxicity notation is no longer required during off treatment follow-up prior to progression and off treatment follow-up after progression. Also, recurrence assessments are required at the time of recurrence (per the CT scan schedule). An "X" has been placed under the recurrence column.
- Җ footnote: This footnote has been updated as follows:

  Original
  "Scan (CT scan of the chest, abdomen and pelvis with IV and oral contrast; CT scan of chest without contrast and MRI of abd/pelvis with gadolinium) and recurrence assessment must be performed within the week prior to beginning Cycle 4 and Cycle 7 and again at the end of protocol treatment (i.e. after Cycle 9) and then every 6 months for two years, and yearly thereafter until recurrence, death or 10 years after registration (whichever comes first).

  New
  "Scan (CT scan of the chest, abdomen and pelvis with IV and oral contrast; CT scan of chest without contrast and MRI of abd/pelvis with gadolinium) and recurrence assessment must be performed *every 4 months (18 weeks) during Year 1 (regardless of early protocol treatment discontinuation), every 6 months for Years 2 and 3,* ~~within the week prior to beginning Cycle 4 and Cycle 7 and again at the end of protocol treatment (i.e. after Cycle 9) and then every 6 months for two years,~~ and yearly thereafter until recurrence, death or 10 years after registration (whichever comes first)."

11. **Pages 44, Section 13.2, Investigator/Site Requirements**: In the second paragraph, the last sentence has been revised as follows:

Original
"These forms are available on the CTSU Web site (enter credentials at https://www.ctsu.org; then click on the Register tab) or by calling the PMB at 301/496-5725 Monday through Friday between 8:30 a.m. and 4:30 p.m. Eastern time."

New
"These forms are available on the CTSU Web site ~~(enter credentials at https://www.ctsu.org; then click on the Register tab) or by calling the PMB at 301/496-5725 Monday through Friday between 8:30 a.m. and 4:30 p.m. Eastern time.~~ (http://ctep.cancer.gov/investigatorResources/investigator_registration.htm). Questions should be directed to the CTEP Investigator Registration Help Desk by e-mail at pmbregpend@ctep.nci.nih.gov."

12. **Page 45, Section 13.2, Investigator/Site Requirements**: Under 'requirements for site registration', the following note has been added:

"Note: Sites participating on the NCI CIRB initiative and accepting CIRB approval for the study are not required to submit separate IRB approval documentation to the CTSU Regulatory Office for initial, continuing or amendment review. This information will be provided to the CTSU Regulatory Office from the CIRB at the time the site's Signatory Institution accepts the CIRB approval. The Signatory site may be contacted by the CTSU Regulatory Office or asked to complete information verifying the participating institutions on the study. Other site registration requirements (i.e., laboratory certifications, protocol-specific training certifications, or modality credentialing) must be submitted to the CTSU Regulatory Office or compliance communicated per protocol instructions."

SWOG Authorized Document

13. **Pages 46-47, Section 13.4, Registration Procedures**: Registrations procedures in this section have been replaced with the updated standard language.

14. **Page 54, Section 15.2e.1, Plasma, Buffy Coat, and Whole Blood Specimen Banking**: The laboratory contact for Lab #201 has been updated.

Institutions **must** update their local consent forms to include the changes to the Model Consent Form **within 90 days** of distribution of this notice.

SWOG considers that the Model Consent Form changes **do not** represent an alteration in risk/benefit ratio. Therefore, local accrual does **not** need to be suspended pending implementation of these changes.

Patients currently receiving everolimus/placebo (or in follow-up on this study) and patients who sign a consent form prior to local implementation of the consent form changes **must** be informed of the following changes. The manner by which this notification takes place is at the discretion of the local institution. At a minimum, the patient must be notified by the next visit and this notification process must be documented in the patient chart.

Note: CTSU will facilitate a Spanish-language translation of the model consent form as soon as the revision is posted on their bi-monthly mailout. This process can take approximately 4-6 weeks, depending on the complexity of the changes and the need for CIRB review and approval. Please refer to the CTSU website (www.ctsu.org) to access the Spanish-language model consent form.

The model consent form has been revised as follows:

1. The version date of the model consent form has been updated.

2. **Page 5, During the study...**: The first paragraph has been updated to provide clarification as follows:

   Original: "...need to come to the clinic for doctor visits every six weeks for the first 54 weeks while on treatment. After you are finished with the study treatment, you will return to the clinic with scans every six months for the first two years and then yearly thereafter until 10 years after registration."

   New: "...need to come to the clinic for doctor visits every six weeks for the first 54 weeks ~~while on treatment~~ *including visits for scans every 18 weeks (or every 4 months)*. After you are finished with the study treatment, you will return to the clinic with scans every six months for ~~the first two years~~ *Years 2 and 3,* and then yearly thereafter until 10 years after registration."

3. **Pages 6-7, What side effects or risks can I expect from being in the study?**: The everolimus/placebo risks and side effects list has been revised to be consistent with the most recent version of the Investigator's Brochure. Specifically, risk attributions that have changed include the following:

   - Changed from 'Common, some may be serious' to 'Occasional, some may serious': *Vomiting; cough, difficult or labored breathing*
   - Added to 'occasional, some may be serious': **inflammation of the lungs (pneumonia); decrease in blood platelets, which can increase your risk of**

bleeding or bruising; decrease in white blood cells, which can increase your risk of infection

- Added to 'rare, and serious': **allergic reaction, which can cause swelling of the face or throat; severe reduction in blood cells, which can cause weakness, bruising, or make infections more likely**

4. **Page 8, What side effects or risks can I expect from being in the study?**: The paragraph that begins with "The study drug may interact with other medications..." has been updated to address the risk of pneumocystis jirovecii pneumonia (PJP) as follows:

   <u>Original</u>
   "The study drug may interact with other medications. You should tell your study doctor about all medications (over the counter, herbal, and prescription) you are currently taking and check with your study doctor before beginning any new medications."

   <u>New</u>
   "The study drug may interact with other medications. *Cases of a type of pneumonia (caused by a fungus called Pneumocystis jirovecii), some with a fatal outcome, have been reported in patients who received everolimus. This type of pneumonia has been linked to the use of corticosteroids or other drugs that can weaken the immune system while using everolimus.* You should tell your study doctor about all medications (over the counter, herbal, and prescription) you are currently taking and check with your study doctor before beginning any new medications."

5. **Page 9, Will my medical information be kept private?**: The paragraph that begins with "A description of this clinical trial will be available.." has been corrected to the current NCI approved language as follows:

   Original: "A description of this clinical trial will be available on http://www.ClinicalTrials.gov. This Web site will not include information that can identify you. At most, the Web site will include a summary of study results. You can search this Web site at any time."

   New: "A description of this clinical trial will be available on http://www.ClinicalTrials.gov, *as required by U.S. Law*. This Web site will not include information that can identify you. At most, the Web site will include a summary of ~~study~~ *the* results. You can search this Web site at any time."

Due to extensive repagination, an entire replacement protocol is attached. Please discard any previous versions of the protocol, and attach this memorandum to the front of your copy of <u>S0931</u>.

This memorandum serves to notify the NCI and the SWOG Statistical Center.

cc: PROTOCOL & INFORMATION OFFICE
Cathy M. Tangen, Dr.P.H.
Melissa Plets, M.S.
Jean Barce
Austin Hamm
Amy Johnson
Meredith Lavin – Novartis
Heather Campbell, Pharm.D. – VACSP
Sharon Jenkins - VACSP

SWOG
Leading cancer research. Together.

GROUP CHAIR'S OFFICE
Charles D. Blanke, MD
CHAIR
3181 SW Sam Jackson Pk Rd
MC: L586
Portland, OR 97239
503-494-5586
503-346-8038 FAX

OPERATIONS OFFICE
4201 Medical Dr
Suite 250
San Antonio, TX 78229
210-614-8808
210-614-0006 FAX

STATISTICAL CENTER
1730 Minor Ave
Suite 1900
Seattle, WA 98101
206-652-2267
206-342-1616 FAX

1100 Fairview Ave North
M3-C102
PO Box 19024
Seattle, WA 98109
206-667-4623
206-667-4408 FAX

swog.org

March 15, 2015

TO: ALL NATIONAL CLINICAL TRIALS NETWORK (NCTN) MEMBERS

FROM: SWOG Operations Office

RE: IND Safety Reports for Everolimus

MEMORANDUM

IRB Review Requirements

( ) Full board review required. Reason:
- ( ) Initial activation (should your institution choose to participate)
- ( ) Increased risk to patient
- ( ) Complete study redesign
- ( ) Addition of tissue banking requirements
- ( ) Study closure due to new risk information

(√) Expedited review allowed

( ) No review required

MEMORANDUM

The following new safety reports have been posted regarding adverse events that occurred in association with the drug everolimus. Please access these safety reports via the study's abstract page or the safety report link on the SWOG website (https://swog.org/safetyreports/safetyreports.asp).

These safety reports pertain to the following studies:	Reports:
**S0528** Early Therapeutics	Feb. 5, 2015 Mfr Rpt #PHHO2015DE001378
**S0931** Genitourinary	Feb. 23, 2015 Mfr Rpt #PHHO2014DE012547
**S1207** Breast	
**S1222** Breast	

Protocol amendments are not necessary at this time, but your consent form may be revised to include the information provided in these reports if it is deemed necessary by your institution. Please append this notice and these reports to your copy of the protocol and forward copies to your Institutional Review Board (IRB) as required by your local policies and procedures. Should any further information regarding these adverse events be made available, it will be forwarded to you.

This memorandum serves to notify the NCI and the SWOG Statistical Center.

cc:
PROTOCOL & INFORMATION OFFICE
Megan Othus, Ph.D.
Cathy M. Tangen, Dr.P.H.
William Barlow, Ph.D.
Danika Lew, M.S.
Melissa Plets, M.S.
Jean Barce
Jennie Barrett
Carol French – AstraZeneca
William Turnbull – AstraZeneca
Jean Sowa – AstraZeneca
Kathleen DeRose – GSK
Royce Ann Adkins – Path-tec
Meredith Lavin – Novartis
Kristin White – Novartis
Heather Campbell, PharmD – VACSP/PCC

SWOG Authorized Document

Austin Hamm
Louise Highleyman
Larry Kaye
Laura Kingsbury, M.R.T.
Christine McLeod
Iris Syquia
Craig Silva

Anne Davis – VACSP/PCC
Michael Moody – VACSP/PCC
Sharon Jenkins – VACSP/PCC
James Rasp – VACSP/PCC
Emily Tabinski – VACSP/PCC
Katie VonDerau – WPC

SWOG Authorized Document

SWOG
Leading cancer research. Together.

GROUP CHAIR'S OFFICE
Charles D. Blanke, MD
CHAIR
3181 SW Sam Jackson Pk Rd
MC: L586
Portland, OR 97239
503-494-5586
503-346-8038 FAX

OPERATIONS OFFICE
4201 Medical Dr
Suite 250
San Antonio, TX 78229
210-614-8808
210-614-0006 FAX

STATISTICAL CENTER
1730 Minor Ave
Suite 1900
Seattle, WA 98101
206-652-2267
206-342-1616 FAX

1100 Fairview Ave North
M3-C102
PO Box 19024
Seattle, WA 98109
206-667-4623
206-667-4408 FAX

swog.org

February 1, 2015

TO: ALL NATIONAL CLINICAL TRIALS NETWORK (NCTN) MEMBERS

FROM: SWOG Operations Office

RE: IND Safety Reports for Everolimus

MEMORANDUM

IRB Review Requirements

( ) Full board review required. Reason:
- ( ) Initial activation (should your institution choose to participate)
- ( ) Increased risk to patient
- ( ) Complete study redesign
- ( ) Addition of tissue banking requirements
- ( ) Study closure due to new risk information

(√) Expedited review allowed

( ) No review required

MEMORANDUM

The following new safety reports have been posted regarding adverse events that occurred in association with the drug everolimus. Please access these safety reports via the study's abstract page or the safety report link on the SWOG website (https://swog.org/safetyreports/safetyreports.asp).

These safety reports pertain to the following studies:	Reports:	
**S0528** Early Therapeutics	Aug. 28, 2014	Mfr Rpt #PHHY2014BE103661
**S0931** Genitourinary	Sep. 5, 2014	Mfr Rpt #PHHO2014JP009849
**S1207** Breast	Jan. 9, 2015	Mfr Rpt #PHHO2014DE017192
**S1222** Breast	Jan. 12, 2015	Mfr Rpt #PHHO2014ES017327

Protocol amendments are not necessary at this time, but your consent form may be revised to include the information provided in these reports if it is deemed necessary by your institution. Please append this notice and these reports to your copy of the protocol and forward copies to your Institutional Review Board (IRB) as required by your local policies and procedures. Should any further information regarding these adverse events be made available, it will be forwarded to you.

This memorandum serves to notify the NCI and the SWOG Statistical Center.

cc: PROTOCOL & INFORMATION OFFICE
Megan Othus, Ph.D.
Cathy M. Tangen, Dr.P.H.
William Barlow, Ph.D.
Danika Lew, M.S.
Melissa Plets, M.S.
Jean Barce
Jennie Barrett
Carol French – AstraZeneca
John Parnell – AstraZeneca
Jean Sowa – AstraZeneca
Kathleen DeRose – GSK
Royce Ann Adkins – Path-tec
Meredith Lavin – Novartis
Kristin White – Novartis
Heather Campbell, PharmD – VACSP/PCC

SWOG Authorized Document

Austin Hamm
Larry Kaye
Laura Kingsbury, M.R.T.
Christine McLeod
Iris Syquia
Craig Silva

Anne Davis – VACSP/PCC
Michael Moody – VACSP/PCC
Sharon Jenkins – VACSP/PCC
James Rasp – VACSP/PCC
Emily Tabinski – VACSP/PCC
Katie VonDerau – WPC

SWOG Authorized Document

SWOG
Leading cancer research. Together.

GROUP CHAIR'S OFFICE
Charles D. Blanke, MD
CHAIR
3181 SW Sam Jackson Pk Rd
MC: L586
Portland, OR 97239
503-494-5586
503-346-8038 FAX

OPERATIONS OFFICE
4201 Medical Dr
Suite 250
San Antonio, TX 78229
210-614-8808
210-614-0006 FAX

STATISTICAL CENTER
1730 Minor Ave
Suite 1900
Seattle, WA 98101
206-652-2267
206-342-1616 FAX

1100 Fairview Ave North
M3-C102
PO Box 19024
Seattle, WA 98109
206-667-4623
206-667-4408 FAX

swog.org

January 15, 2015

TO: ALL NATIONAL CLINICAL TRIALS NETWORK (NCTN) MEMBERS

FROM: SWOG Operations Office

RE: IND Safety Reports for Everolimus

MEMORANDUM

IRB Review Requirements

( ) Full board review required. Reason:
- ( ) Initial activation (should your institution choose to participate)
- ( ) Increased risk to patient
- ( ) Complete study redesign
- ( ) Addition of tissue banking requirements
- ( ) Study closure due to new risk information

(√) Expedited review allowed

( ) No review required

MEMORANDUM

The following new safety reports have been posted regarding adverse events that occurred in association with the drug everolimus. Please access these safety reports via the study's abstract page or the safety report link on the SWOG website (https://swog.org/safetyreports/safetyreports.asp).

These safety reports pertain to the following studies:	Reports:
**S0528** Early Therapeutics	Dec. 17, 2014 Mfr Rpt #PHH02014AR004429
**S0931** Genitourinary	Dec. 19, 2014 Mfr Rpt #PHHO2014FR016631
**S1207** Breast	
**S1222** Breast	

Protocol amendments are not necessary at this time, but your consent form may be revised to include the information provided in these reports if it is deemed necessary by your institution. Please append this notice and these reports to your copy of the protocol and forward copies to your Institutional Review Board (IRB) as required by your local policies and procedures. Should any further information regarding these adverse events be made available, it will be forwarded to you.

This memorandum serves to notify the NCI and the SWOG Statistical Center.

cc: PROTOCOL & INFORMATION OFFICE
Megan Othus, Ph.D.
Cathy M. Tangen, Dr.P.H.
William Barlow, Ph.D.
Danika Lew, M.S.
Melissa Plets, M.S.
Jean Barce
Jennie Barrett
Carol French – AstraZeneca
John Parnell – AstraZeneca
Jean Sowa – AstraZeneca
Kathleen DeRose – GSK
Royce Ann Adkins – Path-tec
Meredith Lavin – Novartis
Kristin White – Novartis
Heather Campbell, PharmD – VACSP/PCC

SWOG Authorized Document

Austin Hamm	Anne Davis – VACSP/PCC
Larry Kaye	Michael Moody – VACSP/PCC
Laura Kingsbury, M.R.T.	Sharon Jenkins – VACSP/PCC
Christine McLeod	James Rasp – VACSP/PCC
Iris Syquia	Emily Tabinski – VACSP/PCC
Craig Silva	Katie VonDerau – WPC

SWOG Authorized Document

SWOG
Leading cancer research. Together.

GROUP CHAIR'S OFFICE
Charles D. Blanke, MD
CHAIR
3181 SW Sam Jackson Pk Rd
MC: L586
Portland, OR 97239
503-494-5586
503-346-8038 FAX

OPERATIONS OFFICE
4201 Medical Dr
Suite 250
San Antonio, TX 78229
210-614-8808
210-614-0006 FAX

STATISTICAL CENTER
1730 Minor Ave
Suite 1900
Seattle, WA 98101
206-652-2267
206-342-1616 FAX

1100 Fairview Ave North
M3-C102
PO Box 19024
Seattle, WA 98109
206-667-4623
206-667-4408 FAX

swog.org

January 1, 2015

TO: ALL NATIONAL CLINICAL TRIALS NETWORK (NCTN) MEMBERS

FROM: SWOG Operations Office

RE: IND Safety Report for Everolimus

MEMORANDUM

IRB Review Requirements

( ) Full board review required. Reason:
- ( ) Initial activation (should your institution choose to participate)
- ( ) Increased risk to patient
- ( ) Complete study redesign
- ( ) Addition of tissue banking requirements
- ( ) Study closure due to new risk information

(√) Expedited review allowed

( ) No review required

MEMORANDUM

The following new safety report has been posted regarding an adverse event that occurred in association with the drug everolimus. Please access this safety report via the study's abstract page or the safety report link on the SWOG website (https://swog.org/safetyreports/safetyreports.asp).

This safety report pertains to the following studies:	Report:
**S0528** Early Therapeutics	Dec. 15, 2014 AE #2086422
**S0931** Genitourinary	
**S1207** Breast	
**S1222** Breast	

Protocol amendments are not necessary at this time, but your consent form may be revised to include the information provided in this report if it is deemed necessary by your institution. Please append this notice and this report to your copy of the protocol and forward a copy to your Institutional Review Board (IRB) as required by your local policies and procedures. Should any further information regarding this adverse event be made available, it will be forwarded to you.

This memorandum serves to notify the NCI and the SWOG Statistical Center.

cc:
PROTOCOL & INFORMATION OFFICE
Megan Othus, Ph.D.
Cathy M. Tangen, Dr.P.H.
William Barlow, Ph.D.
Danika Lew, M.S.
Melissa Plets, M.S.
Jean Barce
Jennie Barrett
Austin Hamm
Carol French – AstraZeneca
John Parnell – AstraZeneca
Jean Sowa – AstraZeneca
Kathleen DeRose – GSK
Royce Ann Adkins – Path-tec
Meredith Lavin – Novartis
Kristin White – Novartis
Heather Campbell, PharmD – VACSP/PCC
Anne Davis – VACSP/PCC

SWOG Authorized Document

Larry Kaye
Laura Kingsbury, M.R.T.
Christine McLeod
Iris Syquia
Craig Silva
Louise Highleyman

Michael Moody – VACSP/PCC
Sharon Jenkins – VACSP/PCC
James Rasp – VACSP/PCC
Emily Tabinski – VACSP/PCC
Katie VonDerau – WPC

SWOG Authorized Document

SWOG
Leading cancer research. Together.

GROUP CHAIR'S OFFICE
Charles D. Blanke, MD
CHAIR
3181 SW Sam Jackson Pk Rd
MC: L586
Portland, OR 97239
503-494-5586
503-346-8038 FAX

OPERATIONS OFFICE
4201 Medical Dr
Suite 250
San Antonio, TX 78229
210-614-8808
210-614-0006 FAX

STATISTICAL CENTER
1730 Minor Ave
Suite 1900
Seattle, WA 98101
206-652-2267
206-342-1616 FAX

1100 Fairview Ave North
M3-C102
PO Box 19024
Seattle, WA 98109
206-667-4623
206-667-4408 FAX

swog.org

December 15, 2014

TO: ALL NATIONAL CLINICAL TRIALS NETWORK (NCTN) MEMBERS

FROM: SWOG Operations Office

RE: IND Safety Report for Everolimus

MEMORANDUM

IRB Review Requirements

( ) Full board review required. Reason:
- ( ) Initial activation (should your institution choose to participate)
- ( ) Increased risk to patient
- ( ) Complete study redesign
- ( ) Addition of tissue banking requirements
- ( ) Study closure due to new risk information

(√) Expedited review allowed

( ) No review required

MEMORANDUM

The following new safety report has been posted regarding an adverse event that occurred in association with the drug everolimus. Please access this safety report via the study's abstract page or the safety report link on the SWOG website (https://swog.org/safetyreports/safetyreports.asp).

This safety report pertains to the following studies:	Report:
**S0528** Early Therapeutics<br>**S0931** Genitourinary<br>**S1207** Breast<br>**S1222** Breast	Nov. 24, 2014 Mfr Rpt #2140561

Protocol amendments are not necessary at this time, but your consent form may be revised to include the information provided in this report if it is deemed necessary by your institution. Please append this notice and this report to your copy of the protocol and forward a copy to your Institutional Review Board (IRB) as required by your local policies and procedures. Should any further information regarding this adverse event be made available, it will be forwarded to you.

This memorandum serves to notify the NCI and the SWOG Statistical Center.

cc:
PROTOCOL & INFORMATION OFFICE
Megan Othus, Ph.D.
Cathy M. Tangen, Dr.P.H.
William Barlow, Ph.D.
Danika Lew, M.S.
Melissa Plets, M.S.
Jean Barce
Jennie Barrett
Austin Hamm
Carol French – AstraZeneca
John Parnell – AstraZeneca
Jean Sowa – AstraZeneca
Kathleen DeRose – GSK
Royce Ann Adkins – Path-tec
Meredith Lavin – Novartis
Kristin White – Novartis
Heather Campbell, PharmD – VACSP/PCC
Anne Davis – VACSP/PCC

SWOG Authorized Document

Larry Kaye	Michael Moody – VACSP/PCC
Laura Kingsbury, M.R.T.	Sharon Jenkins – VACSP/PCC
Christine McLeod	James Rasp – VACSP/PCC
Iris Syquia	Emily Tabinski – VACSP/PCC
Craig Silva	Katie VonDerau – WPC

SWOG Authorized Document

SWOG
Leading cancer research. Together.

GROUP CHAIR'S OFFICE
Charles D. Blanke, MD
CHAIR
3181 SW Sam Jackson Pk Rd
MC: L586
Portland, OR 97239
503-494-5586
503-346-8038 FAX

OPERATIONS OFFICE
4201 Medical Dr
Suite 250
San Antonio, TX 78229
210-614-8808
210-614-0006 FAX

STATISTICAL CENTER
1730 Minor Ave
Suite 1900
Seattle, WA 98101
206-652-2267
206-342-1616 FAX

1100 Fairview Ave North
M3-C102
PO Box 19024
Seattle, WA 98109
206-667-4623
206-667-4408 FAX

swog.org

December 1, 2014

TO: ALL NATIONAL CLINICAL TRIALS NETWORK (NCTN) MEMBERS

FROM: SWOG Operations Office

RE: IND Safety Report for Everolimus

MEMORANDUM

IRB Review Requirements

( ) Full board review required. Reason:
- ( ) Initial activation (should your institution choose to participate)
- ( ) Increased risk to patient
- ( ) Complete study redesign
- ( ) Addition of tissue banking requirements
- ( ) Study closure due to new risk information

(√) Expedited review allowed

( ) No review required

MEMORANDUM

The following new safety report has been posted regarding an adverse event that occurred in association with the drug everolimus. Please access this safety report via the study's abstract page or the safety report link on the SWOG website (https://swog.org/safetyreports/safetyreports.asp).

This safety report pertains to the following studies:	Report:
**S0528** Early Therapeutics<br>**S0931** Genitourinary<br>**S1207** Breast<br>**S1222** Breast	Nov. 18, 2014 Mfr Rpt #PHHO2014US013821 FU

Protocol amendments are not necessary at this time, but your consent form may be revised to include the information provided in this report if it is deemed necessary by your institution. Please append this notice and this report to your copy of the protocol and forward a copy to your Institutional Review Board (IRB) as required by your local policies and procedures. Should any further information regarding this adverse event be made available, it will be forwarded to you.

This memorandum serves to notify the NCI and the SWOG Statistical Center.

cc:
PROTOCOL & INFORMATION OFFICE
Megan Othus, Ph.D.
Cathy M. Tangen, Dr.P.H.
William Barlow, Ph.D.
Danika Lew, M.S.
Melissa Plets, M.S.
Jean Barce
Jennie Barrett
Carol French – AstraZeneca
John Parnell – AstraZeneca
Jean Sowa – AstraZeneca
Kathleen DeRose – GSK
Royce Ann Adkins – Path-tec
Meredith Lavin – Novartis
Kristin White – Novartis
Heather Campbell, PharmD – VACSP/PCC

SWOG Authorized Document

Austin Hamm
Larry Kaye
Laura Kingsbury, M.R.T.
Christine McLeod
Iris Syquia
Craig Silva

Anne Davis – VACSP/PCC
Michael Moody – VACSP/PCC
Sharon Jenkins – VACSP/PCC
James Rasp – VACSP/PCC
Emily Tabinski – VACSP/PCC
Katie VonDerau – WPC

SWOG Authorized Document

SWOG
Leading cancer research. Together.

GROUP CHAIR'S OFFICE
Charles D. Blanke, MD
CHAIR
3181 SW Sam Jackson Pk Rd
MC: L586
Portland, OR 97239
503-494-5586
503-346-8038 FAX

OPERATIONS OFFICE
4201 Medical Dr
Suite 250
San Antonio, TX 78229
210-614-8808
210-614-0006 FAX

STATISTICAL CENTER
1730 Minor Ave
Suite 1900
Seattle, WA 98101
206-652-2267
206-342-1616 FAX

1100 Fairview Ave North
M3-C102
PO Box 19024
Seattle, WA 98109
206-667-4623
206-667-4408 FAX

swog.org

November 15, 2014

TO: ALL NATIONAL CLINICAL TRIALS NETWORK (NCTN) MEMBERS

FROM: SWOG Operations Office

RE: IND Safety Report for Everolimus

MEMORANDUM

IRB Review Requirements

( ) Full board review required. Reason:
- ( ) Initial activation (should your institution choose to participate)
- ( ) Increased risk to patient
- ( ) Complete study redesign
- ( ) Addition of tissue banking requirements
- ( ) Study closure due to new risk information

(√) Expedited review allowed

( ) No review required

MEMORANDUM

The following new safety report has been posted regarding an adverse event that occurred in association with the drug everolimus. Please access this safety report via the study's abstract page or the safety report link on the SWOG website (https://swog.org/safetyreports/safetyreports.asp).

This safety report pertains to the following studies:	Reports:	
**S0528** Early Therapeutics	Nov. 4, 2014	Mfr Rpt #PHHO2014FR008155
**S0931** Genitourinary	Nov. 4, 2014	Mfr Rpt #PHHO2014US013821
**S1207** Breast		
**S1222** Breast		

Protocol amendments are not necessary at this time, but your consent form may be revised to include the information provided in this report if it is deemed necessary by your institution. Please append this notice and this report to your copy of the protocol and forward a copy to your Institutional Review Board (IRB) as required by your local policies and procedures. Should any further information regarding this adverse event be made available, it will be forwarded to you.

SWOG Authorized Document

This memorandum serves to notify the NCI and the SWOG Statistical Center.

cc:	PROTOCOL & INFORMATION OFFICE	Carol French – AstraZeneca
	Megan Othus, Ph.D.	John Parnell – AstraZeneca
	Cathy M. Tangen, Dr.P.H.	Jean Sowa – AstraZeneca
	William Barlow, Ph.D.	Kathleen DeRose – GSK
	Danika Lew, M.S.	Royce Ann Adkins – Path-tec
	Melissa Plets, M.S.	Meredith Lavin – Novartis
	Jean Barce	Kristin White – Novartis
	Jennie Barrett	Heather Campbell, PharmD – VACSP/PCC
	Austin Hamm	Anne Davis – VACSP/PCC
	Larry Kaye	Michael Moody – VACSP/PCC
	Laura Kingsbury, M.R.T.	Sharon Jenkins – VACSP/PCC
	Christine McLeod	James Rasp – VACSP/PCC
	Iris Syquia	Emily Tabinski – VACSP/PCC
	Craig Silva	Katie VonDerau – WPC

SWOG
Authorized
Document

SWOG
Leading cancer research. Together.

GROUP CHAIR'S OFFICE
Charles D. Blanke, MD
CHAIR
3181 SW Sam Jackson Pk Rd
MC: L586
Portland, OR 97239
503-494-5586
503-346-8038 FAX

OPERATIONS OFFICE
4201 Medical Dr
Suite 250
San Antonio, TX 78229
210-614-8808
210-614-0006 FAX

STATISTICAL CENTER
1730 Minor Ave
Suite 1900
Seattle, WA 98101
206-652-2267
206-342-1616 FAX

1100 Fairview Ave North
M3-C102
PO Box 19024
Seattle, WA 98109
206-667-4623
206-667-4408 FAX

swog.org

September 15, 2014

TO: ALL NATIONAL CLINICAL TRIALS NETWORK (NCTN) MEMBERS

FROM: SWOG Operations Office

RE: IND Safety Report for Everolimus

MEMORANDUM

IRB Review Requirements

( ) Full board review required. Reason:
- ( ) Initial activation (should your institution choose to participate)
- ( ) Increased risk to patient
- ( ) Complete study redesign
- ( ) Addition of tissue banking requirements
- ( ) Study closure due to new risk information

(√) Expedited review allowed

( ) No review required

MEMORANDUM

The following new safety report has been posted regarding an adverse event that occurred in association with the drug everolimus. Please access this safety report via the study's abstract page or the safety report link on the SWOG website (https://swog.org/safetyreports/safetyreports.asp).

This safety report pertains to the following studies:	Reports:
**S0528** Early Therapeutics	Aug. 28, 2014 Mfr Rpt #PHHO2014ES011418
**S0931** Genitourinary	Sep. 4, 2014 Mfr Rpt #PHHO2014JP009849
**S1207** Breast	
**S1222** Breast	

Protocol amendments are not necessary at this time, but your consent form may be revised to include the information provided in this report if it is deemed necessary by your institution. Please append this notice and this report to your copy of the protocol and forward a copy to your Institutional Review Board (IRB) as required by your local policies and procedures. Should any further information regarding this adverse event be made available, it will be forwarded to you.

This memorandum serves to notify the NCI and the SWOG Statistical Center.

cc:
PROTOCOL & INFORMATION OFFICE
Megan Othus, Ph.D.
Cathy M. Tangen, Dr.P.H.
William Barlow, Ph.D.
Danika Lew, M.S.
Melissa Plets, M.S.
Jean Barce
Jennie Barrett
Carol French – AstraZeneca
John Parnell – AstraZeneca
Jean Sowa – AstraZeneca
Kathleen DeRose – GSK
Royce Ann Adkins – Path-tec
Meredith Lavin – Novartis
Kristin White – Novartis
Heather Campbell, PharmD – VACSP/PCC

SWOG Authorized Document

Austin Hamm	Anne Davis – VACSP/PCC
Larry Kaye	Michael Moody – VACSP/PCC
Laura Kingsbury, M.R.T.	Sharon Jenkins – VACSP/PCC
Christine McLeod	James Rasp – VACSP/PCC
Iris Syquia	Emily Tabinski – VACSP/PCC
Craig Silva	Katie VonDerau – WPC

SWOG Authorized Document

SWOG
Leading cancer research. Together.

GROUP CHAIR'S OFFICE
Charles D. Blanke, MD
CHAIR
3181 SW Sam Jackson Pk Rd
MC: L586
Portland, OR 97239
503-494-5586
503-346-8038 FAX

OPERATIONS OFFICE
4201 Medical Dr
Suite 250
San Antonio, TX 78229
210-614-8808
210-614-0006 FAX

STATISTICAL CENTER
1730 Minor Ave
Suite 1900
Seattle, WA 98101
206-652-2267
206-342-1616 FAX

1100 Fairview Ave North
M3-C102
PO Box 19024
Seattle, WA 98109
206-667-4623
206-667-4408 FAX

swog.org

August 15, 2014

TO: ALL NATIONAL CLINICAL TRIALS NETWORK (NCTN) MEMBERS

FROM: SWOG Operations Office

RE: IND Safety Report for Everolimus

MEMORANDUM

IRB Review Requirements

( ) Full board review required. Reason:
- ( ) Initial activation (should your institution choose to participate)
- ( ) Increased risk to patient
- ( ) Complete study redesign
- ( ) Addition of tissue banking requirements
- ( ) Study closure due to new risk information

(√) Expedited review allowed

( ) No review required

MEMORANDUM

The following new safety report has been posted regarding an adverse event that occurred in association with the drug everolimus. Please access this safety report via the study's abstract page or the safety report link on the SWOG website (https://swog.org/safetyreports/safetyreports.asp).

This safety report pertains to the following studies:	Report:
**S0528** Early Therapeutics<br>**S0931** Genitourinary<br>**S1207** Breast<br>**S1222** Breast	Aug. 5, 2014 Mfr Rpt #PHHO2014JP010364

Protocol amendments are not necessary at this time, but your consent form may be revised to include the information provided in this report if it is deemed necessary by your institution. Please append this notice and this report to your copy of the protocol and forward a copy to your Institutional Review Board (IRB) as required by your local policies and procedures. Should any further information regarding this adverse event be made available, it will be forwarded to you.

SWOG Authorized Document

This memorandum serves to notify the NCI and the SWOG Statistical Center.

cc:
PROTOCOL & INFORMATION OFFICE
Megan Othus, Ph.D.
Cathy M. Tangen, Dr.P.H.
William Barlow, Ph.D.
Danika Lew, M.S.
Melissa Plets, M.S.
Rachel Sexton, M.S.
Jean Barce
Jennie Barrett
Austin Hamm
Larry Kaye
Laura Kingsbury, M.R.T.
Christine McLeod
Iris Syquia
Brian Zeller

Carol French – AstraZeneca
John Parnell – AstraZeneca
Jean Sowa – AstraZeneca
Kathleen DeRose – GSK
Scott Adams – Janssen
Meredith Lavin – Novartis
Kristin White – Novartis
Heather Campbell, PharmD – VACSP/PCC
Anne Davis – VACSP/PCC
Michael Moody – VACSP/PCC
Sharon Jenkins – VACSP/PCC
James Rasp – VACSP/PCC
Emily Tabinski – VACSP/PCC
Katie VonDerau – WPC

SWOG Authorized Document

SWOG
Leading cancer research. Together.

GROUP CHAIR'S OFFICE
Charles D. Blanke, MD
CHAIR
3181 SW Sam Jackson Pk Rd
MC: L586
Portland, OR 97239
503-494-5586
503-346-8038 FAX

OPERATIONS OFFICE
4201 Medical Dr
Suite 250
San Antonio, TX 78229
210-614-8808
210-614-0006 FAX

STATISTICAL CENTER
1730 Minor Ave
Suite 1900
Seattle, WA 98101
206-652-2267
206-342-1616 FAX

1100 Fairview Ave North
M3-C102
PO Box 19024
Seattle, WA 98109
206-667-4623
206-667-4408 FAX

swog.org

Distribution Date: June 15, 2014
CTEP Submission Date: May 6, 2014

TO: ALL NATIONAL CLINICAL TRIALS NETWORK (NCTN) MEMBERS – U.S. INSTITUTIONS ONLY

FROM: Gilbert R. Carrizales, Protocol Coordinator (E-mail: gcarrizales@swog.org)

RE: **S0931**, "EVEREST: EVErolimus for Renal Cancer Ensuing Surgical Therapy, A Phase III Study. Study Chairs: Drs. C.W. Ryan, E.I. Heath, P.N. Lara, G.S. Palapattu, and P.C. Mack.

REVISION #3

Study Chair: Christopher W. Ryan, M.D.
Phone number: 503/494-8487
E-mail: ryanc@ohsu.edu

IRB Review Requirements

( ) Full board review required. Reason:
- ( ) Initial activation (should your institution choose to participate)
- ( ) Increased risk to patient
- ( ) Complete study redesign
- ( ) Addition of tissue banking requirements
- ( ) Study closure due to new risk information

(√) Expedited review allowed

( ) No review required

REVISION #3

The protocol has been reformatted and repaginated to meet the current requirements for electronic protocol submission. This includes addition of second level headings in instances where they were previously absent, reformatting the title page to include all second level headings, reformatting the protocol calendar into Microsoft® Word, removal of form numbers, and removal of the consent form as Section 18.0. Additionally, typographical errors and cross-references have been corrected as needed.

The protocol document has been revised as follows:

1. **Pages 1 – 5**: The following changes have been made to these pages:
   - The version date for the protocol and model consent form has been updated (5/6/14).
   - The clinicaltrials.gov identifier (NCT number) has been added below the study title.
   - The heading "Study Coordinators" has been changed to "Study *Chairs*". The change from "Study Coordinators" to "Study *Chairs*" was also made throughout the protocol in Section 7.1 (page 22) and Section 8.9 (page 35).

SWOG Authorized Document

- The pages have been reorganized so that the contact information for all **S0931** co-Study Chairs is now listed on page 1, and the table of contents is listed on pages 3-4.
- Biostatisticians: The SWOG Statistical Center address has been updated. “1100 Fairview Avenue North, MP-557” has been changed to “1100 Fairview Avenue North, *M3-C102*”
- The e-mail addresses for Dr. Maxwell Meng (Alliance Study Chair) and Dr. Mark Stein (ECOG-ACRIN Study Chair) have been updated. ‘CALGB’ and ‘ECOG’ have been changed to ‘*ALLIANCE*’ and ‘*ECOG-ACRIN*’, respectively, in the study chair headings.
- The participant instructions have been revised as follows:

  Original: “ALL SWOG MEMBER, CCOP, AND AFFILIATE MEDICAL ONCOLOGISTS AND SURGEONS; CTSU-U.S. INSTITUTIONS ONLY”

  New: “ALL *NATIONAL CLINICAL TRIALS NETWORK (NCTN) MEMBERS* – U.S. INSTITUTIONS ONLY

- The list of NCTN participating organizations has been added.
- The table of contents has been updated and includes the pages where the participant list and the CTSU contact information are located.
- An updated CTSU contact information page has replaced the previous version. Changes to this page include revised instructions for patient enrollments, data submission, protocol (and related forms) downloading, and regulatory and monitoring for CTSU sites.
- Due to the above changes, the remainder of the protocol has been repaginated.

2. **Pages 12-14, Section 3.1c, Everolimus Drug Information:** Due to recent updates with the everolimus investigator brochure (edition 12), the following changes have been made to the adverse effects table:

**Adverse Events with Possible Relationship to Everolimus**		
**Likely (>20%)**	**Less Likely (≤20%)**	**Rare but Serious (<3%)**
BLOOD AND LYMPHATIC SYSTEM DISORDERS		
Anemia	~~Neutropenia~~<br>***Leukopenia***	***Pancytopenia***
~~Leukopenia~~	***Lymphopenia***	***Pure red cell aplasia***
Thrombocytopenia	***Neutropenia***	
CARDIAC DISORDERS		
	~~Tachycardia~~	Congestive cardiac failure
~~EYE DISORDERS~~		
	~~Eyelid edema~~	
	~~Conjunctivitis~~	
GASTROINTESTINAL DISORDERS		
Stomatitis	~~Hemorrhoids~~***Oral pain***	
Diarrhea	Dysphagia	
Nausea	Dry mouth	
Vomiting	~~Vomiting~~***Dyspepsia***	
	Abdominal pain	
GENERAL DISORDERS AND ADMINISTRATION SITE CONDITIONS		
Asthenia	~~Chest pain~~***Non-cardiac chest pain***	***Impaired wound healing***
Fatigue	~~Chills~~	
~~Edema peripheral~~	~~Impaired wound healing~~	
Peripheral edema	~~Fever~~***Pyrexia***	
	Mucosal inflammation	
	~~Weight decreased~~	
IMMUNE SYSTEM DISORDERS		
		Hypersensitivity

SWOG Authorized Document

INFECTIONS AND INFESTATIONS		
Infections	~~Nasopharyngitis~~	
	~~Pneumonia~~	
	~~Urinary tract infection~~	
	~~Bronchitis~~	
	~~Sinusitis~~	
INVESTIGATIONS		
~~Hemoglobin decreased~~***Weight decreased***	~~Neutrophils decreased~~	
~~Lymphocytes decreased~~	~~Bilirubin increased~~	
~~Platelets decreased~~		
~~Cholesterol increased~~		
~~Triglycerides increased~~		
~~Glucose increased~~		
~~Creatinine increased~~	***Creatinine increased***	
~~Phosphate decreased~~		
~~Aspartate transaminase increased~~	***AST increased***	
~~Alanine transaminase increased~~	***ALT increased***	
METABOLISM AND NUTRITION DISORDERS		
~~Anorexia~~ ***Decreased appetite***	~~New onset of diabetes mellitus~~***Diabetes mellitus***	
Hypercholesterolemia	~~Exacerbation of pre-existing diabetes mellitus~~	
Hyperglycemia	***Dehydration***	
	Hypokalemia	
	Hyperlipidemia	
	Hypophosphatemia	
	Hypertriglyceridemia	
MUSCULOSKELETAL AND CONNECTIVE TISSURE DISORDERS		
	~~Jaw pain~~***Arthralgia***	
	~~Pain in extremity~~	
	~~Back pain~~	
	~~Muscle spasms~~	
NERVOUS SYSTEM DISORDERS		
Dysgeusia	~~Headache~~***Ageusia***	
Headache	~~Dysgeusia~~	
	~~Dizziness~~	
	~~Parasthesia~~	
PSYCHIATRIC DISORDERS		
	Insomnia	
RENAL AND URINARY DISORDERS		
	Renal failure	~~Renal failure~~***Acute renal failure***
	Increased daytime urination	***Proteinuria***
REPRODUCTIVE SYSTEM AND BREAST DISORDERS		
	Menstruation irregular	
	Amenorrhea	
RESPIRATORY, THORACIC AND MEDIASTINAL DISORDERS		
Cough	~~Rhinorrhea~~	***Acute respiratory distress syndrome***
Dyspnea	~~Pharyngolaryngeal pain~~	***Hemoptysis***
Epistaxis	~~Epistaxis~~	***Pulmonary embolism***
Pneumonitis	~~Pneumonitis~~	
	~~Pleural effusion~~	

SWOG Authorized Document

SKIN AND SUBCUTANEOUS TISSUE DISORDERS		
Rash	~~Acneiform dermatitis~~***Acne***	
Pruritus	~~Pruritus~~	
	Dry skin	
	Hand-foot syndrome ~~(palmer-plantar erythrodysethesia syndrome)~~	
	Nail disorder	
	Erythema	
	~~Onychoclasis~~	
	~~Skin lesion~~	
VASCULAR DISORDERS		
	Hypertension	~~Hemorrhage~~***Deep vein thrombosis***
	Hemorrhage	

3. **Page 14, Section 3.1c, Everolimus Drug Information:** The following additional information has been added to the everolimus drug interactions section, and the corresponding drug interaction table has been removed:

   Original: "Due to the extensive number of everolimus drug interactions, a complete patient medication list, including everolimus, should be screened prior to initiation of everolimus."

   New: "*Everolimus is a substrate of cytochrome P450 3A4 (CYP3A4), and also a substrate and moderate inhibitor of the multidrug efflux pump P-glycoprotein (PgP). In vitro, everolimus is a competitive inhibitor of CYP3A4 and a mixed inhibitor of CYP2D6.* Due to the extensive number of everolimus drug interactions, a complete patient medication list, including everolimus, should be screened prior to initiation of everolimus *(as indicated in Section 7.3 and Appendix 18.4)*."

4. **Page 14, Section 3.1d, Everolimus Dosing & Administration:** The following editorial changes have been made to the everolimus dosing and administration section:

   - Section 3.1d.1: 'Dosing – See Treatment Plan' has been changed to 'Dosing – See Treatment Plan *(see Section 7.2)*'.
   - Section 3.1d.2: 'Everolimus should be administered orally…" has been changed to 'Everolimus *(or matching placebo)* should be administered orally…"

5. **Page 18, Section 5.1d, Eligibility Criteria**: The following clarifying note regarding subcentimeter pulmonary nodules has been added at the end of this disease related criterion:

   "*Patients who display subcentimeter pulmonary nodules (by CT scan) that are non-specific and considered unlikely to represent metastatic disease by the treating investigator will be considered eligible.*"

6. **Page 19, Section 5.3a, Eligibility Criteria**: The following new criterion related to the prestudy physical examination and medical history has been added (subsequent sections have been reorganized accordingly on pages 19-20):

   "Patients must have a complete physical examination and medical history within 28 days prior to registration".

SWOG Authorized Document

7. **Page 21, Section 5.4a, Eligibility Criteria**: The criterion, 'Patients must be offered the opportunity to participate in specimen banking for future use to include the translational medicine studies outlined in Section 15.0' has been moved to this page under Section 5.4, 'Translational Medicine Criteria'.

8. **Page 25, Section 7.5a, Criteria for Planned Unblinding**: Updated instructions for planned unblinding have replaced the previous section:

   Original
   "Planned unblinding procedure applies to patients who experience recurrence as defined in Section 10.1. It is vital to properly apply the protocol specified definition of recurrence. If any questions arise with regard to recurrence for a patient, please contact the GU Data Coordinator in Seattle by telephone at 206/652-2267, 6:30 a.m. to 4:00 p.m. Pacific Time, Monday through Friday, excluding holidays, or the **S0931** Study Coordinator, Dr. Ryan or in his absence, Dr. Heath.

   Prior to planned unblinding, the **S0931** Recurrence Assessment Form (Form #46940) must be submitted and processed by the SWOG Data Operations Office and the **S0931** Study Coordinator Dr. Ryan (or Dr. Heath in his absence). Dr. Ryan (or Heath) will provide a written confirmation to the site that the patient has an adequately documented recurrence. This documentation should be placed in the patient's chart."

   New
   The planned unblinding procedure applies to patients who experience recurrence as defined in Section 10.1. It is vital to properly apply the protocol specified definition of recurrence. If any questions arise with regard to recurrence for a patient, please contact the GU Data Coordinator in Seattle by telephone at 206/652-2267, 6:30 a.m. to 4:00 p.m. Pacific Time, Monday through Friday, excluding holidays. ~~or the **S0931** Study Coordinator, Dr. Ryan or in his absence, Dr. Heath.~~

   Prior to *requesting a* planned unblinding, the *following* ~~**S0931** Recurrence Assessment Form (Form #46940)~~ forms must be submitted and processed by the SWOG Data Operations Office ~~and the **S0931** Study Coordinator Dr. Ryan (or Dr. Heath in his absence). Dr. Ryan (or Heath) will provide a written confirmation to the site that the patient has an adequately documented recurrence. This documentation should be placed in the patient's chart~~:

   Off Treatment Notice
   ***S0931** Recurrence Assessment Form*
   Radiology Report(s) supporting recurrence (see Section 10.1)
   Pathology Report(s) supporting recurrence (see Section 10.1)

   *Please submit all required forms to the SWOG Data Operations Center (guquestion@crab.org) with the subject line "**S0931** Patient #XXXXXX, Requesting Planned Unblinding". Please allow a minimum of 2 working days for review of forms.*

   Dr. Ryan (or Heath) will provide a written confirmation to the site that the patient has an adequately documented recurrence. This documentation should be placed in the patient's chart."

9. **Pages 36-38, Section 9.0, Study Calendar**: The following changes have been made to these pages:
   - Calendar: The Cycle 2 and Cycle 3 columns have been condensed to reflect only the study-related procedures at the beginning of each cycle.
   - Footnotes: The following changes have been made to the footnote page:

SWOG Authorized Document

- * footnote: This footnote has been added under the history and physical exam (prestudy) column. This footnote corresponds to the H&P 28-day pre-registration requirement noted in Section 5.3a above.
- ¥ footnote: This footnote has been updated with the note, 'DO NOT COLLECT SPECIMENS AT EACH VISIT DURING FOLLOW-UP', to clarify that blood specimens only need to be collected at the time the patient is removed from protocol treatment.
- Я footnote: The timeframe whereby prestudy tests do not need to be repeated for Cycle 1, Day 1 has been changed from '3 weeks' to '*28 days*'.

10. **Pages 47, Section 14.4d, Data Submission Overview Timepoints**: The Recurrence Assessment Form submission instructions have been updated as follows:

    <u>Original</u>
    "EVERY EIGHTEEN WEEKS FOR THE FIRST 54 WEEKS….UNTIL 10 YEARS AFTER REGISTRATION, RECURRENCE OR DEATH WHICHEVER COMES FIRST"

    <u>New</u>
    "EVERY EIGHTEEN WEEKS FOR THE FIRST 54 WEEKS *(REGARDLESS OF EARLY PROTOCOL TREATMENT DISCONTINUATION)*…UNTIL 10 YEARS AFTER REGISTRATION, RECURRENCE OR DEATH, WHICHEVER COMES FIRST"

11. **Page 53, Section 16.0, Ethical and Regulatory Considerations**: The following new section regarding the confidentiality of this study has been added:

    "*<u>Confidentiality</u>*

    Please note that the information contained in this protocol is considered confidential and should not be used or shared beyond the purposes of completing protocol requirements until or unless additional permission is obtained."

12. **Pages 53-56, Section 16.1, Adverse Event Reporting Requirements**: The following changes have been made to this section:
    - References to the Adverse Event Expedited Reporting System (AdEERS) have been changed to the CTEP Adverse Event Reporting System (CTEP-AERS) in this section and in Section 8.9 (page 35).
    - Page 53, Section 16.1b: The following sentence has been added at the end of the reporting method instruction: "*A CTEP-AERS report must be submitted to the SWOG Operations Office electronically via the CTEP-AERS Web-based application located at: <u>http://ctep.cancer.gov/protocolDevelopment/electronic_applications/adverse_events.htm</u>*"
    - Page 55, Section 16.1g: The definition of 'second malignancy' has been added, and the website link for accessing the adverse event reporting requirements has been updated.

13. **Pages 56-57, Section 16.1h, Adverse Event Reporting Requirements**: A new section regarding when to report pregnancy, fetal death, and death neonatal has been included.

14. **Page 59, Section 17.0, Bibliography**: The everolimus investigator brochure reference, item #17, has been updated.

SWOG Authorized Document

15. **Page 62, Section 18.2, Emergency Unblinding Guidelines**: The following note regarding unblinding requests has been added under the general consideration section:

    "*PLEASE NOTE: Unblinding requests for disease recurrence MUST be handled by the planned unblinding instructions outlined in Section 7.5. Do not contact the Washington Poison Center with these requests*."

16. **Page 66, Section 18.4, Examples of Clinically Relevant Drug Interactions: Substrates, Inducers and Inhibitors of the Isoenzyme CYP3A4**: The following instructions regarding the use of CYP3A4 inducers and inhibitors while on protocol treatment has been added:

    "*Only the strong CYP3A4 inducers and inhibitors listed in Section 5.2e are prohibited during study treatment. Refer to Sections 7.1c and 7.3 for guidance regarding use of moderate CYP3A4 inducers and inhibitors while on treatment*."

17. **Page 68, Section 18.4, Examples of Clinically Relevant Drug Interactions: Substrates, Inducers and Inhibitors of the Isoenzyme CYP3A4**: 'Study agent' has been changed to '*everolimus*' throughout the first paragraph.

The model consent form has been revised as follows:

1. Page 4: Under 'During the study', the following editorial changes have been made to provide clarity:
    - First bullet: 'History and physical exam at Week 4' has been changed to 'History and physical exam *at the beginning of Week 1* and Week 4 *(Note: If you receive a prestudy history and physical exam within 28 days prior to starting on this study, then these tests do not need to be repeated during Week 1*).
    - Second, third, and fourth bullets: '…every six weeks…' has been changed to '*at the beginning of each 6-week cycle (Week 7, 13, 20, etc.) for a maximum of 9 cycles'.*

2. Page 4: In the second paragraph, the last sentence has been updated since adverse effects can occur with placebo pills:

    Original: "These pills will look just like the pills containing everolimus, however, there should not be any side effects."

    New: "These pills will look just like the pills containing everolimus~~, however, there should not be any side effects~~."

3. Pages 6-7: Under 'What side effects or risks can I expect from being in the study?', the everolimus/side effect list has been replaced with the new condensed risk profile table. Specifically, risk attributions that have changed include the following:

    - Added to 'common, some may be serious': **Weight loss; infections; anemia, which may require blood transfusions; altered taste; headache; nosebleed; inflammation of the lungs**
    - Added to 'occasional, some may be serious': **inability to taste; flushing, fever, reddening of skin; kidney failure; frequent urination; abnormal or loss of menstrual period; bleeding; diabetes; nail disorder**
    - Added to 'rare, and serious': **blood clot which may cause swelling pain, shortness of breath; lengthy wound healing; kidney damage which may cause swelling, may require dialysis; coughing or splitting up blood from the lungs or respiratory tract**

SWOG Authorized Document

Note: The condensed risk profile table has been created in an effort to increase patient understanding of protocol treatment-related side effects. Risks which the patient would not otherwise notice such as changes in laboratory values have been removed. In simplifying the risk language, similar risks which may have been listed separately before have now been combined or removed if considered duplicative.

Institutions **must** update their local consent forms to include the changes to the Model Consent Form **within 90 days** of distribution of this notice.

SWOG considers that the Model Consent Form changes **do not** represent an alteration in risk/benefit ratio. Therefore, local accrual does **not** need to be suspended pending implementation of these changes.

Patients currently receiving everolimus/placebo and patients who sign a consent form prior to local implementation of the consent form changes **must** be informed of the following changes. The manner by which this notification takes place is at the discretion of the local institution. At a minimum, the patient must be notified by the next visit and this notification process must be documented in the patient chart.

Due to extensive repagination, an entire replacement protocol is attached. Please discard any previous versions of the protocol, and attach this memorandum to the front of your copy of S0931.

This memorandum serves to notify the NCI and the SWOG Statistical Center.

cc: PROTOCOL & INFORMATION OFFICE
Cathy M. Tangen, Dr.P.H.
Hongli Li, M.S.
Jean Barce
Austin Hamm
Brian Zeller
Patrice Rine – Novartis
Heather Campbell, Pharm.D. – VACSP
Sharon Jenkins - VACSP

SWOG Authorized Document

SWOG
Leading cancer research. Together.

GROUP CHAIR'S OFFICE
Charles D. Blanke, MD
CHAIR
3181 SW Sam Jackson Pk Rd
MC: L586
Portland, OR 97239
503-494-5586
503-346-8038 FAX

OPERATIONS OFFICE
4201 Medical Dr
Suite 250
San Antonio, TX 78229
210-614-8808
210-614-0006 FAX

STATISTICAL CENTER
1730 Minor Ave
Suite 1900
Seattle, WA 98101
206-652-2267
206-342-1616 FAX

1100 Fairview Ave North
M3-C102
PO Box 19024
Seattle, WA 98109
206-667-4623
206-667-4408 FAX

swog.org

May 15, 2014

TO: ALL NATIONAL CLINICAL TRIALS NETWORK (NCTN) MEMBERS

FROM: SWOG Operations Office

RE: IND Safety Report for Everolimus

MEMORANDUM

IRB Review Requirements

( ) Full board review required. Reason:
- ( ) Initial activation (should your institution choose to participate)
- ( ) Increased risk to patient
- ( ) Complete study redesign
- ( ) Addition of tissue banking requirements
- ( ) Study closure due to new risk information

(√) Expedited review allowed

( ) No review required

MEMORANDUM

The following new safety report has been posted regarding an adverse event that occurred in association with the drug everolimus. Please access this safety report via the study's abstract page or the safety report link on the SWOG website (https://swog.org/safetyreports/safetyreports.asp).

This safety report pertains to the following studies:	Report:
**S0528** Early Therapeutics<br>**S0931** Genitourinary<br>**S1207** Breast<br>**S1222** Breast	Apr. 16, 2014 Mfr Rpt #PHHO2012FR013807

Protocol amendments are not necessary at this time, but your consent form may be revised to include the information provided in this report if it is deemed necessary by your institution. Please append this notice and this report to your copy of the protocol and forward a copy to your Institutional Review Board (IRB) as required by your local policies and procedures. Should any further information regarding this adverse event be made available, it will be forwarded to you.

This memorandum serves to notify the NCI and the SWOG Statistical Center.

cc:
PROTOCOL & INFORMATION OFFICE
Megan Othus, Ph.D.
Cathy M. Tangen, Dr.P.H.
William Barlow, Ph.D.
Danika Lew, M.S.
Hongli Li, M.S.
Rachel Sexton, M.S.
Jean Barce
Carol French – AstraZeneca
John Parnell – AstraZeneca
Jean Sowa – AstraZeneca
Kathleen DeRose – GSK
Scott Adams – Janssen
Rebecca De Los Rios – Novartis
Patrice Rine - Novartis
Kristin White – Novartis

SWOG Authorized Document

Jennie Barrett	Heather Campbell, PharmD – VACSP/PCC
Austin Hamm	Anne Davis – VACSP/PCC
Larry Kaye	Michael Moody – VACSP/PCC
Laura Kingsbury, M.R.T.	Sharon Jenkins – VACSP/PCC
Christine McLeod	James Rasp – VACSP/PCC
Iris Syquia	Emily Tabinski – VACSP/PCC
Brian Zeller	Katie VonDerau – WPC

SWOG Authorized Document

SWOG
Leading cancer research. Together.

GROUP CHAIR'S OFFICE
Charles D. Blanke, MD
CHAIR
3181 SW Sam Jackson Pk Rd
MC: L586
Portland, OR 97239
503-494-5586
503-346-8038 FAX

OPERATIONS OFFICE
4201 Medical Dr
Suite 250
San Antonio, TX 78229
210-614-8808
210-614-0006 FAX

STATISTICAL CENTER
1730 Minor Ave
Suite 1900
Seattle, WA 98101
206-652-2267
206-342-1616 FAX

1100 Fairview Ave North
M3-C102
PO Box 19024
Seattle, WA 98109
206-667-4623
206-667-4408 FAX

swog.org

April 15, 2014

TO: ALL NATIONAL CLINICAL TRIALS NETWORK (NCTN) MEMBERS

FROM: SWOG Operations Office

RE: IND Safety Reports for Everolimus

MEMORANDUM

IRB Review Requirements

( ) Full board review required. Reason:
- ( ) Initial activation (should your institution choose to participate)
- ( ) Increased risk to patient
- ( ) Complete study redesign
- ( ) Addition of tissue banking requirements
- ( ) Study closure due to new risk information

(√) Expedited review allowed

( ) No review required

MEMORANDUM

The following new safety reports have been posted regarding adverse events that occurred in association with the drug everolimus. Please access these safety reports via the study's abstract page or the safety report link on the SWOG website (https://swog.org/safetyreports/safetyreports.asp).

These safety reports pertain to the following studies:

S0528 Early Therapeutics
S0722 Lung
S0931 Genitourinary
S1207 Breast
S1222 Breast

Reports:

Mar. 22, 2013	Mfr Rpt #PHHO2013SE003791
Apr. 10, 2013	Mfr Rpt #PHHO2013BR004674
Apr. 10, 2013	Mfr Rpt #PHHO2013BR004675
Apr. 10, 2013	Mfr Rpt #PHHO2013BR004769
Apr. 22, 2013	Mfr Rpt #PHHO2013AU000493
May 7, 2013	Mfr Rpt #PHHO2013FR005718
May 8, 2013	Mfr Rpt #PHHO2013US005955
May 22, 2013	Mfr Rpt #PHHO2013DE003478 FU
Jun. 18, 2013	Mfr Rpt #PHHO2013US007940
Jun. 18, 2013	Mfr Rpt #PHHO2013FR005541
Jun. 19, 2013	Mfr Rpt #PHHO2012BE001726
Jun. 28, 2013	Mfr Rpt #PHHO2013US007122 FU
Jul. 25, 2013	Mfr Rpt #PHHO2013DE004079 FU
Sep. 11, 2013	Mfr Rpt #PHHO2013BR008407
Nov. 5, 2013	Mfr Rpt #PHHO2013DE008522
Dec. 6, 2013	Mfr Rpt #PHHO2013FR015441
Dec. 27, 2013	Mfr Rpt #PHHO2013FR016460 FU
Dec. 27, 2013	Mfr Rpt #PHHO2013FR016402 FU
Mar. 5, 2014	Mfr Rpt #PHHO2014CN001000
Mar. 10, 2014	Mfr Rpt #PHHO2014AT002913

SWOG Authorized Document

Protocol amendments are not necessary at this time, but your consent form may be revised to include the information provided in these reports if it is deemed necessary by your institution. Please append this notice and these reports to your copy of the protocol and forward copies to your Institutional Review Board (IRB) as required by your local policies and procedures. Should any further information regarding these adverse events be made available, it will be forwarded to you.

This memorandum serves to notify the NCI and the SWOG Statistical Center.

cc:
PROTOCOL & INFORMATION OFFICE
Antje Hoering, Ph.D.
Mary Redman, Ph.D.
Cathy M. Tangen, Dr.P.H.
William Barlow, Ph.D.
Danika Lew, M.S.
Hongli Li, M.S.
James Moon, M.S.
Rachel Sexton, M.S.
Jean Barce
Jennie Barrett
Vicki Green
Austin Hamm
Larry Kaye
Laura Kingsbury, M.R.T.
Christine McLeod
Iris Syquia
Brian Zeller
Carol French – AstraZeneca
John Parnell – AstraZeneca
Jean Sowa – AstraZeneca
Scott Adams - Janssen
Rebecca De Los Rios – Novartis
Patrice Rine - Novartis
Kristin White – Novartis
Kathleen DeRose – GSK
Heather Campbell, PharmD – VACSP/PCC
Anne Davis – VACSP/PCC
James Rasp – VACSP/PCC
Sharon Jenkins – VACSP/PCC
Katie VonDereau - WWPC

SWOG Authorized Document

SWOG
Leading cancer research. Together.

January 1, 2013

GROUP CHAIR'S OFFICE
Laurence H. Baker, DO
CHAIR
24 Frank Lloyd Wright Dr
PO Box 483
Ann Arbor, MI 48106
734-998-7130
734-998-7118 FAX

OPERATIONS OFFICE
4201 Medical Dr
Suite 250
San Antonio, T X 78229
210-614-8808
210-614-0006 FAX

STATISTICAL CENTER
1730 Minor Ave
Suite 1900
Seattle, WA 98101
206-652-2267
206-342-1616 FAX

1100 Fairview Ave North
M3-C102
PO Box 19024
Seattle, WA 98109
206-667-4623
206-667-4408 FAX

swog.org

TO: ALL SWOG MEMBER, CCOP AND AFFILIATE MEDICAL INVESTIGATORS AND CLINICAL RESEARCH ASSOCIATES

FROM: SWOG Operations Office

RE: Eligibility Affirmation

MEMORANDUM

By signing the FDA 1572, every SWOG investigator has agreed to conduct studies in compliance with the protocol, and to personally conduct or supervise the investigation. A critical step in this process is verification of patient eligibility.

Effective January 1st, 2013, every registering investigator or another SWOG investigator designate is required to sign a statement on the Registration Worksheet that the eligibility criteria have been confirmed. This worksheet will not be submitted to Data Operations Office but must be maintained at the local institution for review during audits.

As part of this transition, forms and the forms list (Section 18.2) are being removed from active studies and will be posted separately on the individual protocol abstract page for each study. Subsequent pages have been renumbered accordingly. No other form, protocol, or consent form changes have been made as part of the transition.

If you have any questions, please contact the SWOG Operations Office at 210/614-8808.

SWOG
Leading cancer research. Together.

GROUP CHAIR'S OFFICE
Laurence H. Baker, DO
CHAIR
24 Frank Lloyd Wright Dr
PO Box 483
Ann Arbor, MI 48106
734-998-7130
734-998-7118 FAX

OPERATIONS OFFICE
4201 Medical Dr
Suite 250
San Antonio, TX 78229
210-614-8808
210-614-0006 FAX

STATISTICAL CENTER
1730 Minor Ave
Suite 1900
Seattle, WA 98101
206-652-2267
206-342-1616 FAX

1100 Fairview Ave North
M3-C102
PO Box 19024
Seattle, WA 98109
206-667-4623
206-667-4408 FAX

swog.org

December 1, 2012

TO: ALL SWOG MEMBER, CCOP AND AFFILIATE MEDICAL ONCOLOGISTS

FROM: SWOG Operations Office

RE: IND Safety Reports for Everolimus

MEMORANDUM

IRB Review Requirements

( ) Full board review required. Reason:
- ( ) Initial activation (should your institution choose to participate)
- ( ) Increased risk to patient
- ( ) Complete study redesign
- ( ) Addition of tissue banking requirements
- ( ) Study closure due to new risk information

(√) Expedited review allowed

( ) No review required

MEMORANDUM

The following new safety reports have been posted regarding adverse events that occurred in association with the drug everolimus. Please access these safety reports via the study's abstract page or the safety report link on the SWOG website (https://swog.org/safetyreports/safetyreports.asp).

These safety reports pertain to the following studies:	Reports:	
**S0528** Early Therapeutics	Sep. 19, 2012	Mfr Rpt #PHHO2012IT013460
**S0722** Lung	Oct. 18, 2012	Mfr Rpt #PHHO2012JP014859
**S0931** Genitourinary	Oct. 30, 2012	Mfr Rpt #PHHO2012DE015384
	Oct. 30, 2012	Mfr Rpt #PHHO2012DE015612
	Nov. 1, 2012	Mfr Rpt #PHHO2012GB015729
	Nov. 7, 2012	Mfr Rpt #PHHO2012DE005484
	Nov. 12, 2012	Mfr Rpt #PHHO2012DE015384 FU

Protocol amendments are not necessary at this time, but your consent form may be revised to include the information provided in these reports if it is deemed necessary by your institution. Please append this notice and these reports to your copy of the protocol and forward copies to your Institutional Review Board (IRB) as required by your local policies and procedures. Should any further information regarding these adverse events be made available, it will be forwarded to you.

This memorandum serves to notify the NCI and SWOG Statistical Center.

cc: PROTOCOL & INFORMATION OFFICE
Antje Hoering, Ph.D.
Mary Redman, Ph.D.
Cathy M. Tangen, Dr.P.H.
Hongli Li, M.S.
James Moon, M.S.
Rachel Sexton, M.S.
Jean Barce
Vicki Green
Austin Hamm
Larry Kaye
Laura Kingsbury, M.R.T.
Christine McLeod
Brian Zeller
Rebecca De Los Rios – Novartis
Kristin White – Novartis
Robert A. Friedman, BSc, CCRA – GSK
Heather Campbell, PharmD - VACSP
Sharon Jenkins - VACSP

SWOG Authorized Document

SWOG
Leading cancer research. Together.

GROUP CHAIR'S OFFICE
Laurence H. Baker, DO
CHAIR
24 Frank Lloyd Wright Dr
PO Box 483
Ann Arbor, MI 48106
734-998-7130
734-998-7118 FAX

OPERATIONS OFFICE
4201 Medical Dr
Suite 250
San Antonio, T X 78229
210-614-8808
210-614-0006 FAX

STATISTICAL CENTER
1730 Minor Ave
Suite 1900
Seattle, WA 98101
206-652-2267
206-342-1616 FAX

1100 Fairview Ave North
M3-C102
PO Box 19024
Seattle, WA 98109
206-667-4623
206-667-4408 FAX

swog.org

Distribution Date: October 15, 2012
CTEP Submission Date: September 26, 2012

TO: ALL SWOG MEMBER, CCOP, AND AFFILIATE MEDICAL ONCOLOGISTS; CTSU-**U.S. INSTITUTIONS ONLY**

FROM: Gilbert R. Carrizales, M.S., Protocol Coordinator

RE: **S0931**, "EVEREST: EVErolimus for Renal Cancer Ensuing Surgical Therapy, A Phase III Study. Study Coordinators: Drs. C.W. Ryan, E.I. Heath, P.N. Lara, G.S. Palapattu and P.C. Mack.

REVISION #2

Study Coordinator: Christopher W. Ryan, M.D.
Phone number: 503/494-8487
E-mail: ryanc@ohsu.edu

IRB Review Requirements

( ) Full board review required. Reason:
- ( ) Initial activation (should your institution choose to participate)
- ( ) Increased risk to patient
- ( ) Complete study redesign
- ( ) Addition of tissue banking requirements
- ( ) Study closure due to new risk information

(√) Expedited review allowed

( ) No review required

REVISION #2

The study referenced above has been revised as follows:

Face Page: The version date of the protocol has been updated.

Section 5.4, page 15: This section has been revised to allow non-contrast CT scans of the chest/abdomen/pelvis if in the opinion of the investigator it is in the best medical interest of the patient.

Section 9.0, page 29: The "Ӂ" footnote has been revised to be consistent with the change made in Section 5.4 of the protocol.

Please attach this memorandum to the front of your protocol and insert the replacement pages referenced above.

This memorandum serves to notify the NCI and SWOG Statistical Center.

cc: PROTOCOL & INFORMATION OFFICE
Cathy M. Tangen, Dr.P.H.
Hongli Li, M.S.
Jean Barce
Austin Hamm
Brian Zeller
Rebecca De Los Rios – Novartis
Heather Campbell, Pharm. D. – VACSP
Sharon Jenkins - VACSP

SWOG Authorized Document

SWOG
Leading cancer research. Together.

GROUP CHAIR'S OFFICE
Laurence H. Baker, DO
CHAIR
24 Frank Lloyd Wright Dr
PO Box 483
Ann Arbor, MI 48106
734-998-7130
734-998-7118 FAX

OPERATIONS OFFICE
4201 Medical Dr
Suite 250
San Antonio, T X 78229
210-614-8808
210-614-0006 FAX

STATISTICAL CENTER
1730 Minor Ave
Suite 1900
Seattle, WA 98101
206-652-2267
206-342-1616 FAX

1100 Fairview Ave North
M3-C102
PO Box 19024
Seattle, WA 98109
206-667-4623
206-667-4408 FAX

swog.org

Distribution Date: August 15, 2012
CTEP Submission Date: July 11, 2012

TO: ALL SWOG MEMBER, CCOP, AND AFFILIATE MEDICAL ONCOLOGISTS; CTSU-**U.S. INSTITUTIONS ONLY**

FROM: Jennifer I. Scott, Protocol Coordinator

RE: **S0931**, "EVEREST: EVErolimus for Renal Cancer Ensuing Surgical Therapy, A Phase III Study. Study Coordinators: Drs. C.W. Ryan, E.I. Heath, P.N. Lara, G.S. Palapattu and P.C. Mack.

REVISION #1

Study Coordinator: Christopher W. Ryan, M.D.
Phone number: 503/494-8487
E-mail: ryanc@ohsu.edu

IRB Review Requirements

( ) Full board review required. Reason:
- ( ) Initial activation (should your institution choose to participate)
- ( ) Increased risk to patient
- ( ) Complete study redesign
- ( ) Addition of tissue banking requirements
- ( ) Study closure due to new risk information

(√) Expedited review allowed

( ) No review required

REVISION #1

Institutions **must** update their local consent forms to include the changes to the Model Consent Form.

SWOG considers that the Model Consent Form changes **do not** represent an alteration in risk-benefit ratio. Therefore, local accrual does **not** need to be suspended pending implementation of these changes.

Patients currently receiving everolimus/placebo, and patients who sign a consent form prior to Institutional Review Board (IRB) approval and local implementation of the consent form changes, need not be informed of these changes unless required by the local IRB.

SWOG Authorized Document

The study referenced above has been revised as follows:

Face Page: The participants list has been revised to delete "UCOP" as this program has been discontinued. Benjamin Ely is no longer with the Group. His name and contact information have been replaced with that for Hongli Li, M.S. The version date of the protocol has been updated.

Page 2: CALGB has been added as an endorsing cooperative group and Dr. Maxwell Meng has been added as the CALGB Study Coordinator.

Page 3: The third column of the CTSU table has been revised to replace the previous data submission instructions with the current online data submission instructions. The following text has been deleted from the table, "Note: Non-lead group institutions will order the following supplies from the CTSU Operations Office: (if applicable)" since this is not applicable to this study.

Schema, page 4: The "Note" section regarding the procedure for optional unblinding of treatment at recurrence has been added at the bottom of the schema.

Section 1.1, page 5: The text "one year" has been replaced with "54 weeks" to be consistent with the remainder of the protocol.

Sections 1.2c-d, page 5: These sections have been revised to more clearly reflect that specimens will be banked for future study.

Section 2.0, page 8: The blood sampling schedule noted in the last paragraph on this page has been revised to be consistent with the remainder of the protocol.

Section 3.0, pages 9-14: The section regarding the Investigator's Brochure has been added to Section 3.0, page 9. Section 3.1 has been re-formatted by the SWOG Pharmaceutical Committee to be consistent with the Group's current standard format for protocol drug information. This format is similar to the format used by CTEP for protocols utilizing CTEP-supplied agents.

Section 4.0, pages 14a-b: Pages 14a and b were added to prevent extensive repagination of the remainder of the protocol. The T1 definition was added. An end parenthesis mark and period were added to the T4 definition. The N1 and pT3a definitions were revised to be consistent with the AJCC Seventh Edition, 2010.

Section 5.0, page 15: The form number for the **S0931** Prestudy Form has been revised in Section 5.0.

Section 5.10, page 16: The word "strong" has been added to clarify CYP3A4 inducers. The parenthetical sentence at the end of this section has been revised for clarification and the cross-reference to Section 7.1 corrected.

Section 5.17, page 16: The text "DNA", "RNA" and "PCR" have been removed from the third sentence of this section. The text "9.0 for testing information and Section" has been added to the last sentence of this section.

SWOG Authorized Document

Sections 5.19 & 5.20, page 16: Section 5.19 has been deleted. Sections 7.1a and b have been moved to become Sections 5.19 and 5.20 respectively. The words “should” have been replaced with “must” as these are now eligibility requirements and the timeframe of within 28 days prior to registration has been added. The remainder of Section 5.0 has been renumbered accordingly.

Section 5.25 (formerly Section 5.24), page 17: This section has been revised to be consistent with the Group’s current standard language regarding reproductive potential.

Section 5.27 (formerly Section 5.26), page 17: This section has been revised to reflect the Group’s current standard language with regards to OPEN registration requirements.

Sections 7.1a-b, pages 18-19: These two sections have been moved to Section 5.0 as Section 5.20 and 5.21 respectively. The remainder of Section 7.1 has been renumbered accordingly.

Section 7.1c, page 19: This section has been revised to replace the text “carried out with” with “avoided if possible, or used subject to”.

Section 7.1d, page 19: This section has been added to indicate that alkaline phosphatase should be obtained to be consistent with the study calendar.

Section 7.3, page 20: The bullet point regarding drugs known to be inhibitors or inducers of CYP3A4 has been revised to delineate between drugs known to be strong inhibitors versus drugs or substances known to be moderate inhibitors or inducers. Drugs known to be strong inhibitors or inducers must not be administered whereas drugs or substances known to be moderate inhibitors or inducers of CYP3A should be avoided or used subject to caution. A reference has been added with regards to inhibitors of P-glycoprotein to Section 19.4. The Treatment Form number has been updated.

Section 7.4, page 20: This section has been revised to reflect the Group’s standard language regarding the Intake Calendar.

Section 7.5, pages 20-20b: A new section 7.5 has been added to include procedures for unblinding patients who have recurred (per Section 10.1). Emergency unblinding instructions have not changed and are still outlined in Appendix 19.2. Pages 20a and 20b were added to prevent extensive repagination of the remainder of the protocol. The remainder of Section 7.0 has been renumbered accordingly.

Section 7.6a, page 20b: The text “if the patient has documented recurrence as defined in Section 10.1, unblinding of study treatment as outlined in Section 7.5 may be requested” has been added.

Section 8.4, page 23: The word "days" was added to the first sentence.

Section 9.0 (Study Calendar), page 29: A cross-reference to the Scan footnote has been added to the Recurrence Assessment line. The scan footnote has been revised to be consistent with the protocol. An additional sentence has been added to this footnote regarding imaging intervals. “X’s” have been removed from the Week 18 column as these tests do not have to be obtained specifically on Week 18, but rather repeated prior to beginning each cycle. The cross references to protocol sections have been corrected in the first and third footnotes. The footnote regarding assessment of hepatitis B/C medical history has been revised to be consistent with changes made to the protocol.

SWOG Authorized Document

Section 13.3, page 34: The acronym OPEN has been defined in the second paragraph of this section.

Section 14.4, page 37: The form number for the **S0931** Prestudy Form has been revised. The form number for the **S0931** Tumor Specimen Processing Form has been updated.

Section 14.6, page 37: The text “WITHIN 7 DAYS OF COMPLETION OF EACH” has replaced "AFTER EVERY" in the header of this section. The form number for the **S0931** Treatment Form has been updated in this section.

Sections 14.8, 14.10 and 14.11, page 38: The form number for the **S0931** Treatment Form has been updated in these sections.

Section 15.0, pages 39-39c: This section has been updated to provide clearer specimen submission guidelines consistent with the Group's current standard format for specimen submission instructions. The types of specimens collected, the frequency at which they are collected, and how they are collected has not changed. Pages 39a-39c have been added to prevent extensive repagination of the remainder of the protocol.

Section 16.1 pages 40-43: These sections have been revised to reflect the Group’s current SAE guidelines. Page 43 was intentionally left blank to prevent extensive repagination of the protocol.

Section 18.0, Master Forms, pages 46, 63-66 and 70: The **S0931** Prestudy Form has been revised to remove the two parenthetical sections noted with pT3a. The form number for the **S0931** Prestudy Form has been revised from Form #6723 to Form # 7940. Page 1 of the **S0931** Treatment Form has been revised to add an agent dose modification code of “10 = dose missed”, an additional box under “Modifications” and the “*” note at the bottom. The form number for the **S0931** Treatment Form has been revised from Form # 44488 to Form #26931. The **S0931** Tumor Specimen Processing Form has been revised to include a box for unknown under duration of fixation. The form number for the **S0931** Tumor Specimen Processing form has been revised from Form #55588 to Form # 61976.

Model Informed Consent, pages 50-58a: The paragraph mid-way on page 50 beginning with “You will take two pills once a day” has been revised to include the text “and no more than a light fat-free meal.” The following sentence regarding taking the study drug on an empty stomach has been deleted. A paragraph has been added to page 50 prior to the section titled “How long will I be in the study?” to include information about unblinding after recurrence. The list of risks found on pages 51-53 has been revised to be consistent with the changes made to Section 3.0 of the protocol. Specifically: “Infections” has been moved from “Likely” and is now more specifically listed in “Less Likely” as “Urinary tract infections (these could be serious)” and “Infections in your lungs (these could be serious)”; “Renal failure”, “Bleeding”, and “Congestive cardiac failure” have been moved from “Less Likely” to a new “Rare, but Serious” section; “Allergic reaction” has also been added to “Rare, but Serious” ; “Lowered numbers of lymph cells in the blood” has been added to “Likely”; “Slower wound healing”; “Sinus inflammation”, “Back pain” and “Muscle cramps” have been added to “Less Likely.” Page 52a was added to prevent extensive repagination of the protocol. A paragraph has been added to page 54 indicating that a description of the trial is available on ClinicalTrials.gov. A paragraph has been added below item 2 on page 55 to include the text “If a problem with getting everolimus or placebo occurs, your study doctor will talk to you about these options.” The two italicized paragraphs on page 56 have been deleted as these

SWOG Authorized Document

are instructional for the writer of the model informed consent. The "Consent Form for Use of Specimens" (pages 57-58) has been revised. The paragraph beginning "We would like to keep…" has been moved to the top of the consent. The text "of the" and "that are left over" have been deleted from the first sentence of this section. The text "sent to an outside lab for research purposes" has been replaced with the text "banked for future research" as specimens currently collected on this trial will be banked until funding is obtained. The last five sentences of this paragraph have been deleted because they are redundant. The section including the address of the former repository in Colorado has been deleted. Two paragraphs have been added to the "Things to Think About" section on page 58 to include information about the Genetic Information Nondiscrimination Act of 2008. Page 58a was added to prevent repagination of the remainder of the protocol.

Section 19.3, pages 78-79: This section has been updated with the Group's current standard instructions regarding the determination of expedited adverse event reporting requirements.

Please attach this memorandum to the front of your protocol and insert the replacement pages referenced above.

This memorandum serves to notify the NCI and SWOG Statistical Center.

cc: PROTOCOL & INFORMATION OFFICE
Cathy M. Tangen, Dr.P.H.
Hongli Li, M.S.
Jean Barce
Austin Hamm
Brian Zeller
Rebecca De Los Rios – Novartis
Heather Campbell, Pharm. D. – VACSP
Sharon Jenkins - VACSP

SWOG
Leading cancer research. Together.

GROUP CHAIR'S OFFICE
Laurence H. Baker, DO
CHAIR
24 Frank Lloyd Wright Dr
PO Box 483
Ann Arbor, MI 48106
734-998-7130
734-998-7118 FAX

OPERATIONS OFFICE
4201 Medical Dr
Suite 250
San Antonio, TX 78229
210-614-8808
210-614-0006 FAX

STATISTICAL CENTER
1730 Minor Ave
Suite 1900
Seattle, WA 98101
206-652-2267
206-342-1616 FAX

1100 Fairview Ave North
M3-C102
PO Box 19024
Seattle, WA 98109
206-667-4623
206-667-4408 FAX

swog.org

April 1, 2012

TO: ALL SWOG MEMBER, CCOP AND AFFILIATE MEDICAL ONCOLOGISTS

FROM: SWOG Operations Office

RE: IND Safety Report for Everolimus

MEMORANDUM

IRB Review Requirements

( ) Full board review required. Reason:
- ( ) Initial activation (should your institution choose to participate)
- ( ) Increased risk to patient
- ( ) Complete study redesign
- ( ) Addition of tissue banking requirements
- ( ) Study closure due to new risk information

(√) Expedited review allowed

( ) No review required

MEMORANDUM

The following new safety report has been posted regarding an adverse event that occurred in association with the drug everolimus. Please access this safety report via the study's abstract page or the safety report link on the SWOG website (https://swog.org/safetyreports/safetyreports.asp).

This safety report pertains to the following studies:	Report:
**S0528** Early Therapeutics<br>**S0722** Lung<br>**S0931** Genitourinary	Mar. 19, 2012 AE #1141853

Protocol amendments are not necessary at this time, but your consent form may be revised to include the information provided in this report if it is deemed necessary by your institution. Please append this notice and this report to your copy of the protocol and forward a copy to your Institutional Review Board (IRB) as required by your local policies and procedures. Should any further information regarding this adverse event be made available, it will be forwarded to you.

This memorandum serves to notify the NCI and SWOG Statistical Center.

cc: PROTOCOL & INFORMATION OFFICE
Antje Hoering, Ph.D.
Megan Othus, Ph.D.
Mary Redman, Ph.D.
Cathy M. Tangen, Dr.P.H.
Danika Lew, M.A.
James Moon, M.S.
Rachel Sexton, M.S.
Jean Barce
Vicki Green
Austin Hamm
Larry Kaye
Laura Kingsbury, M.R.T.
Christine McLeod
Brian Zeller
Amy Bartalotta – Novartis
Elizabeth Miles, M.S. – Novartis
Kristin White – Novartis
Robert A. Friedman, BSc, CCRA – GSK
Heather Campbell, PharmD - VACSP
Sharon Jenkins - VACSP

SWOG Authorized Document

SWOG
Leading cancer research. Together.

GROUP CHAIR'S OFFICE
Laurence H. Baker, DO
CHAIR
24 Frank Lloyd Wright Dr
PO Box 483
Ann Arbor, MI 48106
734-998-7130
734-998-7118 FAX

OPERATIONS OFFICE
4201 Medical Dr
Suite 250
San Antonio, TX 78229
210-614-8808
210-614-0006 FAX

STATISTICAL CENTER
1730 Minor Ave
Suite 1900
Seattle, WA 98101
206-652-2267
206-342-1616 FAX

1100 Fairview Ave North
M3-C102
PO Box 19024
Seattle, WA 98109
206-667-4623
206-667-4408 FAX

swog.org

April 1, 2011

TO: ALL SWOG MEMBER, CCOP, UCOP AND AFFILIATE MEDICAL ONCOLOGISTS; CTSU-**U.S. INSTITUTIONS ONLY**

FROM: Jennifer I. Scott, Protocol Coordinator

RE: **S0931**, "EVEREST: EVErolimus for Renal Cancer Ensuing Surgical Therapy, A Phase III Study. Study Coordinators: Drs. C.W. Ryan, E.I. Heath, P.N. Lara, G.S. Palapattu and P.C. Mack.

STATUS NOTICE

Study Coordinator: Christopher W. Ryan, M.D.
Phone number: 503/494-8487
E-mail: ryanc@ohsu.edu

IRB Review Requirements

(√) Full board review required. Reason:
- (√) Initial activation (should your institution choose to participate)
- ( ) Increased risk to patient
- ( ) Complete study redesign
- ( ) Addition of tissue banking requirements
- ( ) Study closure due to new risk information

( ) Expedited review allowed

( ) No review required

ACTIVATION

The study referenced above is now open for participation. The entire protocol is attached for your use.

This memorandum serves to notify the NCI and Southwest Oncology Group Statistical Center.

cc: PROTOCOL & INFORMATION OFFICE
Cathy M. Tangen, Dr.P.H.
Benjamin W. Ely, M.S.
Bryan Goldman, M.S.
Jean Barce
Janice Leaman
Brian Zeller
Amy Bartalotta – Novartis
Heather Campbell, Pharm. D. – VACSP
Sharon Jenkins - VACSP

SWOG Authorized Document

PRIVILEGED COMMUNICATION
FOR INVESTIGATIONAL USE ONLY

Activated April 1, 2011

SWOG

EVEREST: EVErolimus for Renal Cancer Ensuing Surgical Therapy, A Phase III Study

NCT #01120249

STUDY CHAIRS:

Christopher W. Ryan, M.D. (Medical Oncology)
Oregon Health & Science University
3303 SW Bond Avenue,
CR14
Portland, OR 97239
Phone: 503/494-8487
FAX: 503/494-6197
E-mail: ryanc@ohsu.edu

Elisabeth I. Heath, M.D. (Medical Oncology)
Wayne State University
Karmanos Cancer Institute
4100 John R
4 HWCRC
Detroit, MI 48201
Phone: 313/576-8715
FAX: 313/576-8767
E-mail: heathe@karmanos.org

Primo N. Lara, Jr., M.D. (Medical Oncology
& Translational Medicine)
UC Davis Cancer Center
4501 X Street
Sacramento, CA 95817
Phone: 916/734-3771
FAX: 916/734-7946
E-mail: pnlara@ucdavis.edu

AGENTS:

SWOG-held IND Agent:
Everolimus (RAD001, Afinitor®, Zortress®)

(NSC- 733504) (IND-75,093)

Ganesh S. Palapattu, M.D. (Urology)
The Methodist Hospital
6560 Fannin Street, Suite 2100
Houston, TX 77030
Phone: 713/441-8137
FAX: 713/441-6463
E-mail: gpalapattu@tmhs.org

Philip C. Mack, Ph.D. (Translational Medicine)
UC Davis Cancer Center
Division of Hematology/Oncology
4501 X Street
Sacramento, CA 95817
Phone: 916/734-8022
FAX: 916/734-2361
E-mail: pcmack@ucdavis.edu

BIOSTATISTICIANS:

Cathy M. Tangen, Dr.P.H. (Biostatistics)
Melissa Plets, M.S.
SWOG Statistical Center
Fred Hutchinson Cancer Research Center
1100 Fairview Avenue North, M3-C102
P.O. Box 19024
Seattle, WA 98109-1024
Phone: 206/667-4623
FAX: 206/667-4408
E-mail: ctangen@fredhutch.org
E-mail: mplets@fredhutch.org

ALLIANCE STUDY CHAIR:

Maxwell V. Meng, M.D.
University of California at San Francisco –
Mount Zion
E-mail: mmeng@urology.ucsf.edu

ECOG-ACRIN STUDY CHAIR:

Mark Stein, M.D.
The Cancer Institute of New Jersey
E-mail: mark.stein@rutgers.edu

SWOG Authorized Document

PARTICIPANTS

ALL NATIONAL CLINICAL TRIAL NETWORK (NCTN) MEMBERS – U.S. INSTITUTIONS ONLY

SWOG/SWOG

ALLIANCE/Alliance for Clinical Trials in Oncology

ECOG-ACRIN/ECOG-ACRIN Cancer Research Group

NRG/NRG Oncology

SWOG Authorized Document

TABLE OF CONTENTS

TITLE 1
PARTICIPANTS 2
TABLE OF CONTENTS 3
CANCER TRIALS SUPPORT UNIT (CTSU) ADDRESS AND CONTACT INFORMATION 5
SCHEMA 6
1.0 OBJECTIVES 7
1.1 Primary Objective 7
1.2 Secondary Objectives 7
1.3 Translational Medicine Objectives 7
2.0 BACKGROUND 7
3.0 DRUG INFORMATION 11
3.1 Everolimus (RAD001, Afinitor®, Zortress®) (NSC- 733504) (IND-75,093) 11
4.0 STAGING CRITERIA 18
5.0 ELIGIBILITY CRITERIA 19
5.1 Disease Related Criteria 20
5.2 Prior Therapy Criteria 20
5.3 Clinical/Laboratory Criteria 21
5.4 Translational Medicine Criteria 22
5.5 Regulatory Criteria 22
6.0 STRATIFICATION FACTORS 23
a. Risk group based on pathologic stage (intermediate high-risk vs very high risk) (see Section 4.0), ... 23
b. Histology (clear cell vs non-clear cell); (NOTE: The tumor will be classified as clear cell if any component of clear cell histology is identified in the specimen.) 23
c. Performance status (0 vs 1). 23
7.0 TREATMENT PLAN 23
7.1 Good Medical Practice 23
7.2 Treatment Schedule 24
7.3 Concomitant Therapy 24
7.4 Intake Calendar 25
7.5 Unblinding Procedures 25
7.6 Criteria for Removal from Protocol Treatment 27
7.7 Discontinuation of Treatment 27
7.8 Follow-Up Period 27
8.0 DOSE MODIFICATIONS 27
8.1 NCI Common Terminology Criteria for Adverse Events 27
8.2 General Dose Modification Considerations 27
8.3 Everolimus Dose Levels 28
8.4 Everolimus Dose Modifications 28
8.5 Infection Precaution 32
8.6 Management of Hepatitis Reactivation 32
8.7 Emergency Unblinding 34
8.8 Dose Modification Contacts 34
8.9 Adverse Event Reporting 34
9.0 STUDY CALENDAR 36
10.0 CRITERIA FOR EVALUATION AND ENDPOINT DEFINITIONS 39
10.1 Recurrence 39
10.2 Symptomatic deterioration 39
10.3 Performance Status 40
10.4 Recurrence-Free Survival 40
10.5 Time to Death 40
11.0 STATISTICAL CONSIDERATIONS 40
11.1 Accrual Goal 40
11.2 Analysis of Primary Endpoint 41
11.3 Analysis of Secondary Endpoints 42

11.4 Analysis of Translational Medicine Endpoints 43
11.5 Pharmacokinetic Analysis of Blood Samples 44
11.6 Data and Safety Monitoring Committee Oversight 45
12.0 DISCIPLINE REVIEW **45**
13.0 REGISTRATION GUIDELINES **45**
13.1 Registration Timing 45
13.2 Investigator/Site Requirements 45
13.3 OPEN Registration Requirements 46
13.4 Registration Procedures 47
13.5 Exceptions to SWOG registration policies will not be permitted. 49
14.0 DATA SUBMISSION SCHEDULE **49**
14.1 Data Submission Requirement 49
14.2 Master Forms 49
14.3 Data Submission Procedures 49
14.4 Data Submission Overview and Timepoints 50
15.0 SPECIAL INSTRUCTIONS **51**
15.1 Tissue Specimen Banking 51
15.2 Plasma, Buffy Coat, and Whole Blood Specimen Banking 52
16.0 ETHICAL AND REGULATORY CONSIDERATIONS **55**
16.1 Adverse Event Reporting Requirements 55
17.0 BIBLIOGRAPHY **60**
18.0 APPENDIX **63**
18.1 New York Heart Association Classifications 64
18.2 Emergency Unblinding Guidelines 65
18.3 Examples of Clinically Relevant Drug Interactions: Substrates, Inducers and Inhibitors of the Isoenzyme CYP3A4 67
18.4 Intake Calendar 70

SWOG Authorized Document

CANCER TRIALS SUPPORT UNIT (CTSU) ADDRESS AND CONTACT INFORMATION

<table>
<tr><th>To submit site registration documents:</th><th>For patient enrollments:</th><th>Submit study data directly to the Lead Cooperative Group unless otherwise specified in the protocol:</th></tr>
<tr><td>CTSU Regulatory Office
1818 Market Street,
Suite 1100
Philadelphia, PA19103

Fax: 215-569-0206

Email:
CTSURegulatory@ctsu.coccg.org

For more information, call the CTSU Help Desk at 888-823-5923 or the Regulatory Help Desk at 866-651-CTSU.</td><td>Please refer to the patient enrollment section of the protocol for instructions on using the Oncology Patient Enrollment Network (OPEN) which can be accessed at https://www.ctsu.org/OPEN_SYSTEM/ or https://OPEN.ctsu.org.

Contact the CTSU Help Desk with any OPEN-related questions at ctsucontact@westat.com.</td><td><u>Online Data Submission</u>: Institutions participating through the CTSU are required to submit and amend their data electronically via Online Data Submission. Access the SWOG Workbench using your CTSU User ID and password at the following url:

https://crawb.crab.org/TXWB/ctsulogon.aspx

<u>Exceptions</u>: Data items that are not available for online submission (operative and pathology reports, patient completed forms, scan reports, etc.) may be submitted by fax at 800-892-4007.

Do not submit data forms to CTSU Data Operations.

Do not copy the CTSU on data submission.</td></tr>
<tr><td colspan="3">The most current version of the study protocol and all supporting documents must be downloaded from the protocol-specific Web page of the CTSU Member Web site located at https://www.ctsu.org. Access to the CTSU members' website is managed through the Cancer Therapy and Evaluation Program - Identity and Access Management (CTEP-IAM) registration system and requires user log on with CTEP-IAM username and password.</td></tr>
<tr><td colspan="3"><u>For patient eligibility</u> questions contact the SWOG Data Operations Center by phone or email:

Phone: 206/652-2267
E-mail: guquestion@crab.org

<u>For treatment or toxicity related</u> questions contact the Study PI of the Coordinating Group (Dr. Christopher Ryan at 503/494-8487).</td></tr>
<tr><td colspan="3"><u>For questions unrelated to patient eligibility, treatment, or data</u> submission contact the CTSU Help Desk by phone or e-mail:

CTSU General Information Line: 888-823-5923
E-mail: ctsucontact@westat.com

All calls and correspondence will be triaged to the appropriate CTSU representative.</td></tr>
<tr><td colspan="3"><u>For detailed information on the regulatory and monitoring procedures for CTSU sites</u> please review the CTSU Regulatory and Monitoring Procedures policy located on the CTSU members' website:

https://www.ctsu.org > education and resources tab > CTSU Operations Information >CTSU Regulatory and Monitoring Policy</td></tr>
<tr><td colspan="3">The CTSU website is located at https://www.ctsu.org</td></tr>
</table>

SCHEMA

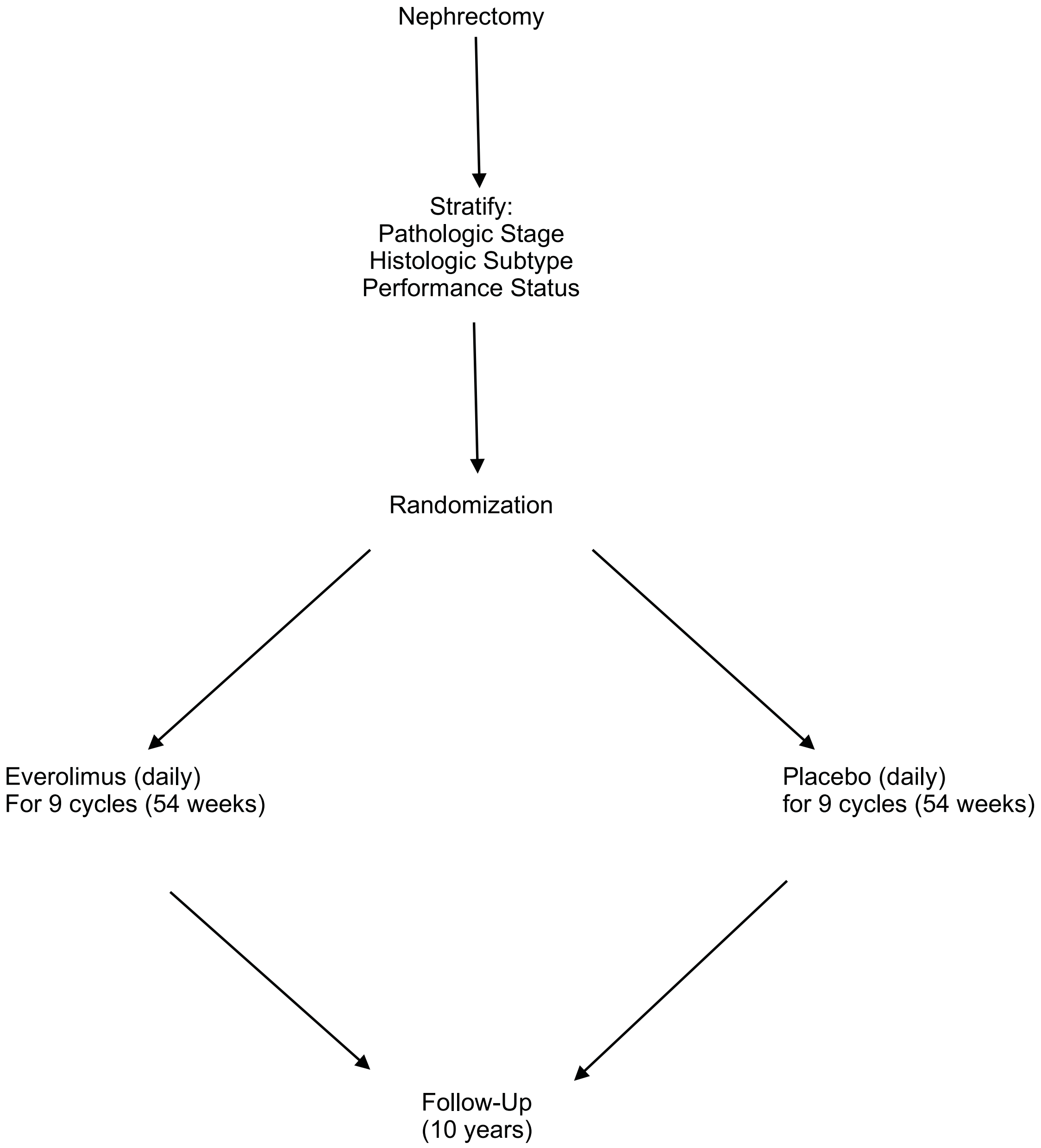

NOTE: Procedure for optional unblinding of treatment at recurrence outlined in Section 7.5.

SWOG Authorized Document

1.0 OBJECTIVES

1.1 Primary Objective

The primary objective of this study is to compare recurrence-free survival in renal carcinoma patients randomly assigned to 54 weeks of everolimus versus 54 weeks of placebo after nephrectomy or partial nephrectomy.

1.2 Secondary Objectives

a. To compare overall survival in those patients randomized to everolimus versus those randomized to placebo.

b. To compare qualitative and quantitative toxicity between the two study arms.

1.3 Translational Medicine Objectives

a. To bank tissue and biologic specimens for future study of molecular biomarkers relevant to the AKT/mTOR and other pathways implicated in the pathogenesis of renal carcinoma and to investigate their potential predictive and prognostic value.

b. To bank blood specimens for the future study of the relationship between steady state trough levels of everolimus and relevant side effects (lymphopenia, infection, hyperglycemia, hypercholesterolemia, hypertriglyceridemia) in patients treated on this study with everolimus.

2.0 BACKGROUND

Nearly 58,000 patients are expected to be diagnosed with kidney cancer in the United States in 2009. *(1)* Despite significant improvements made over the last several years in treatment of advanced kidney cancer, 13,000 patients are expected to die from their disease in 2009. Many of these patients originally present with localized disease and are treated with surgical resection with curative intent. However, up to one-third of patients with fully resected, localized disease will develop either a local or distant recurrence; the majority of whom will succumb to distant metastases. *(2,3)* Despite this significant risk, current standard treatment after resection in these patients is surveillance. Multiple studies investigating interferon or interleukin-2 in the adjuvant setting have failed to demonstrate any benefit over observation alone. *([4],[5],[6],[7])* Studies investigating autologous tumor vaccines have shown equivocal results. *(8,9)* Such first-generation adjuvant studies predated VEGF- and mTOR-directed therapies which have now been established in the treatment of advanced disease based on improved progression-free- and overall-survival. *(10,11,12,13,14,15)*

The Eastern Cooperative Oncology Group **E2805** study (ASSURE) is a second-generation adjuvant study investigating the potential role of the multi-kinase inhibitors sorafenib and sunitinib as adjuvant therapy in the post-operative setting for patients with fully resected localized disease. ASSURE is a randomized Phase III study of one year of sunitinib versus one year of sorafenib versus one year of placebo in patients with intermediate to high-risk M0 renal carcinoma beginning within 12 weeks of nephrectomy. *(16)* This study completed accrual September 1, 2010. It is likely that results will not be available for several years after study closure. Until the results of ASSURE are known, surveillance will remain as standard treatment for such patients. Several additional industry-sponsored, international trials investigating the utility of adjuvant VEGF-R tyrosine kinase inhibitors are currently enrolling, including the SORCE trial (sorafenib), S-TRAC trial (sunitinib), NCT 00492258, NCT 00375674, and NCT 01235962.

ASSURE was designed to study the adjuvant activity of sunitinib and sorafenib, the first two agents to receive FDA approval for advanced renal carcinoma in the post-immunotherapy era, both of

which are VEGF-R tyrosine kinase inhibitors. Inhibitors of mTOR have since emerged as treatment options for advanced renal carcinoma. mTOR is a serine/threonine kinase that recognizes stress signals via the PI3K-AKT pathway and phosphorylates p70 ribosomal S6 kinase 1 (S6K1) and eukaryotic initiation factor 4E binding protein 1 (4EP1). Its role in progression of renal carcinoma may be due to its downstream regulation of hypoxia-inducible factor 1-α. *(17)* Recent Phase III studies have established mTOR inhibition as a clinically relevant treatment strategy for advanced renal carcinoma. In a Phase II trial, temsirolimus, a pro-drug of rapamycin, was shown to improve progression-free and overall-survival in modified Memorial Sloan-Kettering Cancer Center (MSKCC) criteria poor-risk, advanced renal carcinoma patients, and received FDA approval for treatment of the disease. *(18,19)* Everolimus is an orally-administered derivative of rapamycin that was studied in a placebo-controlled Phase III study in patients with advanced renal carcinoma who had received prior therapy with sorafenib, sunitinib, or both. In this study, everolimus improved progression-free survival compared with placebo (hazard ratio 0·30, 95% CI 0·22–0·40, p<0·0001) and has been FDA-approved for the treatment of patients with advanced renal cell carcinoma after failure of treatment with sunitinib or sorafenib. *(20)*

<u>Everolimus</u>

Everolimus is an orally bioavailable macrolide derivative of rapamycin that targets the activity of mTOR kinase and demonstrates potent antiproliferative effects against a variety of tumor types in the pre-clinical setting. *(21)* Everolimus exerts its activity through high affinity interaction with an intracellular receptor protein, the immunophilin FKBP12. The FKBP12/everolimus complex subsequently interacts with the mTOR protein kinase, inhibiting downstream signaling events involved in regulation of the G1 to S-phase transition. The antitumor effect of everolimus may be due to direct inhibition of tumor cell growth and indirectly by inhibiting angiogenesis. The observation of in vivo sensitivity of xenografts comprised of cells demonstrating resistance to everolimus in vitro attributes to the drugs potential to act on the vascular component of the supporting peritumoral stroma. The antiangiogenic property of everolimus confirmed through experiments demonstrating the effect of everolimus in countering VEGF-induced proliferation of human umbilical endothelial cells (HUVEC) in vitro, VEGF-driven angiogenesis in a chamber implant murine model and neovascularization in a murine orthotopic melanoma model.

<u>Clinical Development</u>

Everolimus has been extensively investigated as an immunosuppressant in solid organ transplantation and is approved in renal and cardiac transplantation in many countries. Everolimus has been in development for patients with various hematologic and nonhematologic malignancies both as a single agent or in combination with other antitumor agents. Diseases under study included renal cell carcinoma, breast cancer, carcinoid neuroendocrine tumors, pancreatic islet cell tumors (pNET), hematologic malignancies including chronic myelogenous leukemia (CML), non-Hodgkin's and mantle cell lymphoma, hepatocellular, gastric, colorectal, gastrointestinal stromal tumors (GIST), prostate and others.

Phase I studies of cancer patients with single-agent everolimus have been conducted with both weekly and daily regimens. Dose-limiting toxicities included stomatitis, fatigue, hyperglycemia, and neutropenia. *(22,23)*

A Phase III, randomized, double blind, placebo controlled study in patients with mRCC who progressed on VEGF-R tyrosine kinase inhibitors demonstrated that everolimus administered daily at an oral dose of 10 mg prolonged median progression free survival from 1.87 months for patients receiving placebo to 4.9 months for everolimus treated patients (hazard ratio 0.33, 95% CI 0.25-0.43, p<0·0001). *(24,25)*

Everolimus 5 mg and 10 mg tablets were recently approved under the trade name Afinitor® for patients with advanced renal cell carcinoma (RCC) after failure of treatment with Sutent® (sunitinib) or Nexavar® (sorafenib) in the US, EU and several other countries and is undergoing registration in other regions worldwide.

SWOG Authorized Document

The most common treatment-related adverse effects observed in the renal carcinoma study included stomatitis (40%), rash (40%), fatigue (20%), asthenia (18%), diarrhea (17%), anorexia (16%), and nausea (15%). The most common laboratory abnormalities include anemia (91%), hypercholesterolemia (76%), hypertriglyceridemia (71%), hyperglycemia (50%), increased creatinine (46%), and lymphopenia. (42%). *(26)*

Study Rationale

Given that mTOR inhibition has been shown to improve progression-free survival in two different patient populations of advanced renal carcinoma, it is warranted to study an mTOR inhibitor in the adjuvant setting. Everolimus has the advantage of oral dosing, making it potentially more suitable for adjuvant administration, and has been chosen for this study. The dosing will be 10 mg orally once daily which is the dosing schedule used in the Phase III trial and was the maximum daily dose evaluated in Phase I cancer trials. *(27,28,29)* The control arm will be placebo with a randomized double-blind design, justified by the fact that surveillance is the current standard treatment in this disease setting. We hypothesize that one year of adjuvant treatment with everolimus will improve recurrence-free survival without addition of excessive toxicity.

Accrual to the ASSURE trial was quicker than anticipated with a projected accrual rate of 480 patients per year. However, a high treatment discontinuation rate was noted with approximately 25% of patients on the active arms discontinuing treatment within three months due to adverse events or patient refusal. ASSURE was amended to lower the starting doses of sorafenib and sunitinib in an attempt to ameliorate these effects and to increase the sample size to account for the high dropout rate. Given that everolimus has a different side effect profile than sunitinib or sorafenib, it is quite possible that this agent may be more tolerable in the adjuvant setting and result in better patient adherence. As an example, hand foot syndrome is not a common side effect of mTOR inhibitor therapy, and this particular toxicity has been cited as one of the potential early-onset VEGF-TKI toxicities that have influenced the ASSURE dropout rate. *(30)*

ASSURE employs the UCLA Integrated Staging System (UISS) in determining risk grouping. *(31)* Intermediate-risk, high-risk, and node-positive patients were eligible for ASSURE, based on an analysis of the UCLA database that demonstrated a five year recurrence free rate of 61.8% in the intermediate risk group, 41.9% in the high risk group, and 36% in the node-positive group. *(32)* Although it was expected that 69% of accrued subjects would be intermediate risk and 31% would have high-risk or node positive disease (“very high-risk”), actual accrual numbers reveal that a full 50% of accrued patients are very-high risk. *(33)* We therefore expect a significant proportion of very-high risk patients to be enrolled in this trial, a population in whom mTOR inhibition may theoretically be most relevant given its proven effectiveness in metastatic poor prognosis patients. *(34)* If that proportion of very-high risk patients is not met, closure of lower risk strata will occur to ensure adequate representation. Similarly, given the potential particular activity of mTOR inhibition in non-clear cell histology renal carcinoma, accrual of non-clear cell subjects will be ensured by closure of the clear-cell strata if adequate representation is not achieved. *(35)*

Translational Medicine

Predictive and Prognostic Value of AKT/mTOR Pathway Status in Intermediate and High-Risk, Localized Renal Carcinoma

In order to investigate molecular biomarkers relevant to AKT/mTOR pathway inhibition in this trial, relevant translational studies are planned. The overall **hypothesis** is that patients with RCC tumors characterized by AKT/mTOR pathway activation will benefit from mTOR-targeted therapy. In light of the paucity of information regarding the role of AKT/mTOR activation in post-nephrectomy tumor recurrence and survival, it is critical to distinguish between the prognostic and/or predictive roles of these biomarkers. In the context of this Phase III trial, the following aims are proposed: 1) To define the status (activation, expression and functional) of factors involved in AKT/mTOR signaling in RCC tumor specimens, including AKT, mTOR, S6K, 4EBP1, HIFα, vHL, PTEN and other pathway-specific factors. 2) To perform exploratory microarray analysis in tumor tissue to identify genes and pathways prognostic for recurrence and/or predictive of response to everolimus. Pathways to be analyzed will include but not be limited to PI3K-AKT-mTOR, HIF-1α protein translation and VEGFR-PDGFR (using Ingenuity Pathway

Analysis). Additionally, levels of tumor-associated plasma proteins from serial draws will be examined using multiplex analysis. 3) To correlate molecular biomarkers with patient outcome, including recurrence-free survival and overall survival, and to identify specific cohorts of patients, categorized by molecular phenotype, who benefit from everolimus therapy.

The following specimens will be collected for the above studies (further described in Section 15.0): 1) Tissue specimen: a paraffin embedded nephrectomy block will be collected from all consenting patients; 2) blood specimens: (plasma and buffy coats) for correlative studies will be collected after registration, but prior to treatment; at the beginning of Cycles 2 and 3; at the end of therapy (54 weeks); and at the time of recurrence.

Pharmacodynamic Evaluation of Everolimus in Adjuvant Renal Carcinoma Subjects

Everolimus was originally developed for its immunosuppressant properties in the prevention of rejection after solid-organ transplantation. Chronic daily dosing of an mTOR inhibitor has not been studied in an adjuvant cancer population, and potential adverse effects of longer-term administration of everolimus need to be carefully assessed. While this trial will evaluate one year of adjuvant therapy, it is possible that longer dosing duration may eventually prove advantageous, and understanding and predicting for chronic toxicity is important in this population. We will therefore study adverse effects of everolimus in this study population and determine a relationship with steady-state trough levels of the drug.

The published literature on steady-state pharmacokinetics and chronic toxicity has largely been in the post-transplant setting in which everolimus has been co-administered with other immunosuppressant drugs as part of triple-therapy rejection prophylaxis. *(36,37)* The applicability of this toxicity data to the adjuvant renal carcinoma population is clouded by the potential contribution of co-administered immunosuppressant drugs and other factors peculiar to post-transplant setting. Since the adjuvant renal carcinoma setting represents a population with curative potential, mTOR inhibitor side effects with potential long-term implications are of concern, including hyperlipidemia and immunosuppression, which may be a particular issue with the daily dosing regimen studied in cancer. *(38)*

In renal transplantation patients, steady-state minimum concentrations were well correlated with AUC (correlation coefficient [r value] = 0.88). *(39)* A relationship was seen between AUC and thrombocytopenia (p=0.03), and there was a positive trend between AUC and both hypercholesterolemia and hypertriglyceridemia. These results suggest that monitoring C_{min} may serve as a good marker for overall everolimus exposure. *(40)* In the post-transplantation setting, trough levels are recommended to be in the desired range of 3 to 8 ng/mL to optimize efficacy and decrease the incidence of adverse reactions, although it has been suggested that everolimus use with levels of up to 15 ng/mL is manageable. *(41,42)* In Phase I studies in advanced cancer patients using 10 mg daily dosing, trough levels exceeded the recommended range for the transplant population. *(43,44)*

In order to investigate the relationship between steady-state trough concentrations of everolimus in adjuvant renal cancer patients and drug toxicity, we will collect specimens to study the incidence of lymphopenia, infection, thrombocytopenia, hyperglycemia, hypercholesterolemia, hypertriglyceridemia, stomatitis, creatinine elevation, and non-infectious pneumonitis as pharmacodynamic markers of everolimus exposure. Blood sampling for pre-dose trough levels of everolimus will be obtained after registration, but prior to treatment; at the beginning of Cycles 2 and 3; and at the time the patient is removed from protocol treatment. Everolimus whole-blood concentrations will be determined via high-performance liquid chromatography/mass spectroscopy assay. Relationships between everolimus steady-state C_{min} and the incidence of specific toxicities will be explored.

Trough levels of everolimus in post-nephrectomy renal carcinoma patients will correlate with toxicity and yield an upper threshold level that can be used in developing a therapeutic range for everolimus in the adjuvant population.

SWOG Authorized Document

Inclusion of Women and Minorities:

This study was designed to include women and minorities, but was not designed to measure differences of intervention effects. The anticipated accrual in the ethnicity/race and sex categories is shown in the table below. Both men and women of all races and ethnic groups are eligible for this study.

Ethnic Category	Females	Males	Total
Hispanic or Latino	55	89	144
Not Hispanic or Latino	436	957	1393
**Total Ethnic**	491	1046	1537
**Racial Category**			
American Indian or Alaskan Native	1	4	5
Asian	10	42	52
Black or African American	29	42	71
Native Hawaiian or other Pacific Islander	0	0	0
White	451	958	1409
**Racial Category: Total of all Subjects**	491	1046	1537

3.0 DRUG INFORMATION

nvestigator's Brochure

For information regarding Investigator's Brochures, please refer to SWOG Policy 15.

For this study, everolimus is investigational and is being provided under an IND held by SWOG. For IND's filed by SWOG, the protocol serves as the Investigator Brochure for the performance of the protocol. In such instances submission of the protocol to the IRB should suffice for providing the IRB with information about the drug. However, in cases where the IRB insists on having the official Investigator Brochure from the company, further information may be requested by contacting the SWOG Operations Office at 210/614-8808.

3.1 Everolimus (RAD001, Afinitor®, Zortress®) (NSC- 733504) (IND-75,093)

a. PHARMACOLOGY

Mechanism of Action: Everolimus binds to the cytosolic immunophyllin FKBP12; both agents inhibit growth factor-driven cell proliferation, including that of T-cells and vascular smooth muscle cells. The everolimus and FKBP12 complex selectively inhibits mTOR (mammalian target of rapamycin), an intracellular protein kinase implicated in the control of cellular proliferation of neoplastic cells, specifically in the progression of cells from G1 to S phase. Everolimus also reduces angiogenesis by inhibiting VEGF and HIF-1 expression.

b. PHARMACOKINETICS

1. Absorption: Everolimus levels peak in 1-3 hours after oral administration. There is rapid but moderate absorption.

2. Distribution: Everolimus is about 74% protein bound in healthy subjects and patients with moderate hepatic impairment.

SWOG Authorized Document

3. Metabolism: Everolimus is extensively metabolized by CYP3A4 and forms 6 weak metabolites. It is also a P-glycoprotein substrate.

4. Elimination: Everolimus is extensively eliminated via the bile. The elimination half-life of everolimus is about 30 hours and is prolonged in patients with hepatic impairment. Everolimus is primary excreted through the feces.

c. ADVERSE EFFECTS

1. The Comprehensive Adverse Events and Potential Risks list (CAEPR) provides a single list of reported and/or potential adverse events (AE) associated with an agent using a uniform presentation of events by body system. In addition to the comprehensive list, a subset, the Specific Protocol Exceptions to Expedited Reporting (SPEER), appears in a separate column and is identified with bold and italicized text. This subset of AEs (SPEER) is a list of events that are protocol specific exceptions to expedited reporting to NCI (except as noted below). Refer to the 'CTEP, NCI Guidelines: Adverse Event Reporting Requirements' http://ctep.cancer.gov/protocolDevelopment/electronic_applications/docs/aeguidelines.pdf for further clarification. *Frequency is provided based on 3033 patients.* Below is the CAEPR for Everolimus (RAD-001).

NOTE: Report AEs on the SPEER **ONLY IF** they exceed the grade noted in parentheses next to the AE in the SPEER. If this CAEPR is part of a combination protocol using multiple investigational agents and has an AE listed on different SPEERs, use the lower of the grades to determine if expedited reporting is required.

Version 2.4, July 10, 2017[1]

**Adverse Events with Possible Relationship to Everolimus (RAD-001) (CTCAE 4.0 Term) [n= 3033]**			**Specific Protocol Exceptions to Expedited Reporting (SPEER)**
**Likely (>20%)**	**Less Likely (<=20%)**	**Rare but Serious (<3%)**	
BLOOD AND LYMPHATIC SYSTEM DISORDERS			
Anemia			***Anemia (Gr 2)***
GASTROINTESTINAL DISORDERS			
	Abdominal pain		
	Constipation		
Diarrhea[2]			***Diarrhea[2] (Gr 2)***
Mucositis oral[3]			***Mucositis oral[3] (Gr 2)***
	Nausea		***Nausea (Gr 2)***
	Vomiting		***Vomiting (Gr 2)***
GENERAL DISORDERS AND ADMINISTRATION SITE CONDITIONS			
	Edema limbs		***Edema limbs (Gr 2)***
Fatigue			***Fatigue (Gr 2)***
	Fever		***Fever (Gr 2)***
INFECTIONS AND INFESTATIONS			
	Infection[4]		***Infection[4] (Gr 2)***

**Adverse Events with Possible Relationship to Everolimus (RAD-001) (CTCAE 4.0 Term) [n= 3033]**			**Specific Protocol Exceptions to Expedited Reporting (SPEER)**
**Likely (>20%)**	**Less Likely (<=20%)**	**Rare but Serious (<3%)**	
INJURY, POISONING AND PROCEDURAL COMPLICATIONS			
		Wound complication[5]	
INVESTIGATIONS			
	Alanine aminotransferase increased		***Alanine aminotransferase increased (Gr 2)***
	Alkaline phosphatase increased		***Alkaline phosphatase increased (Gr 2)***
	Aspartate aminotransferase increased		***Aspartate aminotransferase increased (Gr 2)***
	Cholesterol high		***Cholesterol high (Gr 2)***
	Creatinine increased		***Creatinine increased (Gr 2)***
	Lymphocyte count decreased		***Lymphocyte count decreased (Gr 2)***
	Neutrophil count decreased		***Neutrophil count decreased (Gr 2)***
	Platelet count decreased		***Platelet count decreased (Gr 2)***
	Weight loss		
	White blood cell decreased		***White blood cell decreased (Gr 2)***
METABOLISM AND NUTRITION DISORDERS			
	Anorexia		***Anorexia (Gr 2)***
	Hyperglycemia[6]		***Hyperglycemia[6] (Gr 2)***
	Hypertriglyceridemia		***Hypertriglyceridemia (Gr 2)***
	Hypophosphatemia		***Hypophosphatemia (Gr 2)***
MUSCULOSKELETAL AND CONNECTIVE TISSUE DISORDERS			
	Arthralgia		
	Back pain		
	Pain in extremity		
NERVOUS SYSTEM DISORDERS			
	Dysgeusia		
	Headache		***Headache (Gr 2)***
RENAL AND URINARY DISORDERS			
		Acute kidney injury	
RESPIRATORY, THORACIC AND MEDIASTINAL DISORDERS			
	Cough		***Cough (Gr 2)***

Adverse Events with Possible Relationship to Everolimus (RAD-001) (CTCAE 4.0 Term) [n= 3033]			Specific Protocol Exceptions to Expedited Reporting (SPEER)
**Likely (>20%)**	**Less Likely (<=20%)**	**Rare but Serious (<3%)**	
	Dyspnea		***Dyspnea (Gr 2)***
	Epistaxis		***Epistaxis (Gr 2)***
	Pneumonitis[7]		
SKIN AND SUBCUTANEOUS TISSUE DISORDERS			
	Dry skin		
	Pruritus		
Rash maculo-papular			***Rash maculo-papular (Gr 2)***
		Skin and subcutaneous tissue disorders - Other (angioedema)[8]	

[1] This table will be updated as the toxicity profile of the agent is revised. Updates will be distributed to all Principal Investigators at the time of revision. The current version can be obtained by contacting PIO@CTEP.NCI.NIH.GOV. Your name, the name of the investigator, the protocol and the agent should be included in the e-mail.

[2] Includes diarrhea, enteritis, enterocolitis, colitis, defecation urgency, and steatorrhea.

[3] Includes stomatitis, aphthous stomatitis, gingival pain/swelling/ulceration, glossitis, glossodynia, lip ulceration, mouth ulceration, tongue ulceration, and mucosal inflammation.

[4] Infection includes all 75 infection sites under the INFECTIONS AND INFESTATIONS SOC.

[5] Everolimus delays wound healing and increases the occurrence of wound-related complications like wound dehiscence, wound infection, incisional hernia, lymphocele, and seroma.

[6] Hyperglycemia may result in either exacerbation of or development of new onset diabetes mellitus.

[7] Includes pneumonitis, interstitial lung disease, lung infiltration, pulmonary alveolar hemorrhage, pulmonary toxicity, alveolitis, pulmonary fibrosis, and restrictive pulmonary disease.

[8] Patients taking concomitant ACE inhibitor therapy may be at increased risk for angioedema.

[9] Includes agitation, anxiety, panic attack, aggression, abnormal behavior, and obsessive compulsive disorder.

Adverse events reported on Everolimus (RAD-001) trials, but for which there is insufficient evidence to suggest that there was a reasonable possibility that Everolimus (RAD-001) caused the adverse event:

BLOOD AND LYMPHATIC SYSTEM DISORDERS - Blood and lymphatic system disorders - Other (thrombotic microangiopathy)
CARDIAC DISORDERS - Atrial fibrillation; Cardiac disorders - Other (myocardial abnormality); Chest pain - cardiac; Heart failure; Left ventricular systolic dysfunction; Myocardial infarction; Sinus bradycardia; Sinus tachycardia; Supraventricular tachycardia
ENDOCRINE DISORDERS - Endocrine disorders - Other (increased blood follicle stimulating hormone [FSH] levels); Endocrine disorders - Other (increased blood luteinizing hormone [LH] levels); Endocrine disorders - Other (low testosterone); Hypothyroidism
EYE DISORDERS - Blurred vision; Conjunctivitis; Keratitis
GASTROINTESTINAL DISORDERS - Ascites; Colitis; Dry mouth; Dyspepsia; Dysphagia; Flatulence; Gastroesophageal reflux disease; Gastrointestinal disorders - Other (Dieulafoy's lesion); Hemorrhoids; Intra-abdominal hemorrhage; Oral pain; Pancreatitis; Periodontal disease; Toothache
GENERAL DISORDERS AND ADMINISTRATION SITE CONDITIONS - Chills; Edema face; Edema trunk; Flu like symptoms; Irritability; Non-cardiac chest pain; Pain
HEPATOBILIARY DISORDERS - Hepatic failure; Hepatobiliary disorders - Other (hepatomegaly)
IMMUNE SYSTEM DISORDERS - Allergic reaction
INVESTIGATIONS - Activated partial thromboplastin time prolonged; Blood bilirubin increased; CPK increased; GGT increased; INR increased; Investigations - Other (bicarbonate decreased); Investigations - Other (increased lactate dehydrogenase); Investigations - Other (low density lipoprotein raised); Investigations - Other (thrombocythemia)
METABOLISM AND NUTRITION DISORDERS - Dehydration; Glucose intolerance; Hypercalcemia; Hyperkalemia; Hypoalbuminemia; Hypocalcemia; Hypokalemia; Hypomagnesemia; Hyponatremia; Metabolism and nutrition disorders - Other (high ammonia); Metabolism and nutrition disorders - Other (hyperlipidemia)
MUSCULOSKELETAL AND CONNECTIVE TISSUE DISORDERS - Bone pain; Chest wall pain; Generalized muscle weakness; Muscle weakness lower limb; Musculoskeletal and connective tissue disorder - Other (muscle spasms); Myalgia
NEOPLASMS BENIGN, MALIGNANT AND UNSPECIFIED (INCL CYSTS AND POLYPS) - Neoplasms benign, malignant and unspecified (incl cysts and polyps) - Other (ovarian cysts)
NERVOUS SYSTEM DISORDERS - Dizziness; Encephalopathy; Hydrocephalus; Lethargy; Paresthesia
PSYCHIATRIC DISORDERS - Agitation; Anxiety[9]; Delirium; Depression; Insomnia; Mania
RENAL AND URINARY DISORDERS - Hematuria; Proteinuria; Urinary frequency
REPRODUCTIVE SYSTEM AND BREAST DISORDERS - Dysmenorrhea; Genital edema; Irregular menstruation; Menorrhagia; Vaginal hemorrhage
RESPIRATORY, THORACIC AND MEDIASTINAL DISORDERS - Bronchopulmonary hemorrhage; Pharyngolaryngeal pain; Pleural effusion; Respiratory failure; Respiratory, thoracic and mediastinal disorders - Other (rales); Respiratory, thoracic and mediastinal disorders - Other (rhinorrhea); Sore throat; Voice alteration
SKIN AND SUBCUTANEOUS TISSUE DISORDERS - Nail loss; Palmar-plantar erythrodysesthesia syndrome; Rash acneiform; Skin and subcutaneous tissue disorders - Other (nail disorder); Skin and subcutaneous tissue disorders - Other (skin lesion); Skin ulceration

SWOG Authorized Document

VASCULAR DISORDERS - Flushing; Hypertension; Lymphedema; Phlebitis; Thromboembolic event; Vascular disorders - Other (acute bowel ischemia); Vascular disorders - Other (hemorrhage)

Note: Everolimus (RAD-001) in combination with other agents could cause an exacerbation of any adverse event currently known to be caused by the other agent, or the combination may result in events never previously associated with either agent.

2. Pregnancy and Lactation: Pregnancy category D. It is not known if everolimus is excreted in human milk.

   Drug Interactions: Everolimus is a substrate of cytochrome P450 3A4 (CYP3A4) and also a substrate and moderate inhibitor of the multidrug efflux pump P-glycoprotein (PgP). *In vitro*, everolimus is a competitive inhibitor of CYP3A4 and a mixed inhibitor of CYP2D6. Due to the extensive number of everolimus drug interactions, a complete patient medication list, including everolimus, should be screened prior to initiation of everolimus (as indicated in Section 7.3 and Appendix 18.3).

   Patients taking concomitant angiotensin converting enzyme (ACE) inhibitor therapy may be at increased risk for angioedema (e.g. swelling of the airways or tongue, with or without respiratory impairment).

3. Hepatic Impairment: Dosage adjustment of everolimus is recommended in moderate hepatic impairment (Child-Pugh class B). Contact the Study Chair for guidance. Use of everolimus is not recommended in severe hepatic impairment (Child-Pugh class C).

d. DOSING & ADMINISTRATION

1. Dosing – See Treatment Plan (see Section 7.2)

2. Everolimus (or matching placebo) should be administered orally, once daily preferably in the morning with a glass of water and no more than a light fat-free meal. Tablets should be swallowed whole with a glass of water. Grapefruit or citrus juices should be avoided. The tablets must not be chewed or crushed. If unable to swallow whole tablet, may disperse tablet completely in 30 mL water and drink immediately; rinse container with additional 30 mL water and swallow.

e. STORAGE & STABILITY

The intact blister packs should be stored at controlled room temperature (15°-30°C) and protected from light. Current stability data permit shelf life of 24 months for 5 mg tablet variant based on solid dispersion dried by paddle dryer and 36 months for 5 mg tablet variant based on solid dispersion dried by evaporation/drying oven if stored below 30°C in the original double sided aluminum blister and protected from light and moisture.

f. HOW SUPPLIED

1. Everolimus or matching placebo will be supplied as tablets blister-packed under aluminum foil in units of 10 tablets. Blisters should be opened only immediately prior to ingestion as the drug is both hygroscopic and light-sensitive.

SWOG Authorized Document

Everolimus or matching placebo 5 mg tablets are white to slightly yellow, elongated tablets with a beveled edge and no score, engraved with “5” on one side and “NVR” on the other.

Everolimus is commercially available, however it is considered investigational for this study. Everolimus 5 mg tablets and placebo to match everolimus 5 mg tablets will be supplied by Novartis Pharmaceuticals Corporation and will be distributed by the Department of Veterans Affairs Cooperative Studies Program Clinical Research Pharmacy Coordinating Center (PCC). Each participating Institution must have a Pharmacy ID Number, in addition to their Institution Number, before randomization of subjects can begin. SWOG institutions already have a pharmacy associated with them, so they do not need to report a Pharmacy ID number since that information is already linked in the SWOG database. However, Institutions registering via CTSU must be assigned a pharmacy ID number by the PCC. With the initial and each subsequent randomization, the Institution registering via CTSU will be required to provide their Institution’s Pharmacy ID Number. No randomizations can be completed without a Pharmacy ID Number. For CTSU Participating Institutions, each Institution must call the PCC at (505) 248-3203 to register their Institution with the PCC and receive a Pharmacy ID Number. This registration by CTSU Participating Institutions with the PCC must be completed **at least 1 working day prior to the randomization of the first subject** at the site or randomization of the first subject will have to be postponed. When calling the PCC, the caller will be asked which study they are calling in regards to. To facilitate the caller being transferred to the correct PCC staff, the caller should indicate the “SWOG protocol “S0931”, “EVEREST”, or “P261” (the PCC internal number for the study). To register an Institution, the PCC will require:

- the name of the receiving individual,
- complete street address, and phone number,
- e-mail address of the receiving individual.

Everolimus or matching placebo will be packaged by the PCC and supplied to the Institutions in kits of 10 blister cards, with each kit containing sufficient drug for one cycle of treatment. No supply of unassigned everolimus or matching placebo will be maintained at the institutions. Rather everolimus or matching placebo will be supplied to the site in a “just in time” manner. **Upon notification of a randomization by the SWOG Statistical Center, the PCC will ship the first patient-specific kit to an Institution to arrive within four working days.** Each kit will be labeled specifically for an individual subject with the subject’s SWOG Patient Number.

Subsequent patient-specific kits will automatically be shipped to the Institution approximately mid-cycle of Cycles 1-8. The blister card and kits labels will be permanently attached. The site will write down the kit numbers on the Drug Accountability Record Form when the kit is dispensed.

If a subject requires a replacement kit (Emergency Kit) for lost (etc.) medication, an Emergency Kit should be supplied by calling the PCC at 505/248-3203.

Prior to dispensing the next kit of study medication, or at the study visit at the end of everolimus or placebo treatment, the old blister cards and any

SWOG Authorized Document

unused tablets are to be collected from the subjects and quantity remaining logged on to the Drug Accountability Records. Any unused, partially used, or expired study drug remaining when a subject goes off treatment should be destroyed on-site in accordance with local institutional policy. In the event that a site does not have documented destruction procedures in place, call PCC at (505/248-3203 for instructions on how to return leftover study drug.

2. Drug Handling and Accountability

a. Drug accountability**:** The investigator, or a responsible party designated by the investigator, must maintain a careful record of the receipt, dispensing, and destruction of all study drugs received from the distributor using the NCI Oral Drug Accountability Record Form (NCI Oral DARF)**,** available at http://ctep.cancer.gov. A separate record must be maintained for each patient on this protocol.

b. Electronic logs are allowed as long as a print version of the log process is the exact same appearance as the current NCI Oral DARF.

3. Drug Return and/or Disposition Instruction

a. Drug Returns: All unused drug, unopened and unused blister cards remaining when a subject goes off treatment, and expired blister cards should be destroyed on-site in accordance with institutional policy. Partially used blister cards with remaining tablets should be documented in the patient-specific accountability record (i.e., logged in as "# of tablets returned") and destroyed on-site in accordance with institutional policy.

b. Drug Transfers: Blister cards **MAY NOT** be transferred from one patient to another patient or from one protocol to another protocol. All other transfers (e.g. a patient moves from one participating institution to another participating institution) must be approved **in advance** by calling the PCC at (505) 248-3203.

Contact Information: Questions about drug orders, transfers, returns or accountability should be addressed to the PCC at (505) 248-3203.

4.0 STAGING CRITERIA

<u>DEFINITION OF TNM</u> (AJCC Seventh Edition, 2010)

Primary Tumor (T)

TX	Primary tumor cannot be assessed
T0	No evidence of primary tumor
T1	Tumor 7 cm or less in greatest dimension, limited to the kidney
T1a	Tumor 4 cm or less in greatest dimension, limited to the kidney
T1b	Tumor more than 4 cm but not more than 7 cm in greatest dimension, limited to the kidney
T2	Tumor more than 7 cm in greatest dimension, limited to the kidney
T2a	Tumor more than 7 cm but less than or equal to 10 cm in greatest dimension, limited to the kidney
T2b	Tumor more than 10 cm, limited to the kidney

SWOG Authorized Document

T3	Tumor extends into major veins or perinephric tissues, but not into the ipsilateral adrenal gland and not beyond Gerota's Fascia
T3a	Tumor grossly extends into the renal vein or its segmental (muscle containing) branches, or tumor invades perirenal and/or renal sinus fat but not beyond Gerota's Fascia
T3b	Tumor grossly extends into the vena cava below the diaphragm
T3c	Tumor grossly extends into the vena cava above the diaphragm or invades the wall of the vena cava
T4	Tumor invades beyond Gerota's Fascia (including contiguous extension into the ipsilateral adrenal gland). NOTE: Non-contiguous adrenal lesion is considered M1 disease and not eligible for protocol participation.

Regional Lymph Nodes (N)

NX	Regional lymph nodes cannot be assessed
N0	No regional lymph node metastasis
N1	Metastasis in regional lymph node(s)

Distant Metastasis (M)

M0	No distant metastasis

HISTOLOGIC GRADE (G)

GX	Grade cannot be assessed
G1	Well differentiated
G2	Moderately differentiated
G3	Poorly differentiated
G4	Undifferentiated

PATHOLOGIC STAGE

Intermediate High-Risk Group

pT1b	G3-4, N0 (or pNX where clinically N0), M0
pT2a-b	G1-2, N0 (or pNX where clinically N0), M0
pT2 a-b	G3-4, N0 (or pNX where clinically N0), M0
pT3a	G1-2, N0 (or pNX where clinically N0), M0

Microvascular invasion of the renal vein of pT1a-pT3a (as long as pT3a is Grade 1-2) N0 (or pNX where clinically N0) M0

Very High Risk Group

pT3a	G3-4, N0 (or pNX where clinically N0), M0
pT3b-c	G any, N0 (or pNX where clinically N0), M0
pT4	G any, N0 (or pNX where clinically N0), M0
pT any	G any, N+ (fully resected), M0

Microvascular invasion of the renal vein with above other characteristics

5.0 ELIGIBILITY CRITERIA

Each of the criteria in the following section must be met in order for a patient to be considered eligible for registration. Use the spaces provided to confirm a patient's eligibility. For each criterion requiring test results and dates, please record this information on the **S0931** Prestudy Form and submit to the Data

SWOG Authorized Document

Operations Center in Seattle (see Section 14.0). Any potential eligibility issues should be addressed to the Data Operations Center in Seattle at guquestion@crab.org or 206/652-2267 prior to registration.

In calculating days of tests and measurements, the day a test or measurement is done is considered Day 0. Therefore, if a test is done on a Monday, the Monday 2 weeks later would be considered Day 14. This allows for efficient patient scheduling without exceeding the guidelines. **If Day 14, 28, 42, or 84 falls on a weekend or holiday, the limit may be extended to the next working day.**

5.1 Disease Related Criteria

a. Patients must have histologically or cytologically confirmed renal cell carcinoma (clear cell or non-clear cell allowed, but collecting duct or medullary carcinomas excluded). Patients must be considered pathologically either Intermediate High Risk or Very High Risk as defined in Section 4.0. Patients must not have a history of distant metastases. Patients with microvascular invasion of the renal vein of any grade or stage (as long as M0) are also eligible.

b. Patients must have undergone a full surgical resection (radical nephrectomy or partial nephrectomy), including removal of all clinically positive nodes. Surgical margins must be negative. Patients with positive renal vein margins are eligible unless there is invasion of the renal vein **wall** at the margin (provided no other margins are positive). Patients must plan to start study drug within 84 days after the date of full surgical resection. Patients must have recovered from any surgical related complications.

c. Patients with bilateral renal tumors are eligible provided both tumors have undergone full surgical resection and at least one of the tumors meets all eligibility criteria in Sections 5.1a-b. Patients must plan to start study drug within 84 days after the date of the resection of the first tumor.

d. Patients must not have any evidence of residual or metastatic renal cell cancer on CT scan of the chest, abdomen, and pelvis, all with oral and IV contrast after nephrectomy and within a maximum of 28 days prior to registration. An MRI scan of the abdomen/pelvis with gadolinium and a non-contrast CT of the chest is an acceptable imaging alternative. Non-contrast CT of the chest/abdomen/pelvis should only be performed if, in the opinion of the investigator, it is in the best medical interest of the patient to not receive IV contrast of any form. NOTE: PET/CT is not an acceptable imaging alternative. Patients who display subcentimeter pulmonary nodules (by CT scan) that are non-specific and considered unlikely to represent metastatic disease by the treating investigator will be considered eligible.

5.2 Prior Therapy Criteria

a. Patients must not have received any prior anti-cancer therapy (except for radical or partial nephrectomy noted above) for renal cell carcinoma, including systemic therapy in the adjuvant or neoadjuvant setting, immunotherapy, investigational therapy, surgical metastaticity, or radiation therapy.

b. Patients must not be planning to receive other anti-cancer agents including investigational agents while on protocol treatment.

c. Patients must not be receiving chronic, systemic treatment with corticosteroids or other immunosuppressive agent. Topical or inhaled corticosteroids are allowed.

d. Patients must not have received immunization with an attenuated live vaccine within seven days prior to registration nor have plans to receive such vaccination while on protocol treatment.

SWOG Authorized Document

e. Patients must not be taking, nor plan to take while on protocol treatment, strong CYP3A4 inhibitors, (e.g. ketoconazole, itraconazole, voriconazole, posaconazole, fluvoxamine, nefazodone, nelfinavir, ritonavir) and/or strong CYP3A4 inducers (e.g. phenytoin, rifampin, rifabutin) within 14 days prior to randomization. (Moderate inhibitors or inducers of isoenzyme CYP3A4 should be avoided, but if necessary can be used with caution. See Section 7.1c.)

5.3 Clinical/Laboratory Criteria

a. Patients must have a complete physical examination and medical history within 28 days prior to registration.

b. Patients must not have any Grade III/IV cardiac disease as defined by the New York Heart Association Criteria (i.e., patients with cardiac disease resulting in marked limitation of physical activity or resulting in inability to carry on any physical activity without discomfort), unstable angina pectoris, myocardial infarction within 6 months, or serious uncontrolled cardiac arrhythmia. See Appendix 18.1.

c. Patients must not have any known uncontrolled underlying pulmonary disease (e.g. FEV1 or DLCO 50% or less of predicted **OR** O2 saturation 88% or less at rest on room air).

d. Patients must not have any known hypersensitivity to everolimus or other rapamycins (sirolimus, temsirolimus) or to its excipients.

e. Patients must have absolute neutrophil count (ANC) ≥ 1,500/mcL, platelet count ≥ 100,000/mcL and hemoglobin ≥ 9.0 g/dl obtained within 28 days prior to registration.

f. Patients must have serum creatinine ≤ 2.0 x upper limit of normal (ULN) or calculated creatinine clearance (CrCl) ≥ 30mL/min obtained within 28 days prior to registration.

$$\text{Calculated creatinine clearance} = \frac{(140 - \text{age}) \times \text{wt (kg)} \times 0.85 \text{ (if female)}}{72 \times \text{creatinine (mg/dl)}}$$

g. Patients must have bilirubin ≤ 1.5 x ULN, and SGOT and SGPT ≤ 2.5 x ULN obtained within 28 days prior to registration.

h. Patients must NOT have liver disease such as cirrhosis or severe hepatic impairment (Child-Pugh Class C). NOTE: A detailed assessment of Hepatitis B/C medical history and risk factors must be done at screening for all patients. HBV and HCV testing are required at screening for all patients with a positive medical history based on risk factors and/or confirmation of prior HBV/HCV infection. (See Section 9.0 for testing information and Section 8.6 for management of patients with positive testing.)

i. Patients must not have a known history of HIV seropositivity.

j. Patients must not have uncontrolled hyperlipidemia (fasting serum cholesterol >300 mg/dL AND fasting triglycerides > 2.5 x ULN) obtained within 28 days prior to registration. Optimal lipid control must be achieved before registration and monitored during protocol treatment (see Section 8.4b).

k. Patients must not have uncontrolled diabetes mellitus (defined by fasting serum glucose > 1.5 x ULN) obtained within 28 days prior to registration. Optimal

SWOG Authorized Document

glucose control must be achieved before registration and monitored during protocol treatment (see Section 8.4b).

l. Patients must be able to take oral medications. Patient may not have any impairment of gastrointestinal function or gastrointestinal disease that may significantly alter the absorption of everolimus (e.g. ulcerative disease, uncontrolled nausea, vomiting, diarrhea, malabsorption syndrome or small bowel resection).

m. Patients must have a Zubrod Performance Status of 0 or 1 (see Section 10.3).

n. All patients must be 18 years of age or older.

o. No other prior malignancy is allowed except for the following: adequately treated basal cell or squamous cell skin cancer, in situ cervical cancer, adequately treated Stage I or II cancer from which the patient is currently in complete remission, or any other cancer from which the patient has been disease-free for 5 years.

p. Patients must not be pregnant or nursing due to animal studies that have shown reproductive toxicity effects. Women/men of reproductive potential must have agreed to use an effective contraceptive method during protocol treatment and up to 8 weeks after ending protocol treatment. A woman is considered to be of "reproductive potential" if she has had menses at any time in the preceding 12 consecutive months. In addition to routine contraceptive methods, "effective contraception" also includes heterosexual celibacy and surgery intended to prevent pregnancy (or with a side-effect of pregnancy prevention) defined as a hysterectomy, bilateral oophorectomy or bilateral tubal ligation. However, if at any point a previously celibate patient chooses to become heterosexually active during the time period for use of contraceptive measures outlined in the protocol, he/she is responsible for beginning contraceptive measures.

5.4 Translational Medicine Criteria

a. Patients must be offered the opportunity to participate in specimen banking for future use to include the translational medicine studies outlined in Section 15.0.

5.5 Regulatory Criteria

a. All patients must be informed of the investigational nature of this study and must sign and give written informed consent in accordance with institutional and federal guidelines.

b. As part of the OPEN registration process (see Section 13.4 for OPEN access instructions) the treating institution's identity is provided in order to ensure that the current (within 365 days) date of institutional review board approval for this study has been entered into the data base.

SWOG Authorized Document

6.0 STRATIFICATION FACTORS

Patients will be randomized using a dynamic balancing algorithm *(45)* with stratification based on:

a. Risk group based on pathologic stage (intermediate high-risk vs very high risk) (see Section 4.0),

b. Histology (clear cell vs non-clear cell); (NOTE: The tumor will be classified as clear cell if ***any*** component of clear cell histology is identified in the specimen.)

c. Performance status (0 vs 1).

7.0 TREATMENT PLAN

For treatment or dose modification related questions, please contact Dr. Christopher Ryan at 503/494-8487 or Dr. Elisabeth Heath at 313/576-8715. For dosing principles or questions, please consult the SWOG Policy #38 "Dosing Principles for Patients on Clinical Trials" at http://swog.org (then click on "Policies and Manuals" under the "Visitors" menu and choose Policy #38).

7.1 Good Medical Practice

The following tests (and/or assessments) are recommended within 28 days prior to registration in accordance with Good Medical Practice. Results of these tests do not determine eligibility and minor deviations from normal limits would be acceptable if they do not affect patient safety in the clinical judgment of the treating physician. If there are significant deviations in these tests/assessments that could impact on patient safety, it is highly recommended that the registering investigator discuss the patient with the Study Chair prior to registering.

a. Patients should not have an uncontrolled intercurrent illness including, but not limited to active bleeding diathesis, uncontrolled infection/disorders, nonmalignant medical illnesses that are uncontrolled or whose control may be jeopardized by the treatment with the study therapy, or psychiatric illness/social situations that would limit compliance with study requirements.

b. Testing for hepatitis B viral load and serologic markers: HBV-DNA, HBsAg, HBs Ab, and HBc Ab should be performed at screening for all patients with the following risk factors (see Section 7.3 and 8.6):

- Patients who currently live in (or have lived in) Asia, Africa, Central and South America, Eastern Europe, Spain, Portugal, and Greece.
- known or suspected past hepatitis B infection,
- blood transfusion(s) prior to 1990,
- current or prior IV drug users,
- current or prior dialysis,
- current or prior high-risk sexual activity,
- body piercing (other than ear piercing) or tattoos,
- mother known to have hepatitis B
- history suggestive of hepatitis B infection, e.g., dark urine, jaundice, right upper quadrant pain.
- Additional patients at the discretion of the investigator
- household contact with hepatitis B infected patient(s),

Testing for hepatitis C should be performed using quantitative RNA-PCR at screening for patients with the following risk factors (see Section 8.6):

- known or suspected past hepatitis C infection (including patients with past interferon 'curative' treatment),
- blood transfusions prior to 1990,
- current or prior IV drug users,
- current or prior dialysis,
- household contact of hepatitis C infected patient(s),
- current or prior high-risk sexual activity,
- body piercing other than ear piercing or tattoos,
- Additional patients at the discretion of the investigators

c. Coadministration of moderate CYP3A4 inhibitors (such as erythromycin, fluconazole, calcium channel blockers, benzodiazepines) and inducers (such as bamazepine, phenobarbital) should be avoided if possible, or used subject to caution. Coadministration with substrates, inducers, or inhibitors of P-glycoprotein should be avoided if possible, or used subject to caution. See Appendix 18.3. Grapefruit and grapefruit juice affect cytochrome P450 and P-glycoprotein activity and should be avoided.

d. Alkaline phosphatase obtained.

7.2 Treatment Schedule

All patients will be randomized to receive either everolimus or placebo daily for nine, six week cycles.

AGENT	DOSE	ROUTE	DAYS	DURATION
Everolimus or Matched Placebo	10 mg (two 5 mg Tablets)	Oral	Every Day	378 days (54 weeks)

* Everolimus (or matched placebo) tablets are supplied as 5 mg tablets.

Everolimus must be administered orally, once daily preferably in the morning with a glass of water and no more than a light fat-free meal. Tablets must be swallowed whole with a glass of water. The tablets must not be chewed or crushed, and grapefruit or citrus juices should be avoided.

A cycle of treatment is six weeks. Assessment for the primary endpoint (recurrence) must occur after every three cycles (18 weeks) for 54 weeks, then every six months for two years, and then annually thereafter until recurrence, death, or 10 years after registration, whichever comes first (<u>regardless whether patient is still on active protocol treatment</u>). Recurrence assessments consist of physical exams and CT scans.

Radiographic assessment for pneumonitis must be undertaken as per Section 8.4c.

7.3 Concomitant Therapy

All medications taken other than study drugs and significant non-drug therapies (including physical therapy and blood transfusions) must be recorded. If concomitant therapy must be added or changed, the reason and name of the drug/therapy must be documented in the "comments" section of the **S0931** Treatment Form.

In general, the use of any concomitant medication/therapies deemed necessary for the care of the patient are allowed, including drugs given prophylactically (e.g. antiemetics ± steroids), with the following exceptions:

- No other investigational therapy must be given to patients.
- No chronic treatment with systemic steroids or another immunosuppressive agent. Topical or inhaled corticosteroids are allowed.
- No anticancer agents other than the study medications administered as part of this study protocol must be given to patients. If such agents are required for a patient then the patient must be withdrawn from protocol treatment.
- Growth factors (e.g. G-CSF, G-GM-CSF, erythropoietin, platelets growth factors etc.) are not to be administered prophylactically but may be prescribed by the treating physician for rescue from severe hematologic events.
- Live vaccines must not be administered to patient due to immunosuppressant potential of everolimus.
- Drugs known to be strong inhibitors or inducers of the isoenzyme CYP3A4 must not be administered as systemic therapy (see Section 5.2e). Drugs or substances known to be moderate inhibitors or inducers of CYP3A (see Appendix 18.3) should be avoided if possible or used subject to caution as these can alter everolimus metabolism. Co-administration with strong or moderate inhibitors of P-glycoprotein (PgP) (see Appendix 18.3) should be avoided if possible, or used subject to caution Seville orange, star fruit, grapefruit and their juices affect P450 and PgP activity. Concomitant use should be avoided.
- It is highly recommended that patients positive HBV-DNA or HBsAg are treated prophylactically with an antiviral for 1-2 weeks prior to receiving study drug (see Section 8.5).
- The antiviral treatment should continue throughout the entire study period and for at least 4 weeks after the last dose of study drug.
- Patients on antiviral prophylaxis treatment or positive HBV antibodies should be tested for HBV-DNA according to study visit schedule (See Section 8.6 and 9.0).

7.4 Intake Calendar

Everolimus drug compliance will be recorded on a treatment Intake Calendar (see Appendix 18.4). Institutional CRAs will review and ascertain patient adherence with protocol therapy at the end of treatment for each cycle. Calendar should be kept in the patient's clinic chart. Sites utilizing the CIRB must use the Intake Calendar provided. Sites not utilizing the CIRB may use institutional pill diaries or other source documentation in place of Intake Calendar at the discretion of the treating physician.

7.5 Unblinding Procedures

The procedures for emergency unblinding are outlined in Appendix 18.2. Patients who have recurrence of disease as defined in Section 10.1 have the option to be unblinded in planned fashion, as described in this section. Emergency unblinding will be done for anything other than recurrence as defined in Section 10.1. Patients who undergo optional treatment unblinding and are found to have been randomized to “placebo” will not receive investigationally-supplied everolimus. Novartis Pharmaceutical Corporation will NOT provide investigational supplies of everolimus once a patient has been unblinded.

a. Criteria for Planned Unblinding:

The planned unblinding procedure applies to patients who experience recurrence as defined in Section 10.1. It is vital to properly apply the protocol specified definition of recurrence. If any questions arise with regard to recurrence for a patient, please contact the GU Data Coordinator in Seattle by e-mail at guquestion@crab.org or by telephone at 206/652-2267, 6:30 a.m. to 4:00 p.m. Pacific Time, Monday through Friday, excluding holidays.

Prior to requesting a planned unblinding, the following forms must be submitted and processed by the SWOG Data Operations Office:

SWOG Authorized Document

Off Treatment Notice
S0931 Recurrence Assessment Form
Radiology report(s) supporting recurrence (see Section 10.1)
Pathology report(s) supporting recurrence (see Section 10.1)

Please submit all required forms to the SWOG Data Operations Center (guquestion@crab.org) with the subject line "**S0931** Patient #XXXXXX, Requesting Planned Unblinding". Please allow a minimum of 2 working days for review of forms.

Dr. Ryan (or Heath) will provide a written confirmation to the site that the patient has an adequately documented recurrence. This documentation should be placed in the patient's chart.

b. Planned Unblinding Procedures

You may unblind patients at time of disease recurrence from Member, Affiliate, and CCOP institutions to a Therapeutics study using the SWOG planned unblinding program. To access the planned unblinding program go to the SWOG Web site (http://swog.org) and click on the *Logon* link to go to the SWOG Members Area logon page (https://swog.org/members/logon.asp). This Web program is available at any time except for periods listed under *Down Times.* Log on as an Individual User using your SWOG Roster ID Number and individual web user password. Help for the logon process may be found at https://swog.org/members/logonhelp.asp. After you have logged on, click on the *Workbenches* link, then the *CRA Workbench* link, and then the *Planned Unblinding* link. Go to the Entry Page for the Planned Unblinding program. The CRA must have the SWOG ID number, initials, and investigator number available for the unblinding.

To unblind a patient at disease recurrence, you must meet the following criteria (in order):

1. You are entered into the SWOG Roster and issued a SWOG Roster ID Number.

2. You are associated as an investigator or CRA/RN to the institution where a planned unblinding is occurring.

3. You are granted permission to use the Planned Unblinding program at that institution.

For assistance with points 1 and 2 call the SWOG Operations Office at 210/614-8808. For point 3 you must contact your Web User Administrator. Each SWOG institution has one or more Web User Administrators who may set up Web Users at their institution and assign permissions and passwords to these users.

Non-SWOG sites may unblind patients at time of disease recurrence using the following url: https://crawb.crab.org/TXWB/ctsulogon.aspx.

If the Web Planned Unblinding program is not used, the planned unblinding must be done by phone.

Member, Affiliate and CCOP Institutions

Planned unblinding by phone of patients from Member, Affiliate and CCOP institutions must be done through the SWOG Data Operations Center in Seattle by telephoning

206/652-2267, 6:30 a.m. to 4:00 p.m. Pacific Time, Monday through Friday, excluding holidays.

NOTE: When unblinding **S0931** patients in the SWOG Planned Unblinding Program leave “Bottle ID” field blank.

7.6 Criteria for Removal from Protocol Treatment

a. Recurrence of disease or symptomatic deterioration (as defined in Section 10.0). If the patient has documented recurrence as defined in Section 10.1, unblinding of study treatment as outlined in Section 7.5 may be requested.

b. Unacceptable toxicity defined as any toxicity requiring discontinuation of study drug per Section 8.0. If a patient is removed from protocol treatment due to toxicity, he/she must continue to be followed for recurrence according to the same schedule as detailed in Sections 7.2 and 9.0 (every 4 months [18 weeks] during Year 1 (regardless whether patient is still on active protocol treatment), then every six months for Years 2 and 3, and then annually thereafter until recurrence, death, or 10 years after registration, whichever comes first. Recurrence assessments consist of physical exams and CT scans)

c. Delay in protocol treatment > 21 days due to pneumonitis or > 28 days due to any other reason. If a patient is removed from protocol treatment due to toxicity, the must continue to be followed for recurrence according to the same schedule as detailed in Sections 7.2 and 9.0 (every 4 months [18 weeks] during Year 1 (regardless whether patient is still on active protocol treatment), then every six months for two years, and then annually thereafter until recurrence, death, or 10 years after registration, whichever comes first. Recurrence assessments consist of physical exams and CT scans)

d. Completion of nine cycles of protocol treatment.

e. The patient may withdraw from the study at any time for any reason.

7.7 Discontinuation of Treatment

All reasons for discontinuation of treatment must be documented in the study forms.

7.8 Follow-Up Period

All patients will be followed for a maximum of 10 years after registration or until death (whichever occurs first) for recurrence and survival.

8.0 DOSE MODIFICATIONS

8.1 NCI Common Terminology Criteria for Adverse Events

This study will utilize the CTCAE (NCI Common Terminology Criteria for Adverse Events) Version 4.0 for toxicity and Serious Adverse Event reporting. A copy of the CTCAE Version 4.0 can be downloaded from the CTEP home page (http://ctep.cancer.gov). All appropriate treatment areas should have access to a copy of the CTCAE Version 4.0.

8.2 General Dose Modification Considerations

a. Missed doses are to be omitted rather than made up.

b. If multiple toxicities are experienced, dose modifications will be based on the toxicity requiring the largest dose reduction.

SWOG Authorized Document

c. Reductions are based on the dose given in the preceding cycle and are based on toxicities observed since the prior toxicity evaluation.

d. Once dose is reduced, patients will continue at new dose. No dose re-escalations are allowed.

e. If protocol treatment is held > 21 days for pneumonitis or > 28 days due to any other reason, patient should be removed from protocol treatment.

8.3 Everolimus Dose Levels

Dose Levels	Dose
Full	10 mg daily (two 5 mg tablets)
-1 Level	5 mg daily
-2 Level	5 mg every other day
-3 Level	Discontinue study drug

8.4 Everolimus Dose Modifications

NOTE: NO DOSE ESCALATION OR RE-ESCALATION IS ALLOWED.

Toxicity	Actions
**Non-hematological toxicity**	
Grade 2<br>(except pneumonitis – see 8.4.c)<br>(except hyperlipidemia – see 8.4.b)*	If the toxicity is tolerable to the patient, maintain the same dose. If the toxicity is intolerable to patient, interrupt everolimus until recovery to Grade ≤ 1. Then reintroduce everolimus at same dose.<br>If event returns to Grade 2, then interrupt everolimus until recovery to Grade ≤ 1. Then reintroduce everolimus at one lower dose level.
Grade 3<br>(except pneumonitis – see 8.4.c)<br>(except hyperlipidemia – see 8.4.b)*	Interrupt everolimus until recovery to Grade ≤ 1. Then reintroduce everolimus at one lower dose level. For pneumonitis consider the use of a short course of corticosteroids.
Grade 4	Discontinue everolimus.
**Hematological toxicity**	
Grade 2 Thrombocytopenia (platelets < 75, ≥ 50×10^9/L)	Interrupt everolimus until recovery to Grade ≤1 (≥ 75 $\times 10^9$/L). Then reintroduce everolimus at same dose.<br>If thrombocytopenia again returns to Grade 2, interrupt everolimus until recovery to Grade ≤1. Then reintroduce everolimus at one lower dose level.
Grade 3 Thrombocytopenia (platelets < 50, ≥ 25×10^9/L)	Interrupt everolimus until recovery to Grade ≤ 1 (platelets ≥ 75 $\times 10^9$/L). Then resume everolimus at one lower dose level. If Grade 3 thrombocytopenia recurs, discontinue everolimus.
Grade 4 Thrombocytopenia (platelets < 25 $\times 10^9$/L)	Discontinue everolimus.

SWOG Authorized Document

Toxicity	Actions
**Non-hematological toxicity**	
Grade 3 Neutropenia (neutrophils < 1, ≥ 0.5 x10^9/L)	Interrupt everolimus until recovery to Grade ≤1 (neutrophils ≥ 1.5 x 10^9/L). Then resume everolimus at the same dose. If ANC again returns to Grade 3, hold everolimus until the ANC ≥ 1.5 x 10^9/L. Then resume everolimus at one lower dose level. Discontinue patient from study therapy for a third episode of Grade 3 neutropenia.
Grade 4 Neutropenia (neutrophils < 0.5 x10^9/L)	Interrupt everolimus until recovery to Grade ≤ 1 (neutrophils ≥ 1.5 x 10^9/L). Then resume everolimus at one lower dose level. If Grade 3 or Grade 4 neutropenia occurs despite this dose reduction, discontinue everolimus.
Grade 3 febrile neutropenia (not life-threatening)	Interrupt everolimus until resolution of fever and neutropenia to Grade ≤ 1. Hold further everolimus until the ANC ≥ 1,500/mm^3 and fever has resolved. Then resume everolimus at one lower dose level. If febrile neutropenia recurs, discontinue everolimus.
Grade 4 febrile neutropenia (life-threatening)	Discontinue everolimus.

* Grade 2-3 hyperlipidemia (hypercholesterolemia and/or hypertriglyceridemia) should be managed using medical therapies (see Section 8.4b).

If a patient requires a dose delay of > 21 days for pneumonitis or > 28 days for any other reason from the intended day of the next scheduled dose, then the patient must be removed from the protocol treatment.

a. Hematological Toxicity

No dose adjustments are recommended for anemia. The treating physician may hold everolimus for anemia if it is felt to be in the best interest of the patient. Aranesp (darbepoetin alfa) and Procrit (epoetin alfa) are not indicated for anemia. If patient does not recover after 28 days of holding drug then the patient must be removed from treatment.

Growth factors (e.g. G-CSF, GM-CSF, erythropoietin, platelet growth factors, etc.) are not to be administered prophylactically but may be prescribed by the treating physician for rescue from severe hematologic events.

b. Hyperlipidemia and Hyperglycemia

Treatment of hyperlipidemia should take into account the pre-treatment status and dietary habits. Blood tests to monitor hyperlipidemia must be taken in the fasting state. Grade 2 or greater hypercholesterolemia (> 300 mg/dL or 7.75 mmol/L) or Grade 2 or greater hypertriglyceridemia (> 300 mg/dL - 500 mg/dL; >3.42 mmol/L- 5.7 mmol/L) should be treated with a 3-hydroxy-3-methyl-glutaryl (HMG)-CoA reductase inhibitor (e.g., atorvastatin, pravastatin) or appropriate lipid-lowering medication, in addition to diet. Patients should be monitored clinically and through serum biochemistry for the development of rhabdomyolysis and other adverse events as required in the product label/data sheets for HMG-CoA reductase inhibitors.

Note: Concomitant therapy with fibrates and an HMG-CoA reductase inhibitor is associated with an increased risk of a rare but serious skeletal muscle toxicity manifested by rhabdomyolysis, markedly elevated creatine phosphokinase (CPK) levels and myoglobinuria, acute renal failure and sometimes death. The risk

SWOG Authorized Document

versus benefit of using this therapy should be determined for individual patients based on their risk of cardiovascular complications of hyperlipidemia.

Grade 3 hyperglycemia has been observed in patients receiving everolimus therapy. The fasting state of patients should be verified when interpreting results. It is suggested that optimal glucose control should be achieved before starting a patient on everolimus and should be monitored during everolimus therapy.

c. Pneumonitis

Pneumonitis is a recognized adverse effect of rapamycins (sirolimus, temsirolimus, and everolimus). Numerous case reports in the literature suggest that rapamycin-associated pneumonitis is relatively unaggressive, limited in extent, and reversible upon drug discontinuation. The term 'pneumonitis' is used here to describe non-infectious, non-malignant infiltration in the lungs which is evident radiologically. More precise diagnosis should follow histocytological examination following lung biopsy, generally during bronchoscopy which may or may not be symptomatic.

Both asymptomatic and symptomatic non-infectious pneumonitis have been noted in patients receiving everolimus. A diagnosis of non-infectious pneumonitis should be considered in patients presenting with non-specific respiratory signs and symptoms such as hypoxia, pleural effusion, cough or dyspnea, and in whom infectious, neoplastic and other non medical causes have been excluded by means of appropriate investigations. Patients should be advised to report promptly any new or worsening respiratory symptoms.

In order to monitor for asymptomatic non-infectious pneumonitis, the regularly scheduled chest CTs for disease surveillance should be assessed for evidence of pneumonitis during protocol treatment. Additional chest CT scans may be performed, when clinically necessary. If non-infectious pneumonitis develops, a consultation with a pulmonologist should be considered.

Management of Non-Infectious Pneumonitis

Worst Grade Pneumonitis	Required Investigations	Management of Pneumonitis	Everolimus Dose Adjustment
Grade 1	CT scans with lung windows and pulmonary function testing including: spirometry, DLCO, and room air O_2 saturation at rest. Repeat chest x-ray/CT scan every 2 Cycles until return to baseline.	No specific therapy is required	Administer same dose of everolimus.
Grade 2	CT scan with lung windows and pulmonary function testing including: spirometry, DLCO, and room air O_2 saturation at rest. Repeat each subsequent Cycle until return to baseline. Consider bronchoscopy	Symptomatic only. Prescribe corticosteroids if cough is troublesome.	Reduce everolimus one dose level until recovery to ≤ Grade 1. Everolimus may also be interrupted if symptoms are troublesome.

SWOG Authorized Document

Worst Grade Pneumonitis	Required Investigations	Management of Pneumonitis	Everolimus Dose Adjustment
	*		Patients will be removed from protocol treatment if they fail to recover to ≤ Grade 1 within 21 days.
Grade 3	CT scan with lung windows and pulmonary function testing including: spirometry, DLCO, and room air O_2 saturation at rest. Repeat each subsequent Cycle until return to baseline. Bronchoscopy is recommended *	Prescribe corticosteroids if infective origin is ruled out. Taper as medically indicated.	Hold treatment until recovery to ≤ Grade 1. May restart protocol at one lower dose level. Patients will be removed from protocol treatment if they fail to recover to ≤ Grade 1 within 21 days.
Grade 4	CT scan with lung windows and required pulmonary function testing includes: spirometry, DLCO, and room air O_2 saturation at rest. Repeat each subsequent Cycle until return to baseline. Bronchoscopy is recommended *.	Prescribe corticosteroids if infective origin is ruled out. Taper as medically indicated.	Discontinue treatment.

*A bronchoscopy with biopsy and/or bronchoalveolar lavage is recommended.

d. Oral Mucositis

In addition to the dose modifications for non-hematological toxicity outlined in Section 8.4, oral mucositis due to everolimus should be treated using local supportive care. Follow the paradigm below for treatment of oral mucositis:

1. For mild toxicity (Grade 1), use conservative measures such as non-alcoholic mouth wash or salt water (0.9%) mouth wash several times a day until resolution.

2. For more severe toxicity (Grade 2 in which case patients have pain but are able to maintain adequate oral alimentation, or Grade 3 in which case patients cannot maintain adequate oral alimentation), the suggested treatments are topical analgesic mouth treatments (i.e., local anesthetics such as benzocaine, butyl aminobenzoate, tetracaine hydrochloride, menthol, or phenol) with or without topical corticosteroids, such as triamcinolone oral paste 0.1% (Kenalog in Orabase®).

3. Agents containing hydrogen peroxide, iodine, and thyme derivatives may tend to worsen mouth ulcers. It is preferable to avoid these agents.

4. Antifungal agents must be avoided unless a fungal infection is diagnosed. In particular, systemic imidazole antifungal agents (ketoconazole, fluconazole, itraconazole, etc.) should be avoided in all patients due to their strong inhibition of everolimus metabolism, thereby leading to higher everolimus exposures. Therefore, topical antifungal agents are preferred if an infection is diagnosed. Similarly, antiviral agents such as acyclovir should be avoided unless a viral infection is diagnosed.

e. Nausea

Routine premedication for nausea is not necessary, but symptomatic patients should be treated with standard antinausea/antiemetic therapy as necessary.

If the patient vomits after taking the tablets, the dose is replaced only if the tablets can actually be seen and counted.

f. Diarrhea

Diarrhea has been seen with everolimus. In general, diarrhea has been transient, usually not of sufficient severity to hinder administration of everolimus and responsive to loperamide. The recommended dose of loperamide is 4 mg at first onset, followed by 2 mg PO q 2-4 hours until diarrhea free for 12 hours.

8.5 Infection Precaution

Everolimus has immunosuppressive properties and may predispose patients to infections, especially those with opportunistic pathogens. Localized and systemic infections, including pneumonia, other bacterial infections and invasive fungal infections, such as aspergillosis, candidiasis, or pneumocystis jirovecii pneumonia (PJP) and viral infections including reactivation of hepatitis B virus, have been described in patients taking everolimus. Some of these infections have been severe (e.g. leading to sepsis; respiratory or hepatic failure) and occasionally have had a fatal outcome. Physicians and patients should be aware of the increased risk of infection with everolimus, be vigilant for symptoms and signs of infection, and institute appropriate treatment promptly.

8.6 Management of Hepatitis Reactivation

In cancer patients with hepatitis B, whether carriers or in chronic state, use of antivirals during anticancer therapy has been shown to reduce the risk of hepatitis B virus (HBV) reactivation and associated HBV morbidity and mortality. *(Loomba et al. 2008)*

Monitoring and prophylactic treatment for hepatitis B reactivation
Table 1 provides details of monitoring and prophylactic therapy according to the baseline results of viral load and serologic markers testing.

Table 1 Action to be taken for positive baseline hepatitis B results

Test	Result	Result	Result	Result	Result
HBV-DNA	+	+ or -	-	-	-
HBsAg	+ or -	+	-	-	-
HBs Ab	+ or -	+ or -	**+**<br><br>and no prior HBV	+ or -	-<br><br>or + with prior HBV

Test	Result	Result	Result	Result	Result
			vaccination		vaccination
HBc Ab	+ or -	+ or -	+ or -	+	-
Recommendation	Prophylaxis treatment should be started 1-2 weeks prior to first dose of study drug<br>Monitor HBV-DNA approximately every 4-6 weeks		No prophylaxis<br>Monitor HBV-DNA approximately every 4-6 weeks		No specific action

Antiviral prophylaxis therapy should continue for at least 4 weeks after last dose of study drug.

For hepatitis B reactivation, definition and management guidelines, see Table 2 Guidelines for management of hepatitis B.

Table 2 Guidelines for management of hepatitis B

HBV reactivation (with or without clinical signs and symptoms)*	
For patients with baseline results:<br>Positive HBV-DNA<br>OR<br>positive HBsAg<br>-------------------------------<br>reactivation is defined as:<br>[Increase of 1 log in HBV-DNA relative to baseline HBV-DNA value OR new appearance of measurable HBV-DNA]<br>AND<br>ALT elevation x 5 ULN	Treat: Start a second antiviral<br>AND<br>Interrupt study drug administration until resolution:<br>≤ Grade 1 ALT (or baseline ALT, if > Grade 1) and<br>≤ baseline HBV-DNA levels<br>If resolution occurs within ≤ 28 days study drug should be re-started at one dose lower, if available. If the patient is already receiving the lowest dose of study drug according to the protocol, the patient should restart at the same dose after resolution. Both antiviral therapies should continue at least 4 weeks after last dose of study drug.<br>If resolution occurs > 28 days Patients should discontinue study drug but continue both antiviral therapies at least 4 weeks after last dose of study drug.
For patients with baseline results: Negative HBV-DNA and HBsAg<br>AND<br>[Positive HBs Ab (with no prior history of vaccination against HBV), OR positive HBc Ab]<br>-------------------------------	Treat: Start first antiviral medication<br>AND<br>Interrupt study drug administration until resolution:<br>≤ baseline HBV-DNA levels<br>If resolution occurs within ≤ 28 days study drug should be re-started at one dose lower, if available. If the patient is already receiving the lowest dose of study drug according to the protocol, the patient should restart at the same dose after resolution. Antiviral therapy should continue at least 4 weeks after last dose of study drug.

SWOG Authorized Document

HBV reactivation (with or without clinical signs and symptoms)*	
reactivation is defined as:<br><br>New appearance of measurable HBV-DNA	If resolution occurs > 28 days Patients should discontinue study drug but continue antiviral therapy at least 4 weeks after last dose of study drug.

* All reactivations of hepatitis B are to be recorded as Grade 3 (CTCAE v 4.0 Infections and Infestations: Hepatitis Viral), unless considered life threatening by the investigator; in which case they should be recorded as Grade 4 (CTCAE 4.0 Infections and Infestations: Hepatitis Viral). Date of viral reactivation is the date on which **both** DNA and ALT criteria were met (e.g. for a patient who was HBV-DNA positive on 01-JAN-10 and whose ALT reached ≥ 5 × ULN on 01-APR-10, the date of viral reactivation is 01-APR-10).

Monitoring for Hepatitis C

The following two categories of patients should be monitored every 4-6 weeks for HCV reactivation:

- Patients with detectable HCV RNA-PCR test at baseline.
- Patients known to have a history of HCV infection, despite a negative viral load test at baseline (including those that were treated and are considered 'cured')

For definition of hepatitis C reactivation and the management guidelines, see Table 3 Guidelines for management of hepatitis C.

Table 3 Guidelines for management of hepatitis C

HCV reactivation*	
For patients with baseline results:<br>Detectable HCV-RNA,<br><br>reactivation is defined as:<br>ALT elevation x 5 ULN	Discontinue study drug
For patients with baseline results:<br>Knowledge of past hepatitis C infection with no detectable HCV-RNA,<br><br>reactivation is defined as:<br>New appearance of detectable HCV-RNA	Discontinue study drug

* All reactivations of hepatitis C are to be recorded as Grade 3 (CTCAE 4.0 Infections and Infestations: Hepatitis Viral), unless considered life threatening by the investigator; in which case they should be recorded as Grade 4 (CTCAE 4.0 Infections and Infestations: Hepatitis Viral).

8.7 Emergency Unblinding

For emergency unblinding guidelines see Section 18.2.

8.8 Dose Modification Contacts

For treatment or dose modification related questions, please contact Dr. Christopher Ryan at 503/494-8487 or Dr. Elisabeth Heath at 313/576-8715.

8.9 Adverse Event Reporting

Toxicities (including suspected reactions) that meet the expedited reporting criteria as outlined in Section 16.0 of the protocol must be reported to the Operations Office, Study Chair, the NCI via CTEP-AERS, and to the IRB per local IRB requirements.

SWOG Authorized Document

9.0 STUDY CALENDAR

		Cycle 1						Cycle 2		Cycle 3 §				√
**REQUIRED STUDIES**	PRE	WK	WK	WK	WK	WK	WK	WK	WK	WK	WK	Off Treat F/U Prior to Recurrence	Recurrence	Off Treat F/U After Recurrence
	STUDY	1	2	3	4	5	6	7 ------->12		13 ------->18				
**PHYSICAL**														
History and Physical Exam	X*	XЯ			X			X		X		X	X	X
Hepatitis B/C Screening †	X													
Weight and Performance Status	X	XЯ						X		X				
Recurrence Assessment ¶ Ж											X	X¶	X	
Toxicity Notation Ω		X	X	X	X	X	X	X		X				
Review Intake Calendar								X		X				
**LABORATORY**														
WBC/Differential /Platelets	X							X		X				
Hemoglobin	X							X		X				
Bilirubin	X							X		X				
SGOT and SGPT	X							X		X				
Serum Creatinine or Calculated Creatinine Clearance	X							X		X				
Alkaline Phosphatase	X							X		X				
Fasting Glucose, Cholesterol and Triglycerides	X							X		X				
HBV DNA or HCV RNA-PCR Δ								X		X				

Study Calendar continued on next page. Click here for footnotes.

		Cycle 1						Cycle 2		Cycle 3 §				√
**REQUIRED STUDIES**	PRE STUDY	WK	WK	WK	WK	WK	WK	WK	WK	WK	WK	Off Treat F/U Prior to Recurrence	Recurrence	Off Treat F/U After Recurrence
		1	2	3	4	5	6	7 ------->12		13 ------->18				
**SPECIMEN SUBMISSION ¥**														
Archived Tumor Specimen	X													
Buffy Coat and Plasma Specimens	X							X		X		X	X	
Whole Blood for Trough Levels	X							X		X		X		
**X-RAYS AND SCANS**														
CT Scan of Chest/Abdomen/Pelvis £ Ж	X										X £ Ж	X	X	
Bone Scan Ħ	XĦ											XĦ		
**TREATMENT** (see Section 7.0 for details)														
Everolimus/Placebo		X -->X												

Click here for footnotes.

FOOTNOTES:

NOTE: Forms are found in Section 18.0**. Forms submission guidelines may be found in** Section 14.0**.**

* Physical exam and medical history must be completed within 28 day prior to registration (see Section 5.3a).

Δ Only for patients with positive HBV or HCV studies at baseline (see Section 8.6).

† An assessment of Hepatitis B/C medical history and risk factors must be done at screening (see Section 5.3h). Patients with risk factors for Hepatitis B should undergo HBV DNA, HBsAg, HBsAb, and HBcAb testing (see Sections 7.1, 7.3 and 8.6). Patients with risk factors for Hepatitis C infection should undergo HCV RNA-PCR testing (see Sections 7.1 and 8.6)

§ Protocol treatment will continue at the same intervals indicated for Cycle 3 for a maximum of 9 cycles (54 weeks) or until the patient has met any of the criteria listed in Section 7.6.

√ After recurrence, follow-up will occur (with lab tests and scans performed at the discretion of the treating physician) every 6 months for the first two years and then yearly thereafter until 10 years after registration.

£ CT scans of the chest must also be assessed for radiographic evidence of pneumonitis (see Section 8.4c).

Ж Scan (CT scan of the chest, abdomen and pelvis with IV and oral contrast; CT scan of chest without contrast and MRI of abd/pelvis with gadolinium) and recurrence assessment must be performed every 4 months (18 weeks) during Year 1 (regardless of early protocol treatment discontinuation), every 6 months for Years 2 and 3, and yearly thereafter until recurrence, death, or 10 years after registration whichever comes first.
The surveillance imaging therapy intervals prescribed here are considered standard of care by expert consensus (SWOG Genitourinary Committee) based on the high risk of cancer recurrence in the patients enrolled in this study. Non-contrast CT of the chest/abdomen/pelvis should only be used for surveillance imaging if, in the opinion if the investigator, it is in the best medical interest of the patient to not receive IV contrast of any form.
NOTE: PET/CT is not an acceptable imaging alternative.

¥ See Section 15.0. If patient has consented to banking for the translational medicine component, it is very important to obtain whole blood for trough levels and buffy coat and plasma specimens from any patient that is being removed from protocol treatment, regardless of the time point. DO NOT COLLECT SPECIMENS AT EACH VISIT DURING FOLLOW-UP. Additionally, specimens for buffy coat and plasma specimens should be collected at disease recurrence. **Specimens must be drawn prior to daily dose of study drug.**

Ω Once everolimus treatment has been initiated, weekly toxicity assessments are required during the first cycle. Toxicity assessments during the first cycle may be performed via a phone call during weeks when a physical exam is not required.

Ħ Bone scan recommended if patient has rising alkaline phosphatase or bone pain.

Я If prestudy history and physical exam, weight and performance status are obtained within 28 days prior to registration, they do not need to be repeated for Cycle 1, Day 1.

¶ If patient is taken off protocol treatment due to toxicity or other reason besides recurrence, they must continue to be followed for recurrence with physical exams and scans every 4 months (18 weeks) during Year 1 (regardless of early protocol treatment discontinuation), every 6 months for Years 2 and 3, and yearly thereafter until recurrence, death, or 10 years after registration whichever comes first.

10.0 CRITERIA FOR EVALUATION AND ENDPOINT DEFINITIONS

10.1 Recurrence

See Section 9.0 for recurrence assessment schedule.

The following are the only acceptable criteria for renal cell cancer recurrence. Anything not listed as acceptable should not be an indication to alter protocol therapy. Any recurrence of malignant disease should be proven by tissue biopsy whenever possible. Supporting documentation must be submitted following diagnosis of renal cell carcinoma recurrence.

The following criteria of treatment failure constitute the only acceptable evidence of disease recurrence. Supporting documentation includes a copy of radiology and pathology reports.

Lung: (i) positive cytology or biopsy in the presence of a solitary lesion, (ii) radiologic evidence of multiple lesions felt to be consistent with metastases. (iii) neoplastic pleural effusion should be established by cytology or pleural biopsy.

Liver: (i) positive cytology or biopsy (ii) multiple new focal defects on MRI scan, CT or ultrasound that are enlarging in size as documented by repeat scans. (iii) proof of neoplastic abdominal ascites should be established by cytology or pleural biopsy.

Central Nervous System: positive brain CT scan or MRI scan or cerebral spinal fluid cytology.

Subcutaneous and Lymph Node Recurrence: (i) positive biopsy (ii) progressively enlarging solid mass or node(s) as evidenced by repeat CT or MRI scans (iii) ureteral obstruction in the presence of a mass as documented on CT or MRI scan.

Other Organs: (i) positive radiographic study and biopsy/aspiration cytology (ii) progressively enlarging solid mass or node(s) as evidenced by repeat CT or MRI scans.

Renal Bed: (i) positive radiographic study and biopsy/aspiration cytology (ii) progressively enlarging solid mass or node(s) as evidenced by two CT or MRI scans separated by at least a 4 week interval.

Skeletal: (i) positive radiographic study such as bone scan, (ii) for a solitary lesion or equivocal finding on scan, a biopsy is required to demonstrate recurrence, (iii) MRI or CT of solitary or equivocal lesion seen on bone scan that confirms metastasis is also acceptable.

10.2 Symptomatic deterioration

Global deterioration of health status requiring discontinuation of treatment without objective evidence of progression. Efforts should be made to obtain objective evidence of progression after discontinuation.

SWOG Authorized Document

10.3 Performance Status

Patients will be graded according to the Zubrod performance status scale.

**POINT**	**DESCRIPTION**
0	Fully active, able to carry on all pre-disease performance without restriction.
1	Restricted in physically strenuous activity but ambulatory and able to carry out work of a light or sedentary nature, e.g., light housework, office work.
2	Ambulatory and capable of self-care but unable to carry out any work activities; up and about more than 50% of waking hours.
3	Capable of limited self-care, confined to bed or chair more than 50% of waking hours.
4	Completely disabled; cannot carry on any self-care; totally confined to bed or chair.

10.4 Recurrence-Free Survival

From date of registration to date of first documentation of recurrence outlined in Section 10.1 or death due to any cause. Patients last known to be alive and recurrence-free are censored at date of last contact.

10.5 Time to Death

From date of registration to date of death due to any cause. Patients last known to be alive are censored at date of last contact.

11.0 STATISTICAL CONSIDERATIONS

11.1 Accrual Goal

ECOG's ASSURE trial randomized on average, 480 patients per year (40 per month). To be slightly more conservative, we anticipate that 28 patients per month (336 per year) will be randomized to this study. Based on the distribution of risk groups observed on **E2805** including half of the patients falling into the very-high risk group, it will be assumed that the placebo arm will produce a median recurrence-free survival of 4.9 years. **E2805** has also experienced a significant proportion of patients who have not completed the one year of protocol-specified treatment. Based on data from a recent amendment to **E2805**, twenty-five percent of patients have withdrawn from treatment on this study within the first three months. Although we expect everolimus to be better tolerated than sorafenib or sunitinib, we would estimate that about 15% of patients will stop protocol treatment within the first three months. Using a weighted average of the log hazard rates to obtain the effective hazard rate for the treatment arm under the alternative hypothesis, the ideal hazard ratio (HR) of 0.75 becomes an effective hazard ratio of 0.78 when lack of compliance is considered. Assuming exponential survival, 3.5 years of patient accrual, four additional years of follow-up and a sample size of 1170 patients (585 experimental, 585 placebo), the study has 85% power to detect a 22% reduction in the hazard rate of recurrence-free survival with everolimus compared to placebo (hazard ratio 0.78). This corresponds to an improvement in the median RFS from 4.9 to 6.3 years if the RFS

SWOG Authorized Document

distributions are exponential. We will use a one sided stratified log rank test with α = 0.025.

July 2015 Revision: As we now have a large amount of data to evaluate the rate of compliance to everolimus, we see that our original assumption of a 15% non-compliance rate was an underestimate. We propose to adjust the study design based on the observed compliance data from this trial in consultation with the study Data and Safety Monitoring Committee, and based on the following assumptions: those who receive less than 3 months of study drug have the same risk of recurrence as the placebo group, and those who receive 3-6 months of study drug have half the treatment benefit as those who receive more than six months.

We still assume an ideal HR=0.75, but we increase the accrual duration by one year to 4.5 years, and keep 4 years of follow-up. We are also reducing the statistical power from 85% to 80% in order to achieve a sample size that seems achievable within a reasonable amount of time. By making the prior assumptions about treatment efficacy, we will have an effective HR of 0.82 based on the proportion of dropouts and when they became noncompliant.

Assuming exponential recurrence-free survival, 4.5 years of patient accrual, four additional years of follow-up and an updated sample size of 1464 eligible patients (732 per arm), the study has 80% power to detect a 18% reduction in the hazard rate of recurrence free survival with everolimus compared to placebo (HR=0.82). This corresponds to an improvement in the median RFS from 4.9 to 6.0 years if the RFS distributions are exponential. We will use a one sided stratified log rank test with alpha=0.025.

11.2 Analysis of Primary Endpoint

This study's design is based on assumptions about the accrual rate, the distribution of intermediate-high and very-high risk patients that have been randomized on ASSURE and histology. The total projected accrual adjusted for a 5% ineligibility rate is 1,537 patients (previously 1,218). If accrual to the intermediate-high risk strata reaches 50% of the total projected accrual then the stratum will be closed to further accrual. If accrual to the clear cell histology stratum reaches 85% of the total projected accrual then the stratum will be closed to further accrual. This will ensure accrual patterns in the strata with high event rates are comparable to those assumed in the study design. If the event rate information becomes available from ASSURE, the study team may consider recommending that the sample size be reevaluated. The sample size adjustment would be based on baseline characteristics. Only the number of patients accrued would possibly change, but the number of events and formal analysis timepoints, which are based on number of events, would not change.

The first interim analysis will be conducted after approximately 1,000 patients have been entered (n=500 per arm), approximately 4 years after the study opens and when 30% of the expected number of recurrences have occurred on the placebo arm. A second interim analysis will be conducted approximately 5 years after the study opens and when 50% of the expected events on the placebo arm have been observed. The third interim analysis will be conducted when 70% of the expected events on the placebo arm have been observed. The fourth interim analysis will occur approximately 7.5 years after study activation when 90% of the expected events on the placebo arm have been observed. For each interim analysis, timing of analysis will be based on percent of expected information and not on calendar time. Evidence suggesting early termination of the trial and a conclusion that everolimus is not better than placebo would be if the alternative hypothesis of a 22% improvement in recurrence-free survival with the experimental arm is rejected at the 0.002 level. In addition, the null hypothesis of no difference in recurrence-free survival will be tested at the one-sided level of 0.001. If the decision is to continue the study to completion, the final analysis will be done after complete accrual

SWOG Authorized Document

and approximately four years of follow-up at complete information when 804 total events have occurred across both arms. A stratified, one-sided log-rank test will be used for testing the primary hypothesis at the 0.022 level to account for interim testing.

December 2020 Update:

The expected endpoint rate in the placebo arm is lower than anticipated. Although three interim analyses have been conducted to date, the number of events is slowing making it difficult to reach the targeted number of events. Therefore, we consider some modification to the statistical analysis plan. This plan was developed by two independent biostatisticians who had not seen and had no access to endpoint data for this trial, with medical input from the primary study chair. The SWOG Data and Safety Monitoring Committee reviewed and approved this revision

The median recurrence-free survival of ASSURE (6.6 years) was longer than the six years assumed for this trial so the actual number of events in the placebo arm may be less than originally projected.

To keep power at 80% under current trial conditions, we continue to use the 4.5 years of accrual but allow 6 additional years of follow-up after the last accrual for a total of 10.5 years. Assuming a more conservative control arm hazard rate of 0.1027 (median RFS=6.75 years), the expected date of data maturity (804 total events) under the alternative hypothesis is September 2021. However, the necessary 804 events may never be attained because of drop out or failure of the underlying exponential distribution assumption and so the final analysis is expected in March 2022 even if 804 events have not been observed. In addition, the 4th and final interim analysis should be conducted one year prior in March 2021 regardless of the percent of expected information. The futility analysis is being changed from a one-sided 0.001 to a 1-sided value of 0.025 in the last interim analysis. This will have minimal effect on power if the true hazard ratio is 0.82 but would lead to earlier reporting if the observed hazard ratio is close to 1.0.

Updated Timing of Scheduled Analyses on <u>**S0931**</u>, **December 2020 Update**

Analysis	Estimated Study Time	# of Events on Placebo Arm* (% of total expected)	# of Events on Everolimus Arm (assuming Ha)	Testing Ho, Level of significance	Testing Ha, Level of significance
1st interim analysis	4 years after activation	119 (30%)	104	.002	.001
2nd interim	5 years after activation	208 (50%)	183	.002	.001
3rd interim	6 years after activation	297 (70%)	264	.002	.001
4th interim		March 2021		.002	.025
Final analysis		422 (100%)#	382#	0.022	N/A

* timing of all planned analyses is based on the number of events in this column
# timing of final analysis at 804 total events or a maximum time of March 2022

11.3 Analysis of Secondary Endpoints

Secondary analyses will be performed for comparison between the two arms regarding survival and adverse events. With 732 patients on each arm, toxicity rates can be estimated to within at worst ± 3.7% (95% confidence interval). Additional exploratory analyses will include assessing the treatment effect within subgroups defined by the stratification factors as well as the interaction of everolimus with each of the stratification

factors, and whether the surgery was partial or a radical nephrectomy. With at least 15% of the patients having non-clear cell histology, for this subgroup the difference in the survival rates between the treatment arms can be estimated to within at worst ± 5% (95% confidence interval).

11.4 Analysis of Translational Medicine Endpoints

(A) Prognostic significance: Proportional hazards regression will be used to evaluate the association between genes and pathways and recurrence-free survival and overall survival. Additional exploratory methodology may include classification and regression trees or logic regression to identify combinations of markers that may be associated with the endpoints of interest. The following table shows the minimally detectable RFS hazard ratio for various prevalence of markers and rates of sample submission with 80% statistical power and a two-sided alpha=0.05. This table assumes no treatment by marker interaction, and a median RFS of 4.9 years in those with the biomarker of interest.

Minimally Detectable Hazard Ratios for Recurrence-Free Survival

	sample submission (n=1170)	sample submission (n=936)	sample submission (n=702)
10% prev of a SNP	1.46	1.52	1.63
20% prev. of a SNP	1.33	1.38	1.45
30% prev. of a SNP	1.28	1.32	1.38

(B) Predictive significance: In order to evaluate whether molecular biomarkers in the AKT/mTOR pathway are predictive factors for everolimus, we will assess the interaction of the biomarker with treatment in a proportional hazards regression model assessing RFS. The entry in the following table is the minimally detectable ratio of the HR's of placebo/everolimus for those without the pathway activation relative to those that do have the activation. For simplicity of calculations, we assume that there is no prognostic association of biomarker activation and RFS in the placebo group, and the treatment effect for those without activation is null. That is, everolimus only has an impact on RFS among those with marker activation.

Minimally Detectable Interaction for Recurrence-Free Survival

Table entry is the hazard ratio of (Everolimus/Placebo) for those without marker activation relative to the HR of (Ever/Pl) for those with marker activation, assuming 80% statistical power and a two-sided α=0.05			
	100% sample submission	80% sample submission	60% sample submission
10% prev. of a marker	2.40	2.75	3.49
20% prev. of a marker	1.85	2.00	2.28
30% prev. of a marker	1.68	1.80	1.99

If 936 of patients randomized to this trial provide a usable tissue sample for these translational objectives, and the prevalence of the biomarker of interest is 20%, then we will have reasonable power (80%) to detect a hazard ratio of approximately 0.50 for those with a marker compared to a HR of 1.0 for those without the marker (1.0/0.50 = 2.00). Frequency and severity of adverse events and proximity to sample collection will be correlated with steady-state C_{min}. Assuming 90% of those randomized to everolimus will submit one or more serum samples, we will have approximately 526 subjects to analyze.

SWOG Authorized Document

Initially, correlations of adverse events and the C_{min} in closest proximity to the adverse event will be explored in a manner analogous to multiple t-tests. Logistic regression modeling will be used to assess whether Cmin is a predictor for the presence of the adverse events after adjusting for other patient and disease covariates. Proportional odds (PO) models may be used to model severity of an adverse event in ordinal categories, if the PO modeling assumption is supported by the data.

11.5 Pharmacokinetic Analysis of Blood Samples

For the pharmacokinetic analysis of blood samples, we will use blood samples that were collected at Week 7 or Week 13 and the patient must have been randomized to the everolimus arm and be receiving protocol treatment at the time of the blood draw. At the time of the on-study analysis in the winter of 2014, there were 83 patients with one blood draw and 165 with two for a total of 248 patients. For those with two on-treatment measurements, the average of the two will be used for analysis to reduce measurement error bias.

The purpose of the analysis is to correlate everolimus blood concentrations from early blood draws with the experience of adverse events by the patients, both in terms of general grades of toxicities but also by specific adverse events that have been more frequently reported on the everolimus arm. The following calculations assume a PK analysis will be conducted prior to accrual completion in order to gain insight into the relationship between blood levels of the study drug and adverse events. We anticipate there will be more samples to evaluate later in the trial and so effect sizes will be smaller with larger sample sizes.

Most Prevalent Grade 2-4 Adverse Events in the Everolimus Arm of EVEREST

Among all Randomized Patients on the Everolimus Arm Who Received Any Study Drug

**Toxicity**	**% of Evaluable with Grade 2-4**
High Cholesterol	10%
Fatigue	19%
Hyperglycemia	14%
Hypertriglyceridemia	27%
Oral mucositis	35%
Rash	14%
**Composite Endpoints**	
Any Toxicity Grade 2-4	87%
Any Toxicity Grade 3-4	46%
Refusal/Tox within first 3 months	25%
Refusal/Tox within first 6 months	41%
Refusal/Tox prior to 12 months	52%

In order to simplify power calculations related to everolimus blood concentrations, we will assume the distribution of values will be divided into quartiles. The following table indicates the minimally detectable odds ratio that could be detected between the lowest and highest quartile in terms of predicting an adverse event. A range of prevalences are illustrated below to show how the effect size changes with the prevalence of the adverse event.

Minimally Detectable Odds Ratio for Highest versus Lowest Quartile of everolimus in the blood, assuming 80% Statistical Power and a Two-sided Alpha=0.05.

	Prevalence of Adverse Event Endpoint				
	10%	20%	30%	40%	50%

Q4 vs. Q1 of everolimus concentration	6.5	3.6	3.0	2.7	2.7

An individual adverse event that is only reported by 10% of patients who receive everolimus will need to have a very strong association with concentration of everolimus in the blood (odds ratio 6.5 between highest and lowest concentration) in order to be detected as a significant association. An association of any Grade 3-4 toxicity only needs to be in the range of an odds ratio of 2.7 to be detectable.

For patients who have had a dose reduction of protocol therapy at the time of their blood draw, we will handle this in two ways. First, we will just analyze the everolimus level as it appears in the blood and ignore the dose received, and the second approach will standardize the amount of everolimus in the blood conditional on the dose of drug taken.

Logistic regression will be used to model adverse event outcomes with everolimus blood concentration as a predictor, adjusting for other stratification factors and age, gender and race.

11.6 Data and Safety Monitoring Committee Oversight

A Data and Safety Monitoring Committee will oversee the conduct of the study. The Committee consists of four members from outside of SWOG, 3 SWOG members, 3 non-voting representatives from the National Cancer Institute (NCI), and the Group Statistician (non-voting). The members of this Committee will receive confidential reports every 6 months from SWOG Statistical Center, and will meet at the Group's bi-annual meetings as necessary. The Committee will be responsible for decisions regarding possible termination and/or early reporting of the study.

12.0 DISCIPLINE REVIEW

There will be no formal discipline review done in conjunction with this study.

13.0 REGISTRATION GUIDELINES

13.1 Registration Timing

Patients must be registered prior to initiation of treatment (no more than ten working days prior to planned start of treatment).

13.2 Investigator/Site Requirements

This study is supported by the NCI Cancer Trials Support Unit (CTSU).

Prior to the recruitment of a patient for this study, investigators must be registered members of a Cooperative Group. Each investigator must have an NCI investigator number and must maintain an “active” investigator registration status through the annual submission of a complete investigator registration packet (FDA Form 1572 with original signature, current CV, Supplemental Investigator Data Form with signature and Financial Disclosure Form with original signature) to the Pharmaceutical Management Branch, CTEP, DCTD, NCI. These forms are available on the CTSU Web site (http://ctep.cancer.gov/investigatorResources/investigator_registration.htm).Questions should be directed to the CTEP Investigator Registration Help Desk by e-mail at pmbregpend@ctep.nci.nih.gov.

SWOG Authorized Document

Each investigator or group of investigators at a clinic site must obtain IRB approval for this protocol and submit IRB approval and supporting documentation to the CTSU Regulatory Office before they can enroll patients. Study centers can check the status of their registration packets by querying the Regulatory Support System (RSS) site registration status page of the CTSU member web site by entering credentials at https://www.ctsu.org.

Requirements for site registration:

- CTSU IRB Certification
- CTSU IRB/Regulatory Approval Transmittal Sheet

Note: Sites participating on the NCI CIRB initiative and accepting CIRB approval for the study are not required to submit separate IRB approval documentation to the CTSU Regulatory Office for initial, continuing or amendment review. This information will be provided to the CTSU Regulatory Office from the CIRB at the time the site's Signatory Institution accepts the CIRB approval. The Signatory site may be contacted by the CTSU Regulatory Office or asked to complete information verifying the participating institutions on the study. Other site registration requirements (i.e., laboratory certifications, protocol-specific training certifications, or modality credentialing) must be submitted to the CTSU Regulatory Office or compliance communicated per protocol instructions.

13.3 OPEN Registration Requirements

The individual registering the patient must have completed the appropriate SWOG Registration Worksheet. The completed form must be referred to during the registration but should not be submitted as part of the patient data.

Oncology Patient Enrollment Network (OPEN) will also ask additional questions that are not present on the SWOG Registration Worksheet. The individual registering the patient must be prepared to provide answers to the following questions:

a. Institution CTEP ID

b. Protocol Number

c. Registration Step

d. Treating Investigator

e. Cooperative Group Credit

f. Credit Investigator

g. Patient Initials

h. Patient's Date of Birth

i. Patient SSN (SSN is desired, but optional. Do not enter invalid numbers.)

j. Country of Residence

k. ZIP Code

l. Gender (select one):

- Female Gender
- Male Gender

SWOG Authorized Document

m. Ethnicity (select one):

- Hispanic or Latino
- Not Hispanic or Latino
- Unknown

n. Method of Payment (select one):

- Private Insurance
- Medicare
- Medicare and Private Insurance
- Medicaid
- Medicaid and Medicare
- Military or Veterans Sponsored NOS
- Military Sponsored (Including Champus & Tricare)
- Veterans Sponsored
- Self Pay (No Insurance)
- No Means of Payment (No Insurance)
- Other
- Unknown

o. Race (select all that apply):

- American Indian or Alaska Native
- Asian
- Black or African American
- Native Hawaiian or other Pacific Islander
- White
- Unknown

13.4 Registration Procedures

a. All site staff (SWOG and CTSU Sites) will use OPEN to enroll patients to this study. OPEN is a web-based application that is integrated with the CTSU Enterprise System for regulatory and roster data and, at the time of patient registration, initializes the patient in the Rave database. OPEN can be accessed at https://open.ctsu.org or from the OPEN tab on the CTSU members' side of the website at https://www.ctsu.org, or from the OPEN Patient Registration link on the SWOG CRA Workbench.

b. Prior to accessing OPEN site staff should verify the following:

- All eligibility criteria have been met within the protocol stated timeframes and the affirmation of eligibility on the Registration Worksheet has been signed by the registering investigator or another investigator designate. Site staff should refer to Section 5.0 to verify eligibility.

- All patients have signed an appropriate consent form and HIPAA authorization form (if applicable).

- The study site is listed as "approved" in the CTSU RSS.

c. Access requirements for OPEN:

- Site staff will need to be registered with CTEP and have a valid and active CTEP-IAM account. This is the same account (user ID and password) used for the CTSU members' web site. Additional information about obtaining a CTEP-IAM account can be found at

http://ctep.cancer.gov/branches/pmb/associate_registration.htm. Questions should be directed to the CTEP Associate Registration Help Desk by e-mail at ctepreghelp@ctep.nci.nih.gov.

- To perform registrations, the site must have been assigned the 'Registrar' role on the SWOG or CTSU roster:

1. If you are a SWOG member, to perform registrations on SWOG protocols you must have an equivalent 'Registrar' role on the SWOG roster. Role assignments are handled through SWOG.

2. If you are not a SWOG member, to perform registrations on SWOG protocols you must have the role of Registrar on the CTSU roster. Site and/or Data Administrators can manage CTSU roster roles via the new Site Roles maintenance feature under RSS on the CTSU members' web site. This will allow them to assign staff the "Registrar" role.

Note: The OPEN system will provide the site with a printable confirmation of registration and treatment information. Please print this confirmation for your records.

d. Further instructional information is provided on the OPEN tab of the CTSU members' side of the CTSU website at https://www.ctsu.org or at https://open.ctsu.org. For any additional questions contact the CTSU Help Desk at 1-888-823-5923 or ctsucontact@westat.com.

13.5 Exceptions to SWOG registration policies will not be permitted.

a. Patients must meet all eligibility requirements.

b. Institutions must be identified as approved for registration.

c. Registrations may not be cancelled.

d. Late registrations (after initiation of treatment) will not be accepted.

14.0 DATA SUBMISSION SCHEDULE

14.1 Data Submission Requirement

Data must be submitted according to the protocol requirements for **ALL** patients registered, whether or not assigned treatment is administered, including patients deemed to be ineligible. Patients for whom documentation is inadequate to determine eligibility will generally be deemed ineligible.

14.2 Master Forms

Master forms are included in Section 18.0 and (with the exception of the sample consent form and the Registration Worksheet) must be submitted to the Data Operations Center in Seattle. Data from approved SWOG institutions must be submitted on-line via the Web; see Section 14.3a for details. Exceptions to online data submission are patient-completed (e.g. Quality of Life) forms and source documents (e.g. pathology/operative/lab reports).

14.3 Data Submission Procedures

a. SWOG institutions must submit data electronically via the Web by using the SWOG CRA Workbench. To access the CRA Workbench, go to the SWOG Web site (http://swog.org) and logon to the Members Area. After you have logged on, click on the *CRA Workbench* link to access the home page for CRA Workbench website. Next, click on the *Data Submission* link and follow the instructions. For new users, the link to a "Starter Kit" of help files may be found by clicking on the **Starter Kit** link at the Members' logon page.

To submit data via the web the following must be done (in order):

1. You are entered into the SWOG Roster and issued a SWOG Roster ID Number,
2. You are associated as an investigator or CRA/RN at the institution where the patient is being treated or followed, and
3. Your Web User Administrator has added you as a web user and has given you the appropriate system permissions to submit data for that institution.

For assistance with points 1 and 2 call the Operations Office at 210/614-8808. For point 3, contact your local Web User Administrator (refer to the "Who is my Web User Administrator?" function on the swog.org Members logon page). For other difficulties with the CRA Workbench, please email *technicalquestion@crab.org*.

b. If you need to submit data that are not available for online data submission, the only alternative is via facsimile. Should the need for this occur, institutions may

submit data via facsimile to 800/892-4007 or 206/342-1680 locally. Please do not use cover sheet for faxed data. Please make sure that each page of all faxed data include the SWOG patient number, study ID and patient initials.

c. Data Submission Instructions for non-SWOG Institutions (CTSU): See the CTSU participation table.

14.4 Data Submission Overview and Timepoints

a. WITHIN 7 DAYS OF REGISTRATION:

Submit copies of the following:

S0931 Prestudy Form

S0931 Tumor Specimen Processing Form (for patients who consent to the translational medicine studies)

Surgical and Radiology Reports

Pathology Report confirming renal cell carcinoma including subtypes.

b. IF PATIENT CONSENTED:

Submit specimens within 28 days of obtaining as outlined in Section 15.0.

c. WITHIN 7 DAYS OF COMPLETION OF EACH CYCLE (EVERY SIX WEEKS) WHILE ON PROTOCOL TREATMENT:

S0931 Treatment Form

S0931 Adverse Event Form

d. EVERY EIGHTEEN WEEKS FOR THE FIRST 54 WEEKS (REGARDLESS OF EARLY PROTOCOL TREATMENT DISCONTINUATION), AND THEN EVERY 6 MONTHS FOR THE FOLLOWING 2 YEARS, AND THEN ANNUALLY UNTIL 10 YEARS AFTER REGISTRATION, RECURRENCE OR DEATH WHICHEVER COMES FIRST:

S0931 Recurrence Assessment Form

e. WITHIN 14 DAYS OF DISCONTINUATION OF ALL PROTOCOL TREATMENT:

Off Treatment Notice

Final **S0931** Treatment Form

Final **S0931** Adverse Event Form

f. AFTER OFF ALL PROTOCOL TREATMENT, EVERY SIX MONTHS FOR TWO YEARS AND ANNUALLY UNTIL TEN YEARS AFTER REGISTRATION OR UNTIL DEATH:

Submit copies of the **S0931** Specific Follow Up Form

SWOG Authorized Document

g. WITHIN 14 DAYS OF RECURRENCE:

S0931 Adverse Event Form

Off Treatment Notice

If patient was off protocol treatment:

S0931 Specific Follow-Up Form

For all patients:

S0931 Recurrence Assessment Form

Radiology and pathology reports documenting recurrence.

h. WITHIN 4 WEEKS OF KNOWLEDGE OF DEATH:

If patient was still on protocol treatment:

Notice of Death

Final S0931 Treatment Form

Final S0931 Adverse Event Form

If patient was off protocol treatment:

Notice of Death

S0931 Specific Follow-Up Form

15.0 SPECIAL INSTRUCTIONS

15.1 Tissue Specimen Banking

Tissue specimens for banking and future translational medicine studies will be submitted to the SWOG Specimen Repository – Solid Tissue, Myeloma and Lymphoma Division, Lab #201. Specimen banking is optional for the patient.

a. With patient's consent tissue specimens must be submitted at the timepoint listed below in Section 15.1b (see Section 9.0):

b. A paraffin embedded block of tumor or 10 unstained slides obtained from the prestudy nephrectomy specimen must be submitted to Lab #201 within 7 days of registration. This specimen will be banked until funding is obtained to perform translational medicine studies which will include assessing molecular biomarkers relevant to the AKT/mTOR and other pathways implicated in the pathogenesis of renal carcinoma and investigating their potential predictive and prognostic value.

Specimen collection and submission instructions can be accessed on the SWOG Specimen Submission webpage (http://swog.org/Members/ClinicalTrials/Specimens/STSpecimens.asp), or via the link on the **S0931** protocol abstract page on the SWOG website (www.swog.org).

c. Specimen collection kits are not being provided for this submission; sites will use institutional supplies.

SWOG Authorized Document

15.2 Plasma, Buffy Coat, and Whole Blood Specimen Banking

Plasma, buffy coat and whole blood specimens for banking and future translational medicine studies will be submitted to the SWOG Specimen Repository – Solid Tissue, Myeloma and Lymphoma Division, Lab #201. Specimen banking is optional for the patient.

a. With patient's consent plasma and buffy coat and whole blood specimens must be submitted at the timepoints listed below in Section 15.2b. Collection instructions are outlined in Section 15.2c and submission instructions are outlined in Section 15.2e.

b. With patient's consent plasma and whole blood specimens must be submitted at the following times (see Section 9.0):

1. Plasma and Buffy Coat

Blood specimens (two purple top EDTA tubes for plasma and buffy coats) will be collected after registration, but prior to Cycle 1 treatment; at the beginning of Cycles 2 and 3; at the time of removal from protocol treatment (regardless of timepoint); and at the time of disease recurrence. These specimens must be shipped within 28 days after obtaining to Lab #201. These specimens will be banked until funding is obtained to perform translational medicine studies which will include assessing molecular biomarkers relevant to the AKT/mTOR and other pathways implicated in the pathogenesis of renal carcinoma and investigating their potential predictive and prognostic value.

2. Whole Blood

Five mL of venous blood will be collected in a purple-top EDTA tube immediately prior to Cycles 1-3 and at the time the patient is removed from protocol treatment. **Prior to Cycles 1-3, patients must be instructed not to take their morning everolimus dose until after the samples have been obtained**. These specimens must be shipped within 28 days after obtaining to Lab #201. These specimens will be banked until funding is obtained to perform translational medicine studies which will include determining the steady-state everolimus trough levels.

c. Plasma and Whole Blood Specimen Collection Instructions:

1. Plasma and Buffy Coat Specimen Collection and Processing Instructions:

a. Blood samples should be collected using standard venipuncture technique.

b. Cryovials must be labeled with the protocol number (**S0931**), SWOG patient number, patient's initials, and date and time of specimen collection.

c. Collect ~6 mL whole blood in each of two (2) purple top tubes (EDTA tubes).

d. Immediately invert tube (gently) 8-10 times. This reduces the possibility of clot formation.

e. Centrifuge sample at 800 x g for 10 minutes (~1500-2000 rpm, determined by centrifuge rotor size).

f. Immediately after centrifuging, transfer plasma in 1 mL aliquots to labeled cryovials.

g. Pipette slowly to avoid disturbing the buffy coat layer.

h. Leave a small amount of plasma (~.5 cm) above the buffy coat layer in each tube.

i. After removing plasma from both tubes, transfer buffy coat to labeled cryovials.
- The buffy layer is the off-white layer between the plasma and the red blood cells.
- Pipette slowly in a circular motion to obtain as many buffy coat cells as possible.
- Contamination of the buffy coat with red blood cells is expected and not a concern.

j. Immediately freeze vials in a freezing apparatus (ex. Mr. Frosty). If not available, slow freeze vials on dry ice or in a -70°C freezer. Store frozen plasma vials in a -70°C to -80°C freezer until ready to ship. If a -70°C to -80°C freezer is not available, a -20°C would be sufficient.

2. Whole Blood Specimen Collection Instructions:

a. Prior to each scheduled appointment, patients must be instructed not to take their morning everolimus dose until after the sample has been obtained.

b. Blood samples should be collected using standard venipuncture technique.

c. Cryovials must be labeled with the protocol number (**S0931**), SWOG patient number, patient's initials, and date and time of specimen collection.

d. Collect 5 mL of venous blood into a purple top EDTA tubes.

e. Immediately invert tube (gently) 8-10 times. This reduces the possibility of clot formation.

f. If you are using plastic vacutainer tubes, samples can be directly frozen at -20°C. If the blood collection tubes are glass, whole blood should be transferred to an appropriately labeled polypropylene freezer vial and frozen at <-20°C. **Note: Plasma should not be separated from whole blood. Whole blood should be frozen directly without separation.**

d. Specimen collection kits are not being provided for this submission; sites will use institutional supplies.

e. SHIPPING SAMPLES

1. SWOG Specimen Tracking System (STS)

All specimen submissions for this study must be entered and tracked using the SWOG online Specimen Tracking system. SWOG members may log on the online system via the CRA Workbench. To access the CRA Workbench, go to the SWOG Web site (http://swog.org) and logon to the Members Area. After you have logged on using your SWOG roster ID number and password, click on the *CRA Workbench* link to access the home page for CRA Workbench website. First time non- SWOG users must refer to start-up instructions located at https://gill.crab.org/SpecTrack/.

A copy of the Shipment Packing List produced by the online Specimen Tracking system should be printed and placed in the pocket of the specimen bag if it has one, or in a separate resealable bag. The Specimen Submission Form is NOT required when the online system is used.

ALL SPECIMENS MUST BE LOGGED VIA THIS SYSTEM; THERE ARE NO EXCEPTIONS.

To report technical problems with Specimen Tracking, such as database errors or connectivity issues, please send an email to technicalquestion@crab.org. For procedural help with logging and shipping specimens, there is an introduction to the system on the Specimen Tracking main page

(http://dnet.crab.org/SpecTrack/Documents/Instructions.pdf); or contact the Data Operations Center at 206/652-2267 to be routed to the Data Coordinator for further assistance.

In the online specimen tracking system, the appropriate SWOG laboratory for submission of plasma, whole blood and tissue specimens for SWOG Repository submission is identified as follows:

Lab #201:	SWOG Specimen Repository – Solid Tissue, Myeloma and Lymphoma Division
Contact:	Erin Grundy
Phone:	614/355-3099
Email:	bpcbank@nationwidechildrens.org

2. Plasma, buffy coats, and whole blood <u>must</u> be shipped on dry ice by overnight courier Monday through Wednesday.

3. Federal guidelines for the shipment of blood products:

   a. The tube must be wrapped in an absorbent material.

   b. The tube must then be placed in an AIRTIGHT container (like a resealable bag).

   c. Pack the resealable bag and tube in a Styrofoam shipping container.

d. Pack the Styrofoam shipping container in a cardboard box.

e. Mark the box "Biohazard".

16.0 ETHICAL AND REGULATORY CONSIDERATIONS

The following must be observed to comply with Food and Drug Administration regulations for the conduct and monitoring of clinical investigations; they also represent sound research practice:

Informed Consent

The principles of informed consent are described by Federal Regulatory Guidelines (Federal Register Vol. 46, No. 17, January 27, 1981, part 50) and the Office for Protection from Research Risks Reports: Protection of Human Subjects (Code of Federal Regulations 45 CFR 46). They must be followed to comply with FDA regulations for the conduct and monitoring of clinical investigations.

Institutional Review

This study must be approved by an appropriate institutional review committee as defined by Federal Regulatory Guidelines (Ref. Federal Register Vol. 46, No. 17, January 27, 1981, part 56) and the Office for Protection from Research Risks Reports: Protection of Human Subjects (Code of Federal Regulations 45 CFR 46).

Drug Accountability

An investigator is required to maintain adequate records of the disposition of investigational drugs according to procedures and requirements governing the use of investigational new drugs as described in the Code of Federal Regulations 21 CFR 312.

Monitoring

This study will be monitored by the Clinical Data Update System (CDUS) Version 3.0. Cumulative CDUS data will be submitted quarterly to CTEP by electronic means. Reports are due January 31, April 30, July 31 and October 31.

Confidentiality

Please note that the information contained in this protocol is considered confidential and should not be used or shared beyond the purposes of completing protocol requirements until or unless additional permission is obtained.

16.1 Adverse Event Reporting Requirements

a. Purpose

Adverse event data colle n and reporting, which are required as part of every clinical trial, are done to ensure the safety of patients enrolled in the studies as well as those who will enroll in future studies using similar agents. Adverse events are reported in a routine manner at scheduled times during a trial. (Directions for routine reporting are provided in Section 14.0.) Additionally, certain adverse events must be reported in an expedited manner to allow for more timely monitoring of patient safety and care. The following guidelines prescribe expedited adverse event reporting for this protocol.

b. Reporting method

This study requires that expedited adverse events be reported using the Cancer Therapy Evaluation Program Adverse Event Reporting System (CTEP-AERS). CTEP's guidelines for CTEP-AERS can be found at http://ctep.cancer.gov. A

CTEP-AERS report must be submitted to the SWOG Operations Office electronically via the CTEP-AERS Web-based application located at: http://ctep.cancer.gov/protocolDevelopment/electronic_applications/adverse_events.htm.

c. When to report an event in an expedited manner

Some adverse events require 24-hour notification (refer to Table 16.1) via CTEP-AERS. When Internet connectivity is disrupted, a 24-hour notification is to be made to the SWOG Operations Office by telephone at 210-614-8808 or by email at adr@swog.org. Once Internet connectivity is restored, a 24-hour notification that was made by phone or using adr@swog.org must be entered electronically into CTEP-AERS by the original submitter at the site.

When the adverse event requires expedited reporting, submit the report within the number of calendar days of learning of the event specified in Table 16.1.

d. Other recipients of adverse event reports

The Operations Office will forward reports and documentation to the appropriate regulatory agencies and drug companies as required.

Adverse events determined to be reportable the Institutional Review Board responsible for oversight of the patient must be reported according to local policy and procedures.

e. **Expedited reporting for investigational agents**

Expedited reporting is required if the patient has received at least one dose of the investigational agent as part of the trial. Reporting requirements are provided in Table 16.1. The investigational agent used in this study is everolimus. If there is any question about the reportability of an adverse event or if on-line CTEP-AERS cannot be used, please telephone or email the SAE Specialist at the SWOG Operations Office, 210-614-8808 or adr@swog.org, before preparing the report.

SWOG Authorized Document

Table 16.1:

Late Phase 2 and Phase 3 Studies: Expedited Reporting Requirements for Adverse Events that Occur on studies under a Non-CTEP IND within 30 Days[1] of the Last Administration of the Investigational Agent/Intervention[1] (Everolimus).

FDA REPORTING REQUIREMENTS FOR SERIOUS ADVERSE EVENTS (21 CFR Part 312)
NOTE: Investigators **MUST** immediately report to the sponsor (NCI) **ANY** Serious Adverse Events, whether or not they are considered related to the investigational agent(s)/intervention (21 CFR 312.64)

An adverse event is considered serious if it results in **ANY** of the following outcomes:

1) Death
2) A life-threatening adverse event
3) An adverse event that results in inpatient hospitalization or prolongation of existing hospitalization for ≥ 24 hours
4) A persistent or significant incapacity or substantial disruption of the ability to conduct normal life functions
5) A congenital anomaly/birth defect.
6) Important Medical Events (IME) that may not result in death, be life threatening, or require hospitalization may be considered serious when, based upon medical judgment, they may jeopardize the patient or subject and may require medical or surgical intervention to prevent one of the outcomes listed in this definition. (FDA, 21 CFR 312.32; ICH E2A and ICH E6).

ALL SERIOUS adverse events that meet the above criteria **MUST** be immediately reported to the NCI via CTEP-AERS within the timeframes detailed in the table below.

<table>
<tr><th>Hospitalization</th><th>Grade 1 Timeframes</th><th>Grade 2 Timeframes</th><th>Grade 3 Timeframes</th><th>Grade 4 & 5 Timeframes</th></tr>
<tr><td>Resulting in Hospitalization ≥ 24 hrs</td><td colspan="3">10 Calendar Days</td><td rowspan="2">24-Hour 5 Calendar Days</td></tr>
<tr><td>Not resulting in Hospitalization ≥ 24 hrs</td><td colspan="2">Not required</td><td>10 Calendar Days</td></tr>
</table>

NOTE: Protocol specific exceptions to expedited reporting of serious adverse events are found in the Specific Protocol Exceptions to Expedited Reporting (SPEER) portion of the CAEPR or [Section 16.1f.]

Expedited AE reporting timelines are defined as:

- o "24-Hour; 5 Calendar Days" - The AE must initially be reported via CTEP-AERS within 24 hours of learning of the AE, followed by a complete expedited report within 5 calendar days of the initial 24-hour report.
- o "10 Calendar Days" - A complete expedited report on the AE must be submitted within 10 calendar days of learning of the AE.

[1]Serious adverse events that occur more than 30 days after the last administration of investigational agent/intervention and have an attribution of possible, probable, or definite require reporting as follows:

Expedited 24-hour notification followed by complete report within 5 calendar days for:

- All Grade 4, and Grade 5 AEs

Expedited 10 calendar day reports for:

- Grade 2 adverse events resulting in hospitalization or prolongation of hospitalization
- Grade 3 adverse events

May 5, 2011

f. **Additional Instructions or Exceptions to CTEP-AERS Expedited Reporting Requirements for Late Phase 2 and Phase 3 Trials Utilizing an Agent under a Non-CTEP IND:**

1) **Group-specific instructions.**

Supporting Documentation Submission - Within **5 calendar days** submit the following to the SWOG Operations Office by fax to 210-614-0006 or mail to the address below:

- Printed copy of the first page of the CTEP-AERS report
- Copies of clinical source documentation of the event
- If applicable, and they have not yet been submitted to the SWOG Data Operations Center, copies of Off Treatment Notice and/or Notice of Death.

2) The adverse event listed below does not require expedited reporting via CTEP-AERS:

- Grade 4 myelosuppression

g. **Reporting of Secondary Malignancy Including AML/ALL/MDS**

1. A secondary malignancy is a cancer caused by treatment for a previous malignancy (e.g., treatment with investigational agent/intervention, radiation or chemotherapy). A secondary malignancy is not considered a metastasis of the initial neoplasm.

SWOG requires all secondary malignancies that occur following treatment with an agent under a non-NCI IND to be reported via CTEP-AERS. Three options are available to describe the event.

- Leukemia secondary to oncology chemotherapy (e.g., Acute Myelocytic Leukemia [AML])
- Myelodysplastic syndrome (MDS)
- Treatment-related secondary malignancy

Any malignancy possibly related to cancer treatment (including AML/MDS) should also be reported via the routine reporting mechanisms outlined in each protocol.

Second Malignancy: A second malignancy is one unrelated to the treatment of a prior malignancy (and is NOT a metastasis from the initial malignancy). Second malignancies require ONLY routine reporting via CDUS unless otherwise specified.

For more information see:
http://ctep.cancer.gov/protocolDevelopment/electronic_applications/docs/aeguidelines.pdf.

2. Supporting documentation should be submitted to CTEP in accordance with instructions provided by the CTEP-AERS system. A copy of the report and the following supporting documentation must also be submitted to SWOG Operations Office within 30 days by fax to 210/614-0006 or mail to the address below:

- a copy of the pathology report confirming the AML/ALL /MDS diagnosis
- (if available) a copy of the cytogenetics report

SWOG
ATTN: SAE Program
4201 Medical Drive, Suite 250
San Antonio, Texas 78229

NOTE: If a patient has been enrolled in more than one NCI-sponsored study, the report must be submitted for the most recent trial.

h. Reporting Pregnancy, Fetal Death, and Death Neonatal

1. **Pregnancy** Study participants who become pregnant while on study; that pregnancy should be reported in an expedited manner via CTEP-AERS as **Grade 3 "Pregnancy, puerperium and perinatal conditions – Other (pregnancy)"** under the **Pregnancy, puerperium and perinatal conditions** SOC.

   Additionally, the pregnancy outcome for patients on study should be reported via CTEP-AERS at the time the outcome becomes known, accompanied by the same Pregnancy Report Form used for the initial report.

2. **Fetal Death** Fetal Death defined in CTCAE as "A disorder characterized by death in utero; failure of the product of conception to show evidence of respiration, heartbeat, or definite movement of a voluntary muscle after expulsion from the uterus, without possibility of resuscitation" should be reported expeditiously as **Grade 4 "pregnancy, puerperium and perinatal conditions – Other (pregnancy loss)" under the Pregnancy, puerperium and perinatal conditions** SOC.

3. **Death Neonatal** Neonatal death, defined in CTCAE as "A disorder characterized by cessation of life occurring during the first 28 days of life" that is felt by the investigator to be at least possibly due to the investigational agent/intervention should be reported expeditiously.

   A neonatal death should be reported expeditiously as **Grade 4 "General disorders and administration – Other (neonatal loss)"** under the **General disorders and administration** SOC.

*Fetal death and neonatal death should **NOT** be reported as a Grade 5 event. If reported as such, the CTEP-AERS interprets this as a death of the patient being treated.*

NOTE: When submitting CTEP-AERS reports for "Pregnancy, "Pregnancy loss", or "Neonatal loss", the Pregnancy Information Form should also be completed and faxed with any additional medical information to 301/230-0159. The potential risk of exposure of the fetus to the investigational agent(s) or chemotherapy agent(s) should be documented in the "Description of Event" section of the CTEP-AERS report.

The Pregnancy Information Form is available at:
http://ctep.cancer.gov/protocolDevelopment/adverse_effects.htm

SWOG Authorized Document

17.0 BIBLIOGRAPHY

1 Jemal A, Siegel R, Ward E, et al. Cancer statistics. CA Cancer J Clin 59:225-249, 2009.

2 Janzen NK, Kim HL, Figlin RA, Belldegrun AS. Surveillance after radical or partial nephrectomy for localized renal cell carcinoma and management of recurrent disease. Urol Clin North Am 30:843-52, 2003.

3 Lam JS, Shvarts O, Leppert JT, Figlin RA, Belldegrun AS. Renal cell carcinoma 2005: new frontiers in staging, prognostication and targeted molecular therapy. J Urol 173:1853-62, 2005.

4 Atzpodien J, Schmitt E, Gertenbach U, et al. Adjuvant treatment with interleukin-2- and interferon-alpha2a-based chemoimmunotherapy in renal cell carcinoma post tumour nephrectomy: results of a prospectively randomized trial of the German Cooperative Renal Carcinoma Chemoimmunotherapy Group (DGCIN). Br J Cancer 92:843-6, 2005.

5 Clark JI, Atkins MB, Urba WJ, et al. Adjuvant high-dose bolus interleukin-2 for patients with high-risk renal cell carcinoma: a cytokine working group randomized trial. J Clin Oncol 21:3133-40, 2003.

6 Pizzocaro G, Piva L, Colavita M, et al. Interferon adjuvant to radical nephrectomy in Robson stages II and III renal cell carcinoma: a multicentric randomized study. J Clin Oncol 19:425-31, 2001.

7 Messing EM, Manola J, Wilding G, et al. Phase III study of interferon alfa-NL as adjuvant treatment for resectable renal cell carcinoma: an Eastern Cooperative Oncology Group/Intergroup trial. J Clin Oncol 21:1214-22, 2003.

8 Jocham D, Richter A, Hoffmann L, et al. Adjuvant autologous renal tumour cell vaccine and risk of tumour progression in patients with renal-cell carcinoma after radical nephrectomy: phase III, randomized controlled trial. Lancet 363:594-9, 2004.

9 Wood C, Srivastava P, Bukowski R, et al. An adjuvant autologous therapeutic vaccine (HSPPC-96; vitespen) versus observation alone for patients at high risk of recurrence after nephrectomy for renal cell carcinoma: a multicentre, open-label, randomized phase III trial. Lancet 372:145-54, 2008.

10 Motzer RJ, Hutson TE, Tomczak P, et al. Sunitinib versus interferon alfa in metastatic renal-cell carcinoma. N Engl J Med 356:115-24, 2007.

11 Escudier B, Eisen T, Stadler WM, et al. Sorafenib in advanced clear-cell renal-cell carcinoma. N Engl J Med 356:125-34, 2007.

12 Hudes G, Carducci M, Tomczak P, et al. Temsirolimus, interferon alfa, or both for advanced renal-cell carcinoma. N Engl J Med 356:2271-81, 2007.

13 Escudier B, Pluzanska A, Koralewski P, et al. Bevacizumab plus interferon alfa-2a for treatment of metastatic renal cell carcinoma: a randomized, double-blind phase III trial. Lancet 370:2103-11, 2007.

14 Rini BI, Halabi S, Rosenberg JE, et al. Bevacizumab plus interferon alfa compared with interferon alfa monotherapy in patients with metastatic renal cell carcinoma: CALGB 90206. J Clin Oncol 26:5422-8, 2008.

15 Motzer RJ, Escudier B, Oudard S, et al. Efficacy of everolimus in advanced renal cell carcinoma: a double-blind, randomized, placebo-controlled phase III trial. Lancet 372:449-56, 2008.

SWOG Authorized Document

16 Lam JS, Shvarts O, Leppert JT, Pantuck AJ, Figlin RA, Belldegrun AS. Postoperative surveillance protocol for patients with localized and locally advanced renal cell carcinoma based on a validated prognostic nomogram and risk group stratification system. J Urol 174:466-72; discussion 72; quiz 801, 2005.

17 Hudson CC, Liu M, Chiang GG, et al. Regulation of hypoxia-inducible factor 1alpha expression and function by the mammalian target of rapamycin. Mol Cell Biol 22:7004-14, 2002.

18 Hudes G, Carducci M, Tomczak P, et al. Temsirolimus, interferon alfa, or both for advanced renal-cell carcinoma. N Engl J Med 356:2271-81, 2007.

19 Motzer RJ, Bacik J, Murphy BA, Russo P, Mazumdar M. Interferon-alfa as a comparative treatment for clinical trials of new therapies against advanced renal cell carcinoma. J Clin Oncol 20:289-96, 2002.

20 Motzer RJ, Escudier B, Oudard S, et al. Efficacy of everolimus in advanced renal cell carcinoma: a double-blind, randomized, placebo-controlled phase III trial. Lancet 372:449-56, 2008.

21 Everolimus Investigators Brochure, Edition 12, Novartis Pharmaceuticals, 2013.

22 O'Donnell A, Faivre S, Burris HA, 3rd, et al. Phase I pharmacokinetic and pharmacodynamic study of the oral mammalian target of rapamycin inhibitor everolimus in patients with advanced solid tumors. J Clin Oncol 26:1588-95, 2008.

23 Tabernero J, Rojo F, Calvo E, et al. Dose- and schedule-dependent inhibition of the mammalian target of rapamycin pathway with everolimus: a phase I tumor pharmacodynamic study in patients with advanced solid tumors. J Clin Oncol 26:1603-10, 2008.

24 Motzer RJ, Escudier B, Oudard S, et al. Efficacy of everolimus in advanced renal cell carcinoma: a double-blind, randomized, placebo-controlled phase III trial. Lancet 372:449-56, 2008.

25 Motzer RJ, et al. Phase III trial of everolimus for metastatic renal cell carcinoma: final results and analysis of prognostic factors. Cancer 116(18):4256-65, 2010.

26 Motzer RJ, Escudier B, Oudard S, et al. Efficacy of everolimus in advanced renal cell carcinoma: a double-blind, randomized, placebo-controlled phase III trial. Lancet 372:449-56, 2008.

27 Motzer RJ, Escudier B, Oudard S, et al. Efficacy of everolimus in advanced renal cell carcinoma: a double-blind, randomized, placebo-controlled phase III trial. Lancet 372:449-56, 2008.

28 O'Donnell A, Faivre S, Burris HA, 3rd, et al. Phase I pharmacokinetic and pharmacodynamic study of the oral mammalian target of rapamycin inhibitor everolimus in patients with advanced solid tumors. J Clin Oncol 26:1588-95, 2008.

29 Tabernero J, Rojo F, Calvo E, et al. Dose- and schedule-dependent inhibition of the mammalian target of rapamycin pathway with everolimus: a phase I tumor pharmacodynamic study in patients with advanced solid tumors. J Clin Oncol 26:1603-10, 2008.

30 http://clinicaltrials.gov/ct/show/NCT00326898.

31 Zisman A, Pantuck AJ, Dorey F, Said JW, Shvarts O, Quintana D, Gitlitz BJ, deKernion JB, Figlin RA, Belldegrun AS. Improved prognostication of renal cell carcinoma using an integrated staging system. J Clin Oncol Mar 15;19(6):1649-57, 2001.

32 Lam JS, Shvarts O, Leppert JT, Pantuck AJ, Figlin RA, Belldegrun AS. Postoperative surveillance protocol for patients with localized and locally advanced renal cell carcinoma based on a validated prognostic nomogram and risk group stratification system. J Urol 174:466-72; discussion 72; quiz 801, 2005.

SWOG Authorized Document

33 Zisman A, Pantuck AJ, Dorey F, Said JW, Shvarts O, Quintana D, Gitlitz BJ, deKernion JB, Figlin RA, Belldegrun AS. Improved prognostication of renal cell carcinoma using an integrated staging system. J Clin Oncol Mar 15;19(6):1649-57, 2001.

34 Hudes G, Carducci M, Tomczak P, et al. Temsirolimus, interferon alfa, or both for advanced renal-cell carcinoma. N Engl J Med 356:2271-81, 2007.

35 Dutcher JP et al. 26(2):202-9. Epub 2009 Feb 20.

36 Kovarik JM, Kahan BD, Kaplan B, et al. Longitudinal assessment of everolimus in de novo renal transplant recipients over the first post-transplant year: pharmacokinetics, exposure-response relationships, and influence on cyclosporine. Clin Pharmacol Ther 69:48-56, 2001.

37 Vitko S, Margreiter R, Weimar W, et al. Everolimus (Certican) 12-month safety and efficacy versus mycophenolate mofetil in de novo renal transplant recipients. Transplantation 78:1532-40, 2004.

38 Kovarik JM, Kaplan B, Tedesco Silva H, et al. Exposure-response relationships for everolimus in de novo kidney transplantation: defining a therapeutic range. Transplantation 73:920-5, 2002.

39 Kovarik JM, Kahan BD, Kaplan B, et al. Longitudinal assessment of everolimus in de novo renal transplant recipients over the first post-transplant year: pharmacokinetics, exposure-response relationships, and influence on cyclosporine. Clin Pharmacol Ther 69:48-56, 2001.

40 Mabasa VH, Ensom MH. The role of therapeutic monitoring of everolimus in solid organ transplantation. Ther Drug Monit 27:666-76, 2005.

41 Kovarik JM, Kaplan B, Tedesco Silva H, et al. Exposure-response relationships for everolimus in de novo kidney transplantation: defining a therapeutic range. Transplantation 73:920-5, 2002.

42 Kovarik JM, Tedesco H, Pascual J, et al. Everolimus therapeutic concentration range defined from a prospective trial with reduced-exposure cyclosporine in de novo kidney transplantation. Ther Drug Monit 26:499-505, 2004.

43 O'Donnell A, Faivre S, Burris HA, 3rd, et al. Phase I pharmacokinetic and pharmacodynamic study of the oral mammalian target of rapamycin inhibitor everolimus in patients with advanced solid tumors. J Clin Oncol 26:1588-95, 2008.

44 Tabernero J, Rojo F, Calvo E, et al. Dose- and schedule-dependent inhibition of the mammalian target of rapamycin pathway with everolimus: a phase I tumor pharmacodynamic study in patients with advanced solid tumors. J Clin Oncol 26:1603-10, 2008.

45 Pocock SJ, Simon R. Sequential treatment assignment with balancing for prognostic factors in controlled clinical trials. Biometrics 31:103-115, 1975.

SWOG Authorized Document

18.0 APPENDIX

18.1 New York Heart Association Classifications

18.2 Emergency Unblinding Guidelines

18.3 Examples of Clinically Relevant Drug Interactions: Substrates, Inducers and Inhibitors of the Isoenzyme CYP3A4

18.4 Intake Calendar

18.1 New York Heart Association Classifications

TABLE I, NEW YORK HEART ASSOCIATION CLASS

Class	Cardiac Symptoms	Limitations	Need for Additional Rest*	Physical Ability To Work**
I	None	None	None	Full Time
II	Only moderate	Slight	Usually only slight or occasional	Usually full time
III	Defined, with less than ordinary activity	Marked	Usually moderate	Usually part time
IV	May be present even at rest, & any activity increases discomfort	Extreme	Marked	Unable to work

* To control or relieve symptoms, as determined by the patient, rather than as advised by the physician.

** At accustomed occupation or usual tasks.

18.2 Emergency Unblinding Guidelines

a. **General Considerations**

The randomized regimen for this study includes a blinded drug, which is either everolimus or placebo. During the course of this study it may become necessary to identify (or unblind) a patient's treatment assignment. The circumstances that will warrant emergency unblinding and the procedure for emergency unblinding are described in this Appendix.

PLEASE NOTE: Unblinding requests for disease recurrence MUST be handled by the planned unblinding instructions outlined in Section 7.5. Do not contact the Washington Poison Center with these requests.

b. **Criteria for Emergency Unblinding**

In general, treatment assignments will not be emergency unblinded unless there is a compelling medical or ethical reason that the treatment should be identified. In most circumstances it will be appropriate to treat the patient or person who received blinded drug as though he or she received everolimus, irrespective of the drug actually received. Therefore, emergency unblinding should seldom be necessary.

The following events MAY require emergency unblinding of treatment assignments in this study:

1. A compelling medical need as determined by a physician, e.g., existence of a condition for which knowledge of the patient's treatment assignment is necessary for the selection of appropriate care.

2. Administration of blinded drug to a person other than the patient.

c. **Procedure for Emergency Unblinding**

Emergency unblinding of treatment assignments for patients on this study will be performed by the Washington Poison Center (WPC), upon approval from a designated physician (either one of the WPC's resource physicians or Dr. Christopher Ryan). The procedure for emergency unblinding the treatment assignment for a patient on this study is as follows:

1. All requests for emergency unblinding must be made by the registering physician or his/her designee.

2. Call the WPC collect at 206/526-2121 from outside Washington State or toll free at 800/222-1222 from within Washington State. The WPC is accessible 24 hours per day, 365 days per year.

3. The person calling the WPC must be prepared to provide the following information:

   Study number (**S0931**)

   SWOG Patient Number (e.g., "999999")

   Patient Initials

   Name and telephone number of the caller

   Reason emergency unblinding is thought to be required

4. The WPC will contact one of its resource physicians and provide the information received from the caller. If none of the WPC's resource physicians can be contacted, then the WPC will contact Dr. Christopher Ryan. The contacted physician will evaluate the need for emergency unblinding and provide the WPC either approval to unblind or a recommendation for treatment, if any, while maintaining blinding. The WPC will then call the person who initiated the unblinding request and tell him/her either the treatment assignment or the resource physician's treatment recommendation.

5. If the WPC is unable to contact any of its resource physicians or Dr. Ryan within three hours after receiving the request for emergency unblinding, then the WPC will notify the person who initiated the unblinding request that treatment assignment will not be unblinded at that time and treatment of the patient or person who received blinded drug should proceed as if the blinded drug is everolimus. In such cases, the WPC will continue to attempt to contact the resource physicians, and when one of them is contacted, will proceed as in #4 above.

6. Any patient whose treatment assignment is emergency unblinded will receive no further blinded drug, but should continue all other protocol treatment if his/her medical condition permits.

7. Unblinding of treatment assignments for any reason must be documented on the Off Treatment Notice.

Questions regarding the unblinding may be directed to any of the following resource physicians:

Christopher W. Ryan, M.D.
Oregon Health & Science University
3303 SW Bond Avenue, CH14R
Portland, OR 97239
Phone: 503/494-8487

Bruce G. Redman, D.O.
SWOG
24 Frank Lloyd Wright Drive
P.O. Box 483
Ann Arbor, MI 48106
Phone: 734/998-7154

Washington Poison Center
Phone: 206/526-2121

18.3 Examples of Clinically Relevant Drug Interactions: Substrates, Inducers and Inhibitors of the Isoenzyme CYP3A4

Only the strong CYP3A4 inducers and inhibitors listed in Section 5.2e are prohibited during study treatment. Refer to Sections 7.1c and 7.3 for guidance regarding the use of moderate CYP3A4 inducers and inhibitors while on treatment.

CYP3A4 Substrates

Albuterol	Dihydroergotamine	Isradipine	Quinidine
Alfentanil	Diltiazem	Itraconazole	Rabeprazole
Alprazolam	Disopyramide	Ketamine	Ranolazine
Amiodarone	Docetaxel	Ketoconazole	Repaglinide
Amlodipine	Doxepin	Lansoprazole	Rifabutin
Amprenavir	Doxorubicin	Letrozole	Ritonavir
Aprepitant	Doxycycline	Levonorgestrel	Salmeterol
Aripiprazole	Efavirenz	Lidocaine	Saquinavir
Atazanavir	Eletriptan	Losartan	Sibutramine
Atorvastatin	Enalapril	Lovastatin	Sildenafil
Benzphetamine	Eplerenone	Medroxyprogesterone	Simvastatin
Bisoprolol	Ergoloid mesylates	Mefloquine	Sirolimus
Bortezomib	Ergonovine	Mestranol	Spiramycin
Bosentan	Ergotamine	Methadone	Sufentanil
Bromazepam	Erythromycin	Methylergonovine	Sunitinib
Bromocriptine	Escitalopram	Methysergide	Tacrolimus
Budesonide	Estradiol	Miconazole	Tamoxifen
Buprenorphine	Estrogens, conj., synthetic	Midazolam	Tamsulosin
Buspirone	Estrogens, conj., equine	Miglustat	Telithromycin
Busulfan	Estrogens, conj., esterified	Mirtazapine	Teniposide
Carbamazepine	Estrone	Modafinil	Tetracycline
Cerivastatin	Estropipate	Montelukast	Theophylline
Chlordiazepoxide	Ethinyl estradiol	Moricizine	Tiagabine
Chloroquine	Ethosuximide	Nateglinide	Ticlopidine
Chlorpheniramine	Etoposide	Nefazodone	Tipranavir
Cilostazol	Exemestane	Nelfinavir	Tolterodine
Cisapride	Felbamate	Nevirapine	Toremifene
Citalopram	Felodipine	Nicardipine	Trazodone
Clarithromycin	Fentanyl	Nifedipine	Triazolam
Clobazam	Flurazepam	Nimodipine	Trimethoprim
Clonazepam	Flutamide	Nisoldipine	Trimipramine
Clorazepate	Fluticasone	Norethindrone	Troleandomycin
Cocaine	Fosamprenavir	Norgestrel	Vardenafil
Colchicine	Gefitinib	Ondansetron	Venlafaxine
Conivaptan	Haloperidol	Paclitaxel	Verapamil
Cyclophosphamide	Ifosfamide	Pergolide	Vinblastine
Cyclosporine	Imatinib	Phencyclidine	Vincristine
Dantrolene	Indinavir	Pimozide	Vinorelbine
Dapsone	Irinotecan	Pipotiazine	Zolpidem
Dasatinib (1)	Isosorbide	Primaquine	Zonisamide
Delavirdine	Isosorbide dinitrate	Progesterone	Zopiclone
Diazepam	Isosorbide mononitrate	Quetiapine	

CYP3A4 Inhibitors

Acetominophen	Diclofenac	Lomustine	Primaquine
Acetazolamide	Dihydroergotamine	Losartan	Progesterone
Amiodarone	Diltiazem	Lovastatin	Propofol
Amlodipine	Disulfiram	Mefloquine	Propoxyphene
Amprenavir	Docetaxel	Mestranol	Quinidine
Anastrozole	Doxorubicin	Methadone	Quinine
Aprepitant	Doxycycline	Methimazole	Quinupristin
Atazanavir	Drospirenone	Methoxsalen	Rabeprazole
Atorvastatin	Efavirenz	Methylprednisolone	Ranolazine
Azelastine	Enoxacin	Metronidazole	Risperidone
Azithromycin	Entacapone	Miconazole	Ritonavir
Betamethasone	Ergotamine	Midazolam	Saquinavir
Bortezomib	Erythromycin	Mifepristone	Selegiline
Bromocriptine	Ethinyl estradiol	Mirtazapine	Sertraline
Caffeine	Etoposide	Mitoxantrone	Sildenafil
Cerivastatin	Felodipine	Modafinil	Sirolimus
Chloramphenicol	Fentanyl	Nefazodone	Sulconazole
Chlorzoxazone	Fluconazole	Nelfinavir	Tacrolimus
Cimetidine	Fluoxetine	Nevirapine	Tamoxifen
Ciprofloxacin	Fluvastatin	Nicardipine	Telithromycin
Cisapride	Fluvoxamine	Nifedipine	Teniposide
Clarithromycin	Fosamprenavir	Nisoldipine	Testosterone
Clemastine	Glyburide	Nizatidine	Tetracycline
Clofazimine	Grapefruit juice (2)	Norfloxacin	Ticlopidine
Clotrimazole	Haloperidol	Olanzapine	Tranylcypromine
Clozapine	Hydralazine	Omeprazole	Trazodone
Cocaine	Ifosfamide	Orphenadrine	Troleandomycin
Conivaptan	Imatinib	Oxybutynin	Valproic acid
Cyclophosphamide	Indinavir	Paroxetine	Venlafaxine
Cyclosporine	Irbesartan	Pentamidine	Verapamil
Danazol	Isoniazid	Pergolide	Vinblastine
Dasatinib (1)	Isradipine	Phencyclidine	Vincristine
Delavirdine	Itraconazole	Pilocarpine	Vinorelbine
Desipramine	Ketoconazole	Pimozide	Voriconazole
Dexmedetomidine	Lansoprazole	Pravastatin	Zafirlukast
Diazepam	Lidocaine	Prednisolone	Ziprasidone

CYP3A4 Inducers

Aminoglutethimide	Nevirapine	Phenytoin	Rifapentine
Carbamazepine	Oxcarbazepine	Primidone	St. John's wort (3)
Fosphenytoin	Pentobarbital	Rifabutin	
Nafcillin	Phenobarbital	Rifampin	

When drugs classified as 'substrates' are co-administered with everolimus, there is the potential for higher concentrations of the 'substrate'. When everolimus is co-administered with compounds classified as 'inhibitors', increased plasma concentrations of everolimus is the potential outcome. The co-administration of 'inducers' would potentially lower plasma everolimus concentrations.

Note: Adapted from Cytochrome P450 Enzymes: Substrates, Inhibitors, and Inducers. In: Lacy CF, Armstrong LL, Goldman MP, Lance LL eds. Drug Information Handbook 15TH ed. Hudson, OH; LexiComp Inc. 2007: 1899-1912.

Only major substrates and effective inducers are listed. Additional information for drug interactions with cytochrome P450 isoenzymes can be found at http://medicine.iupui.edu/flockhart/.

PgP Inhibitors

PgP Substrates	PgP Inhibitors in vivo	PgP Inducers
digoxin, fexofenadine, indinavir, vincristine, colchicine, topotecan, paclitaxel	amiodarone, azithromycin, captopril, carvedilol, clarithromycin, conivaptan, cyclosporine, diltiazem, elacridar, erythromycin, felodipine, (GF120918), itraconazole, ketocoanzole, lopinavir, (LY335979), mibefradil, nifedipine, nitrendipine, (PSC833), quinidine, ranolazine, ritonavir, talinolol, valspodar, verapamil	rifampin, St John's wort

SWOG Authorized Document

18.4 Intake Calendar

SWOG Patient ID________ Patient Initials (L, F, M) ________ **SWOG Study #**__________

Institution/Affiliate ________________________ Physician ______________________________

Instructions for the participant:
This is a monthly calendar on which you are to record the number of tablets/pills/capsules you take each day. Be sure you have enough calendars to last until your next appointment. If you develop any side effects from the tablets/pills/capsules, mark this on the calendar on the day you note the effect. Bring your calendars with you each time you have an appointment.

If you have questions contact: _____________________ Telephone: ______________________

Your next appointment is: ________________________

Special instructions:

Month: **Year:**

Sunday	Monday	Tuesday	Wednesday	Thursday	Friday	Saturday

Patient Signature:________________________

Informed Consent Model for S0931

***NOTES FOR LOCAL INSTITUTION INFORMED CONSENT AUTHORS:**

This model informed consent form has been reviewed by the DCTD/NCI and is the official consent document for this study. Local IRB changes to this document are allowed. (Institutions should attempt to use sections of this document that are in bold type in their entirety.) Editorial changes to these sections may be made as long as they do not change information or intent. If the institutional IRB insists on making additions, deletions or more substantive modifications to the risks or alternatives sections, they may be justified in writing by the investigator and approved by the IRB. Under these circumstances, the revised language, justification and a copy of the IRB minutes must be forwarded to the SWOG Operations Office for approval before a patient may be registered to this study.

Please particularly note that the questions related to banking of specimens for future study are in bolded type and may not be changed in any way without prior approval from the SWOG Operations Office.

Readability Statistics:	
Flesch Reading Ease	64.7 (targeted above 55)
Flesch-Kincaid Grade Level	8.0 (targeted below 8.5)

- Instructions and examples for informed consent authors are in *[italics]*.
- A blank line, __________, indicates that the local investigator should provide the appropriate information before the document is reviewed with the prospective research participant.
- The term "study doctor" has been used throughout the model because the local investigator for a cancer treatment trial is a physician. If this model is used for a trial in which the local investigator is not a physician, another appropriate term should be used instead of "study doctor".
- The dates of protocol updates in the header and in the text of the consent is for reference to this model only and should not be included in the informed consent form given to the prospective research participant.
- The local informed consent must state which parties may inspect the research records. This includes the NCI, the drug manufacturer for investigational studies, any companies or grantors that are providing study support (these will be listed in the protocol's model informed consent form) and SWOG.

  SWOG must be listed as one of the parties that may inspect the research records in all protocol consent forms for which patient registration is being credited to SWOG. This includes consent forms for studies where all patients are registered directly through the SWOG Data Operations Office, all intergroup studies for which the registration is being credited to SWOG (whether the registration is through the SWOG Data Operations Office or directly through the other group), as well as consent forms for studies where patients are registered via CTSU and the registration is credited to SWOG.

- When changes to the protocol require revision of the informed consent document, the IRB should have a system that identifies the revised consent document, in order to preclude continued use of the older version and to identify file copies. An appropriate method to identify the current version of the consent is for the IRB to stamp the final copy of the consent document with the approval date. The stamped consent document is then photocopied for use. Other systems of identifying the current version of the consent such as adding a version or approval date are allowed as long as it is possible to determine during an audit that the patient signed the most current version of the consent form.

*NOTES FOR LOCAL INVESTIGATORS:

- The goal of the informed consent process is to provide people with sufficient information for making informed choices. The informed consent form provides a summary of the clinical study and the individual's rights as a research participant. It serves as a starting point for the necessary exchange of information between the investigator and potential research participant. This model for the informed consent form is only one part of the larger process of informed consent. For more information about informed consent, review the "Recommendations for the Development of Informed Consent Documents for Cancer Clinical Trials" prepared by the Comprehensive Working Group on Informed Consent in Cancer Clinical Trials for the National Cancer Institute. The Web site address for this document is http://cancer.gov/clinicaltrials/understanding/simplification-of-informed-consent-docs/
- A blank line, __________, indicates that the local investigator should provide the appropriate information before the document is reviewed with the prospective research participant.
- Suggestion for Local Investigators: An NCI pamphlet explaining clinical trials is available for your patients. The pamphlet is titled: "Taking Part in Cancer Treatment Research Studies". This pamphlet may be ordered on the NCI Web site at *https://cissecure.nci.nih.gov/ncipubs* or call 1-800-4- CANCER (1-800-422-6237) to request a free copy.
- Optional feature for Local Investigators: Reference and attach drug sheets, pharmaceutical information for the public, or other material on risks. Check with your local IRB regarding review of additional materials.

*These notes for authors and investigators are instructional and should not be included in the informed consent form given to the prospective research participant.

SWOG Authorized Document

S0931, "EVEREST: EVErolimus for Renal Cancer Ensuing Surgical Therapy, a Phase III Study"

This is a clinical trial, a type of research study. Your study doctor will explain the clinical trial to you. Clinical trials include only people who choose to take part. Please take your time to make your decision about taking part. You may discuss your decision with your friends and family. You can also discuss it with your health care team. If you have any questions, you can ask your study doctor for more explanation.

You are being asked to take part in this study because you have cancer of the kidney that has been surgically removed.

Why is this study being done?

The purpose of this study is to see whether treatment with everolimus after surgery for kidney cancer will increase the time without cancer returning. The current standard treatment after surgery is careful monitoring with no immediate treatment. Studies suggest that one way kidney cancer may grow is through chemical signaling through a protein named "mTOR". Everolimus is a drug that stops signaling through mTOR and may therefore stop the growth of kidney cancer. Everolimus is a drug currently approved for the treatment of patients with advanced or metastatic kidney cancer. It is considered investigational for use after surgery. In this study, you will get either everolimus or placebo (a pill with no medication). You will not get both.

How many people will take part in the study?

About 1,537 people will take part in this study.

What will happen if I take part in this research study?

Before you begin the study …

You will need to have the following exams, tests or procedures to find out if you can be in the study. These exams, tests or procedures are part of regular cancer care and may be done even if you do not join the study. If you have had some of them recently, they may not need to be repeated. This will be up to your study doctor.

- Medical history including screening for hepatitis B and C risk and physical examination,
- Blood tests for blood counts and to test your kidney and liver function,
- Blood tests to check your blood sugar (glucose) and lipids (cholesterol and triglycerides),
- CT scan to assess your disease.

During the study … *(section updated 5/6/14)*
If the exams, tests and procedures show that you can be in the study, and you choose to take part, then you will need the following tests and procedures. They are part of regular cancer care.

SWOG Authorized Document

- History and physical exam at the beginning of Week 1 and Week 4. (NOTE: If you receive a prestudy history and physical exam within 28 days prior to starting on this study, then these tests do not need to be repeated during Week 1.)
- Physical exam at the beginning of each 6 week cycle (Week 7, 13, 19, etc.) for a maximum of 9 cycles, *(2/11/16)*
- Blood tests for blood counts and to test your kidney and liver function at the beginning of each 6 week cycle (Week 7, 13, 19, etc.) for a maximum of 9 cycles, *(2/11/16)*
- Blood tests to check your blood sugar (glucose) and lipids (cholesterol and triglycerides) at the beginning of each 6 week cycle (Week 7, 13, 19, etc.) for a maximum of 9 cycles, *(2/11/16)*
- CT scan to assess your disease every eighteen weeks.

You will be "randomized" into one of the study groups described below. Randomization means that you are put into a group by chance. It is like flipping a coin except that a computer program will place you in one of the study groups. Neither you nor your doctor can choose the group you will be in. You will have an equal chance of being placed in either group. Once you have been placed into one of the groups, neither you nor your doctor will know which group you are in. The treatment in each group will be identical except that half of the patients will receive the investigational drug everolimus and half will take an identical placebo pill. Placebo pills do not contain any medication. These pills will look just like the pills containing everolimus. *(5/6/14)*

You will take two pills once a day by mouth with a glass of water and no more than a light fat-free meal. *(7/11/12)* Tablets must be swallowed whole and not chewed or crushed. Due to interaction with everolimus, you must not consume grapefruit or grapefruit juice while on study.

You will record the number of pills you take each day and any side effects you experience on a calendar. For the first 6 weeks, your doctor’s office will call you to see how you are doing on the weeks that you don’t have visits scheduled. You should bring your calendar with you each time you have a doctor’s visit. During your visits, your pills will be counted and your calendar reviewed. For this study, each six-week treatment period is called a cycle. Treatment will continue for nine cycles (54 weeks) as long as you are able to tolerate treatment and your cancer hasn’t returned. All treatment can be given without being admitted to a hospital.

If your cancer returns, your treatment may be unblinded by your doctor to find out if you were receiving everolimus or placebo. Your doctor may want this information to decide on additional treatment options for you. You or your insurance company will be responsible for the cost of treatment with everolimus if and when your doctor prescribes it for you off study. *(paragraph added 7/11/12)*

How long will I be in the study?

You will be asked to take the study drug for nine six-week cycles, or until your side effects become too great, or until your cancer returns. While you are receiving study treatment, you will

SWOG Authorized Document

need to come to the clinic for doctor visits every six weeks for the first 54 weeks including visits for scans every 18 weeks (or every 4 months). *(12/15/14)* After you are finished with the study treatment, you will return to the clinic with scans every six months for Years 2 and 3, and then yearly thereafter until 10 years after registration. *(12/15/14)*

Can I stop being in the study?

Yes. You can decide to stop at any time. Tell the study doctor if you are thinking about stopping or decide to stop. He or she will tell you how to stop safely. It is important to tell the study doctor if you are thinking about stopping so any risks from the study drug can be evaluated by your doctor. Another reason to tell your doctor that you are thinking about stopping is to discuss what follow-up care and testing could be most helpful for you.

The study doctor may stop you from taking part in this study at any time if he/she believes it is in your best interest; if you do not follow the study rules; or if the study is stopped.

What side effects or risks can I expect from being in the study?

You may have side effects while on the study. Everyone taking part in the study will be watched carefully for any side effects. However, doctors don't know all the side effects that may happen. Side effects may be mild or very serious. Your health care team may give you medicines to help lessen side effects. Many side effects go away soon after you stop taking the study drug. In some cases, side effects can be serious, long lasting, or may never go away. There also is a risk of death.

You should talk to your study doctor about any side effects that you have while taking part in the study.

Risks and side effects related to everolimus/placebo include those which are: *(section updated 12/15/14, 5/6/14, 2/11/16, updated 6/6/16, 11/11/16) (10/3/17)*

**COMMON, SOME MAY BE SERIOUS**<br>In 100 people receiving Everolimus/Placebo, more than 20 and up to 100 may have:
• **Anemia which may require blood transfusion**<br>• **Diarrhea**<br>• **Sores in the mouth which may cause difficulty swallowing**<br>• **Tiredness**<br>• **Rash**

SWOG Authorized Document

**OCCASIONAL, SOME MAY BE SERIOUS**<br>In 100 people receiving everolimus, from 4 to 20 may have:
• **Pain**<br>• **Constipation, nausea, vomiting**<br>• **Swelling of the arms, legs**<br>• **Fever**<br>• **Infection, especially when white blood cell count is low**<br>• **Bruising, bleeding**<br>• **Weight loss, loss of appetite**<br>• **Changes in taste**<br>• **Headache**<br>• **Cough, shortness of breath**<br>• **Nose bleed**<br>• **Damage to the lungs which may cause shortness of breath**<br>• **Dry skin**<br>• **Itching**

**RARE, AND SERIOUS**<br>In 100 people receiving everolimus, 3 or fewer may have:
• **Non-healing surgical site**<br>• **Kidney damage which may require dialysis**<br>• **Allergic reaction which may cause rash, low blood pressure, wheezing, shortness of breath, swelling of the face or throat**

Reproductive risks: You should not become pregnant or father a baby while on this study and for at least 8 weeks following completion of therapy because the drugs in this study can affect an unborn baby. Women should not breastfeed a baby while on this study and for at least 8 weeks following completion of therapy. It is important you understand that you need to use birth control while on this study. Check with your study doctor about what kind of birth control methods to use and how long to use them. Some methods might not be approved for use in this study.

The study drug may interact with other medications. Cases of a type of pneumonia (caused by a fungus called Pneumocystis jirovecii), some with a fatal outcome, have been reported in patients who received everolimus. This type of pneumonia has been linked to the use of corticosteroids or other drugs that can weaken the immune system while using everolimus. Patients taking angiotensin converting enzyme (ACE) inhibitor therapy may be at an increased risk for swelling of the airways or tongue, with or without damage to the lungs. You should tell your study doctor about all medications (over the counter, herbal, and prescription) you are currently taking and check with your study doctor before beginning any new medications. *(Paragraph updated 12/15/14)*

Vaccines help protect people from certain illnesses. There is a chance that receiving everolimus could interfere with any vaccinations you receive. Some vaccines are made from live bacteria or live viruses. You cannot receive this kind of vaccine (for example FluMist™ or BCG) for seven days prior to going on study or during the study.

For more information about risks and side effects, ask your study doctor.

Are there benefits to taking part in the study?

Taking part in this study may or may not make your health better. While doctors hope the study drug will be more useful against cancer compared to the usual treatment, there is no proof of this yet. We do know that the information from this study will help doctors learn more about everolimus as a treatment for cancer. This information could help future cancer patients.

What other choices do I have if I do not take part in this study?

Your other choices may include:
- **Getting treatment or care for your cancer without being in a study**
- **Taking part in another study**
- **Getting no treatment**

Talk to your doctor about your choices before you decide if you will take part in this study.

Will my medical information be kept private?

We will do our best to make sure that the personal information in your medical record will be kept private. However, we cannot guarantee total privacy. Your personal information may be given out if required by law. If information from this study is published or presented at scientific meetings, your name and other personal information will not be used.

Organizations that may look at and/or copy your medical records for research, quality assurance, and data analysis include:
- SWOG
- Qualified representative from Novartis Pharmaceuticals (manufacturer of everolimus)
- The National Cancer Institute (NCI) and other government agencies, like the Food and Drug Administration (FDA), involved in keeping research safe for people
- The Cancer Trials Support Unit (CTSU), a research group sponsored by the National Cancer Institute (NCI) to provide greater access to cancer trials.

A description of this clinical trial will be available on http://www.ClinicalTrials.gov, as required by U.S. law. *(12/15/14)* This Web site will not include information that can identify you. At most, the Web site will include a summary of the results. *(12/15/14)* You can search this Web site at any time. *(paragraph added 7/11/12)*

SWOG Authorized Document

[Note to Local Investigators: The NCI has recommended that HIPAA regulations be addressed by the local institution. The regulations may or may not be included in the informed consent form depending on local institutional policy.]

What are the costs of taking part in this study?

You and/or your health plan/ insurance company will need to pay for some or all of the costs of treating your cancer in this study. Some health plans will not pay these costs for people taking part in studies. Check with your health plan or insurance company to find out what they will pay for. Taking part in this study may or may not cost your insurance company more than the cost of getting regular cancer treatment.

Administration of the drug will be *(provided free of charge/charged in the usual way).* The parts of the research consisting of keeping research records will be paid by those organizing and conducting the research. The research requires that you receive certain standard medical tests and examinations. These standard tests and examinations will be *(charged in the usual way/provided at a reduced rate). (local institutions must choose the option that best fits the hospital's situation)*

Novartis Pharmaceuticals will supply the investigational agent everolimus or placebo at no charge while you take part in this study. Novartis Pharmaceuticals does not cover the cost of getting the everolimus or placebo ready and giving it to you, so you or your insurance company may have to pay for this.

Even though it probably won’t happen, it is possible that Novartis Pharmaceuticals may not continue to provide the everolimus or placebo for some reason. If this would occur, other possible options are:

1. You might be able to get the everolimus from your pharmacy but you or your insurance company may have to pay for it.

2. If there is no everolimus or placebo available at all, no one will be able to get more and the study would close.

If a problem with getting everolimus or placebo occurs, your study doctor will talk to you about these options. *(paragraph added 7/11/12)*

You will not be paid for taking part in this study.

For more information on clinical trials and insurance coverage, you can visit the National Cancer Institute’s Web site at http://cancer.gov/about-cancer/treatment/clinical-trials/paying/insurance. You can print a copy of the "Clinical Trials and Insurance Coverage" information from this Web site. *(revised 2/11/16)*

Another way to get the information is to call 1-800-4-CANCER (1-800-422-6237) and ask them to send you a free copy.

SWOG Authorized Document

What happens if I am injured because I took part in this study?

It is important that you tell your study doctor, __________________ *[investigator's name(s)]*, if you feel that you have been injured because of taking part in this study. You can tell the doctor in person or call him/her at __________________ *[telephone number]*.

You will get medical treatment if you are injured as a result of taking part in this study. You and/or your health plan will be charged for this treatment. The study will not pay for medical treatment.

What are my rights if I take part in this study?

Taking part in this study is your choice. You may choose either to take part or not to take part in the study. If you decide to take part in this study, you may leave the study at any time. No matter what decision you make, there will be no penalty to you and you will not lose any of your regular benefits. Leaving the study will not affect your medical care. You can still get your medical care from our institution.

We will tell you about new information or changes in the study that may affect your health or your willingness to continue in the study.

A Data Safety and Monitoring Board, an independent group of experts, will be reviewing the data from this research throughout the study. We will tell you about important new information from this or other studies that may affect your health, welfare, or willingness to stay in this study.

In the case of injury resulting from this study, you do not lose any of your legal rights to seek payment by signing this form.

Who can answer my questions about the study?

You can talk to your study doctor about any questions or concerns you have about this study. Contact your study doctor __________________ *[name(s)]* at __________________ *[telephone number]*.

For questions about your rights while taking part in this study, call the _________________________ *[name of center]* Institutional Review Board (a group of people who review the research to protect your rights) at __________________ *(telephone number)*. *[Note to Local Investigator: Contact information for patient representatives or other individuals in a local institution who are not on the IRB or research team but take calls regarding clinical trial questions can be listed here.]*

*You may also call the Operations Office of the NCI Central Institutional Review Board (CIRB) at 888-657-3711 (from the continental US only). *[*Only applies to sites using the CIRB.]*

(paragraph deleted 7/11/12)

SWOG Authorized Document

Please note: This section of the informed consent form is about additional research studies that are being done with people who are taking part in the main study. You may take part in these additional studies if you want to. You can still be a part of the main study even if you say 'no' to taking part in any of these additional studies.

You can say "yes" or "no" to each of the following studies. Please mark your choice for each study.

(paragraph deleted 7/11/12)

Future Contact
I agree to allow my study doctor, or someone approved by my study doctor, to contact me regarding future research involving my participation in this study.

Yes **No**

Consent Form for Use of Specimens for Research

About Using Specimens for Research

We would like to keep some specimens for future research. *(7/11/12)* If you agree, these specimens will be kept and may be used in research to learn more about cancer and other diseases. Please read the information sheet called "How are Specimens Used for Research" to learn more about specimen research. *(paragraph moved up 7/11/12)*

If you agree, a sample of your blood and tumor tissue will be banked for future research to study the biology of your cancer and how everolimus works. *(7/11/12)* The blood samples will be obtained to measure the level of everolimus in your blood. We will see if those levels correspond to the risk of getting certain side effects. In the tumor samples we will measure the levels of certain proteins to see if they predict how well everolimus will work. The blood sample (about 3 ½ teaspoons) will be collected before you begin study treatment, before Cycles 2 and 3, when you go off protocol treatment, and if your cancer recurs. The blood sample must be taken before your daily dose of study drug. The tumor tissue sample will be taken from your kidney cancer specimen (obtained from your recent operation). (You will not need to have another surgery for this purpose.) These collections are optional. *(sentences deleted 7/11/12)*

(section deleted 7/11/12)

The research that may be done with your specimens is not designed specifically to help you. It might help people who have cancer and other diseases in the future.

Reports about research done with your specimens will not be given to you or your doctor. These reports will not be put in your health record. The research will not have an effect on your care.

Things to Think About

SWOG Authorized Document

The choice to let us keep the left over specimens for future research is up to you. No matter what you decide to do, it will not affect your care.

If you decide now that your specimens can be kept for research, you can change your mind at any time. Just contact us and let us know that you do not want us to use your specimens. Then any specimens that remain will no longer be used for research.

In the future, people who do research may need to know more about your health. While SWOG may give them reports about your health, it will not give them your name, address, phone number, or any other information that will let the researchers know who you are.

Sometimes specimens are used for genetic research (about diseases that are passed on in families). Even if your specimens are used for this kind of research, the results will not be put in your health records.

If your confidential genetic information is discovered, you may suffer from genetic discrimination. Genetic discrimination occurs if people are treated unfairly because of differences in their genes that increase their chances of getting a certain disease. In the past, this could have resulted in the loss of health insurance or employment. Because of this, The Genetic Information Nondiscrimination Act of 2008, also referred to as GINA, was passed by Congress to protect Americans from such discrimination. The new law prevents discrimination from health insurers and employers. This act was signed into federal law on May 21, 2008, and went into effect May 2009. This law does not cover life insurance, disability insurance and long-term care insurance. *(paragraph added 7/11/12)*

While this study has safeguards in place to protect your confidential genetic information and to make it extremely unlikely that your identity would be connected with any special studies that are performed on your tissue, it is possible that this information could be discovered by someone who is unauthorized to have access to it. *(paragraph added 7/11/12)*

Your specimens will be used only for research and will not be sold. The research done with your specimens may help to develop new products in the future.

Benefits

The benefits of research using specimens include learning more about what causes cancer and other diseases, how to prevent them, and how to treat them.

SWOG Authorized Document

Risks

The greatest risk to you is the release of information from your health records. We will do our best to make sure that your personal information will be kept private. The chance that this information will be given to someone else is very small.

Making Your Choice

Please read each sentence below and think about your choice. After reading each sentence, circle "Yes" or "No." If you have any questions, please talk to your doctor or nurse, or call our research review board at IRB's phone number.

Making Your Choice

Please read each sentence below and think about your choice. After reading each sentence, circle "Yes" or "No." If you have any questions, please talk to your doctor or nurse, or call our research review board at IRB's phone number.

No matter what you decide to do, it will not affect your care.

1. **My specimens may be kept for use in research to learn about, prevent, treat or cure cancer.**

   Yes No

2. **My specimens may be kept for use in research about other health problems (for example: diabetes, Alzheimer's disease, or heart disease).**

   Yes No

3. **Someone may contact me in the future to ask me to allow other uses of my specimens.**

   Yes No

If you decide to withdraw your specimens from a SWOG Specimen Repository in the future, a written withdrawal of consent should be submitted through your study doctor to the SWOG Operations Office. Please designate in the written withdrawal whether you would prefer to have the specimens destroyed or returned to the study doctor.

Where can I get more information?

You may call the National Cancer Institute's Cancer Information Service at:

1-800-4-CANCER (1-800-422-6237) *(revised 2/11/16)*

You may also visit the NCI Web site at http://cancer.gov/

SWOG Authorized Document

- For NCI's clinical trials information, go to: http://cancer.gov/clinicaltrials/
- For NCI's general information about cancer, go to http://cancer.gov/cancerinfo/

You will get a copy of this form. If you want more information about this study, ask your study doctor.

Signature

I have been given a copy of all _____ *[insert total of number of pages]* pages of this form. I have read it or it has been read to me. I understand the information and have had my questions answered. I agree to take part in this study.

Participant ______________________________

Date ___________________________________

SWOG Authorized Document

Specimen Consent Supplemental Sheets

How are Specimens Used for Research?

Where do specimens come from?

A specimen may be from a blood sample or from bone marrow, skin, toenails or other body materials. People who are trained to handle specimens and protect donors' rights make sure that the highest standards of quality control are followed by SWOG. Your doctor does not work for SWOG, but has agreed to help collect specimens from many patients. Many doctors across the country are helping in the same way.

Why do people do research with specimens?

Research with specimens can help to find out more about what causes cancer, how to prevent it, how to treat it, and how to cure it. Research using specimens can also answer other health questions. Some of these include finding the causes of diabetes and heart disease, or finding genetic links to Alzheimer's.

What type of research will be done with my specimen?

Many different kinds of studies use specimens. Some researchers may develop new tests to find diseases. Others may develop new ways to treat or even cure diseases. In the future, some of the research may help to develop new products, such as tests and drugs. Some research looks at diseases that are passed on in families (called genetic research). Research done with your specimen may look for genetic causes and signs of disease.

How do researchers get the specimen?

Researchers from universities, hospitals, and other health organizations conduct research using specimens. They contact SWOG and request samples for their studies. SWOG reviews the way that these studies will be done, and decides if any of the samples can be used. SWOG gets the specimen and information about you from your hospital, and sends the specimen samples and some information about you to the researcher. SWOG will not send your name, address, phone number, social security number or any other identifying information to the researcher.

Will I find out the results of the research using my specimen?

You will not receive the results of research done with your specimen. This is because research can take a long time and must use specimen samples from many people before results are known. Results from research using your specimen may not be ready for many years and will not affect your care right now, but they may be helpful to people like you in the future.

Why do you need information from my health records?

In order to do research with your specimen, researchers may need to know some things about you. (For example: Are you male or female? What is your race or ethnic group? How old are you? Have you ever smoked?) This helps researchers answer questions about diseases. The information that will be given to the researcher may include your age, sex, race, diagnosis, treatments and family history. This information is collected by your hospital from your health record and sent to SWOG. If more information is needed, SWOG will send it to the researcher.

Will my name be attached to the records that are given to the researcher?

No. Your name, address, phone number and anything else that could identify you will be removed before they go the researcher. The researcher will not know who you are.

SWOG Authorized Document

How could the records be used in ways that might be harmful to me?

Sometimes, health records have been used against patients and their families. For example, insurance companies may deny a patient insurance or employers may not hire someone with a certain illness (such as AIDS or cancer). The results of genetic research may not apply only to you, but to your family members too. For disease caused by gene changes, the information in one person's health record could be used against family members.

How am I protected?

SWOG is in charge of making sure that information about you is kept private. SWOG will take careful steps to prevent misuse of records. Your name, address, phone number and any other identifying information will be taken off anything associated with your specimen before it is given to the researcher. This would make it very difficult for any research results to be linked to you or your family. Also, people outside the research process will not have access to results about any one person which will help to protect your privacy.

What if I have more questions?

If you have any questions, please talk to your doctor or nurse, or call our research review board at (Insert IRB's Phone Number).

SWOG Authorized Document
