# Supplementary material for: Adjuvant Everolimus in Non–Clear Cell Renal Cell Carcinoma: A Secondary Analysis of a Randomized Clinical Trial
Source: JAMA Netw Open. 2024 Aug 6;7(8):e2425288. doi: 10.1001/jamanetworkopen.2024.25288 (PMC11304111; doi:10.1001/jamanetworkopen.2024.25288)
Supplement: Supplement 2. — eTable 1. Reasons to Go Off-Study in Each Cohort Analyzed eFigure. Patient Enrollment Flowchart eTable 2. Duration of Treatment in Each Cohort for Those Who Stopped Treatment Early [file jamanetwopen-e2425288-s002.pdf]

Supplemental Online Content

Gulati S, Tangen C, Ryan CW, et al. Adjuvant everolimus in non–clear cell renal cell carcinoma: a secondary analysis of a randomized clinical trial. *JAMA Netw Open*. 2024;7(8):e2425288. doi:10.1001/jamanetworkopen.2024.25288

eTable 1. Reasons to Go Off-Study in Each Cohort Analyzed

eFigure. Patient Enrollment Flowchart

eTable 2. Duration of Treatment in Each Cohort for Those Who Stopped Treatment Early

This supplemental material has been provided by the authors to give readers additional information about their work.

© 2024 Gulati S et al. *JAMA Network Open*.

eTable 1. Reasons for going off-study in each cohort analyzed

Reason Off Treatment	Clear Cell		Papillary		Chromophobe	
	Everolimus (n=626)	Placebo (n=622)	Everolimus (n=57)	Placebo (n=52)	Everolimus (n=53)	Placebo (n=46)
**Completed as Planned**	282	430	26	37	26	34
**AE or Side Effect**	233	34	18	1	21	1
**Refusal Unrelated to AE**	47	42	5	6	4	4
**Recurrence**	48	88	4	8	1	5
**Death**	0	0	0	0	0	1
**Other (not protocol specified)**	16	28	4	0	1	1

© 2024 Gulati S et al. *JAMA Network Open*.

Supplemental figure 1

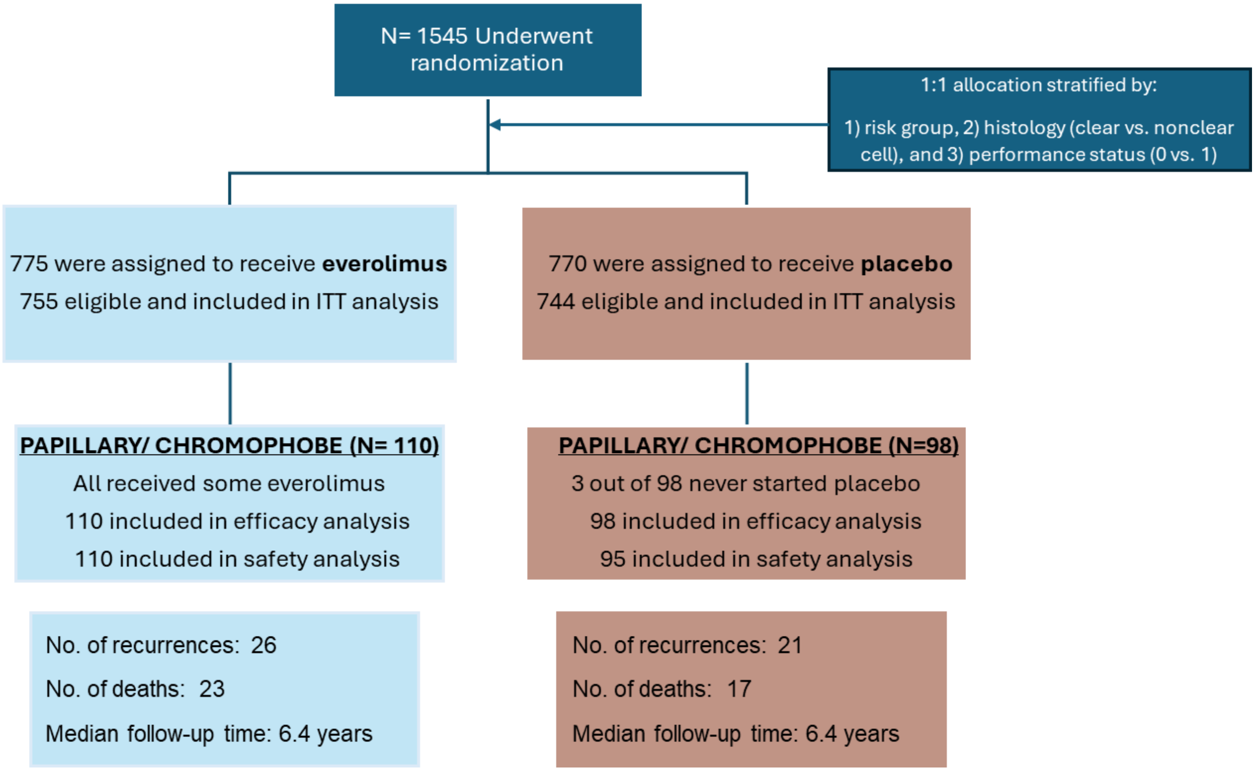

© 2024 Gulati S et al. *JAMA Network Open*.

eTable 2. Duration of treatment in each cohort for those who stopped treatment early

Duration of Treatment	Clear Cell		Papillary		Chromophobe	
	Everolimus (n=344)	Placebo (n=192)	Everolimus (n=31)	Placebo (n=15)	Everolimus (n=27)	Placebo (n=12)
**0-3 months**	166 (48%)	53 (28%)	13 (42%)	5 (33%)	12 (44%)	4 (33%)
**3-6 months**	101 (29%)	64 (33%)	9 (29%)	6 (40%)	8 (30%)	5 (42%)
**6- months and prior to 54 weeks**	77 (22%)	75 (39%)	9 (29%)	4 (27%)	7 (26%)	3 (25%)

© 2024 Gulati S et al. *JAMA Network Open*.
