# Supplementary material for: Adjuvant Everolimus in Non–Clear Cell Renal Cell Carcinoma: A Secondary Analysis of a Randomized Clinical Trial
Source: JAMA Netw Open. 2024 Aug 6;7(8):e2425288. doi: 10.1001/jamanetworkopen.2024.25288 (PMC11304111; doi:10.1001/jamanetworkopen.2024.25288)
Supplement: Supplement 3. — Data Sharing Statement [file jamanetwopen-e2425288-s003.pdf]

Data Sharing Statement

Gulati. Adjuvant Everolimus in Non–Clear Cell Renal Cell Carcinoma. *JAMA Netw Open*. Published August 06, 2024. doi:10.1001/jamanetworkopen.2024.25288

Data
Data available: Yes
Data types: Data (not involving human participants)
How to access data: Deidentified data can be made available upon request
ctangen@fredhutch.org
When available: With publication

Supporting Documents
Document types: None

Additional Information
Who can access the data: researchers whose proposed use of the data has been approved
Types of analyses: for any purpose
Mechanisms of data availability: after approval of a proposal, and with a signed data access agreement
